# Synthesis of *N*-Alkyl-1,3-dihydro-2,1-benzisoxazoles

**DOI:** 10.1021/acs.orglett.4c03509

**Published:** 2024-11-05

**Authors:** Thomas D. Beckler, David Crich

**Affiliations:** † Department of Chemistry, University of Georgia, Athens, Georgia 30602, United States; ‡ Department of Pharmaceutical and Biomedical Sciences, University of Georgia, Athens, Georgia 30602, United States; § Complex Carbohydrate Research Center, University of Georgia, Athens, Georgia 30602, United States

## Abstract

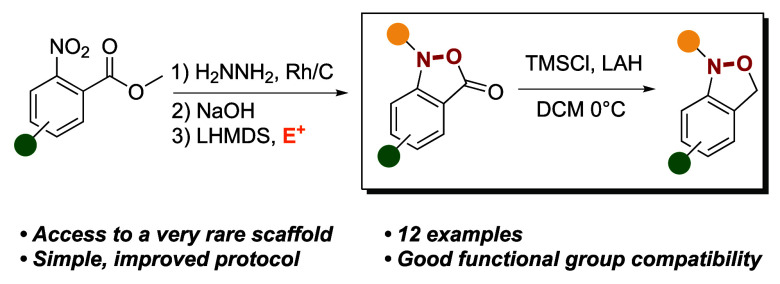

We describe a practical method for the synthesis of various substituted *N*-alkyl-1,3-dihydro-2,1-benzisoxazoles and their 2,1-benzisoxazolone precursors starting from readily available methyl 2-nitrobenzoates. The method entails partial nitro reduction with hydrazine and rhodium on carbon to give the hydroxylamines, followed by base-mediated cyclization to give the corresponding benzisoxazol-3(1*H*)-ones. Subsequent alkylation is conducted under basic conditions and is followed by reduction to the target 1,3-dihydrobenzisoxazoles, achieved with lithium aluminum hydride in the presence of trimethylsilyl chloride.

The isoxazolidine motif is a privileged scaffold in medicinal chemistry^1^ and is present in a wide variety of natural products that exhibit cytotoxic, antifungal, or antimicrobial activity, such as zetekitoxin AB derivatives,^2^ alsmaphorazines A and B,^3,4^ and pyrinodemin A.^5^ Of similar interest are the benzo-fused derivatives of isoxazolidines, benzisoxazoles, as well as their benzisoxazolone derivatives, which were previously investigated for their antibacterial properties.^6,7^ Indeed, the scaffold has received interest because of its presence in biologically active natural products such as parnafungin A and B,^8^ as well as in a series of Pim-1 and -2 kinase inhibitors.^9,10^ The wide range of applications and low acute toxicity of these compounds^11^ suggests the potential of such scaffolds for broader application in medicinal chemistry. Adding additional interest to these scaffolds is the embedded trisubstituted hydroxylamine moiety, which we have been developing as a bioisostere in medicinal chemistry.^12−15^ While much has been reported on the properties of 2,1-benzisoxazolones, existing methods for their synthesis suffer from poor yields, require the use of complex starting materials, or lack efficiency.^7,16−19^ The 1,3-dihydro-2,1-benzisoxazoles remain, to the best of our knowledge, all but unknown.^20,21^ Herein, we report a simple three step approach to generate 2,1-benzisoxazolones, and their subsequent reduction to 1,3-dihydro-2,1-benzisoxazoles in a single step.

For the synthesis of the benzisoxazolones, we initially employed the protocol of Wierenga et al.^7^ in which the partial reduction/cyclization of *o*-nitrobenzoates was carried out with zinc and ammonium chloride. While this protocol was suitable for forming the hydroxylamine **2**, cyclization was nontrivial and required an additional base-mediated step resulting in some decomposition as well as competitive dimerization to the azoxy species **5** (Scheme 1). More importantly, competing over-reduction of the nitro group to the aniline **4** could not be suppressed, resulting in diminished yields and complex reaction mixtures (Scheme 1). Attempted application of similar protocols for nitro reduction^22^ were marginally more successful, but competing over-reduction was still difficult to suppress. Ultimately, we turned to the use of Rh/C and hydrazine,^23^ which afforded the primary hydroxylamines cleanly and in high yield with little to no over-reduction observed.

**Scheme 1:**
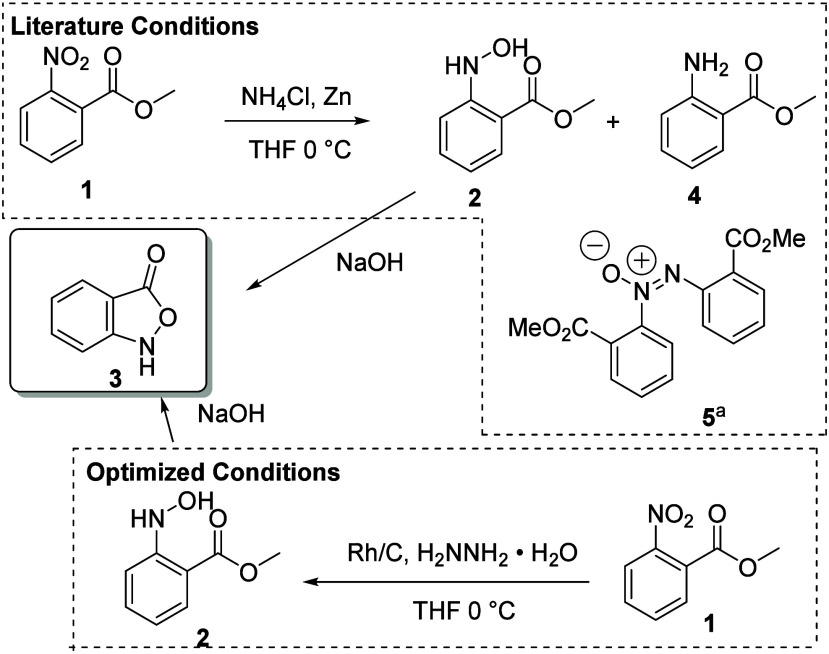
Literature and Optimized Conditions for 2,1-Benzisoxazolone Formation Product observed by mass spectrometry.

By application of the Rh/C and hydrazine conditions, the intermediate hydroxylamines **2** could be readily obtained by simply washing the crude reaction mixtures from the reduction step with 1 M NaOH solution^24^ which yielded the 2,1-benzisoxazol-3(1*H*)-ones **3** in a simple one-pot, two-step procedure. These cyclic *O*-acyl *N*-aryl hydroxylamines were found to be mildly unstable and underwent decomposition under ambient conditions over a period of 2–3 days. Thus, without purification, the crude cyclized products **3** were immediately subjected to alkylation under basic conditions, generating the targeted *N*-alkyl benzisoxazol-3(1*H*)-ones **6**–**17** in moderate to good yields over three steps. While we focused on the use of benzylic and allylic halides as electrophiles in the alkylation step, a primary alkyl iodide was also found to be suitable, giving **17** in fair overall yield (Table 1). Unfortunately, to date we have not been able to obtain useful yields with secondary alkyl halides. The procedure was amenable to multigram scale (**7**, **12**, **14**, and **15**) without significant reduction in yield and was compatible with electron withdrawing (**9**–**13** and **15**–**17**), and donating substituents on the arene (**8** and **14**). This efficient protocol features short reaction times, mild conditions and requires only a single chromatographic purification. In terms of limitations, with the exception of fluoride **10**, 3-substituted-2-nitrobenzoate esters were generally incompatible with the protocol as they underwent premature cyclization to isoxazolones during the reduction step, perhaps due to a favorable conformational restriction of the intermediate hydroxylamine, followed by over-reduction to the anthranilic acids and/or nucleophilic ring opening by the excess hydrazine.

**Table 1:**
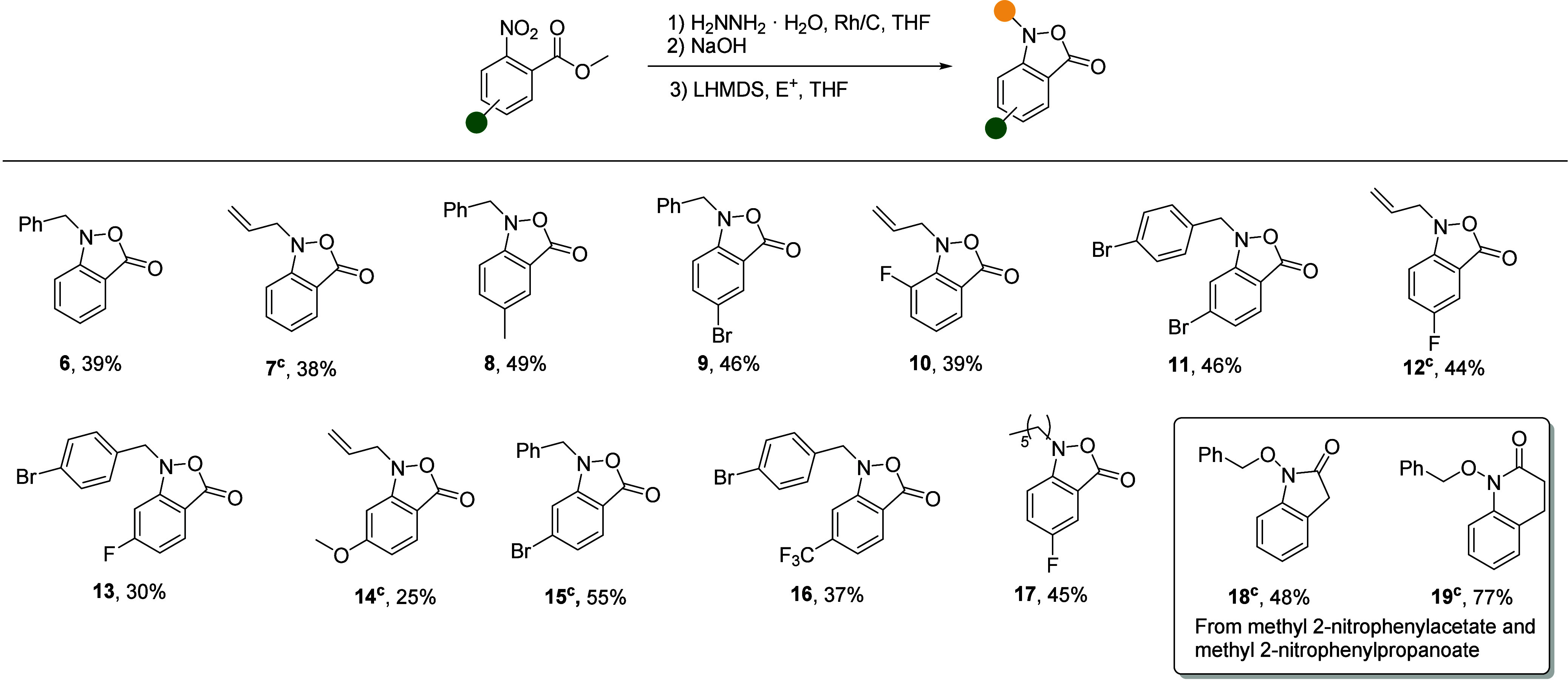
Scope of 2,1-Benzisoxazolone Synthesis^a^,^b^

a Yields reported over three steps, after isolation.

b All reactions conducted on 0.6–0.8 mmol scale unless stated otherwise.

c Reaction conducted on >2 mmol scale.

Attempted extrapolation of the protocol to generate benzo-fused 6- and 7-membered cyclic *O*-acyl *N*-aryl hydroxylamines was unsuccessful owing to the predominance of cyclization on the nitrogen atom of the ambident nucleophilic hydroxylamines, resulting in the formation of **18** and **19** after final alkylation (Table 1).

Turning to the synthesis of the dihydrobenzisoxazoles, we first attempted application of the Rychnovsky ether synthesis protocol,^25^ with an initial partial reduction with DIBALH followed by acetylation of the tetrahedral intermediate, and then a second Lewis acid mediated reduction step (Scheme 2), as previously applied successfully to the synthesis of acyclic trisubstituted hydroxylamines from *O*-acyl-*N*,*O*-disubstituted hydroxylamines.^26^ Unfortunately, the application of this method to the present cyclic *O*-acyl hydroxylamines was unsuccessful, seemingly owing to instability of the intermediate acetoxyisoxazolidines **20** under the initial reduction conditions, leading us to screen other reduction methods, which mostly resulted in N–O bond cleavage (see Table S1).

**Scheme 2:**
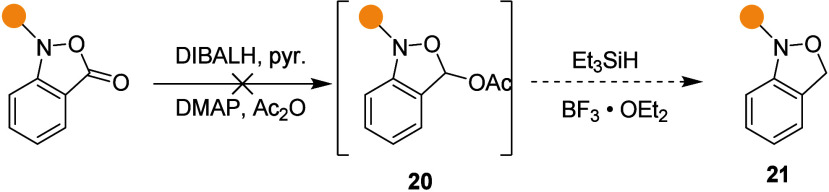
Failed Extrapolation of Reduction Conditions for Acyclic *O*-Acyl Hydroxylamines to the Cyclic Series

Ultimately, we reasoned that silylation of the tetrahedral intermediate from a first reduction step would prevent a ring opening collapse with subsequent over-reduction and provide a siloxy isoxazoline **22** for a second, product-forming reduction step. Reduction with lithium aluminum hydride (LAH, 1.4 equiv) in the presence of trimethylsilyl chloride (TMSCl, 1.2 equiv) in dichloromethane at −10 °C was found to afford the desired product **21**, albeit in poor yield, along with the hydroxymethyl aniline derivative **23** (Table 2, entry 1). Subsequent variation of stoichiometry, temperature, and Lewis acid (Table 2) led us to the optimal conditions of 2.5 equiv each of TMSCl and LAH in dichloromethane at 0 °C, which resulted in the isolation of the desired benzisoxazoline in 58% yield (Table 2, entry 7).

**Table 2:**
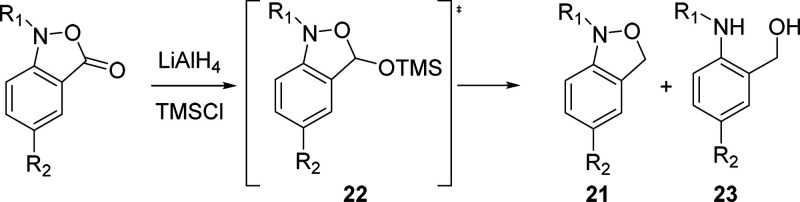
Optimization of the Isoxazolone Reduction

entry	conditions	**20** (yield %)^a^	**23** (yield %)^a^
1^d^	TMSCl (1.2 equiv), LAH (1.4 equiv), DCM, –10 °C	13	8
2^e^	TMSCl (2.5 equiv), LAH (2.1 equiv), DCM, –78 °C	52	16
3^e^	TMSCl (5 equiv), LAH (2.5 equiv), DCM, –78 °C	47	42
4^e^	BF_3_·OEt_2_ (2.5 equiv), LAH (2.5 equiv), DCM, –78 °C	4	trace
5^e^	TMSCl (2.5 equiv), DIBALH (2.5 equiv), DCM, –78 °C	b	12
6^f^	TMSOTf (2.5 equiv), LAH (2.5 equiv), DCM, 0 °C	40	c
7^e^	TMSCl (2.5 equiv), LAH (2.5 equiv), DCM, 0 °C	58	15

a All yields reported after isolation.

b Product not detected.

c Product was not isolated.

d R_1_ = Allyl, R_2_ = H.

e R_1_ = Bn, R_2_ = H.

f R_1_ = Bn, R_2_ = Me.

The optimized reduction conditions were found to be compatible with aryl fluorides (**28**, **30**, **31**, and **35**) and, notably, aryl bromides (**27**, **29**, **31**, **33**, and **34**), which are particularly susceptible to protodehalogenation by LAH,^27^ as well as electron-donating groups (**26** and **32**) and the strongly electron-withdrawing trifluoromethyl group (**34**). Additionally, the reduction was applicable on scales exceeding 2.9 mmol without a significant loss in yield. Overall, this methodology provides easy access to a diverse range of the all but unknown 1,3-dihydro-2,1-benzisoxazoles (Table 3) in fair to good yield.

**Table 3:**
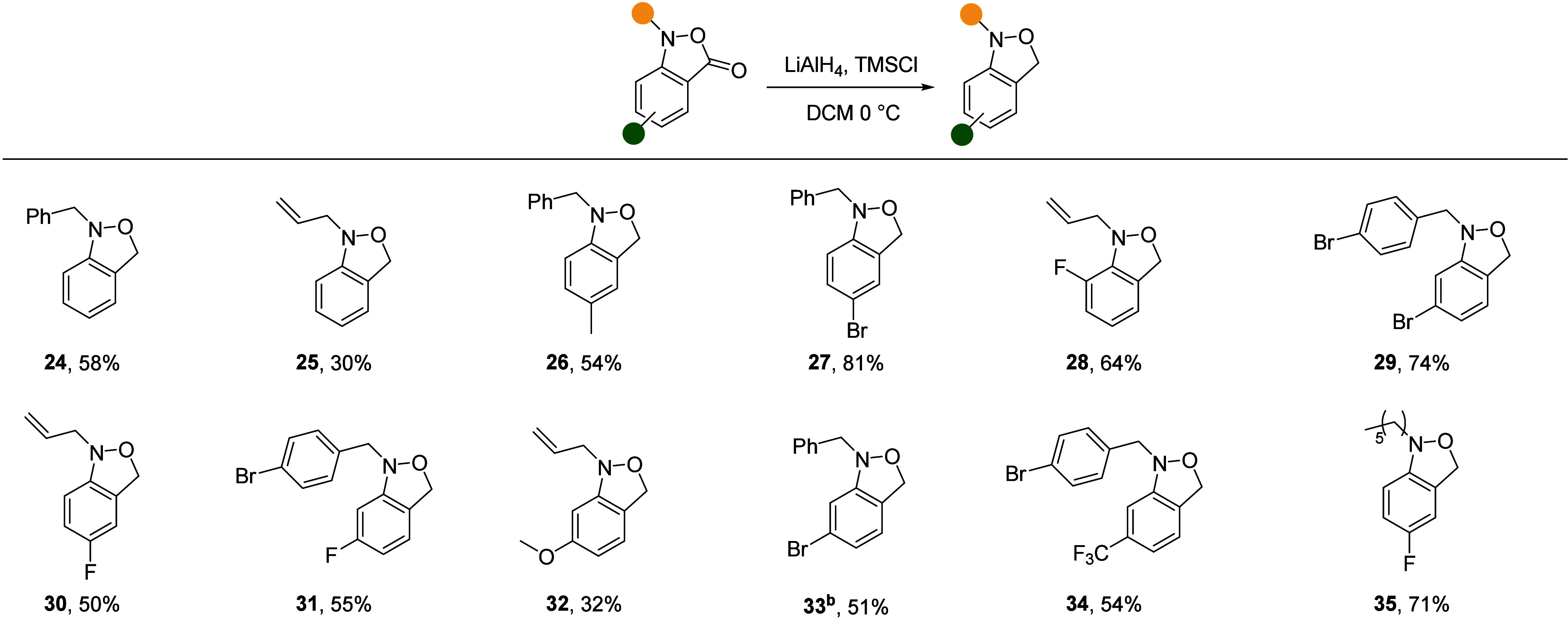
Scope of *N*-Alkyl 1,3-dihydro-2,1-benzisoxazole Synthesis^a^

a Yields reported after isolation.

b Conducted on >2.0 mmol scale.

The accessibility of the bromo-substituted 1,3-dihydro-2,1-benzisoxazole **33** provided the opportunity to assess the compatibility of the trisubstituted hydroxylamine function with typical palladium-catalyzed reactions practiced in drug discovery programs.^28−30^ To this end, **33** was heated for 3 h to 90 °C in 1,4-dioxane/water with phenylboronic acid and K_3_PO_4_ employing 5 mol % SPhos Pd G3 precatalyst, yielding after chromatographic purification the Suzuki product **36** in 74% yield (Scheme 3). Similarly, heating of **33** with morpholine and NaO*t*Bu in toluene for 2.5 h in the presence of 10 mol % SPhos Pd G3 afforded the Buchwald–Hartwig product **37** in 53% isolated yield (Scheme 3). Thus, Pd(0)-catalyzed coupling reactions may now be added to the already wide repertoire^12,13,15,26,34,35^ of oxidative, reductive, and redox neutral reactions compatible with trisubstituted hydroxylamines. These demonstrative reactions also serve to underline the difference in reactivity between the relatively apolar trisubstituted hydroxylamine moiety and the *O*-benzoyl-*N*,*N*-disubstituted hydroxylamines that are so widely employed as electrophilic nitrogen sources under a variety of transition metal and photocatalytic reactions conditions.^31−33^

**Scheme 3:**
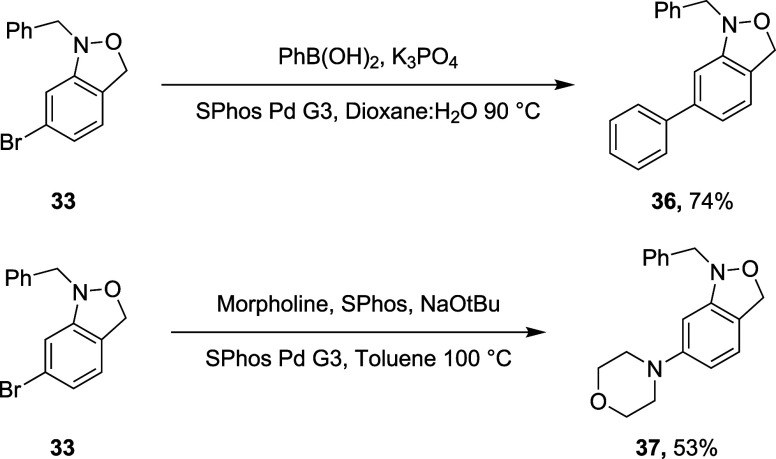
Compatibility of the 1,3-Dihydro-2,1-benzisoxazole Moiety with the Suzuki and Buchwald–Hartwig Reactions

Turning to structural features, a characteristic of the acyclic trisubstituted hydroxylamine moiety in general is its low barrier to pyramidal inversion at nitrogen and to rotation about the N–O bond, prompting us to employ them as adaptable achiral replacements of chiral centers.^34,35^ The 2,1-benzisoxazolone **15** and the 1,3-dihydro-2,1-benzisoxazole **31** were crystalline and amenable to X-ray crystallographic structure determination (Figure 1), which revealed the nitrogen atoms to be highly pyramidal with sums of bond angles at nitrogen of 334.6° and 324.6°,^36,37^ respectively, the former being consistent with an earlier structure.^38^ Consequently, neither fusion to the aromatic ring at either oxidation level nor conjugation of the oxygen lone pairs with the carbonyl group in **15** overcomes the fundamental preference of the hydroxylamine function for sp^3^ hybridization at nitrogen and the minimization of repulsion between the lone pairs on oxygen and nitrogen. A key feature in the use of trisubstituted hydroxylamines as adaptable replacements of chiral centers is the low barrier to nitrogen inversion, which is approximately 15 kcal/mol for the acyclic trialkylhydroxylamines, approximately 15 kcal/mol for saturated isoxazolidines, and approximately 13 kcal/mol for the *N*-alkoxypyrrolidines.^39−41^ With this in mind, we studied the dihydrobenzisoxazole **24** by variable temperature (VT) ^1^H and ^13^C NMR spectroscopy in CD_2_Cl_2_ (see SI) but did not observe significant peak broadening down to −80 °C, the lowest temperature we investigated. It is apparent, therefore, that while benzo-fusion does not change the pyramidal nature of the nitrogen atom in these compounds at the ground state, there is a minimal barrier to inversion due both to the delocalization of the nitrogen lone pair onto the arene at the transition state and to the low barrier to pseudorotation in the five-membered ring, the latter of which is already apparent in the reduced barrier to inversion in the *N*-alkoxypyrrolidines as compared to that of their six-membered counterparts and the acyclic hydroxylamines.^39−41^ Accordingly, we anticipate that the *N*-substituted benzisoxazolones may find application as achiral analogs of 7-substituted isobenzofuranones, paralleling an earlier suggestion by Snyder,^6^ while the dihydrobenzisoxazoles may serve as achiral surrogates of 2-susbtituted dihydro-isobenzofurans, consistent with our application of this general concept in the acyclic series.^12,34^

**Figure 1:**
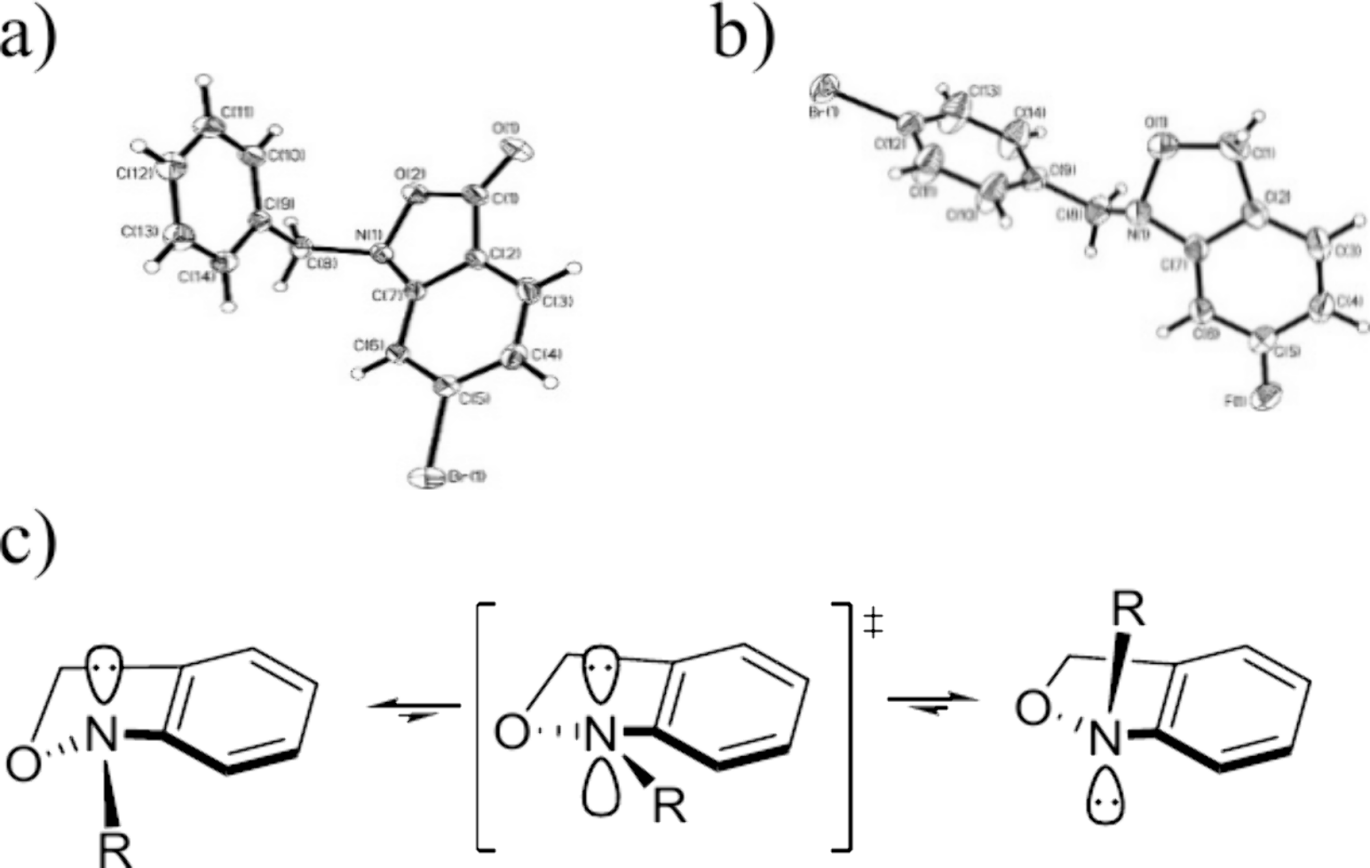
ORTEP diagrams of (a) 2,1-benzisoxazolone **15** (CCDC 2359049) and (b) 1,3-dihydro-2,1-benzisoxazole **31** (CCDC 2359050) and (c) nitrogen inversion via a planar transition state.

Overall, *N*-alkyl-1,3-dihydro-2,1-benzisoxazoles and their 2,1-benzisoxazolone precursors can be synthesized in a straightforward manner from readily available substituted methyl 2-nitrobenzoates. The method entails partial nitro group reduction to the hydroxylamines with Rh/C and hydrazine, followed by cyclization to give the corresponding benzisoxazol-3(1*H*)-ones and finally base-mediated alkylation. Subsequent reduction to the target 1,3-dihydro-2,1-benzisoxazoles is achieved with LAH and TMSCl. This methodology tolerates the presence of both strongly electron-donating and -withdrawing arene substituents. Ultimately, this protocol provides easy access to a diverse range of 2,1-benzisoxazolones and the all but unknown 1,3-dihydro-2,1-benzisoxazoles, with potential applications in medicinal chemistry.

## Data Availability

The data underlying this study are available in the published article and its Supporting Information.

## Supporting Information Available

Detailed experimental procedures, spectroscopic characterizations of all new compounds, VT NMR studies of **23**, and crystallographic data of **15** and **31** (PDF)
