# Supplementary material for: Synthesis of N-Alkyl-1,3-dihydro-2,1-benzisoxazoles
Source: Org Lett. 2024 Nov 5;26(45):9722–7. doi: 10.1021/acs.orglett.4c03509 (PMC11574845; doi:10.1021/acs.orglett.4c03509)
Supplement: Supplementary file 1 — ol4c03509_si_001.pdf [file ol4c03509_si_001.pdf]

SUPPORTING INFORMATION

Synthesis of *N*-Alkyl-1,3-dihydro-2,1-benzisoxazoles

Authors: Thomas D. Beckler[1,2] and David Crich.[1,2,3*]

1. Department of Biomedical and Pharmaceutical Sciences, University of Georgia, 250 West Green Street, Athens, GA 30602, USA.

2. Department of Chemistry, University of Georgia, 302 East Campus Road, GA 30602, USA.

3. Complex Carbohydrate Research Center, University of Georgia, 315 Riverbend Road, Athens, GA, 30602, USA.

*Correspondence to: david.crich@uga.edu

**Compound**	**Expt.**	**Spectra**
General Experimental	S4	-
Table S1 – Unsuccessful Reduction Methods	S6	-
General Procedures	S6-S7	-
1-Benzylbenzo[c]isoxazol-3(*1H*)-one (**6**)	S8	S45-46
1-Allylbenzo[*c*]isoxazol-3(*1H*)-one (**7**)	S9	S47-50
1-Benzyl-5-methylbenzo[c]isoxazol-3(*1H*)-one (**8**)	S10	S51-52
1-Benzyl-5-bromobenzo[*c*]isoxazol-3(*1H*)-one (**9**)	S11	S53-54
1-Allyl-7-fluorobenzo[c]isoxazol-3(*1H*)-one (**10**)	S12-13	S55-59
6-Bromo-1-(4-bromobenzyl)benzo[c]isoxazol-3(*1H*)-one (**11**)	S14	S60-61
1-Allyl-5-fluorobenzo[c]isoxazol-3(*1H*)-one (**12**)	S15-16	S62-66
1-(4-Bromobenzyl)-6-fluorobenzo[c]isoxazol-3(*1H*)-one (**13**)	S17-18	S67-71
1-Allyl-6-methoxybenzo[c]isoxazol-3(*1H*)-one (**14**)	S19	S72-73
1-Benzyl-6-bromobenzo[*c*]isoxazol-3(*1H*)-one (**15**)	S20	S74-75
1-(4-Bromobenzyl)-6-(trifluoromethyl)benzo[c]isoxazol-3(*1H*)-one (**16**)	S21-22	S76-80
5-Fluoro-1-hexylbenzo[*c*]isoxazol-3(*1H*)-one (**17**)	S23-24	S81-85
1-(Benzyloxy)indolin-2-one (**18**)	S25	S86-89
1-(Benzyloxy)-3,4-dihydroquinolin-2(*1H*)-one (**19**)	S26	S90-93
1-Benzyl-1,3-dihydrobenzo[c]isoxazolse (**24**)	S27	S94-95
1-Allyl-1,3-dihydrobenzo[*c*]isoxazole (**25**)	S28	S96-97
1-Benzyl-5-methyl-1,3-dihydrobenzo[c]isoxazole (**26**)	S29	S98-99
1-Benzyl-5-bromo-1,3-dihydrobenzo[c]isoxazole (**27**)	S30	S100-101

1-Allyl-7-fluoro-1,3-dihydrobenzo[c]isoxazole (**28**)	S31	S102-106
6-Bromo-1-(4-bromobenzyl)-1,3-dihydrobenzo[c]isoxazole (**29**)	S32	S107-108
1-Allyl-5-fluoro-1,3-dihydrobenzo[c]isoxazole (**30**)	S33-34	S109-113
1-(4-Bromobenzyl)-6-fluoro-1,3-dihydrobenzo[c]isoxazole (**31**)	S35-36	S114-118
1-Allyl-6-methoxy-1,3-dihydrobenzo[c]isoxazole (**32**)	S37	S119-120
1-Benzyl-6-bromo-1,3-dihydrobenzo[*c*]isoxazole (**33**)	S38	S121-122
1-(4-Bromobenzyl)-6-(trifluoromethyl)-1,3-dihydrobenzo[*c*]isoxazole (**34**)	S39-40	S123-127
5-Fluoro-1-hexyl-1,3-dihydrobenzo[c]isoxazole (**35**)	S41-42	S128-132
1-Benzyl-6-phenyl-1,3-dihydrobenzo[*c*]isoxazole (**36**)	S43	S133-134
1-Benzyl-6-morpholino-1,3-dihydrobenzo[*c*]isoxazole (**37**)	S44	S134-136
VT ^{1}H NMR (500 MHz, $CDCl_3$) spectrum of 1-benzylbenzo[*c*]isoxazol-3(*1H*)-one (**24**)	S137	-
VT ^{13}C NMR (126 MHz, $CDCl_3$) spectrum of 1-benzylbenzo[*c*]isoxazol-3(*1H*)-one (**24**)	S138	-
X-Ray Data of **15**	S139-147	-
X-Ray Data of **31**	S148-156	-
References	S157	-

General Experimental

All reactions were conducted in oven dried glassware capped with a rubber septum under an argon atmosphere unless otherwise stated. All organic solutions were concentrated under reduced pressure on a rotary evaporator and water bath. Flash-column chromatography was performed using silica gel (Sorbent Tech Purity Flash Cartridges Granular Silica Gel 60Å, 40-75µm) or alumina (Agilent SuperFlash SF10 125 µm) cartridges on a Teledyne Isco CombiFlash Next Gen 300+. Thin-layer chromatography (TLC) was carried out with 250 µm glass back silica (XHL) plates with fluorescent indicator (254 nm). TLC plates were visualized by exposure to ultraviolet light (UV) and/or submersion in ceric ammonium molybdate solution (CAM) followed by heating on a hot plate (120 °C, 10-15 s). For compounds that could not be detected on TLC using the above methods, the TLC plate was exposed to I_2.

Materials

Commercial solvents and reagents were used as received without further purification. LAH (1M in THF), Rh/C, and Chlorotrimethylsilane were purchased from Sigma-Aldrich. Hydrazine monohydrate was purchased from Sigma-Aldrich as a 65% solution. Methyl nitrobenzoates starting materials were purchased from Ambeed, Sigma-Aldrich, or AK Scientific.

Instrumentation

^{1}H and ^{13}C Nuclear magnetic resonance (NMR) spectra of all compounds were obtained at 500 and 126 MHz respectively, in CDCl3 (δ 7.26 and 77.16 ppm, respectively). Multiplicities are abbreviated as follows: s (singlet), multiplet (m), br (broad), d (doublet), t (triplet), q (quartet), p (pentet). High resolution mass spectra were obtained on an Orbitrap using electrospray ionization

(ESI). Melting points were determined via capillary melting point apparatus. IR spectra were recorded using Ge/KBr.

Table S1 – Unsuccessful Reduction Methods

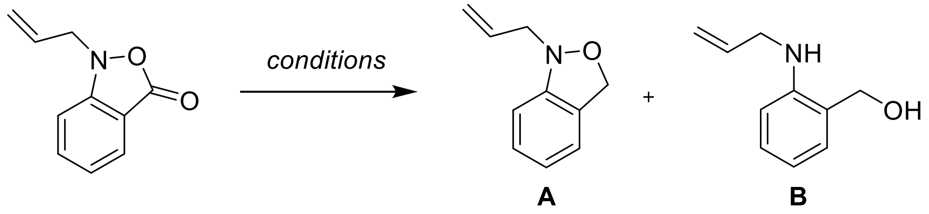

Entry	Condition	A yield (%)	B yield (%)
1	LAH, THF 60 °C	na	34%
2	LAH, THF rt	na	20%
3	DIBALH, THF 0 °C	trace	61%
4	DIBALH, THF rt	trace	48%
5	DIBALH, DCM 40 °C	na	7%
6	SuperHydride, THF rt	na	33%
7	Red-Al, THF rt	trace	10%

General Procedure A for the Synthesis of 2,1-Benzisoxazol-3(1*H*)-ones:

To a stirred 0.2M solution of methyl nitro benzoate (1 mmol, 1 eq) in anhydrous THF under Ar at 0 °C was added 5% Rh/C (0.05 mmol, 0.05 eq) followed by hydrazine monohydrate 65% (1.2 mmol, 1.2 eq) dropwise.[1] After full conversion was observed by TLC (1-3h), the solution was diluted in diethyl ether and filtered through Celite. The crude reaction mixture was washed with 1M NaOH 4 x 10mL per mmol and the resulting aqueous layer was then acidified to pH 5-6 with 1M HCl (5M HCl was used on scale) before being extracted with DCM 4 x 30 mL per mmol.[2] The organic layers were dried over Na_2SO_4, filtered, and concentrated *in vacuo*. The crude product was used in subsequent steps without further purification. **CAUTION:** While we have not experienced any issues, even on scale, hydrazine is a volatile, flammable, toxic, and potentially explosive reagent. Hydrazine should only be used within a fume hood with proper PPE and caution should be exercised when dispensing i.e. slow dropwise addition to prevent spills and runaway reactions.[3]

General Procedure B for the Alkylation of 2,1-benzisoxazlones:

To a stirred 0.05M solution of 2,1-benzisoxazol-3(1*H*)-one (1 mmol, 1 eq) in anhydrous THF under Ar at 0 °C was added LHMDS 1M in THF (1.2 mmol, 1.2 eq) dropwise followed by the corresponding alkyl bromide (1.2 mmol, 1.2 eq). The reaction was stirred until full consumption of starting material was observed via TLC, after which, the reaction was quenched via dropwise addition of 1M HCl. The crude mixture was further diluted with 1M HCl 15 mL per mmol and extracted with EtOAc 15 mL per mmol. The organic layer was subsequently washed with brine, dried over Na_2SO_4, filtered, and concentrated *in vacuo* before being purified via flash column chromatography.

General Procedure C for the Synthesis of 1,3-dihydro-2,1-benzisoxazoles:

To a stirred 0.2M solution of *N*-alkyl 2,1-benzisoxazol-3(1*H*)-one (1 mmol, 1 eq) in anhydrous DCM under Ar at 0 °C was added TMSCl (2.5 mmol, 2.5 eq) followed by dropwise addition of LAH 2M in THF (2.5 mmol, 2.5 eq). The reaction mixture was stirred for 1 h (or until full consumption of starting material by TLC) before being quenched by dropwise addition of EtOAc 4 mL per mmol, followed by sat. Rochelle's salt solution 8 mL per mmol and the reaction mixture was stirred vigorously for 15-30 mins. The crude mixture was further diluted with water before being extracted with EtOAc 3 x 20 mL per mmol and the organic layers washed with brine, dried over Na_2SO_4, filtered, and concentrated *in vacuo* before being purified via flash column chromatography.

1-Benzylbenzo[c]isoxazol-3(*1H*)-one (6):

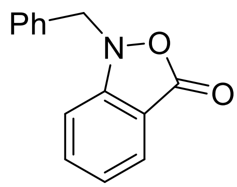

Following the general procedure B, to a stirred solution of benzo[c]isoxazol-3(*1H*)-one (108 mg, 0.80 mmol, 1 eq) in dry THF (0.05M, 16 mL) at 0 °C was added LHMDS (1M in THF) (0.96 mL, 0.96 mmol, 1.2 eq) dropwise followed by benzyl bromide (0.114 mL, 0.96 mmol, 1.2 eq). The reaction mixture was stirred for 12 h before being quenched by dropwise addition of 1M HCl (5 mL) and further diluted in EtOAc and additional 1M HCl (10 mL). Following workup, the crude reaction mixture was purified via flash column chromatography on alumina (eluent: 20:80 EtOAc:hexanes) to afford the title compound **6** (97 mg, 0.43 mmol, 39%) as a white crystalline solid.

TLC: R_f= 0.40 (30:70 EtOAc:Hexanes; UV, CAM)

IR: ν (C=O) 1750 cm^{-1}

m.p = 86.4 °C

^{1}H NMR (500 MHz, $CDCl_3$): δ 7.78 (d, *J* = 7.8 Hz, 1H), 7.62 (t, *J* = 7.8 Hz, 1H), 7.39 – 7.30 (m, 5H), 7.23 (t, *J* = 7.2 Hz, 1H), 7.07 (d, *J* = 8.4 Hz, 1H), 4.75 (s, 2H).

^{13}C NMR (126 MHz, $CDCl_3$): δ 168.0, 157.1, 135.0, 133.3, 129.3, 128.8, 128.7, 126.1, 124.4, 113.9, 112.3, 60.2.

HRMS-ESI (m/z): $[M+H]^+$ calculated for $[C_{14}H_{12}O_2N]^+$: 226.0862, found: 226.0856.

1-Allylbenzo[*c*]isoxazol-3(*1H*)-one (7):

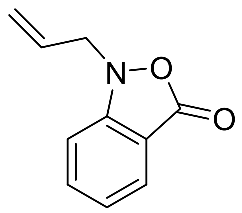

Following the general procedure B, to a stirred solution of benzo[c]isoxazol-3(*1H*)-one (5.6 g, 42 mmol, 1 eq) in dry THF (0.05M, 417 mL) at 0 °C was added LHMDS (1M in THF) (50 mL, 50 mmol, 1.2 eq) dropwise followed by allyl bromide (4.3 mL, 50 mmol, 1.2 eq). The reaction mixture was stirred for 12 h before being quenched by slow addition of 1M HCl (100 mL). Following workup, the crude reaction mixture was purified via flash column chromatography on silica (eluent: 30:70 EtOAc:hexanes) to afford the title compound **7** (4.1 g, 23 mmol, 38%) as a clear yellow oil.

TLC: R_f = 0.48 (30:70 EtOAc:Hexanes; UV, CAM)

IR: ν (C=O) 1753 cm^{-1}

^{1}H NMR (500 MHz, $CDCl_3$): δ 7.78 (d, *J* = 7.9 Hz, 1H, H4), 7.66 – 7.61 (m, 1H, H6), 7.23 (t, *J* = 7.6 Hz, 1H, H5), 7.15 (d, *J* = 8.3 Hz, 1H, H7), 5.81 (ddt, *J* = 16.8, 10.3, 6.4 Hz, 1H, CH), 5.35 (dd, *J* = 17.1, 1.4 Hz, 1H, CH_2=C), 5.26 (dd, *J* = 10.5, 1.4 Hz, 1H, CH_2=C), 4.21 (d, *J* = 6.4 Hz, 2H, CH_2).

^{13}C NMR (126 MHz, $CDCl_3$): δ 168.1, 157.0, 135.1, 129.6, 125.9, 124.3, 121.3, 113.6, 112.1, 58.4.

HRMS-ESI (m/z): $[M+H]^+$ calculated for $[C_{10}H_{10}O_2N]^+$: 176.0706, found: 176.0702.

1-Benzyl-5-methylbenzo[c]isoxazol-3(*1H*)-one (8):

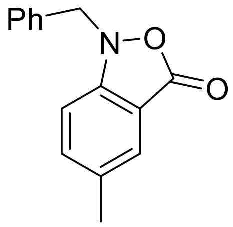

Following general procedure B, to a stirred solution of 5-methylbenzo[c]isoxazol-3(*1H*)-one (113 mg, 0.76 mmol, 1 eq) in dry THF (0.05M, 15 mL) at 0 °C was added LHMDS (1M in THF) (0.91 mL, 0.91 mmol, 1.2 eq) dropwise followed by benzyl bromide (0.11 mL, 0.91 mmol, 1.2 eq). The reaction mixture was stirred for 6 hours before being quenched by dropwise addition of 1M HCl (2 mL) and further diluted in EtOAc and additional 1M HCl (20 mL). Following workup, the crude reaction mixture was purified via flash column chromatography on alumina (eluent: 20:80 EtOAc:1%Et_3N in hexanes) to afford the title compound **8** (119 mg, 0.50 mmol, 49%) as a yellow crystalline solid.

TLC: R_f= 0.58 (30:70 EtOAc:Hexanes; UV, CAM)

m.p = 80.6 °C

IR: ν (C=O) 1747 cm^{-1}

^{1}H NMR (500 MHz, $CDCl_3$): δ 7.55 (s, 1H), 7.43 (d, *J* = 8.5 Hz, 1H), 7.38 – 7.30 (m, 5H), 6.96 (d, *J* = 8.4 Hz, 1H), 4.69 (s, 2H), 2.39 (s, 3H).

^{13}C NMR (126 MHz, $CDCl_3$): δ 168.0, 155.4, 136.4, 134.4, 133.3, 129.2, 128.6, 128.4, 125.1, 113.9, 112.1, 60.4, 20.8.

HRMS-ESI (m/z): $[M+H]^+$ calculated for $[C_{15}H_{14}O_2N]^+$: 240.1019, found: 240.1011.

1-Benzyl-5-bromobenzo[c]isoxazol-3(*1H*)-one (9):

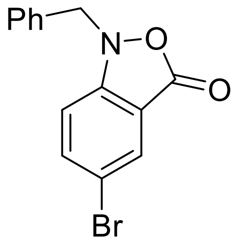

Following general procedure B, to a stirred solution of 5-bromobenzo[c]isoxazol-3(*1H*)-one (124 mg, 0.58 mmol, 1 eq) in dry THF (0.05M, 12 mL) at 0 °C was added LHMDS (1M in THF) (0.70 mL, 0.70 mmol, 1.2 eq) dropwise followed by benzyl bromide (0.08 mL, 0.70 mmol, 1.2 eq). The reaction mixture was stirred for 18 hours before being quenched by dropwise addition of 1M HCl (1 mL) and further diluted in EtOAc and additional 1M HCl (10 mL). Following workup, the crude reaction mixture was purified via flash column chromatography on alumina (eluent: 20:80 EtOAc:1% Et_3N in Hexanes) to afford the title compound **9** (107 mg, 0.35 mmol, 46%) as a white crystalline solid.

TLC: R_f = 0.55 (30:70 EtOAc:Hexanes; UV, CAM)

m.p = 87.4°C

IR: ν (C=O) 1753 cm^{-1}

^{1}H NMR (500 MHz, $CDCl_3$): δ 7.88 (s, 1H), 7.68 (dd, *J* = 8.8, 1.6 Hz, 1H), 7.33 (s, 5H), 6.94 (d, *J* = 8.7 Hz, 1H), 4.74 (s, 2H).

^{13}C NMR (126 MHz, $CDCl_3$): δ 166.3, 155.6, 138.0, 132.8, 129.3, 128.9, 128.6, 117.0, 115.7, 113.9, 60.0.

HRMS-ESI (m/z): $[M+Na]^+$ calculated for $[C_{14}H_{10}O_2NBrNa]^+$:325.9787, found: 325.9779.

1-Allyl-7-fluorobenzo[c]isoxazol-3(*1H*)-one (10):

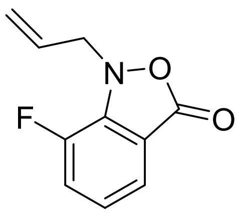

Following general procedure B, to a stirred solution of 7-fluorobenzo[c]isoxazol-3(*1H*)-one (102 mg, 0.67 mmol, 1 eq) in dry THF (0.05M, 13 mL) at 0 °C was added LHMDS (1M in THF) (0.80 mL, 0.80 mmol, 1.2 eq) dropwise followed by allyl bromide (0.07 mL, 0.80 mmol, 1.2 eq). The reaction mixture was stirred for 8 hours before being quenched by dropwise addition of 1M HCl (1 mL) and further diluted in EtOAc and additional 1M HCl (10 mL). Following workup, the crude reaction mixture was purified via flash column chromatography on alumina (eluent: 20:80 EtOAc:1% Et_3N in hexanes) to afford the title compound **10** (76 mg, 0.40 mmol, 39%) as a clear-yellow oil.

TLC: R_f= 0.42 (20:80 EtOAc:Hexanes; UV, CAM)

IR: ν (C=O) 1764 cm^{-1}

^{1}H NMR (500 MHz, $CDCl_3$): δ 7.63 (dd, *J* = 7.8, 1.0 Hz, 1H), 7.36 (ddd, *J* = 10.6, 8.0, 1.0 Hz, 1H), 7.23 (td, *J* = 7.9, 4.0 Hz, 1H), 5.83 – 5.73 (m, 1H), 5.35 (dd, *J* = 17.1, 1.3 Hz, 1H), 5.25 (d, *J* = 10.2 Hz, 1H), 4.35 (d, *J* = 6.6 Hz, 2H).

^{13}C NMR (126 MHz, $CDCl_3$): δ 167.2 (d, $^4J_{C\text{-}F}$ = 1.3 Hz), 148.3 (d, $^1J_{C\text{-}F}$ = 252 H), 144.4 4 (d, $^2J_{C\text{-}F}$ = 13.9 Hz), 129.1, 125.7 (d, $^3J_{C\text{-}F}$ = 5.0 Hz), 122.1, 121.8 (d, $^3J_{C\text{-}F}$ = 5.0 Hz), 121.0 (d, $^2J_{C\text{-}F}$ = 17.6 Hz), 117.6 (d, $^4J_{C\text{-}F}$ = 2.5 Hz), 57.7 (d, $^4J_{C\text{-}F}$ = 3.8 Hz).

^{13}C NMR {^{19}F} (126 MHz, $CDCl_3$): δ 167.2, 148.3, 144.4, 129.1, 125.7, 122.1, 121.8, 121.0, 117.6, 57.7.

HRMS-ESI (m/z): $[M+H]^+$ calculated for $[C_{10}H_9O_2NF]^+$:194.0612, found: 194.0609.

6-Bromo-1-(4-bromobenzyl)benzo[c]isoxazol-3(*1H*)-one (11):

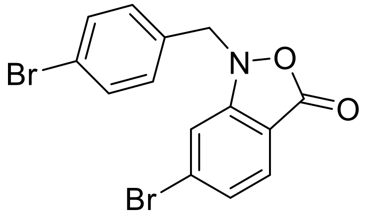

Following general procedure B, to a stirred solution of 6-bromobenzo[c]isoxazol-3(*1H*)-one (170 mg, 0.80 mmol, 1 eq) in dry THF (0.05M, 16 mL) at 0 °C was added LHMDS (1M in THF) (0.96 mL, 0.96 mmol, 1.2 eq) dropwise followed by 4-bromobenzyl bromide (0.239 mg, 0.96 mmol, 1.2 eq). The reaction mixture was stirred for 12 hours before being quenched by dropwise addition of 1M HCl (0.5 mL) and further diluted in EtOAc and additional 1M HCl (15 mL). Following workup, the crude reaction mixture was purified via flash column chromatography on silica (eluent: 10:90 EtOAc:hexanes) followed by trituration with methanol to afford the title compound **11** (168 mg, 0.44 mmol, 46%) as a white crystalline solid.

TLC: R_f= 0.41 (20:80 EtOAc:Hexanes; UV, CAM)

m.p = 188.3 °C

IR: ν (C=O) 1743 cm^{-1}

^{1}H NMR (500 MHz, $CDCl_3$): δ 7.64 (d, *J* = 8.3 Hz, 1H), 7.48 (d, *J* = 8.4 Hz, 2H), 7.37 (d, *J* = 8.3 Hz, 1H), 7.31 (s, 1H), 7.23 (d, *J* = 8.1 Hz, 2H), 4.69 (s, 2H)

^{13}C NMR (126 MHz, $CDCl_3$): δ 166.8, 157.4, 132.1, 131.8, 130.8, 130.4, 128.3, 127.4, 123.1, 115.2, 112.7, 59.3.

HRMS-ESI (m/z): $[M-H]^-$ calculated for $[C_{14}H_8O_2NBr_2]^-$:379.8916, found: 379.8927.

1-Allyl-5-fluorobenzo[c]isoxazol-3(*1H*)-one (12):

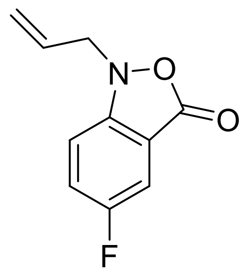

Following general procedure B, to a stirred solution of 5-fluorobenzo[c]isoxazol-3(*1H*)-one (702 mg, 4.6 mmol, 1 eq) in dry THF (0.05M, 91 mL) at 0 °C was added LHMDS (1M in THF) (5.5 mL, 5.5 mmol, 1.2 eq) dropwise followed by allyl bromide (0.48 mL, 5.5 mmol, 1.2 eq). The reaction mixture was stirred for 6 hours before being quenched by dropwise addition of 1M HCl (1 mL) and further diluted in EtOAc and additional 1M HCl (40 mL). Following workup, the crude reaction mixture was purified via flash column chromatography on silica (20:80 EtOAc:Hexanes) to afford the title compound **12** (429 mg, 2.2 mmol, 44%) as a clear-yellow oil.

TLC: R_f= 0.56 (30:70 EtOAc:Hexanes; UV, CAM)

IR: ν (C=O) 1753 cm^{-1}

^{1}H NMR (500 MHz, $CDCl_3$): δ 7.47 – 7.35 (m, 2H), 7.15 (dd, *J* = 8.9, 3.8 Hz, 1H), 5.87 – 5.77 (m, 1H), 5.36 (d, *J* = 17.2 Hz, 1H), 5.29 (d, *J* = 10.3 Hz, 1H), 4.19 (d, *J* = 6.7 Hz, 2H).

^{13}C NMR (126 MHz, $CDCl_3$): δ 167.2 (d, $^4J_{C-F}$ = 4.23 Hz), 159.7 (d, $^1J_{C-F}$ = 246 Hz), 153.7, 129.4 (d, $^2J_{C-F}$ = 26.0 Hz), 123.8, 121.7, 114.9 (d, $^3J_{C-F}$ = 9.39 Hz), 113.8 (d, $^3J_{C-F}$ = 8.18 Hz), 111.1 (d, $^2J_{C-F}$ = 24.5 Hz), 58.9.

^{13}C NMR {^{19}F} (126 MHz, $CDCl_3$): δ 167.2, 159.7, 153.7, 129.4, 123.8, 121.7, 114.9, 113.8, 111.1, 58.9.

HRMS-ESI (m/z): $[M+H]^+$ calculated for $[C_{10}H_9O_2NF]^+$:194.0612, found: 194.0610.

1-(4-Bromobenzyl)-6-fluorobenzo[c]isoxazol-3(*1H*)-one (13):

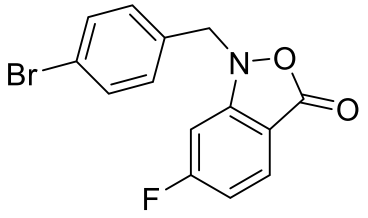

Following general procedure B, to a stirred solution of 6-fluorobenzo[c]isoxazol-3(*1H*)-one (128 mg, 0.84 mmol, 1 eq) in dry THF (0.05M, 16 mL) at 0 °C was added LHMDS (1M in THF) (1.00 mL, 1.0 mmol, 1.2 eq) dropwise followed by 4-bromobenzyl bromide (0.250 mg, 1.0 mmol, 1.2 eq). The reaction mixture was stirred for 12 hours before being quenched by dropwise addition of 1M HCl (1 mL) and further diluted in EtOAc and additional 1M HCl (10 mL). Following workup, the crude reaction mixture was purified via flash column chromatography on silica (eluent: 20:80 EtOAc:Hexanes) to afford the title compound **13** (96 mg, 0.30 mmol, 30%) as a white crystalline solid.

TLC: R_f= 0.55 (30:70 EtOAc:Hexanes; UV, CAM)

m.p = 161.4 °C

IR: ν (C=O) 1745 cm^{-1}

^{1}H NMR (500 MHz, $CDCl_3$): δ 7.76 (dd, *J* = 8.7, 5.1 Hz, 1H), 7.47 (d, *J* = 8.4 Hz, 2H), 7.23 (d, *J* = 8.4 Hz, 2H), 6.95 (t, *J* = 8.7 Hz, 1H), 6.76 (d, *J* = 8.5 Hz, 1H), 4.69 (s, 2H).

^{13}C NMR (126 MHz, $CDCl_3$): δ 167.3 (d, $^1J_{C\text{-}F}$ = 257 Hz), 166.6, 158.1 (d, $^3J_{C\text{-}F}$ = 13.0 Hz), 132.0, 131.8, 130.8, 128.5 (d, $^3J_{C\text{-}F}$ = 11.5 Hz), 123.0, 113.4 (d, $^2J_{C\text{-}F}$ = 24.8 Hz), 109.9, 99.2 (d, $^2J_{C\text{-}F}$ = 27.5 Hz), 59.1.

^{13}C NMR {^{19}F} (126 MHz, $CDCl_3$): δ 167.3, 166.6, 158.1, 132.0, 131.8, 130.8, 128.5, 123.0, 113.4, 109.9, 99.2, 59.1.

HRMS-ESI (m/z): $[M+H]^+$ calculated for $[C_{14}H_{10}O_2NBrF]^+$:321.9874, found: 321.9868.

1-Allyl-6-methoxybenzo[c]isoxazol-3(*1H*)-one (14):

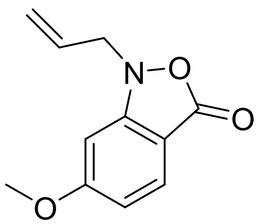

Following general procedure B, to a stirred solution of 6-methoxybenzo[*c*]isoxazol-3(*1H*)-one (1.96 g, 12 mmol, 1 eq) in dry THF (0.05M, 237 mL) at 0 °C was added LHMDS (1M in THF) (14.24 mL, 14 mmol, 1.2 eq) dropwise followed by allyl bromide (1.23 mL, 14 mmol, 1.2 eq). The reaction mixture was stirred for 12 hours before being quenched by dropwise addition of 1M HCl (10 mL) and further diluted in EtOAc and additional 1M HCl (100 mL). Following workup, the crude reaction mixture was purified via flash column chromatography on silica (eluent: 30:70 EtOAc:Hexanes) to afford the title compound **14** (1.15 g, 5.6 mmol, 25%) as an off-white crystalline solid.

TLC: R_f= 0.48 (40:60 EtOAc:Hexanes; UV, CAM)

m.p = 57.7 °C

IR: ν (C=O) 1738 cm^{-1}

^{1}H NMR (500 MHz, $CDCl_3$): δ 7.67 (d, *J* = 8.7 Hz, 1H), 6.79 (d, *J* = 6.6 Hz, 1H), 6.51 (s, 1H), 5.90 – 5.79 (m, 1H), 5.37 (d, *J* = 17.2 Hz, 1H), 5.28 (d, *J* = 9.1 Hz, 1H), 4.19 (d, *J* = 6.3 Hz, 2H), 3.88 (s, 3H).

^{13}C NMR (126 MHz, $CDCl_3$): δ 167.8, 165.9, 159.5, 129.9, 127.1, 121.2, 113.8, 106.4, 94.8, 58.3, 56.0.

HRMS-ESI (m/z): $[M+H]^+$ calculated for $[C_{11}H_{12}O_3N]^+$:206.0812, found: 206.0804.

1-Benzyl-6-bromobenzo[*c*]isoxazol-3(*1H*)-one (15):

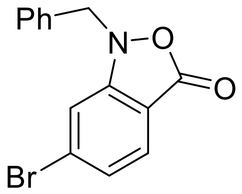

Following general procedure B, to a stirred solution of 6-bromobenzo[*c*]isoxazol-3(*1H*)-one (1.94 g, 6.4 mmol, 1 eq) in dry THF (0.05M, 42.7 mL) at 0 °C was added LHMDS (1M in THF) (13.4 mL, 13 mmol, 1.2 eq) dropwise followed by benzyl bromide (13.4 mL, 13 mmol, 1.2 eq). The reaction mixture was stirred for 12 hours before being quenched by dropwise addition of 1M HCl (10 mL) and further diluted in EtOAc and additional 1M HCl (100 mL). Following workup, the crude reaction mixture was purified via flash column chromatography on silica (eluent: 60:40 DCM:Hexanes) to afford the title compound **15** (1.96 g, 6.5 mmol, 55%) as a white crystalline solid.

TLC: R_f= 0.62 (30:70 EtOAc:Hexanes; UV, CAM)

m.p = 166.5 °C

IR: ν (C=O) 1750 cm^{-1}

^{1}H NMR (500 MHz, $CDCl_3$): δ 7.61 (d, *J* = 8.2 Hz, 1H), 7.33 (m, 6H), 7.25 (s, 1H), 4.73 (s, 2H).

^{13}C NMR (126 MHz, $CDCl_3$): δ 167.0, 157.5, 132.8, 130.2, 129.2, 128.9, 128.9, 127.9, 127.2, 115.3, 112.6, 59.9.

HRMS-ESI (m/z): $[M+H]^+$ calculated for $[C_{14}H_{11}O_2NBr]^+$:303.9968, found: 303.9960.

1-(4-Bromobenzyl)-6-(trifluoromethyl)benzo[c]isoxazol-3(*1H*)-one (16):

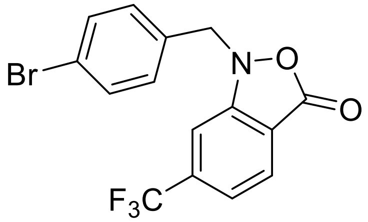

Following general procedure B, to a stirred solution of 6-(trifluoromethyl)benzo[*c*]isoxazol-3(*1H*)-one (156 mg, 0.77 mmol, 1 eq) in dry THF (0.05M, 15 mL) at 0 °C was added LHMDS (1M in THF) (0.922 mL, 0.92 mmol, 1.2 eq) dropwise followed by 4-bromobenzyl bromide (0.422 mg, 1.7 mmol, 2.2 eq). The reaction mixture was stirred for 12 hours before being quenched by dropwise addition of 1M HCl (1 mL) and further diluted in EtOAc and additional 1M HCl (15 mL). Following workup, the crude reaction mixture was purified via flash column chromatography on silica (eluent: 40:60 DCM:Hexanes) to afford the title compound **16** (139 mg, 0.38 mmol, 37%) as a white crystalline solid.

TLC: R_f= 0.49 (20:80 EtOAc:Hexanes; UV, CAM)

m.p = 142.5 °C

IR: ν (C=O) 1750 cm^{-1}

^{1}H NMR (500 MHz, $CDCl_3$): δ 7.92 (d, *J* = 8.2 Hz, 1H), 7.49 (t, *J* = 8.1 Hz, 3H), 7.40 (s, 1H), 7.23 (d, *J* = 8.4 Hz, 2H), 4.76 (s, 2H).

^{13}C NMR (126 MHz, $CDCl_3$): δ 166.4, 156.1, 137.1 (d, $^2J_{C\text{-}F}$ = 33.3 Hz), 132.2, 131.6, 130.8, 127.4, 123.2 (d, $^1J_{C\text{-}F}$ = 274 Hz), 123.2, 121.3 (q, $^4J_{C\text{-}F}$ = 3.39 Hz), 116.5, 109.6 (d, $^4J_{C\text{-}F}$ = 4.23 Hz), 59.4.

^{13}C NMR {^{19}F} (126 MHz, $CDCl_3$): δ 166.4, 156.1, 137.1, 132.2, 131.6, 130.8, 127.4, 123.2, 123.2, 123.2, 121.3, 116.5, 109.6, 59.4.

HRMS-ESI (m/z): $[M-H]^-$ calculated for $[C_{15}H_8O_2NBrF_3]^+$:369.9685, found: 369.9697.

5-Fluoro-1-hexylbenzo[*c*]isoxazol-3(*1H*)-one (17):

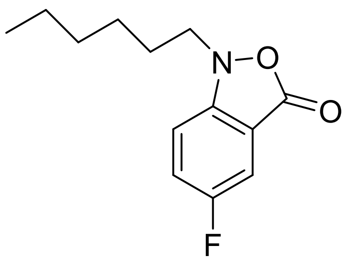

Following general procedure B, to a stirred solution of 5-fluorobenzo[*c*]isoxazol-3(*1H*)-one (359 mg, 2.3 mmol, 1 eq) in dry THF (0.05M, 47 mL) at room temperature was added LHMDS (1M in THF) (2.81 mL, 2.8 mmol, 1.2 eq) dropwise followed by 1-iodohexane (1.38 mL, 9.4 mmol, 4.0 eq). The reaction mixture was heated to 40 °C and stirred for 4.5 hours before being cooled to room temperature, quenched by dropwise addition of 1M HCl (10 mL) and further diluted in EtOAc and additional 1M HCl (30 mL). Following workup, the crude reaction mixture was purified via flash column chromatography on silica (eluent: 20:80 DCM:Hexanes) to afford the title compound **17** (273 mg, 1.2 mmol, 45%) as a bright yellow oil.

TLC: R_f= 0.35 (10:90 EtOAc:Hexanes; UV, CAM)

IR: ν (C=O) 1750 cm^{-1}

^{1}H NMR (500 MHz, $CDCl_3$): δ 7.45 (dd, *J* = 7.0, 2.9 Hz, 1H), 7.39 (td, *J* = 8.7, 2.6 Hz, 1H), 7.09 (dd, *J* = 8.9, 3.7 Hz, 1H), 3.52 (t, *J* = 7.3 Hz, 2H), 1.73 (p, *J* = 7.9 Hz, 2H), 1.41 (p, *J* = 7.0 Hz, 2H), 1.36 – 1.27 (m, 4H), 0.89 (t, *J* = 6.9 Hz, 3H).

^{13}C NMR (126 MHz, $CDCl_3$): δ 167.4 (d, $^{4}J_{C\text{-}F}$ = 4.23 Hz), 159.4 (d, $^{1}J_{C\text{-}F}$ = 245 Hz), 154.1, 123.9 (d, $^{2}J_{C\text{-}F}$ = 25.7 Hz), 114.2 (d, $^{3}J_{C\text{-}F}$ = 9.39 Hz), 113.2 (d, $^{3}J_{C\text{-}F}$ = 8.47 Hz), 111.2 (d, $^{2}J_{C\text{-}F}$ = 24.5 Hz), 57.0, 31.5, 26.6, 26.2, 22.6, 14.1.

^{13}C NMR {^{19}F} (126 MHz, $CDCl_3$): δ 167.4, 159.4, 154.1, 123.9, 114.2, 113.2, 111.2, 57.0, 31.5, 26.6, 26.2, 22.6, 14.1.

HRMS-ESI (m/z): $[M+H]^+$ calculated for $[C_{13}H_{17}O_2NF]^+$:238.1238, found: 238.1241.

1-(Benzyloxy)indolin-2-one (18):

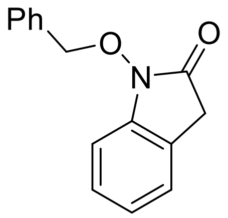

Following general procedure B, to a stirred solution of 1-hydroxyindolin-2-one (477 mg, 3.2 mmol, 1 eq) in dry THF (0.05M, 64 mL) at 0 °C was added DBU (0.573 mL, 3.8 mmol, 1.2 eq) dropwise followed by benzyl bromide (0.456 mL, 3.8 mmol, 1.2 eq). The reaction mixture was stirred for 12 hours before being quenched by dropwise addition of 1M HCl (10 mL) and further diluted in EtOAc and additional 1M HCl (100 mL). Following workup, the crude reaction mixture was purified via flash column chromatography on silica (30:70 EtOAc:Hexanes) to afford the title compound **18** (588 mg, 2.5 mmol, 48%) as a tan crystalline solid.

TLC: R_f= 0.57 (40:60 EtOAc:Hexanes; UV, CAM)

IR: ν (C=O) 1683 cm^{-1}

^{1}H NMR (500 MHz, $CDCl_3$): δ 7.54 – 7.48 (m, 2H, Ph), 7.38 (d, *J* = 5.0 Hz, 3H, Ph), 7.24 – 7.17 (m, 2H, H4, H6), 7.01 (t, *J* = 7.5 Hz, 1H, H5), 6.81 (d, *J* = 7.7 Hz, 1H, H7), 5.19 (s, 2H, CH_2Ph), 3.51 (s, 2H, H3).

^{13}C NMR (126 MHz, $CDCl_3$): δ 170.1, 142.2, 134.4, 129.9, 129.4, 128.8, 128.1, 124.8, 122.9, 120.5, 107.7, 78.2, 34.2.

HRMS-ESI (m/z): $[M+H]^+$ calculated for $[C_{15}H_{14}O_2N]^+$:240.1019, found: 240.1016.

1-(Benzyloxy)-3,4-dihydroquinolin-2(*1H*)-one (19):

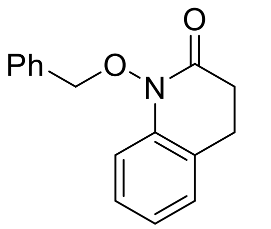

Following general procedure B, to a stirred solution of 1-hydroxy-3,4-dihydroquinolin-2(*1H*)-one (374 mg, 2.3 mmol, 1 eq) in dry THF (0.05M, 46 mL) at 0 °C was added DBU (0.41 mL, 2.8 mmol, 1.2 eq) dropwise followed by benzyl bromide (0.327 mL, 2.8 mmol, 1.2 eq). The reaction mixture was stirred for 12 hours before being quenched by dropwise addition of 1M HCl (10 mL) and further diluted in EtOAc and additional 1M HCl (100 mL). Following workup, the crude reaction mixture was purified via flash column chromatography on silica (30:70 EtOAc:Hexanes) to afford the title compound **19** (518 mg, 2.0 mmol, 77%) as a white crystalline solid.

TLC: R_f = 0.50 (40:60 EtOAc:Hexanes; UV, CAM)

IR: ν (C=O) 1687 cm^{-1}

^{1}H NMR (500 MHz, $CDCl_3$): δ 7.55 (d, *J* = 8.1 Hz, 2H, Ph), 7.44 – 7.36 (m, 3H, Ph), 7.28 (m, 2H, H5, H7), 7.18 (d, *J* = 7.3 Hz, 1H, H8), 7.07 – 7.02 (m, 1H, H6), 5.11 (s, 2H, CH_2Ph), 2.93 (t, *J* = 7.3 Hz, 2H, H3), 2.74 (t, *J* = 7.2 Hz, 2H, H4).

^{13}C NMR (126 MHz, $CDCl_3$): δ 166.1, 138.6, 134.4, 129.8, 129.1, 128.6, 127.8, 127.7, 124.4, 123.6, 112.8, 77.1, 31.8, 25.1.

HRMS-ESI (m/z): $[M+H]^+$ calculated for $[C_{16}H_{16}O_2N]^+$:254.1175, found: 254.1171.

1-Benzyl-1,3-dihydrobenzo[c]isoxazole (24):

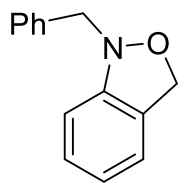

Following general procedure C, to a stirred solution of 1-benzylbenzo[c]isoxazol-3(*1H*)-one (50 mg, 0.22 mmol, 1 eq) in dry DCM (0.2M, 1.11 mL) at 0 °C was added TMSCl (0.070 mL, 0.55 mmol, 2.5 eq) followed by LAH (2M in THF) (0.28 mL, 0.55 mmol, 2.5 eq) dropwise. The reaction mixture was stirred for 1.5 h before being quenched by dropwise addition of EtOAc (2 mL) followed by sat. Rochelle's salt solution (4 mL) and vigorous stirring for 15-30 mins. Following workup, the crude reaction mixture was purified via flash column chromatography on silica (eluent: 20:80 EtOAc:hexanes) to afford the title compound **24** (27 mg, 0.13 mmol, 58%) as a clear-yellow syrup.

TLC: R_f = 0.56 (30:70 EtOAc:Hexanes; UV, CAM)

^{1}H NMR (500 MHz, $CDCl_3$): δ 7.43 (d, *J* = 6.7 Hz, 2H), 7.35 (t, *J* = 7.2 Hz, 2H), 7.31 (t, *J* = 7.2 Hz, 1H), 7.21 (t, *J* = 7.6 Hz, 1H), 7.14 (d, *J* = 7.4 Hz, 1H), 7.03 (t, *J* = 7.4 Hz, 1H), 6.76 (d, *J* = 7.9 Hz, 1H), 5.14 (s, 2H), 4.42 (s, 2H).

^{13}C NMR (126 MHz, $CDCl_3$): δ 149.5, 136.6, 130.1, 129.1, 128.5, 128.0, 127.7, 123.5, 121.6, 111.4, 71.0, 63.0.

HRMS-ESI (m/z): $[M+H]^+$ calculated for $[C_{14}H_{14}ON]^+$: 212.1069, found: 212.1065.

1-Allyl-1,3-dihydrobenzo[*c*]isoxazole (25):

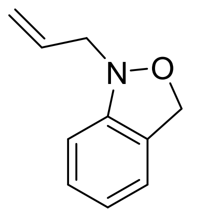

Following general procedure C, to a stirred solution of 1-allylbenzo[c]isoxazol-3(1H)-one (45 mg, 0.26 mmol, 1 eq) in dry DCM (0.2M, 1.3 mL) at 0 °C was added TMSCl (0.082 mL, 0.64 mmol, 2.5 eq) followed by LAH (2M in THF) (0.32 mL, 0.64 mmol, 2.5 eq) dropwise. The reaction mixture was stirred for 1.5 h before being quenched by dropwise addition of EtOAc (1 mL) followed by sat. Rochelle's salt solution (3 mL) and vigorous stirring for 15-30 mins. Following workup, the crude reaction mixture was purified via flash column chromatography on silica (eluent: 10:90 EtOAc:hexanes) to afford the title compound **25** (13 mg, 0.078 mmol, 30%) as a clear-yellow syrup.

TLC: R_f = 0.44 (10:90 EtOAc:Hexanes; UV, CAM)

^{1}H NMR (500 MHz, CDCl$_3$): δ 7.21 (t, *J* = 7.7 Hz, 1H), 7.15 (d, *J* = 7.4 Hz, 1H), 7.02 (t, *J* = 7.4 Hz, 1H), 6.79 (d, *J* = 7.9 Hz, 1H), 6.08 – 5.97 (m, 1H), 5.33 (d, *J* = 17.3 Hz, 1H), 5.24 (d, *J* = 10.3 Hz, 1H), 5.20 (s, 2H), 3.91 (dd, *J* = 6.2, 1.4 Hz, 2H).

^{13}C NMR (126 MHz, CDCl$_3$): δ 149.3, 133.2, 129.9, 128.0, 123.4, 121.6, 118.9, 111.1, 70.9, 61.2.

HRMS-ESI (m/z): $[M+H]^+$ calculated for $[C_{10}H_{12}ON]^+$: 162.0913, found: 162.0913.

1-Benzyl-5-methyl-1,3-dihydrobenzo[c]isoxazole (26):

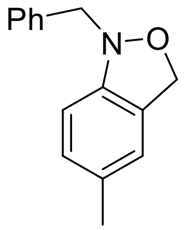

Following general procedure C, to a stirred solution of 1-benzyl-5-methylbenzo[c]isoxazol-3(*1H*)-one (82 mg, 0.34 mmol, 1 eq) in dry DCM (0.2M, 1.72 mL) at 0 °C was added TMSCl (0.11 mL, 0.86 mmol, 2.5 eq) followed by LAH (2M in THF) (0.43 mL, 0.86 mmol, 2.5 eq) dropwise. The reaction mixture was stirred for 1hr before being quenched by dropwise addition of EtOAc (0.5 mL) followed by sat. Rochelle's salt solution (3 mL) and vigorous stirring for 15-30 mins. Following workup, the crude reaction mixture was purified via flash column chromatography on silica (eluent: 10:80 EtOAc:hexanes) to afford the title compound **26** (42 mg, 0.19 mmol, 54%) as a clear-yellow oil.

TLC: R_f= 0.53 (20:80 EtOAc:Hexanes; UV, CAM)

^{1}H NMR (500 MHz, $CDCl_3$): δ 7.43 (d, *J* = 6.9 Hz, 2H), 7.36 (t, *J* = 7.2 Hz, 2H), 7.31 (t, *J* = 7.2 Hz, 1H), 7.01 (d, *J* = 8.0 Hz, 1H), 6.96 (s, 1H), 6.65 (d, *J* = 8.0 Hz, 1H), 5.11 (s, 2H), 4.38 (s, 2H), 2.33 (s, 3H).

^{13}C NMR (126 MHz, $CDCl_3$): δ 147.2, 136.6, 133.2, 130.4, 129.1, 128.5, 128.4, 127.6, 122.1, 111.3, 70.8, 63.3, 20.9.

HRMS-ESI (m/z): $[M+H]^+$ calculated for $[C_{15}H_{16}ON]^+$: 226.1219, found: 226.1226.

1-Benzyl-5-bromo-1,3-dihydrobenzo[c]isoxazole (27):

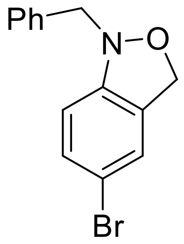

Following general procedure C, to a stirred solution of 1-benzyl-5-bromobenzo[c]isoxazol-3(*1H*)-one (80 mg, 0.26 mmol, 1 eq) in dry DCM (0.2M, 1.32 mL) at 0 °C was added TMSCl (0.08 mL, 0.66 mmol, 2.5 eq) followed by LAH (2M in THF) (0.33 mL, 0.66 mmol, 2.5 eq) dropwise. The reaction mixture was stirred for 1hr before being quenched by dropwise addition of EtOAc (0.5 mL) followed by sat. Rochelle's salt solution (3 mL) and vigorous stirring for 15-30 mins. Following workup, the crude reaction mixture was purified via flash column chromatography on silica (Eluent: 20:80 Acetone:Hexanes) to afford the title compound **27** (62 mg, 0.21 mmol, 81%) as a clear-yellow oil.

TLC: R_f = 0.56 (20:80 EtOAc:Hexanes; UV, CAM)

^{1}H NMR (500 MHz, $CDCl_3$): δ 7.40 (d, J = 6.8 Hz, 2H), 7.38 – 7.28 (m, 4H), 7.24 (s, 1H), 6.60 (d, J = 8.3 Hz, 1H), 5.09 (s, 2H), 4.40 (s, 2H).

^{13}C NMR (126 MHz, $CDCl_3$): δ 148.7, 136.0, 132.4, 130.9, 129.2, 128.6, 127.9, 124.8, 115.8, 112.7, 70.4, 62.7.

HRMS-ESI (m/z): $[M+H]^+$ calculated for $[C_{14}H_{13}ONBr]^+$:290.0175, found: 290.0168.

1-Allyl-7-fluoro-1,3-dihydrobenzo[c]isoxazole (28):

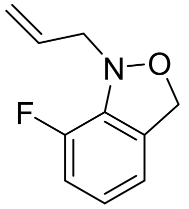

Following general procedure C, to a stirred solution of 1-allyl-7-fluorobenzo[c]isoxazol-3(*1H*)-one (37 mg, 0.19 mmol, 1 eq) in dry DCM (0.2M, 0.96 mL) at 0 °C was added TMSCl (0.061 mL, 0.48 mmol, 2.5 eq) followed by LAH (2M in THF) (0.48 mL, 0.48 mmol, 2.5 eq) dropwise. The reaction mixture was stirred for 1hr before being quenched by dropwise addition of EtOAc (0.5 mL) followed by sat. Rochelle's salt solution (3 mL) and vigorous stirring for 15-30 mins. Following workup, the crude reaction mixture was purified via flash column chromatography on silica (eluent: 20:80 EtOAc:hexanes) to afford the title compound **28** (22 mg, 0.12 mmol, 64%) as a colorless oil.

TLC: R_f= 0.58 (20:80 EtOAc:Hexanes; UV, CAM)

^{1}H NMR (500 MHz, $CDCl_3$): δ 6.99 (td, J = 7.8, 4.5 Hz, 1H), 6.93 (t, J = 8.6 Hz, 2H), 6.00 (m, 1H), 5.30 (d, J = 17.1 Hz, 1H), 5.24 (s, 2H), 5.21 (d, J = 10.2 Hz, 1H), 4.01 (d, J = 6.1 Hz, 2H).

^{13}C NMR (126 MHz, $CDCl_3$): δ 148.9 (d, $^1J_{C\text{-}F}$ = 245 Hz), 135.7 (d, $^2J_{C\text{-}F}$ = 11.3 Hz), 133.6 (d, $^3J_{C\text{-}F}$ = 3.78 Hz), 132.7, 125.2 (d, $^3J_{C\text{-}F}$ = 6.30 Hz), 119.4, 117.2, 115.4 (d, $^2J_{C\text{-}F}$ = 18.9 Hz), 71.8 (d, $^4J_{C\text{-}F}$ = 2.52 Hz), 59.8 (d, $^4J_{C\text{-}F}$ = 3.78 Hz).

^{13}C NMR {^{19}F} (126 MHz, $CDCl_3$): δ 148.9, 135.7, 133.6, 132.7, 125.2, 119.4, 117.2, 115.4, 71.8, 59.8.

HRMS-ESI (m/z): $[M+H]^+$ calculated for $[C_{10}H_{11}ONF]^+$:180.0819, found: 180.0814.

6-Bromo-1-(4-bromobenzyl)-1,3-dihydrobenzo[c]isoxazole (29):

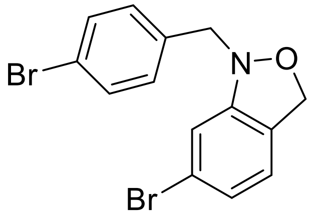

Following general procedure C, to a stirred solution of 6-bromo-1-(4-bromobenzyl)benzo[c]isoxazol-3(*1H*)-one (0.100 mg, 0.26 mmol, 1 eq) in dry DCM (0.2M, 1.31 mL) at 0 °C was added TMSCl (0.083 mL, 0.66 mmol, 2.5 eq) followed by LAH (2M in THF) (0.33 mL, 0.66 mmol, 2.5 eq) dropwise. The reaction mixture was stirred for 1hr before being quenched by dropwise addition of EtOAc (0.5 mL) followed by sat. Rochelle's salt solution (3 mL) and vigorous stirring for 15-30 mins. Following workup, the crude reaction mixture was purified via flash column chromatography on alumina (eluent: 10:90 EtOAc:hexanes) to afford the title compound **29** (71 mg, 0.19 mmol, 74%) as a colorless oil.

TLC: R_f= 0.60 (20:80 EtOAc:Hexanes; UV, CAM)

^{1}H NMR (500 MHz, $CDCl_3$): δ 7.46 (d, *J* = 8.4 Hz, 2H), 7.26 (d, *J* = 8.4 Hz, 2H), 7.13 (d, *J* = 7.9 Hz, 1H), 6.97 (d, *J* = 7.9 Hz, 1H), 6.90 (s, 1H), 5.04 (s, 2H), 4.33 (s, 2H).

^{13}C NMR (126 MHz, $CDCl_3$): δ 150.8, 135.0, 131.7, 130.7, 129.0, 126.5, 122.9, 121.9, 121.5, 114.2, 70.7, 61.6.

HRMS-ESI (m/z): $[M+H]^+$ calculated for $[C_{14}H_{12}ONBr_2]^+$:367.9280, found: 367.9270.

1-Allyl-5-fluoro-1,3-dihydrobenzo[c]isoxazole (30):

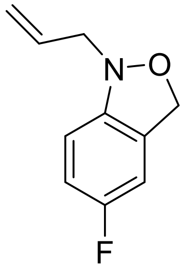

Following general procedure C, to a stirred solution of 1-allyl-5-fluorobenzo[c]isoxazol-3(*1H*)-one (60 mg, 0.31 mmol, 1 eq) in dry DCM (0.2M, 1.54 mL) at 0 °C was added TMSCl (0.098 mL, 0.77 mmol, 2.5 eq) followed by LAH (2M in THF) (0.39 mL, 0.77 mmol, 2.5 eq) dropwise. The reaction mixture was stirred for 1hr before being quenched by dropwise addition of EtOAc (2 mL) followed by sat. Rochelle's salt solution (3 mL) and vigorous stirring for 15-30 mins. Following workup, the crude reaction mixture was purified via flash column chromatography on alumina (eluent: 10:90 EtOAc:Hexanes) to afford the title compound **30** (28 mg, 0.15 mmol, 50%) as a clear-yellow oil.

TLC: R_f= 0.69 (30:70 EtOAc:Hexanes; UV, CAM)

^{1}H NMR (500 MHz, CDCl$_3$): δ 6.94 – 6.83 (m, 2H), 6.71 (dd, *J* = 8.5, 4.2 Hz, 1H), 6.00 (m, 1H), 5.31 (d, *J* = 17.3 Hz, 1H), 5.24 (d, *J* = 10.3 Hz, 1H), 5.16 (s, 2H), 3.85 (d, *J* = 6.3 Hz, 2H).

^{13}C NMR (126 MHz, CDCl$_3$): δ 159.7 (d, $^1J_{C\text{-}F}$ = 242 Hz), 145.4, 132.91, 131.7 (d, $^3J_{C\text{-}F}$ = 8.77 Hz), 119.1, 114.6 (d, $^2J_{C\text{-}F}$ = 24.2 Hz), 112.0 (d, $^3J_{C\text{-}F}$ = 8.77 Hz), 109.0 (d, $^2J_{C\text{-}F}$ = 25.1 Hz), 70.6 (d, $^4J_{C\text{-}F}$ = 2.42 Hz), 61.6.

^{13}C NMR {^{19}F} (126 MHz, CDCl$_3$): δ 159.7, 145.4, 132.9, 131.7, 119.1, 114.6, 112.0, 109.0, 70.6, 61.6.

HRMS-ESI (m/z): $[M+H]^+$ calculated for $[C_{10}H_{11}ONF]^+$:180.0819, found: 180.0811.

1-(4-Bromobenzyl)-6-fluoro-1,3-dihydrobenzo[c]isoxazole (31):

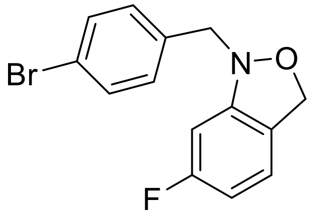

Following general procedure C, to a stirred solution of 1-(4-bromobenzyl)-6-fluorobenzo[c]isoxazol-3(*1H*)-one (0.074 mg, 0.23 mmol, 1 eq) in dry DCM (0.2M, 1.15 mL) at 0 °C was added TMSCl (0.073 mL, 0.58 mmol, 2.5 eq) followed by LAH (2M in THF) (0.29 mL, 0.58 mmol, 2.5 eq) dropwise. The reaction mixture was stirred for 1hr before being quenched by dropwise addition of EtOAc (3 mL) followed by sat. Rochelle's salt solution (3 mL) and vigorous stirring for 15-30 mins. Following workup, the crude reaction mixture was purified via flash column chromatography on silica (eluent: 30:70 EtOAc:Hexanes) to afford the title compound **31** (39 mg, 0.13 mmol, 55%) as a white crystalline solid.

TLC: R_f = 0.71 (30:70 EtOAc:Hexanes; UV, CAM)

m.p = 87.9 °C

^{1}H NMR (500 MHz, CDCl$_3$): δ 7.47 (d, *J* = 8.4 Hz, 2H), 7.28 (d, *J* = 8.3 Hz, 2H), 7.08 – 7.01 (m, 1H), 6.71 (t, *J* = 8.6 Hz, 1H), 6.46 (d, *J* = 9.0 Hz, 1H), 5.07 (s, 2H), 4.34 (s, 2H).

^{13}C NMR (126 MHz, CDCl$_3$): δ 163.0 (d, $^1J_{C\text{-}F}$ = 245 Hz), 150.8 (d, $^3J_{C\text{-}F}$ = 10.6 Hz), 135.0, 131.6, 130.7, 125.2, 122.5 (d, $^3J_{C\text{-}F}$ = 10.3 Hz), 121.9, 110.2 (d, $^2J_{C\text{-}F}$ = 23.3 Hz), 99.2 (d, $^2J_{C\text{-}F}$ = 27.5 Hz), 70.7, 61.6.

^{13}C NMR {^{19}F} (126 MHz, CDCl$_3$): δ 163.0, 150.8, 135.0, 131.6, 130.7, 125.2, 122.5, 121.9, 110.2, 99.2, 70.7, 61.6.

HRMS-ESI (m/z): $[M+H]^+$ calculated for $[C_{14}H_{12}ONBrF]^+$:308.0081, found: 308.0076.

1-Allyl-6-methoxy-1,3-dihydrobenzo[c]isoxazole (32):

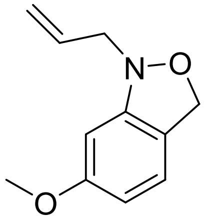

Following general procedure C, to a stirred solution of 1-allyl-6-methoxybenzo[c]isoxazol-3(*1H*)-one (0.606 mg, 3.0 mmol, 1 eq) in dry DCM (0.2M, 14.8 mL) at 0 °C was added TMSCl (0.94 mL, 7.4 mmol, 2.5 eq) followed by LAH (2M in THF) (3.69 mL, 7.4 mmol, 2.5 eq) dropwise. The reaction mixture was stirred for 1hr before being quenched by dropwise addition of EtOAc (5 mL) followed by sat. Rochelle's salt solution (5 mL) and vigorous stirring for 15-30 mins. Following workup, the crude reaction mixture was purified via flash column chromatography on silica (eluent: 10:90 EtOAc:Hexanes) to afford the title compound **32** (183 mg, 0.96 mmol, 32%) as a clear-yellow oil.

TLC: R_f= 0.64 (30:70 EtOAc:Hexanes; UV, CAM)

^{1}H NMR (500 MHz, $CDCl_3$): δ 7.02 (d, *J* = 8.2 Hz, 1H), 6.55 (d, *J* = 10.5 Hz, 1H), 6.35 (s, 1H), 6.08 – 5.95 (m, 1H), 5.33 (d, *J* = 17.3 Hz, 1H), 5.24 (d, *J* = 10.3 Hz, 1H), 5.13 (s, 2H), 3.89 (d, *J* = 6.2 Hz, 2H), 3.79 (s, 3H).

^{13}C NMR (126 MHz, $CDCl_3$): δ 160.2, 150.8, 133.2, 122.0, 121.9, 118.9, 108.7, 97.7,70.7, 61.0, 55.7.

HRMS-ESI (m/z): $[M+H]^+$ calculated for $[C_{11}H_{14}O_2N]^+$:192.1014, found: 192.1012.

1-Benzyl-6-bromo-1,3-dihydrobenzo[*c*]isoxazole (33):

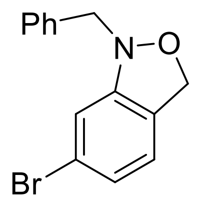

Following general procedure C, to a stirred solution of 1-benzyl-6-bromo-1,3-dihydrobenzo[*c*]isoxazole (1.94 g, 6.4 mmol, 1 eq) in dry DCM (0.2M, 42.7 mL) at 0 °C was added TMSCl (2.03 mL, 16 mmol, 2.5 eq) followed by LAH (2M in THF) (7.99 mL, 16 mmol, 2.5 eq) dropwise. The reaction mixture was stirred for 1hr before being quenched by dropwise addition of EtOAc (10 mL) followed by sat. Rochelle's salt solution (20 mL) and vigorous stirring for 15-30 mins. Following workup, the crude reaction mixture was purified via flash column chromatography on silica (eluent: 10:90 EtOAc:Hexanes) to afford the title compound **33** (935 mg, 3.2 mmol, 51%) as a white crystalline solid.

TLC: R_f = 0.68 (20:80 EtOAc:Hexanes; UV, CAM)

m.p. = 82.6 °C

^{1}H NMR (500 MHz, $CDCl_3$): δ 7.40 (d, *J* = 7.0 Hz, 2H), 7.33 (m, 3H), 7.13 (d, *J* = 7.9 Hz, 1H), 6.98 (d, *J* = 7.9 Hz, 1H), 6.89 (s, 1H), 5.06 (s, 2H), 4.40 (s, 2H).

^{13}C NMR (126 MHz, $CDCl_3$): δ 151.2, 136.0, 129.2, 129.1, 128.6, 128.0, 126.3, 122.9, 121.5, 114.4, 70.7, 62.5.

HRMS-ESI (m/z): $[M+H]^+$ calculated for $[C_{14}H_{13}ONBr]^+$: 290.0175, found: 290.0170.

1-(4-Bromobenzyl)-6-(trifluoromethyl)-1,3-dihydrobenzo[*c*]isoxazole (34):

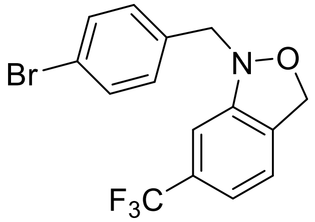

Following general procedure C, to a stirred solution of 1-(4-bromobenzyl)-6-(trifluoromethyl)benzo[*c*]isoxazol-3(*1H*)-one (0.116 mg, 0.31 mmol, 1 eq) in dry DCM (0.2M, 1.56 mL) at 0 °C was added TMSCl (0.1 mL, 0.78 mmol, 2.5 eq) followed by LAH (2M in THF) (0.39 mL, 0.78 mmol, 2.5 eq) dropwise. The reaction mixture was stirred for 1hr before being quenched by dropwise addition of EtOAc (3 mL) followed by sat. Rochelle's salt solution (3 mL) and vigorous stirring for 15-30 mins. Following workup, the crude reaction mixture was purified via flash column chromatography on silica (eluent: 30:70 DCM:Hexanes) to afford the title compound **34** (60 mg, 0.17 mmol, 54%) as a white crystalline solid.

TLC: R_f= 0.57 (20:80 EtOAc:Hexanes; UV, CAM)

m.p = 71.9 °C

^{1}H NMR (500 MHz, $CDCl_3$): δ 7.48 (d, *J* = 8.4 Hz, 2H), 7.32 – 7.27 (m, 3H), 7.23 (d, *J* = 7.8 Hz, 1H), 6.99 (s, 1H), 5.15 (s, 2H), 4.40 (s, 2H).

^{13}C NMR (126 MHz, $CDCl_3$): δ 149.8, 134.9, 133.8, 131.8, 131.0 (d, $^2J_{C-F}$ = 32.7 Hz), 130.7, 124.1 (d, $^1J_{C-F}$ = 273 Hz), 122.2, 122.1, 120.8 (q, $^4J_{C-F}$ = 3.93 Hz), 107.9 (q, $^4J_{C-F}$ = 3.93 Hz), 70.7, 61.7.

^{13}C NMR {^{19}F} (126 MHz, $CDCl_3$): δ 149.8, 134.9, 133.8, 131.8, 131.0, 130.7, 124.1, 122.2, 122.1, 120.8, 107.9, 70.7, 61.7.

HRMS-ESI (m/z): $[M+H]^+$ calculated for $[C_{15}H_{12}ONBrF_3]^+$:358.0049, found: 358.0046.

5-Fluoro-1-hexyl-1,3-dihydrobenzo[c]isoxazole (35):

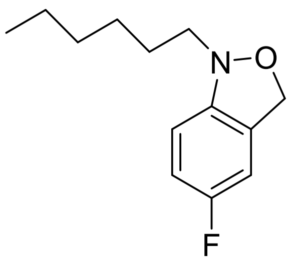

Following general procedure C, to a stirred solution of 5-fluoro-1-hexylbenzo[*c*]isoxazol-3(*1H*)-one (0.150 mg, 0.63 mmol, 1 eq) in dry DCM (0.2M, 3.16 mL) at 0 °C was added TMSCl (0.20 mL, 1.6 mmol, 2.5 eq) followed by LAH (2M in THF) (0.79 mL, 1.6 mmol, 2.5 eq) dropwise. The reaction mixture was stirred for 1hr before being quenched by dropwise addition of EtOAc (3 mL) followed by sat. Rochelle's salt solution (4 mL) and vigorous stirring for 15-30 mins. Following workup, the crude reaction mixture was purified via flash column chromatography on silica (eluent: 40:60 DCM:Hexanes) to afford the title compound **35** (100 mg, 0.45 mmol, 71%) as a yellow oil.

TLC: R_f = 0.48 (10:90 EtOAc:Hexanes; UV, CAM)

^{1}H NMR (500 MHz, CDCl$_3$): δ 6.93 – 6.82 (m, 2H), 6.66 (dd, J = 8.5, 4.2 Hz, 1H), 5.15 (s, 2H), 3.17 (t, J = 7.4 Hz, 2H), 1.70 (p, J = 7.4 Hz, 2H), 1.39 (m, 2H), 1.36 – 1.29 (m, 4H), 0.90 (t, J = 6.7 Hz, 3H).

^{13}C NMR (126 MHz, CDCl$_3$): δ 159.6 (d, $^1J_{C\text{-}F}$ = 241 Hz), 146.2, 131.5 (d, $^3J_{C\text{-}F}$ = 8.48 Hz), 114.6 (d, $^2J_{C\text{-}F}$ = 23.9 Hz), 111.6 (d, $^3J_{C\text{-}F}$ = 8.77 Hz), 109.0 (d, $^2J_{C\text{-}F}$ = 25.1 Hz), 70.4, 59.5, 31.8, 27.1, 27.0, 22.7, 14.2.

^{13}C NMR {^{19}F} (126 MHz, CDCl$_3$): δ 159.6, 146.2, 131.5, 114.6, 111.6, 109.0, 70.4, 59.5, 31.8, 27.1, 27.0, 22.7, 14.2.

HRMS-ESI (m/z): $[M+H]^+$ calculated for $[C_{13}H_{19}ONF]^+$:224.1445, found: 224.1450.

1-Benzyl-6-phenyl-1,3-dihydrobenzo[*c*]isoxazole (36):

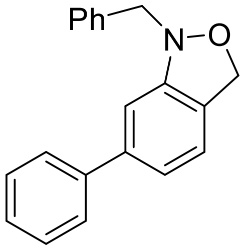

To an oven dried flask was added 1-benzyl-6-bromo-1,3-dihydrobenzo[c]isoxazole (20 mg, 0.069 mmol, 1 eq), SPhos Pd G3 (2.5 mg 0.0035 mmol, 0.05 eq), and phenyl boronic acid (10 mg, 0.083 mmol, 1.2 eq). The flask was purged with nitrogen before the mixture was dissolved in anhydrous 1,4-dioxane (0.692 mL, 0.1M) followed by 0.5M K_3PO_4 (0.166mL, 0.083 mmol, 1.2 eq) and heating to 90 °C. After 3 h, the reaction was cooled to room temperature and the crude mixture diluted with ether and filtered through celite. The resultant mixture was washed with 5 mL NH_4Cl and this aqueous layer extracted 3 x 3 mL with ether. The combined organic layers were dried over Na_2SO_4, concentrated in vacuo, and purified via flash column chromatography on silica (eluent: 40:60 DCM:Hexanes) to afford the title compound **36** (15 mg, 0.51 mmol, 74%) as a clear yellow oil.

TLC: R_f= 0.56 (20:80 EtOAc:Hexanes; UV, CAM)

^{1}H NMR (500 MHz, $CDCl_3$): δ 7.54 (d, *J* = 7.0 Hz, 2H), 7.43 (t, *J* = 8.2 Hz, 4H), 7.35 (t, *J* = 7.2 Hz, 3H), 7.30 (t, *J* = 7.2 Hz, 1H), 7.24 (d, *J* = 6.3 Hz, 1H), 7.18 (d, *J* = 7.7 Hz, 1H), 6.94 (s, 1H), 5.17 (s, 2H), 4.47 (s, 2H).

^{13}C NMR (126 MHz, $CDCl_3$): δ 150.3, 141.7, 141.1, 136.5, 129.3, 129.2, 128.9, 128.6, 127.8, 127.6, 127.3, 122.9, 121.9, 110.2, 70.9, 63.00.

HRMS-ESI (m/z): $[M+H]^+$ calculated for $[C_{20}H_{18}ON]^+$:288.1383, found: 288.1381.

1-Benzyl-6-morpholino-1,3-dihydrobenzo[*c*]isoxazole (37):

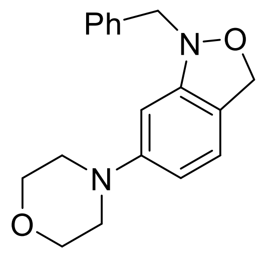

To an oven dried flask was added 1-benzyl-6-bromo-1,3-dihydrobenzo[c]isoxazole (20 mg, 0.069 mmol, 1 eq), SPhos Pd G3 (4.9 mg 0.0069 mmol, 0.10 eq), SPhos (5.7 mg, 0.014 mmol, 0.2 eq), morpholine (0.03 mL, 0.35 mmol, 5 eq), and NaOtBu (8.6 mg, 0.090 mmol, 1.3 eq). The flask was purged with nitrogen before the mixture was dissolved in anhydrous toluene (0.346 mL, 0.2M) and heated to 100 °C. After 2 h, the reaction mixture was cooled to room temperature, diluted with ether, and filtered through celite. The resultant mixture was concentrated in vacuo and purified via flash column chromatography on alumina (eluent: 20:80 EtOAc:Hexanes) to afford the title compound **37** (11 mg, 0.036 mmol, 53%) as a yellow white solid.

TLC: R_f= 0.38 (30:70 EtOAc:Hexanes; UV, CAM)

^{1}H NMR (500 MHz, $CDCl_3$): δ 7.42 (d, *J* = 8.1 Hz, 2H), 7.32 (dt, *J* = 25.0, 7.6 Hz, 3H), 7.02 (d, *J* = 8.3 Hz, 1H), 6.59 (d, *J* = 8.3 Hz, 1H), 6.28 (s, 1H), 5.07 (s, 2H), 4.39 (s, 2H), 3.88 – 3.82 (m, 4H), 3.13 – 3.07 (m, 4H).

^{13}C NMR (126 MHz, $CDCl_3$): δ 152.0, 150.9, 136.7, 129.1, 128.5, 127.7, 122.0, 121.9, 111.5, 99.6, 70.8, 67.0, 63.0, 50.0.

HRMS-ESI (m/z): $[M+H]^+$ calculated for $[C_{18}H_{21}O_2N_2]^+$:297.1598, found: 297.1594.

^{1}H NMR (500 MHz, CDCl3) spectrum of 1-benzylbenzo[*c*]isoxazol-3(*1H*)-one (**6**):

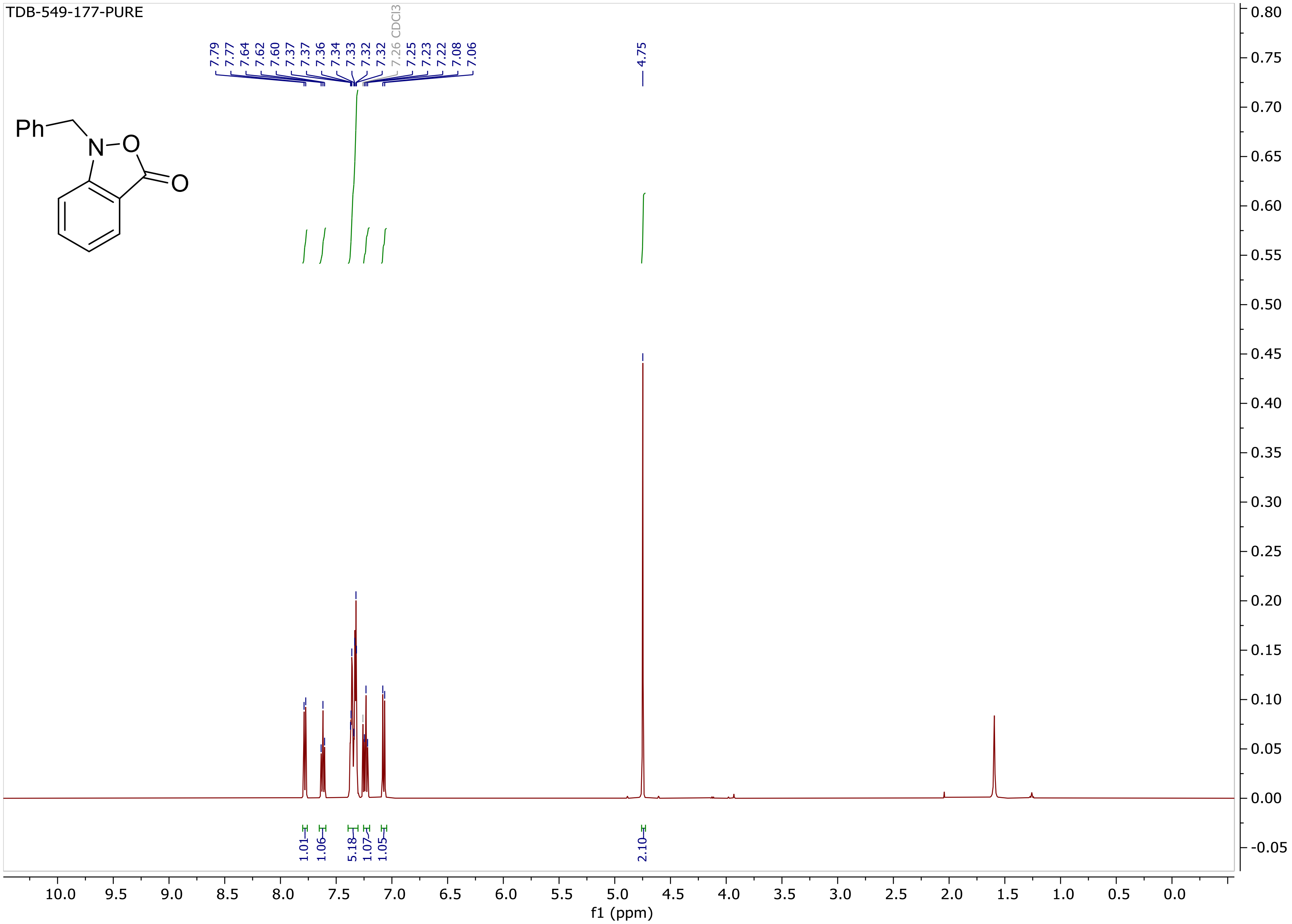

^{13}C NMR (126 MHz, CDCl$_3$) spectrum of 1-benzylbenzo[*c*]isoxazol-3(*1H*)-one (**6**):

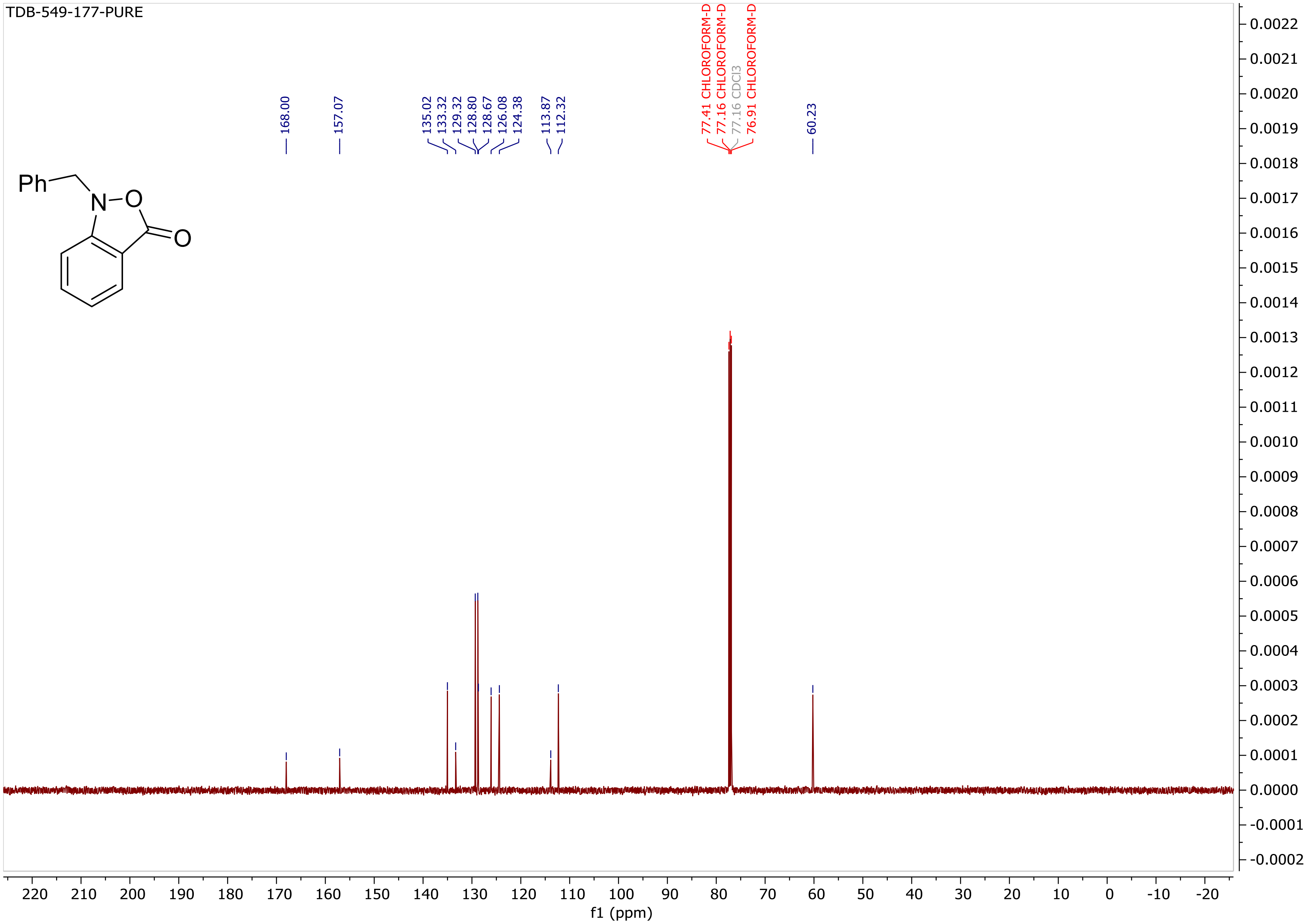

^{1}H NMR (500 MHz, CDCl₃) spectrum of 1-allylbenzo[*c*]isoxazol-3(*1H*)-one (**7**):

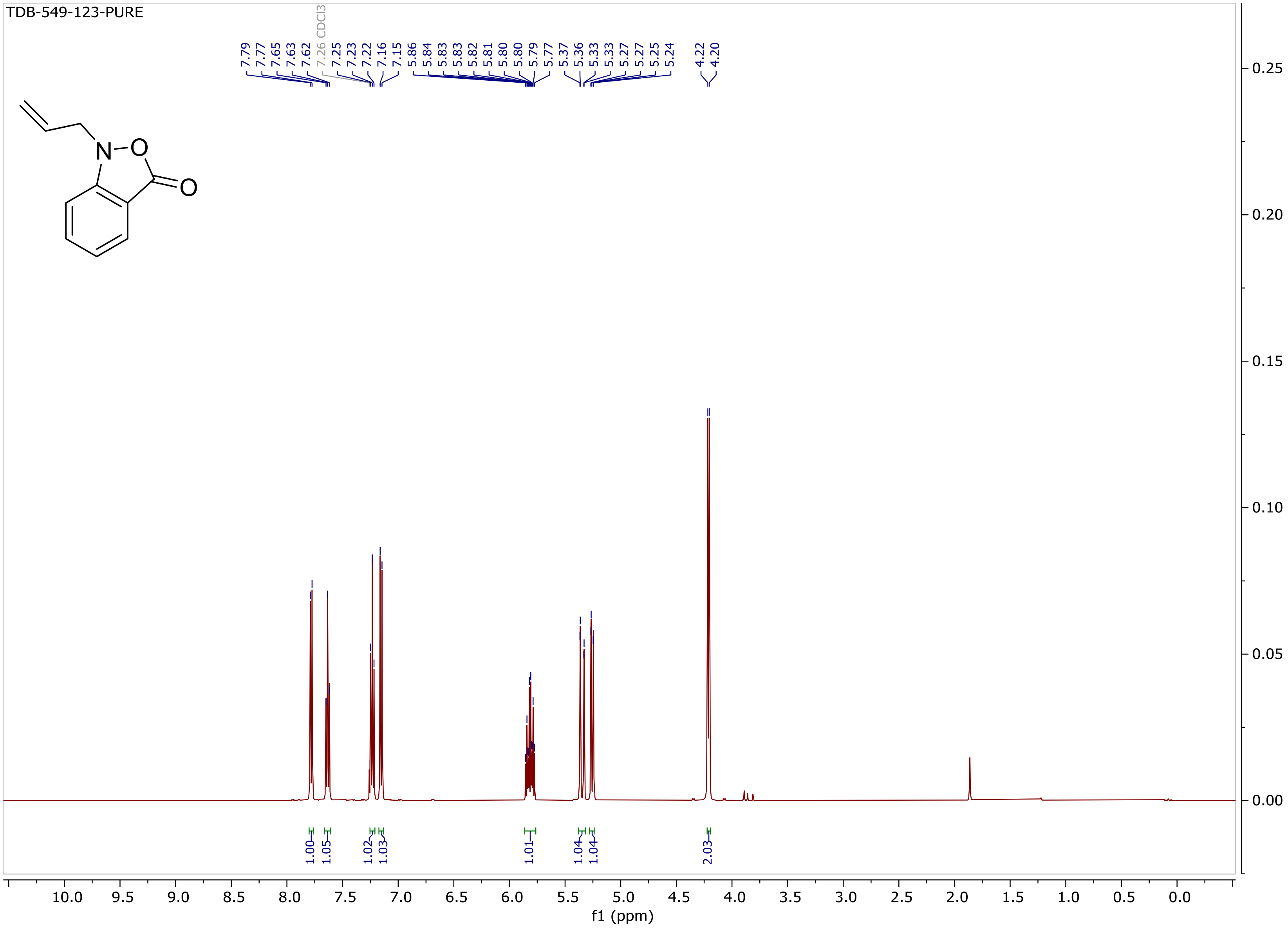

^{13}**C NMR (126 MHz, CDCl$_3$)** spectrum of 1-allylbenzo[*c*]isoxazol-3(*1H*)-one (**7**):

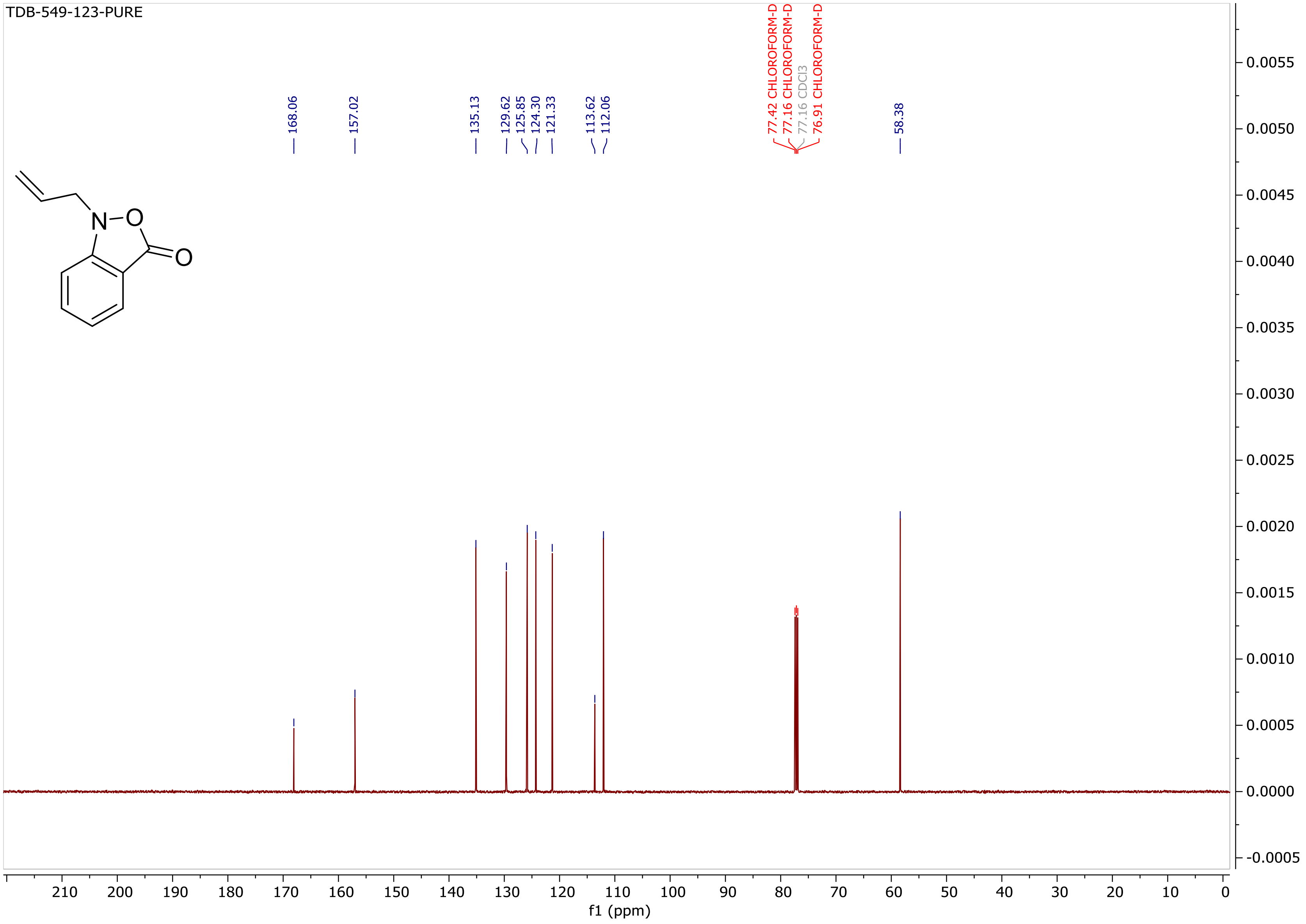

HSQC NMR (500 MHz, CDCl₃) spectrum of 1-allylbenzo[*c*]isoxazol-3(*1H*)-one (**7**):

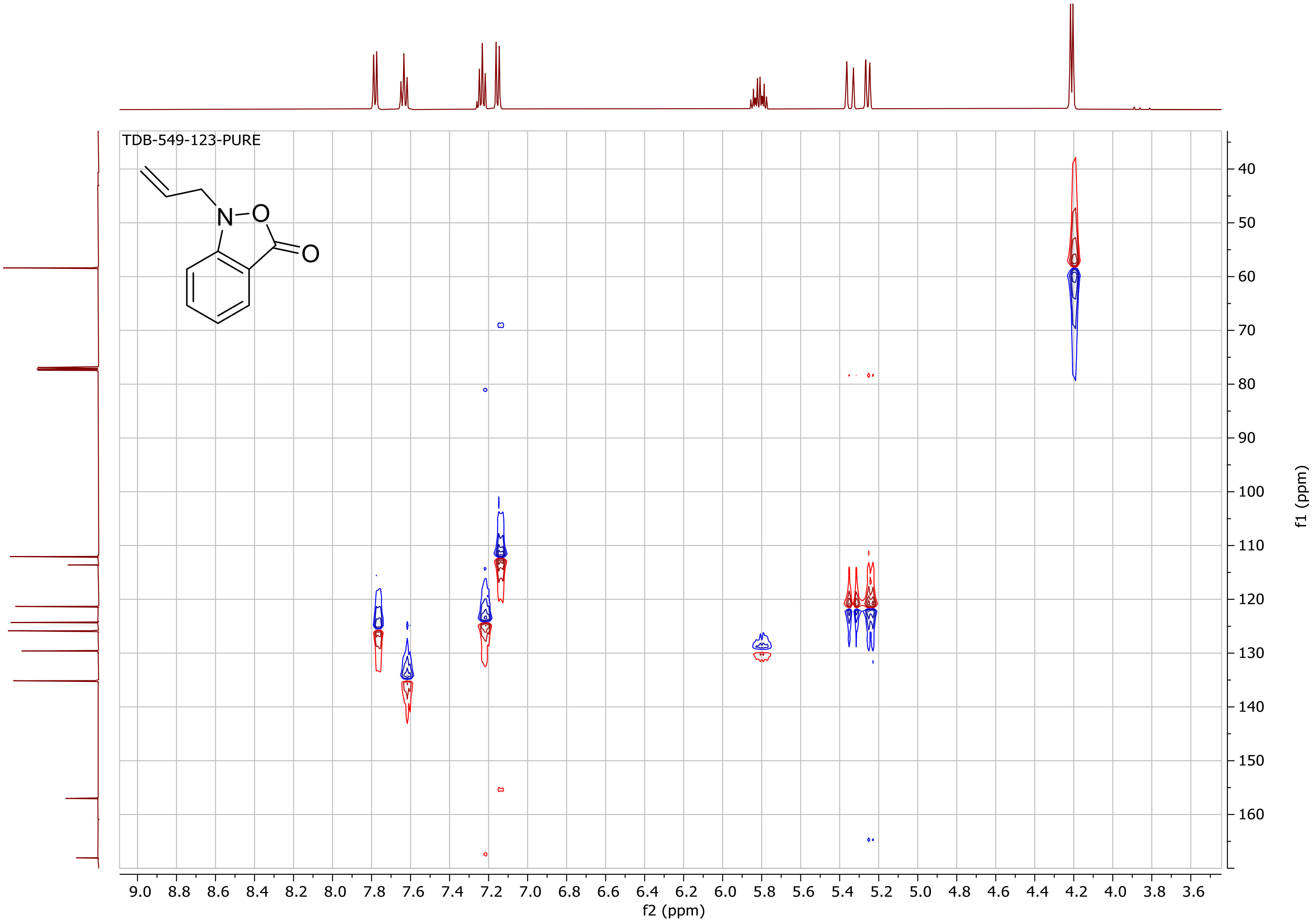

HMBC NMR (500 MHz, CDCl₃) spectrum of 1-allylbenzo[*c*]isoxazol-3(*1H*)-one (**7**):

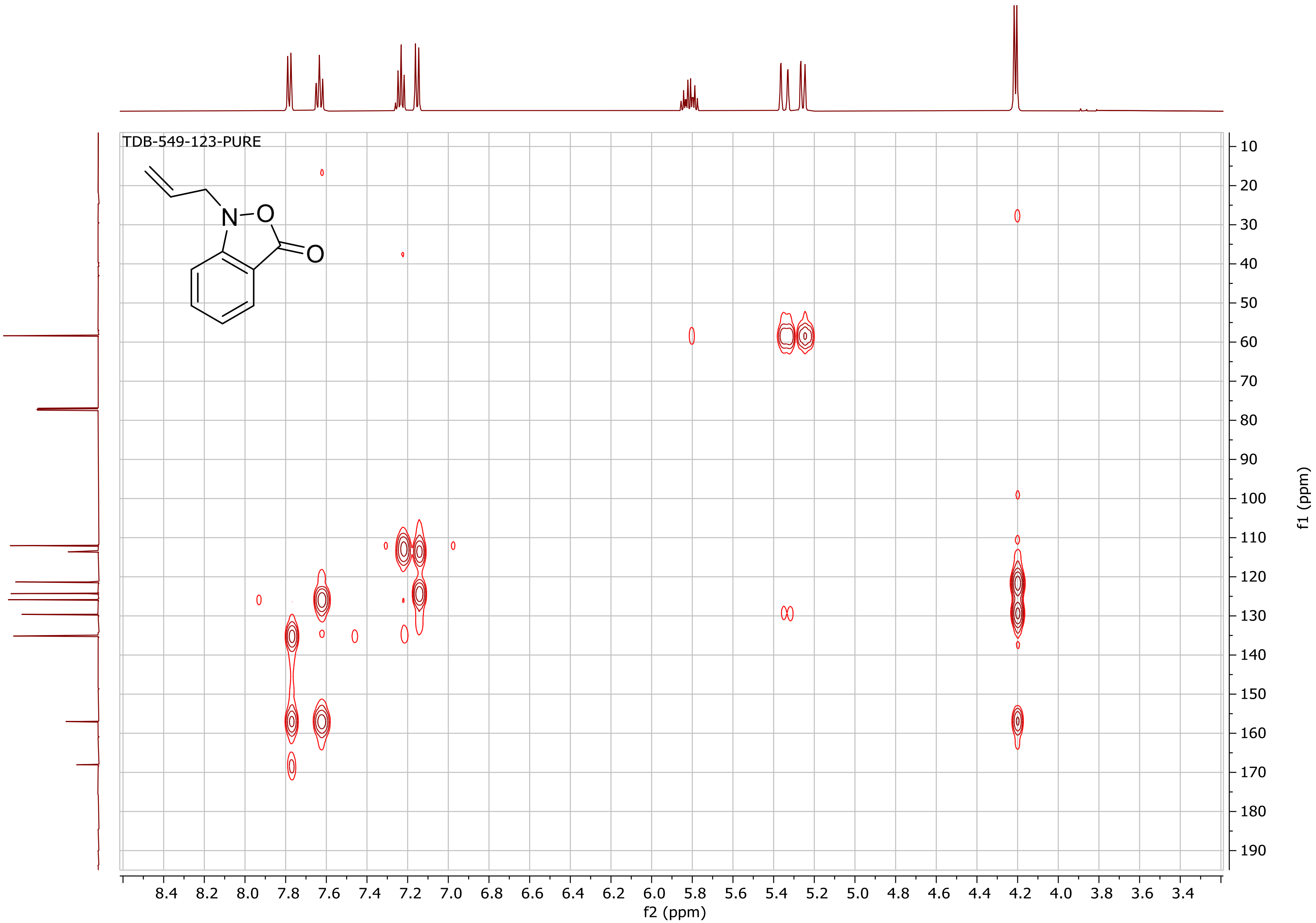

^{1}H NMR (500 MHz, $CDCl_3$) spectrum of 1-benzyl-5-methylbenzo[c]isoxazol-3(1H)-one (**8**):

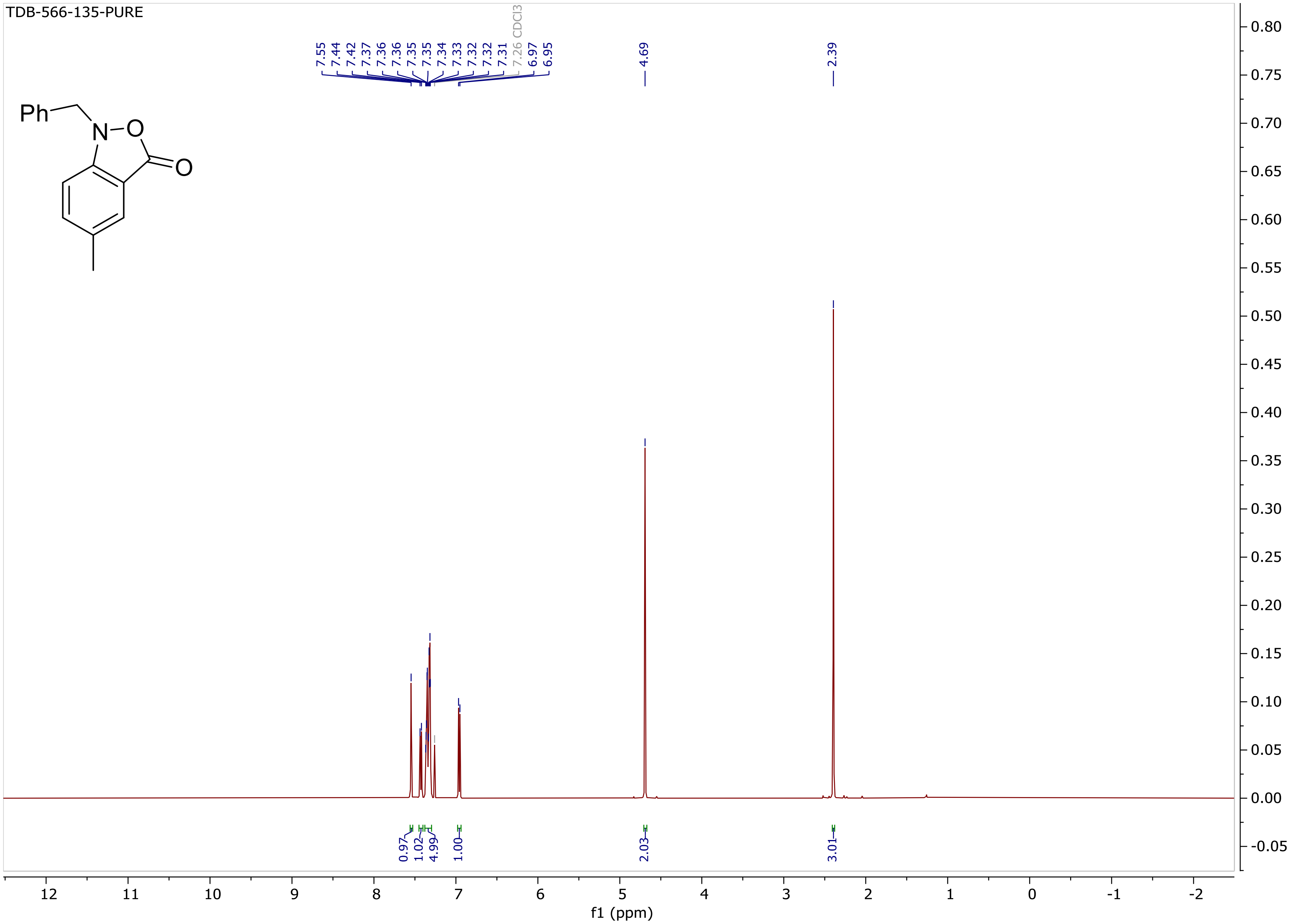

^{13}C NMR (126 MHz, $CDCl_3$) spectrum of 1-benzyl-5-methylbenzo[c]isoxazol-3(1H)-one (**8**):

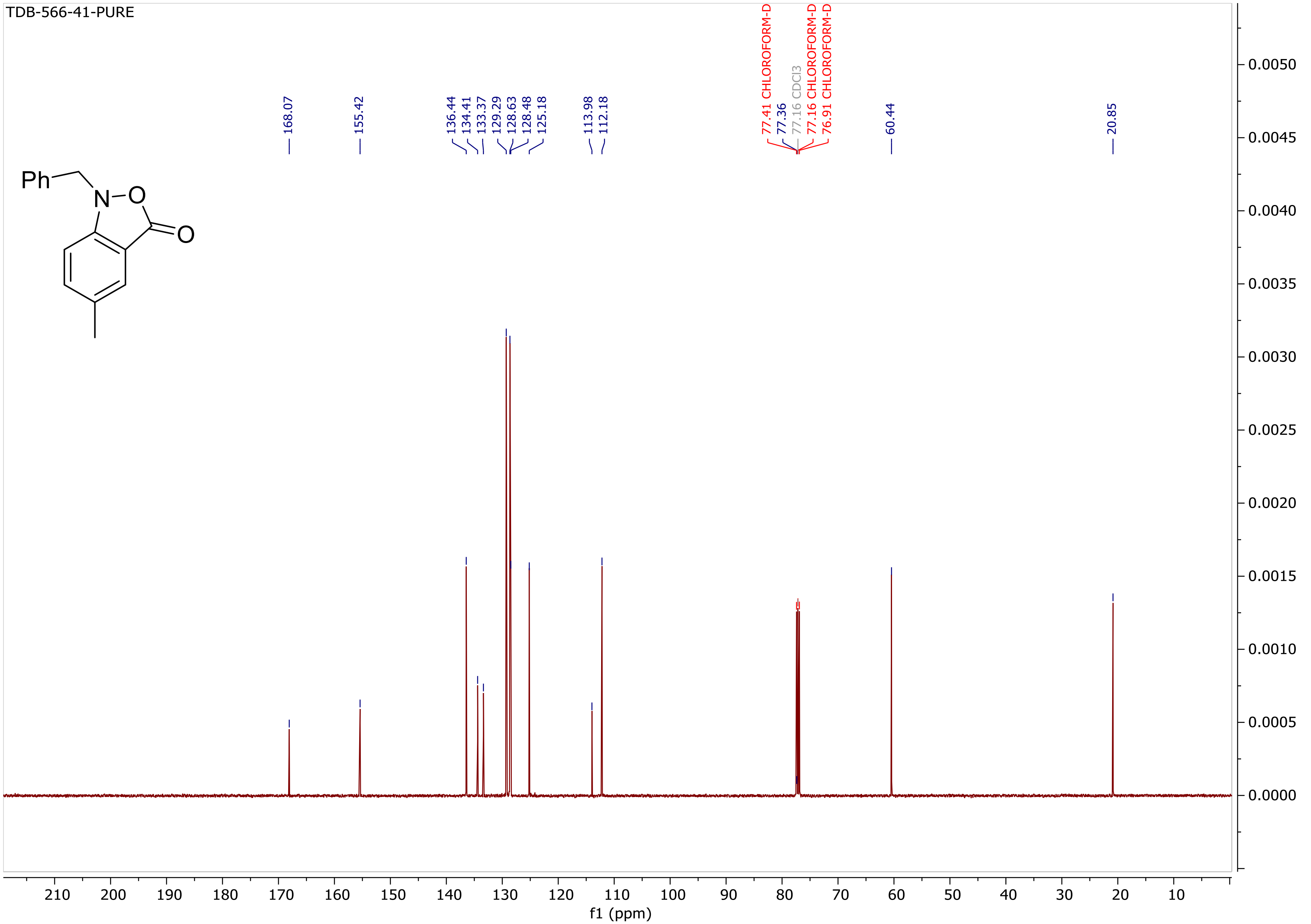

^{1}H NMR (500 MHz, CDCl₃) spectrum of 1-benzyl-5-bromobenzo[c]isoxazol-3(1H)-one (**9**):

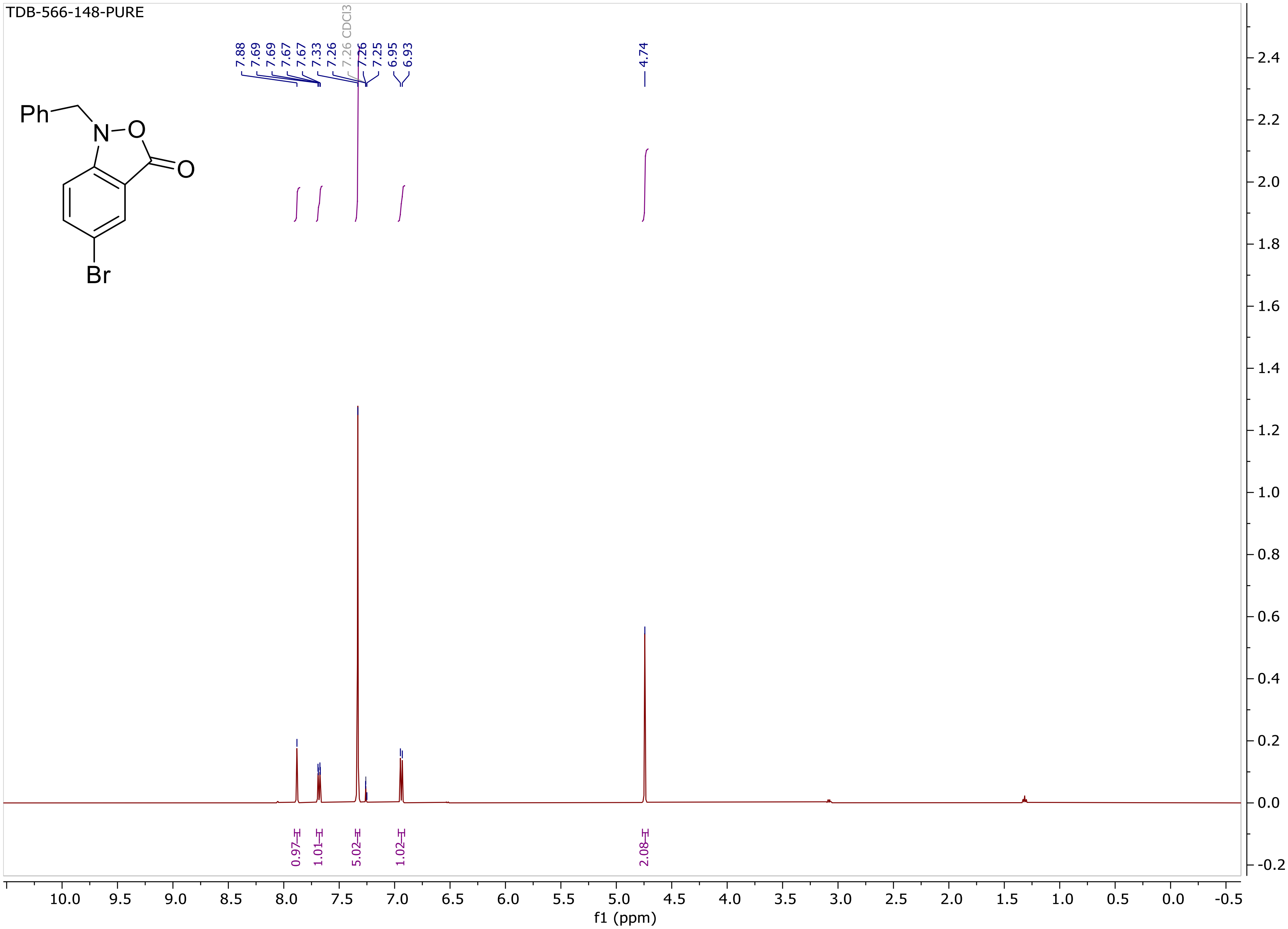

^{13}C NMR (126 MHz, CDCl$_3$) spectrum of 1-benzyl-5-bromobenzo[c]isoxazol-3(1H)-one (**9**):

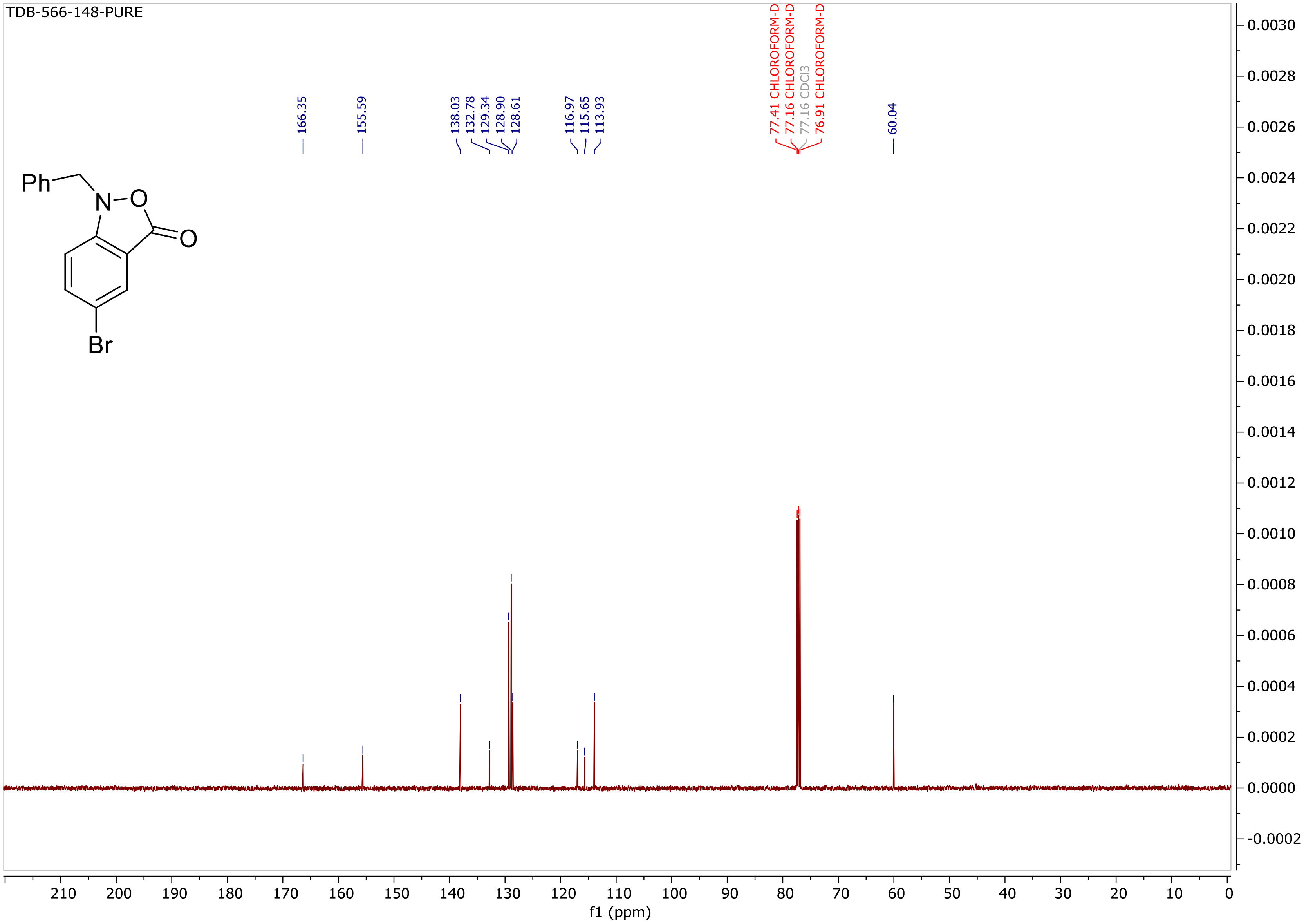

^{1}H NMR (500 MHz, CDCl$_3$) spectrum of 1-allyl-7-fluorobenzo[c]isoxazol-3(1H)-one (**10**):

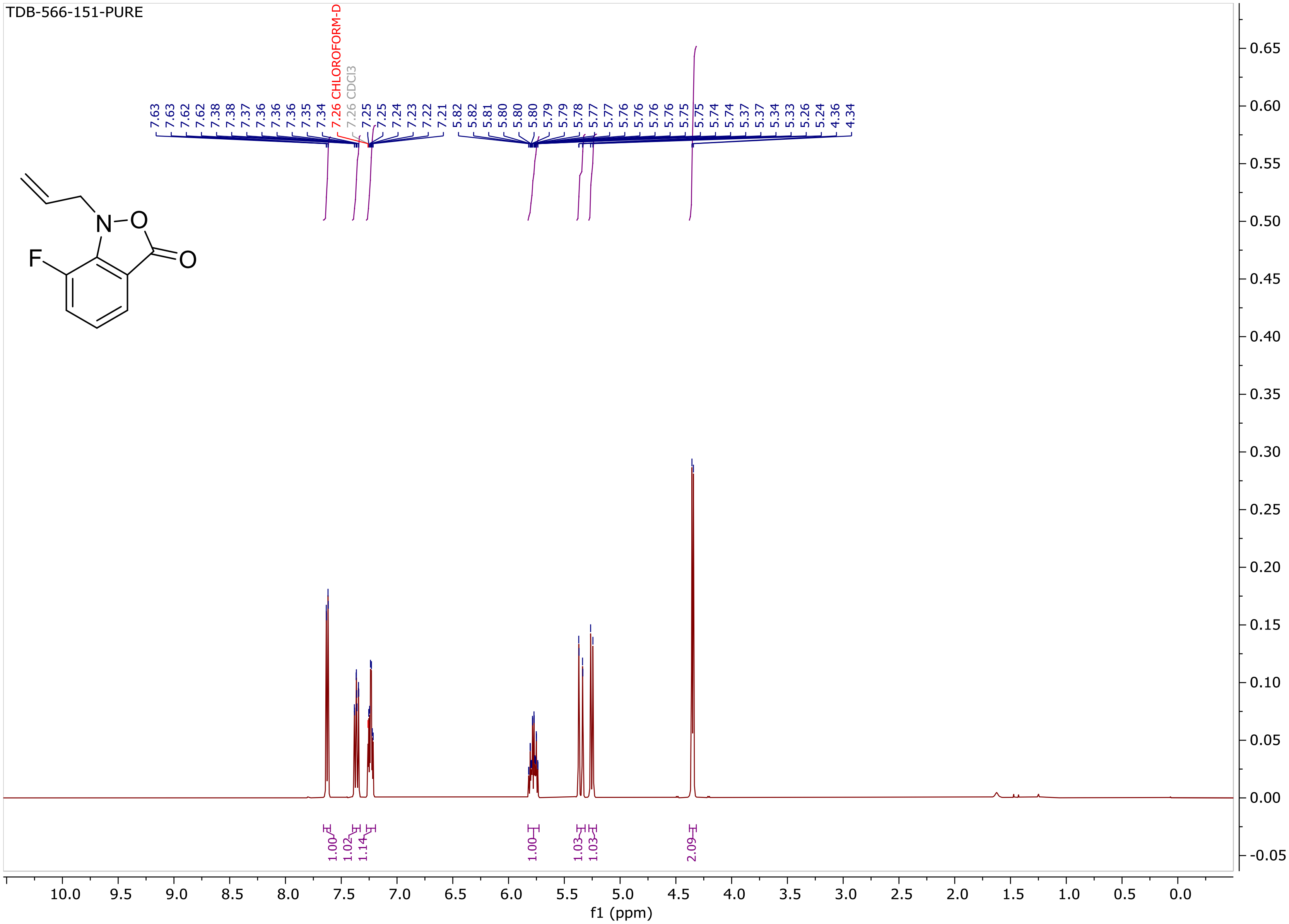

^{13}C NMR (126 MHz, CDCl$_3$) spectrum of 1-allyl-7-fluorobenzo[c]isoxazol-3(1H)-one (**10**):

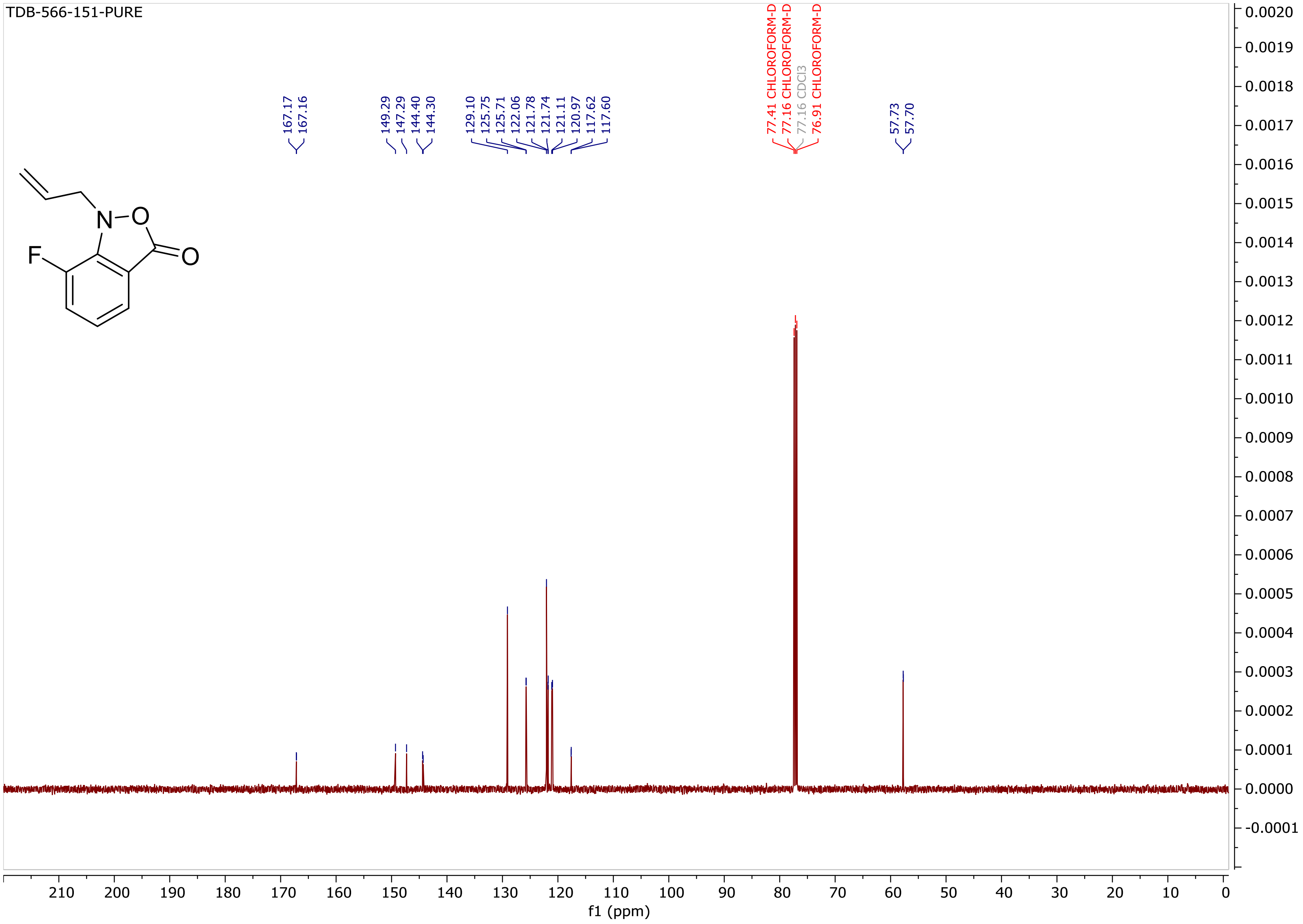

^{13}**C NMR {^{19}F} (126 MHz, CDCl$_3$)** spectrum of 1-allyl-7-fluorobenzo[c]isoxazol-3(1H)-one (**10**):

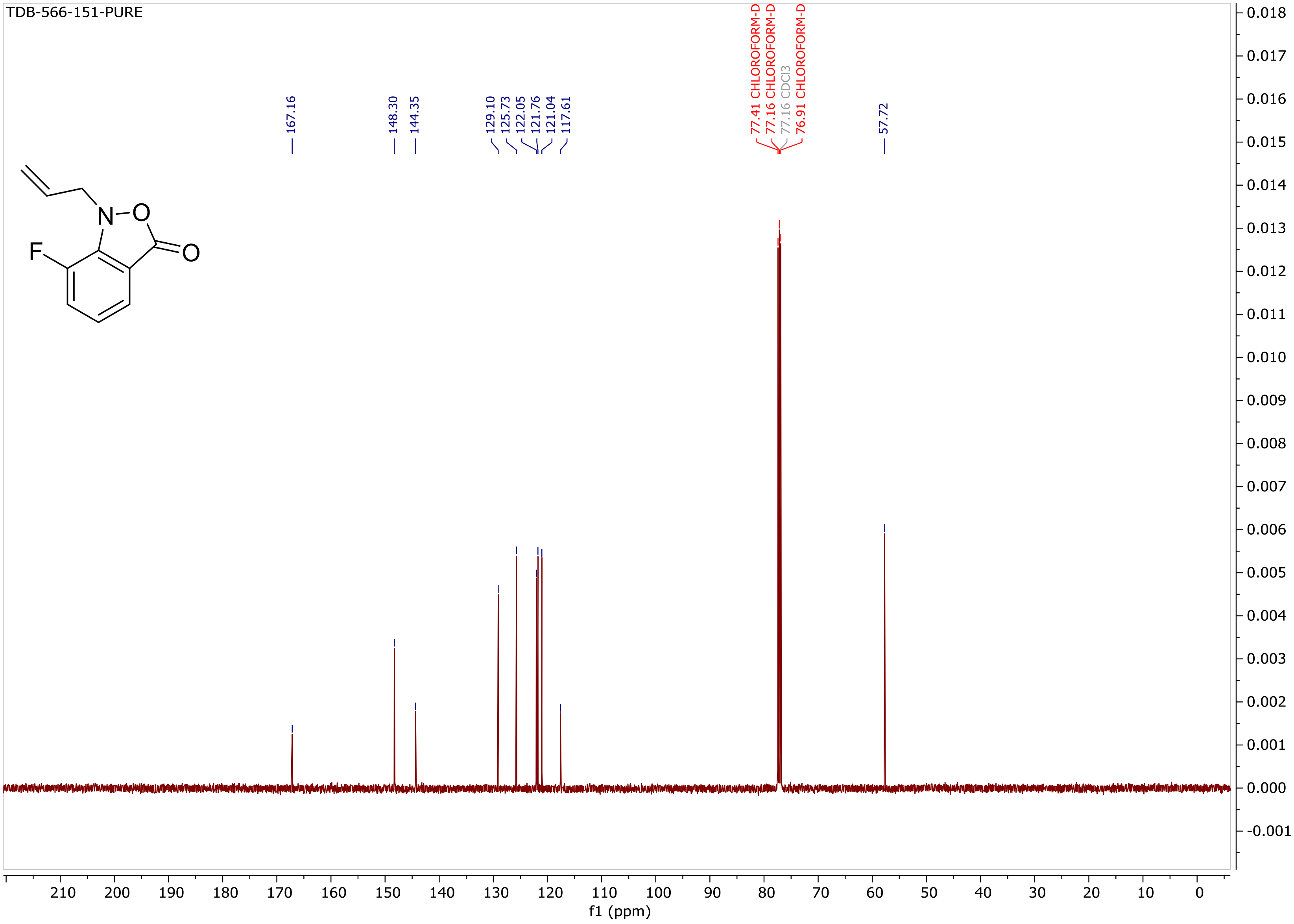

Expanded region of stacked (top) ^{13}C NMR (126 MHz, $CDCl_3$) and (bottom) ^{13}C NMR {^{19}F} (126 MHz, $CDCl_3$) spectrum of 1-allyl-7-fluorobenzo[c]isoxazol-3(1H)-one (**10**):

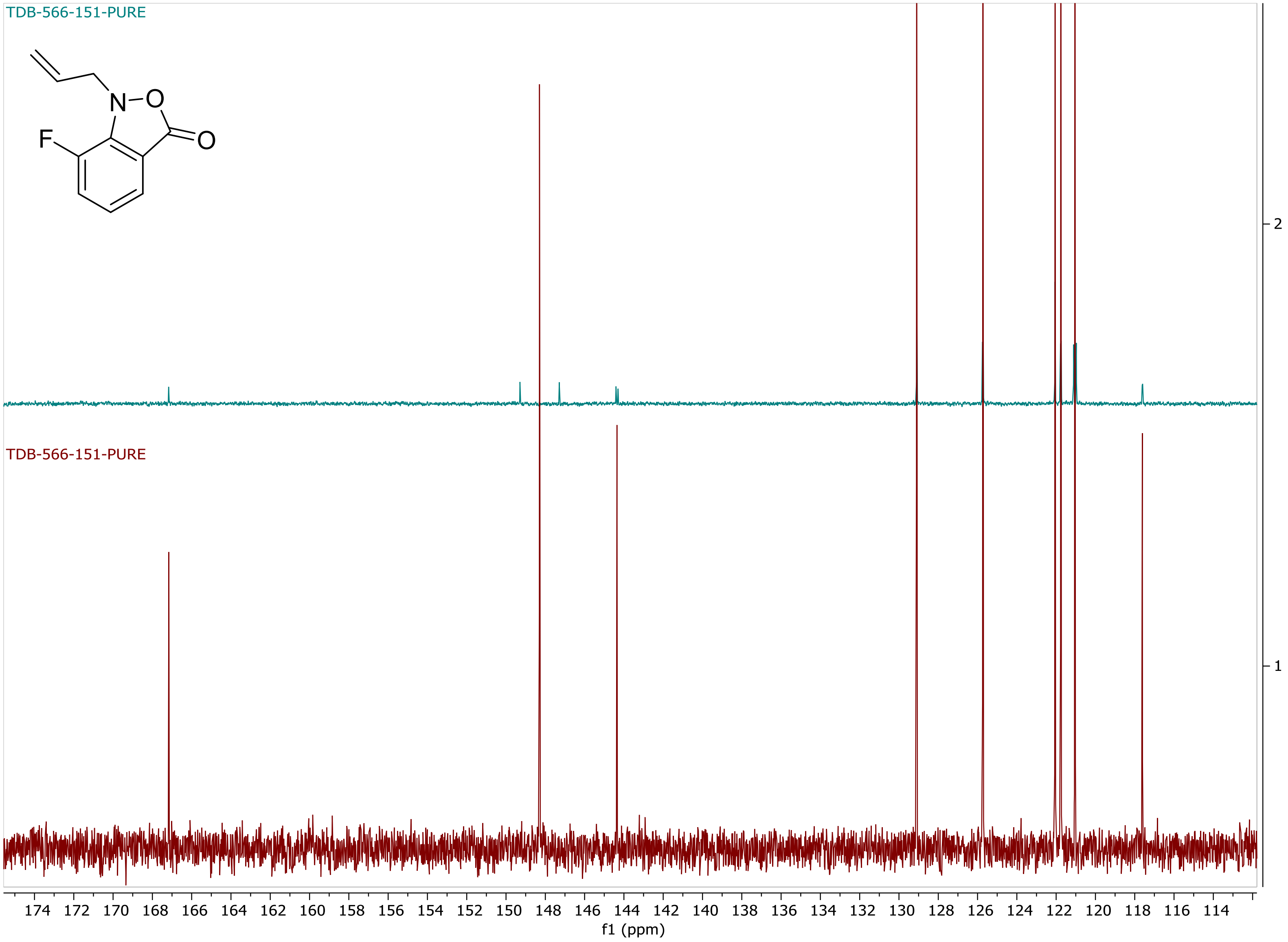

^{19}F NMR {1H} (470 MHz, $CDCl_3$) spectrum of 1-allyl-7-fluorobenzo[c]isoxazol-3(1H)-one (**10**):

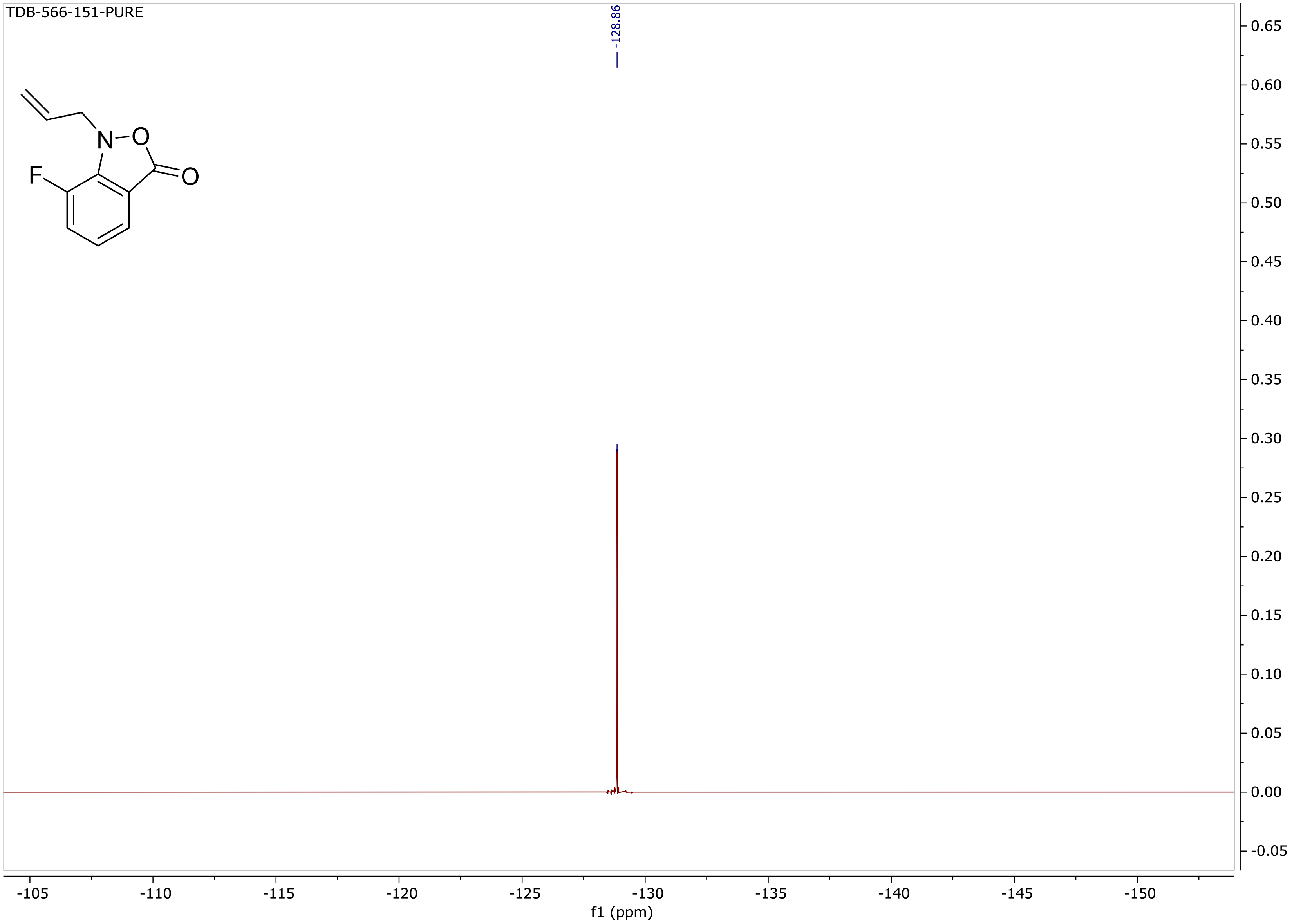

^{1}H NMR (500 MHz, $CDCl_3$) spectrum of 6-bromo-1-(4-bromobenzyl)benzo[c]isoxazol-3(1H)-one (**11**):

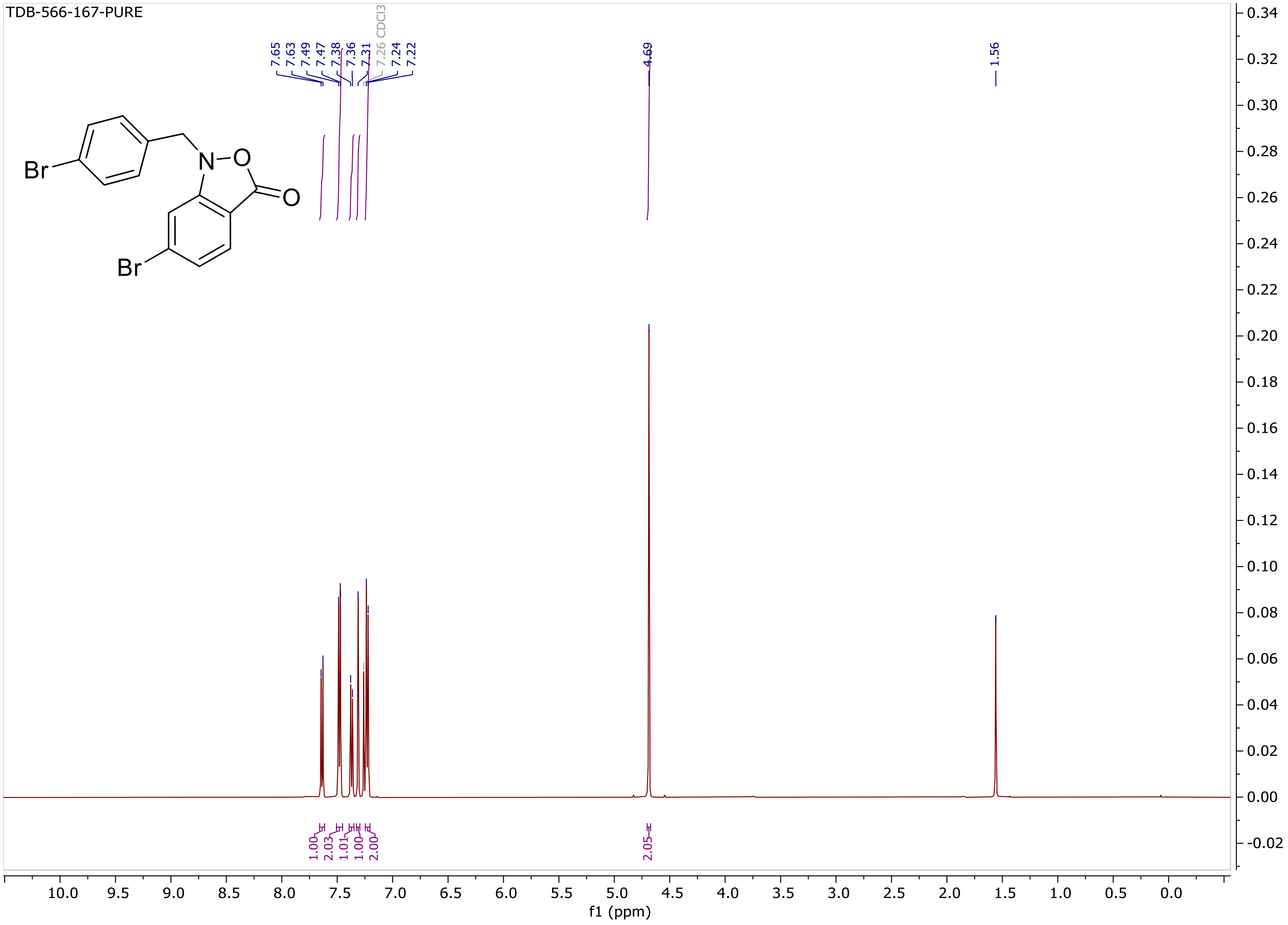

^{13}C NMR (126 MHz, CDCl$_3$) spectrum of 6-bromo-1-(4-bromobenzyl)benzo[c]isoxazol-3(1H)-one (**11**):

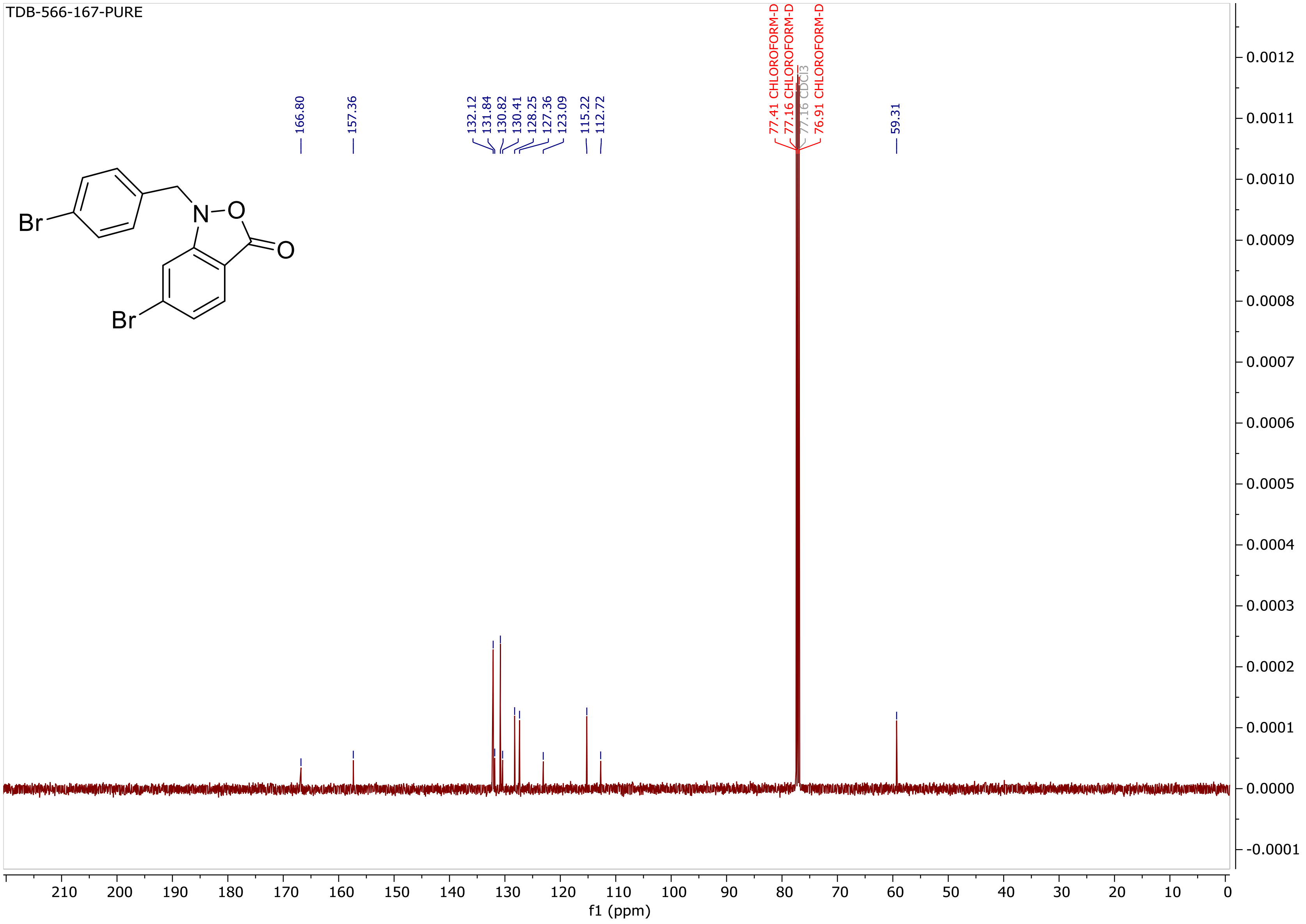

^{1}H NMR (500 MHz, $CDCl_3$) spectrum of 1-allyl-5-fluorobenzo[c]isoxazol-3(1H)-one (**12**):

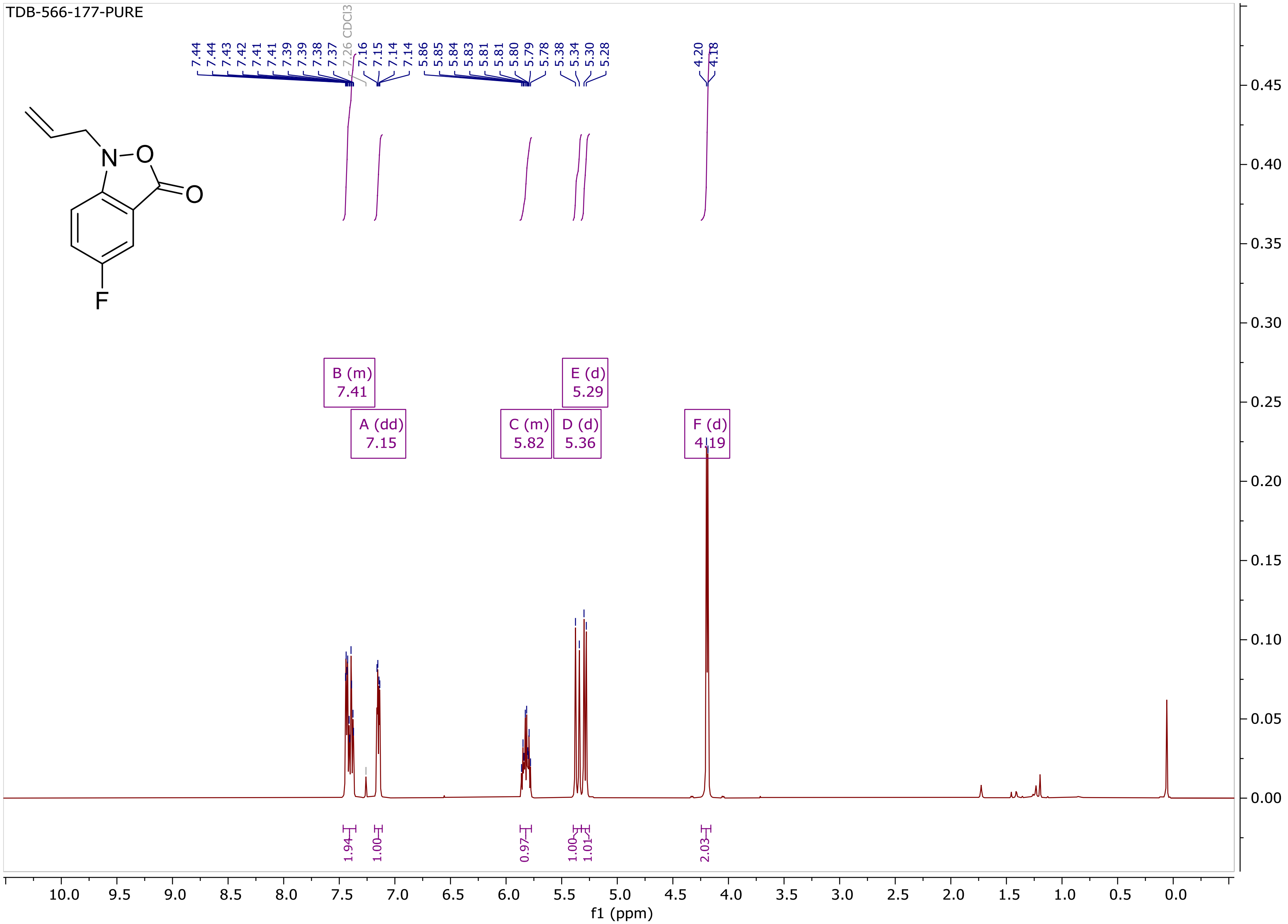

^{13}C NMR (126 MHz, CDCl$_3$) spectrum of 1-allyl-5-fluorobenzo[c]isoxazol-3(1H)-one (**12**):

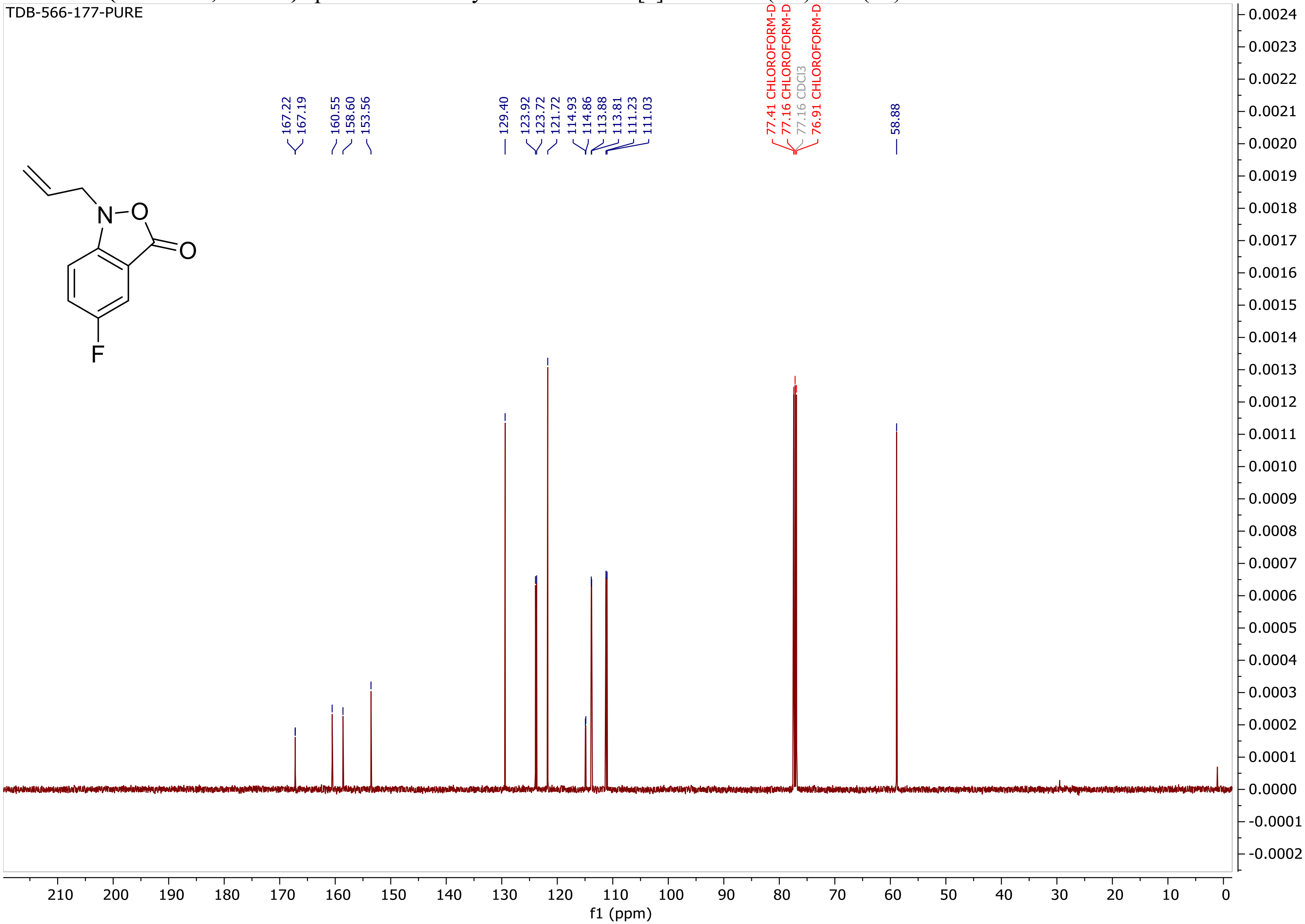

^{13}C NMR {^{19}F} (126 MHz, $CDCl_3$) spectrum of 1-allyl-5-fluorobenzo[c]isoxazol-3(1H)-one (**12**):

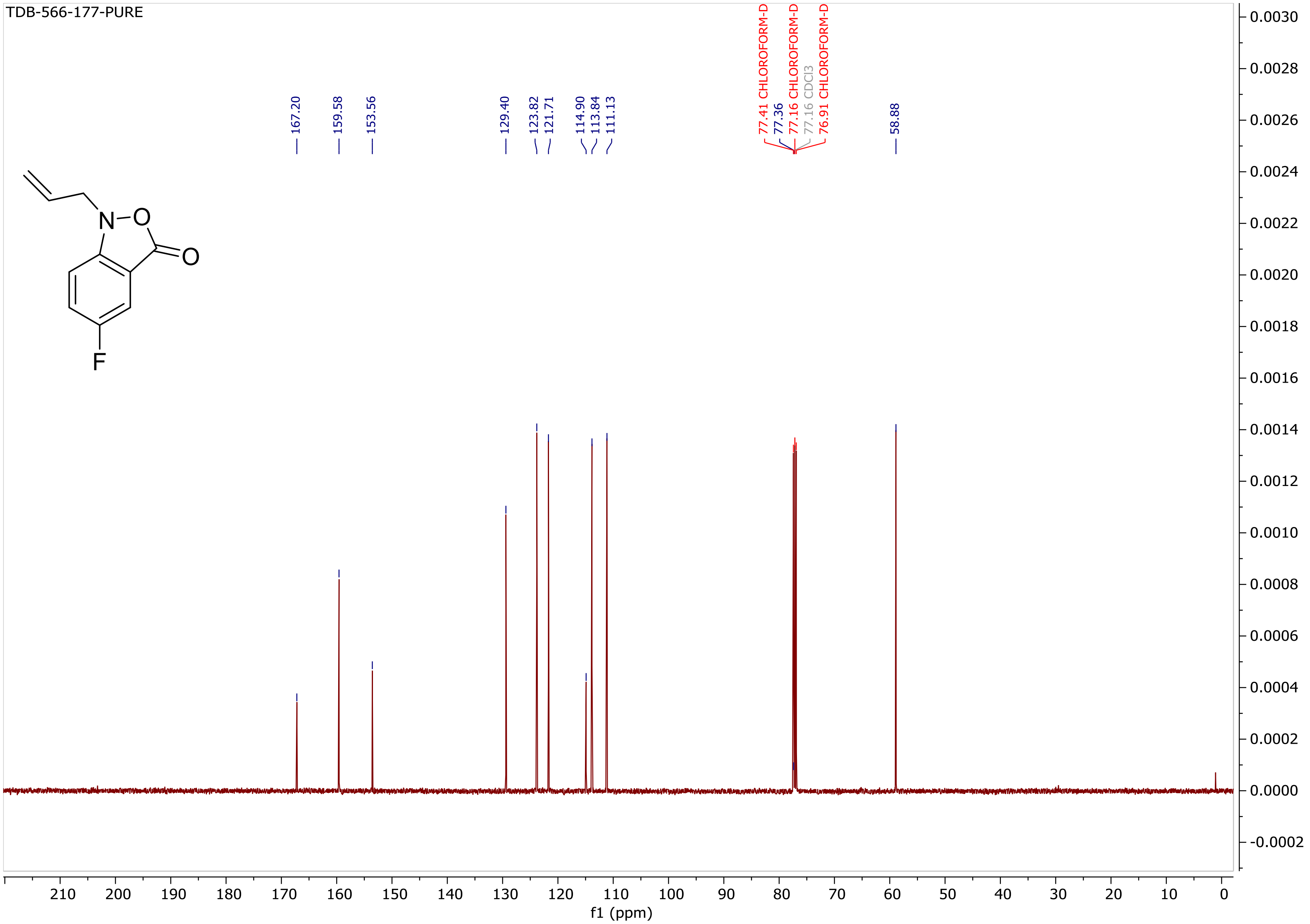

Expanded region of stacked (top) **^{13}C NMR (126 MHz, CDCl$_3$)** and (bottom) **^{13}C NMR {^{19}F} (126 MHz, CDCl$_3$)** spectrum of 1-allyl-5-fluorobenzo[c]isoxazol-3(1H)-one (**12**):

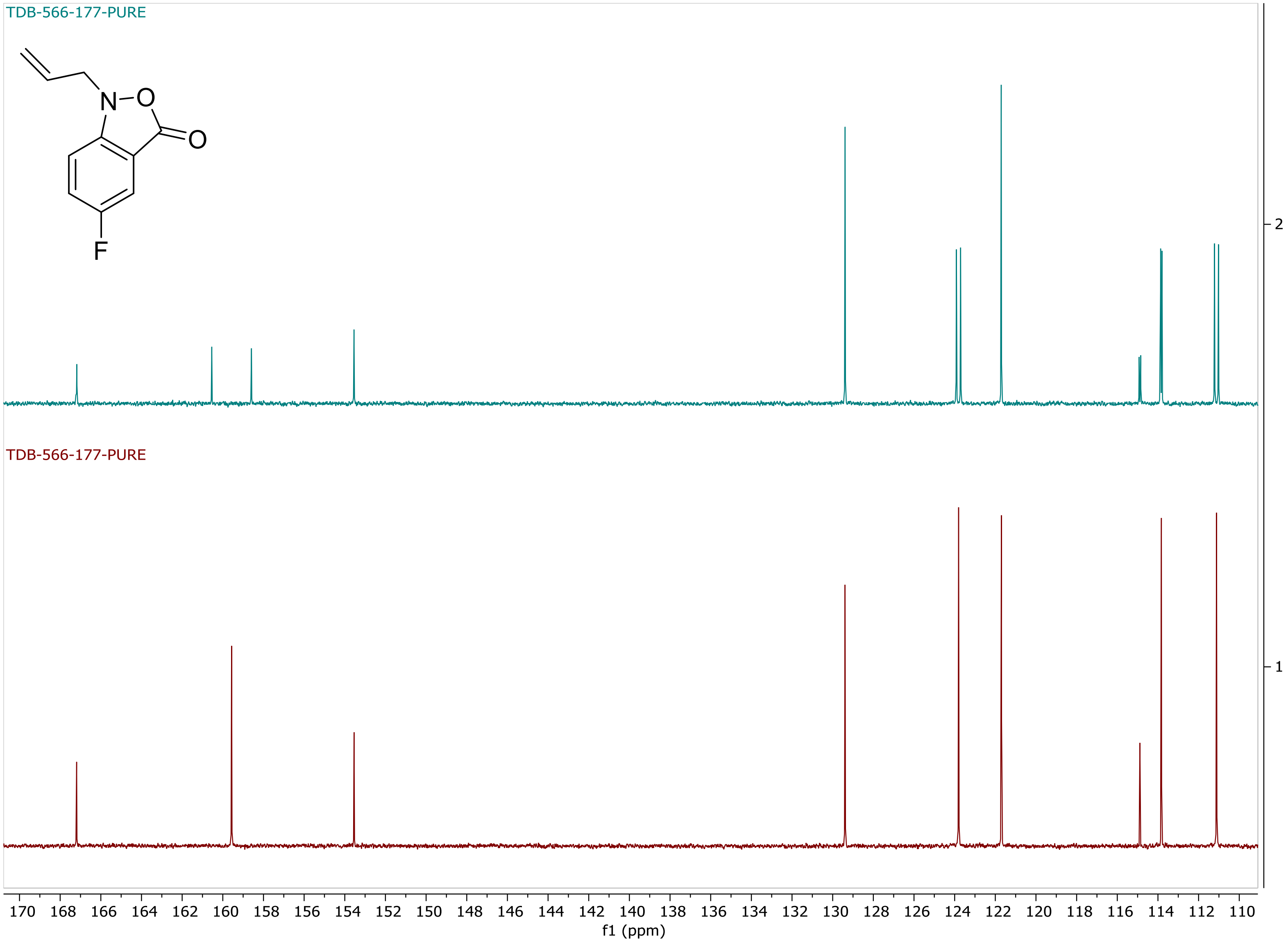

^{19}F NMR {1H} (470 MHz, CDCl$_3$) spectrum of 1-allyl-5-fluorobenzo[c]isoxazol-3(1H)-one (**12**):

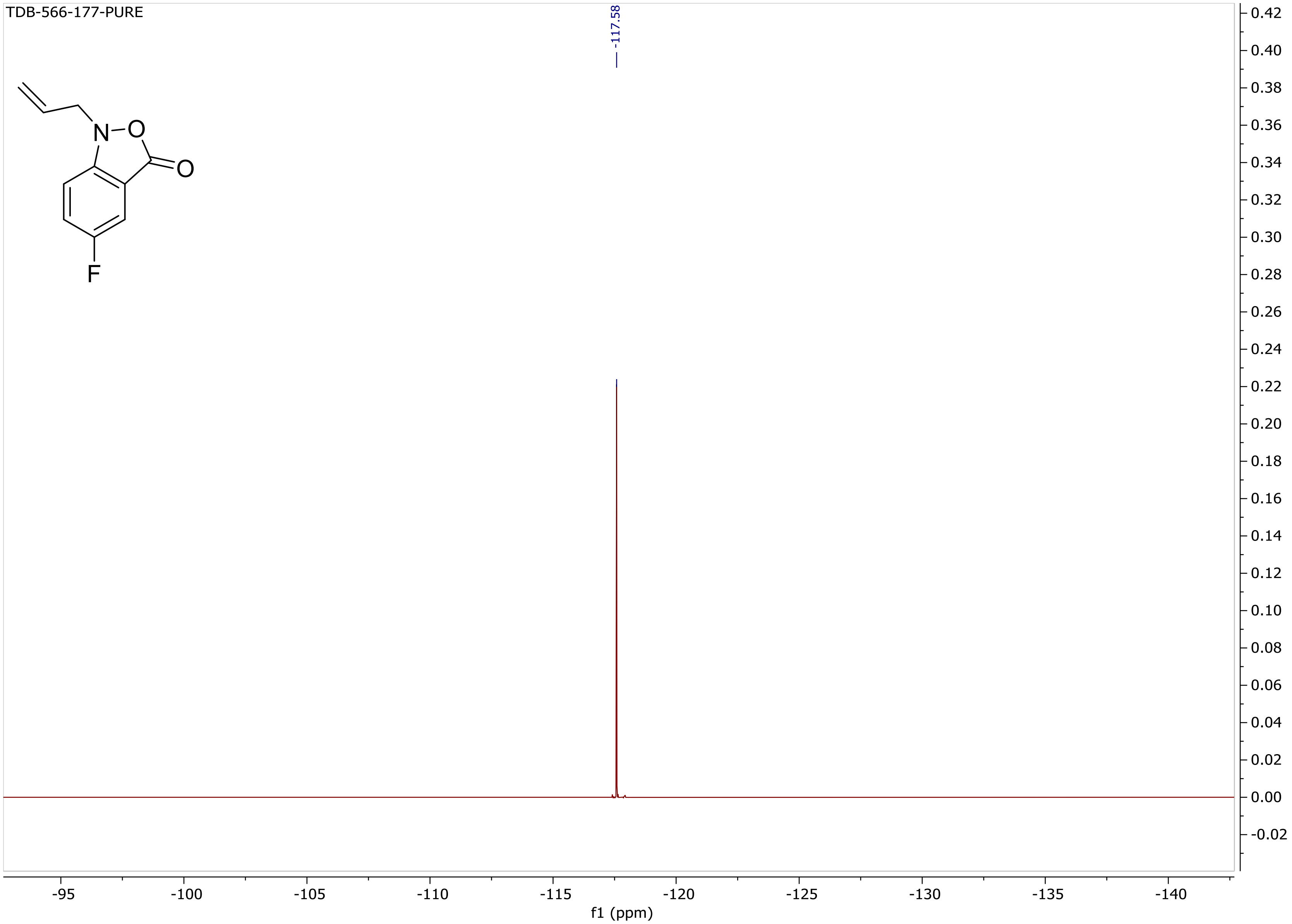

^{1}H NMR (500 MHz, CDCl₃) spectrum of 1-(4-bromobenzyl)-6-fluorobenzo[c]isoxazol-3(1H)-one (**13**):

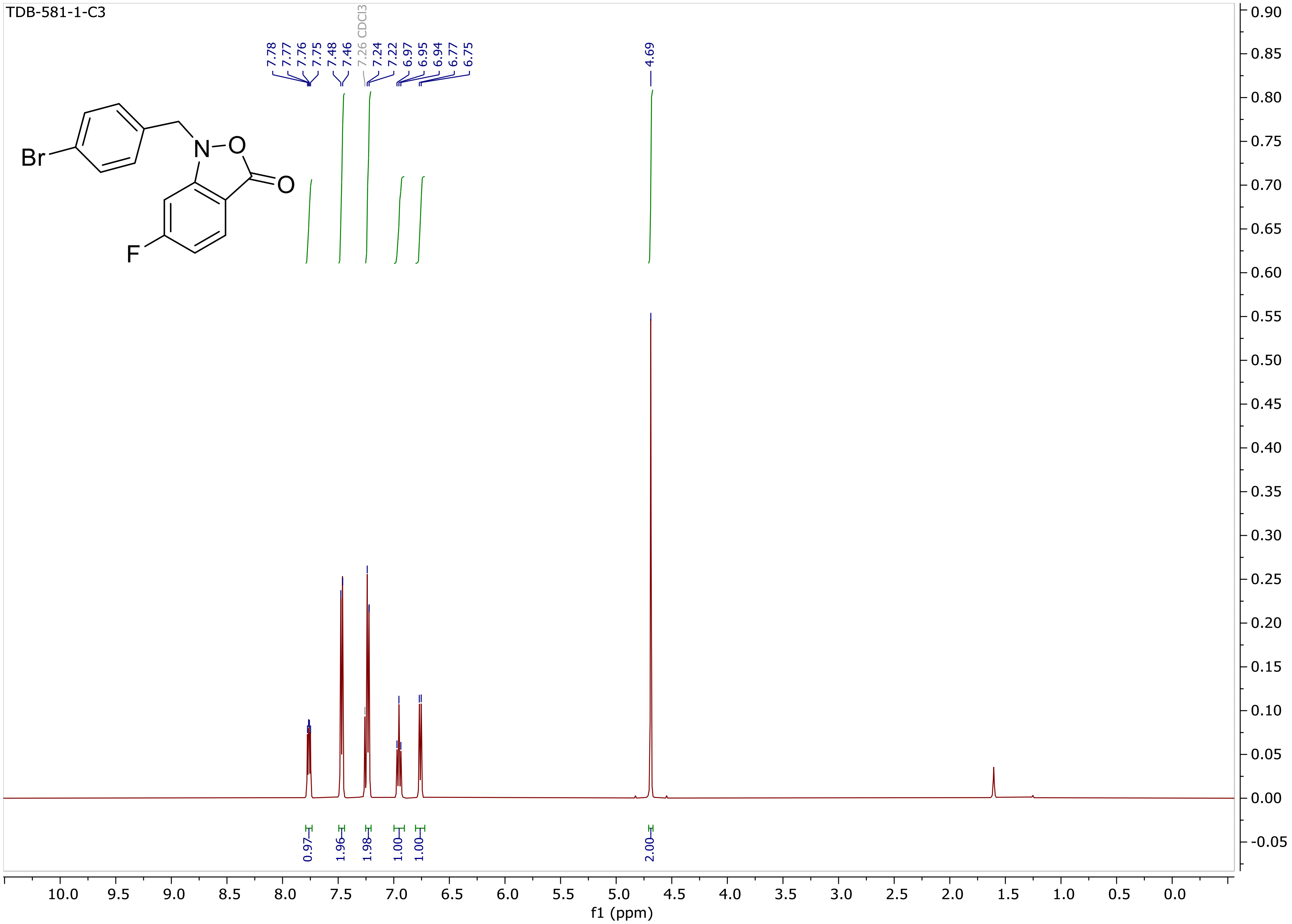

^{13}C NMR (126 MHz, CDCl$_3$) spectrum of 1-(4-bromobenzyl)-6-fluorobenzo[c]isoxazol-3(1H)-one (**13**):

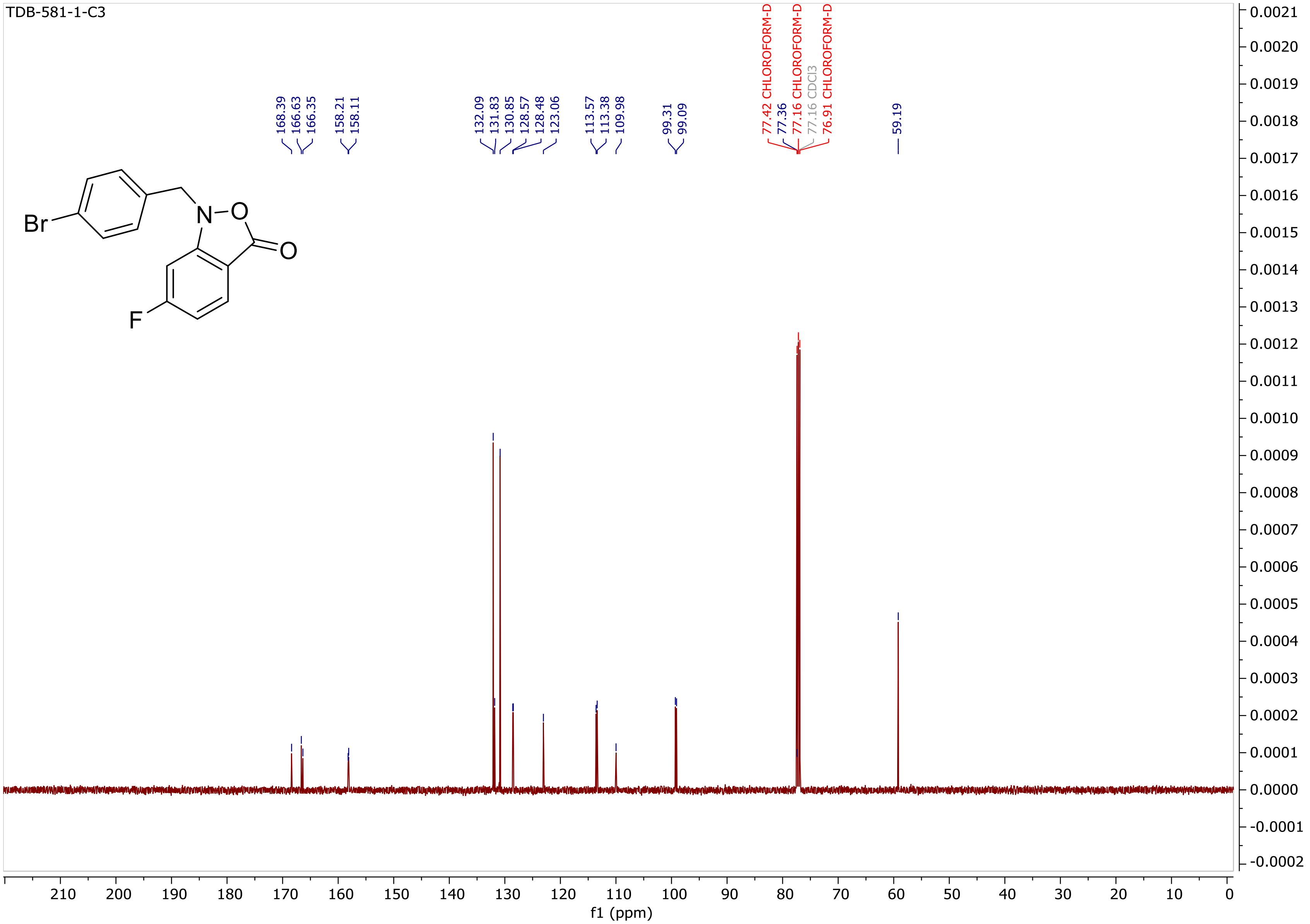

^{13}C NMR {^{19}F} (126 MHz, CDCl$_3$) spectrum of 1-(4-bromobenzyl)-6-fluorobenzo[c]isoxazol-3(1H)-one (**13**):

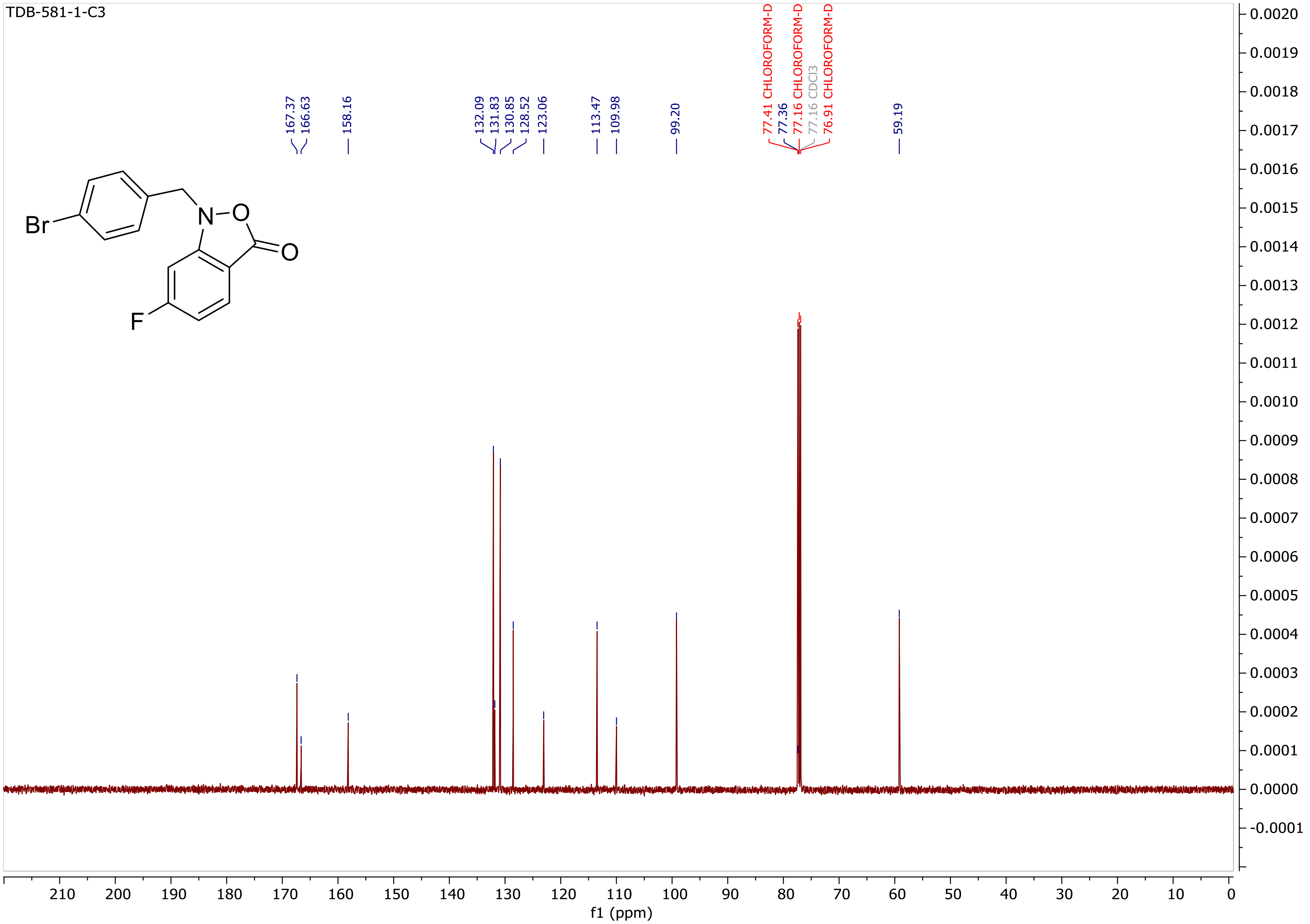

Expanded region of stacked (top) ^{13}C **NMR (126 MHz, CDCl$_3$)** and (bottom) ^{13}C **NMR {^{19}F} (126 MHz, CDCl$_3$)** spectrum of 1-(4-bromobenzyl)-6-fluorobenzo[c]isoxazol-3(1H)-one (**13**):

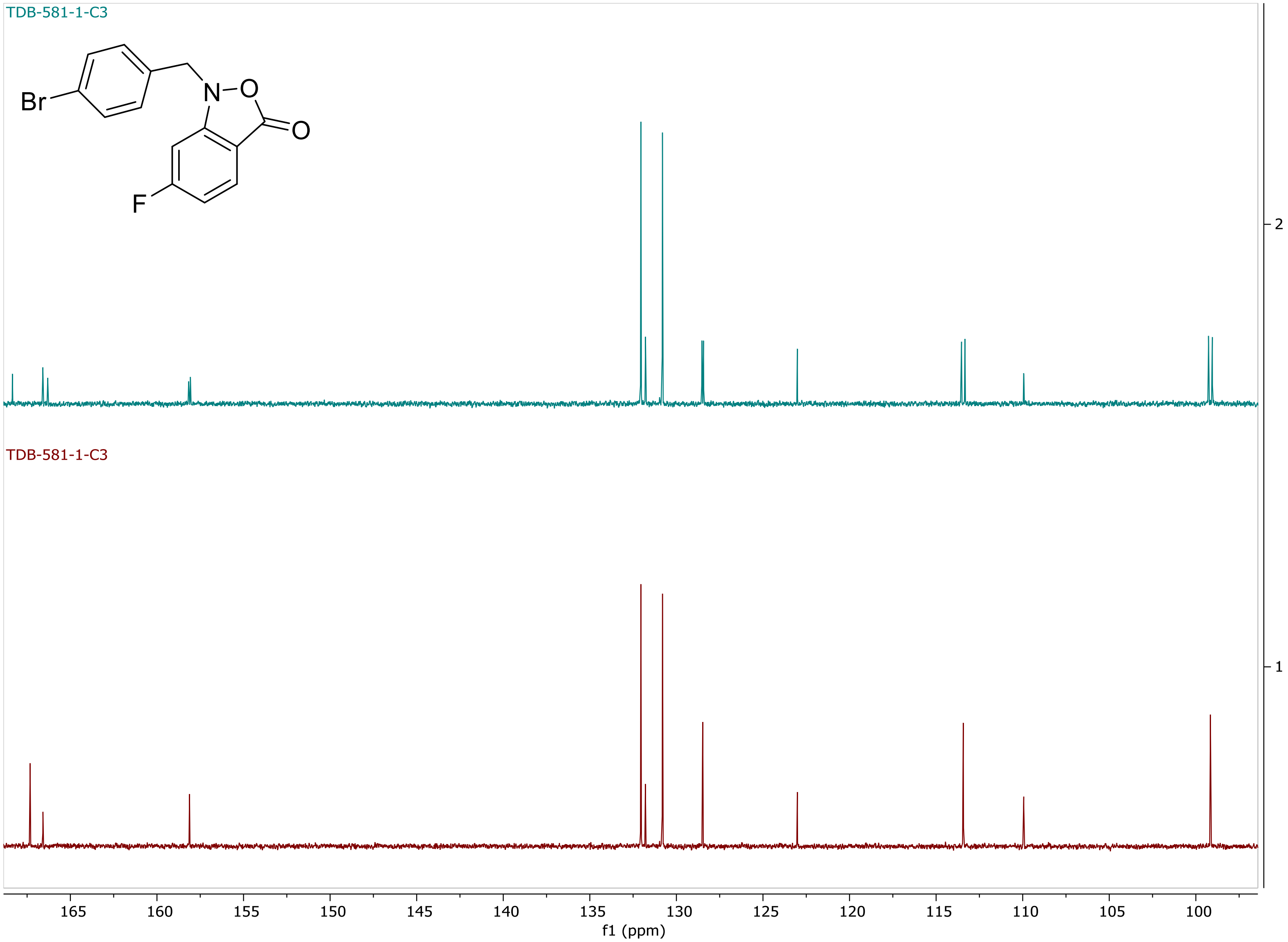

^{19}F NMR {1H} (470 MHz, CDCl$_3$) spectrum of 1-(4-bromobenzyl)-6-fluorobenzo[c]isoxazol-3(1H)-one (**13**):

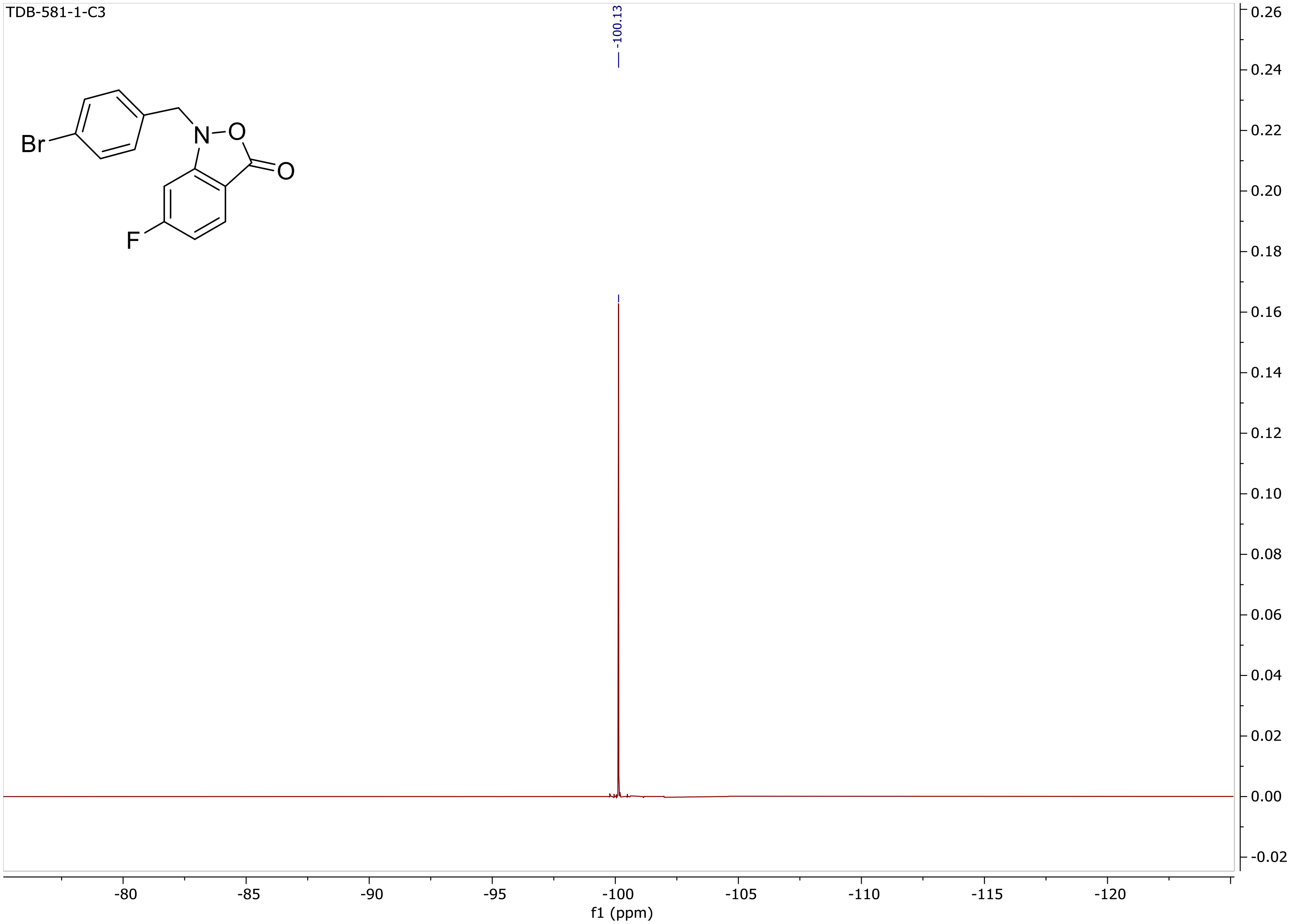

^{1}H NMR (500 MHz, CDCl₃) spectrum of 1-allyl-6-methoxybenzo[c]isoxazol-3(1H)-one (**14**):

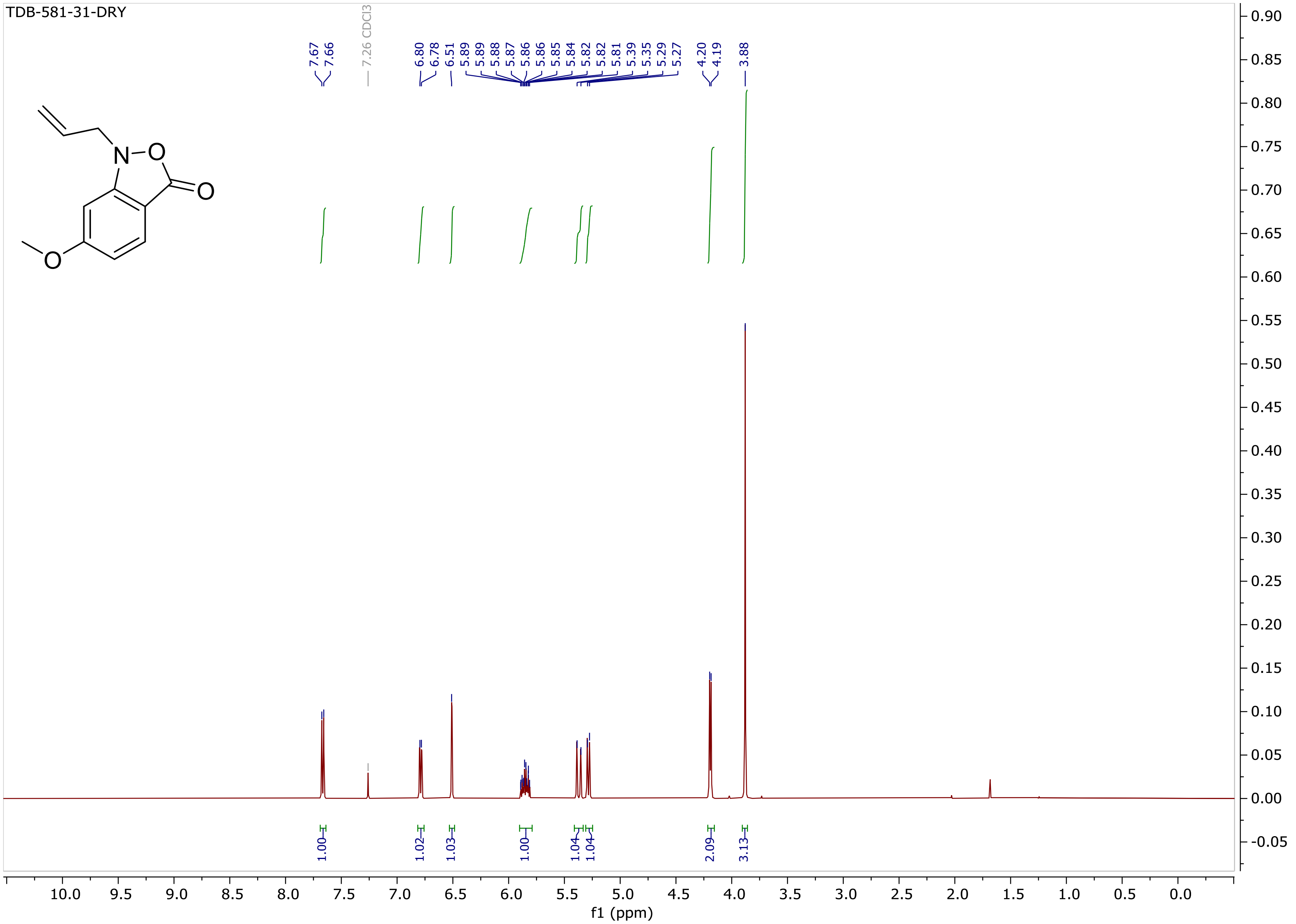

^{13}C NMR (126 MHz, CDCl$_3$) spectrum of 1-allyl-6-methoxybenzo[c]isoxazol-3(1H)-one (**14**):

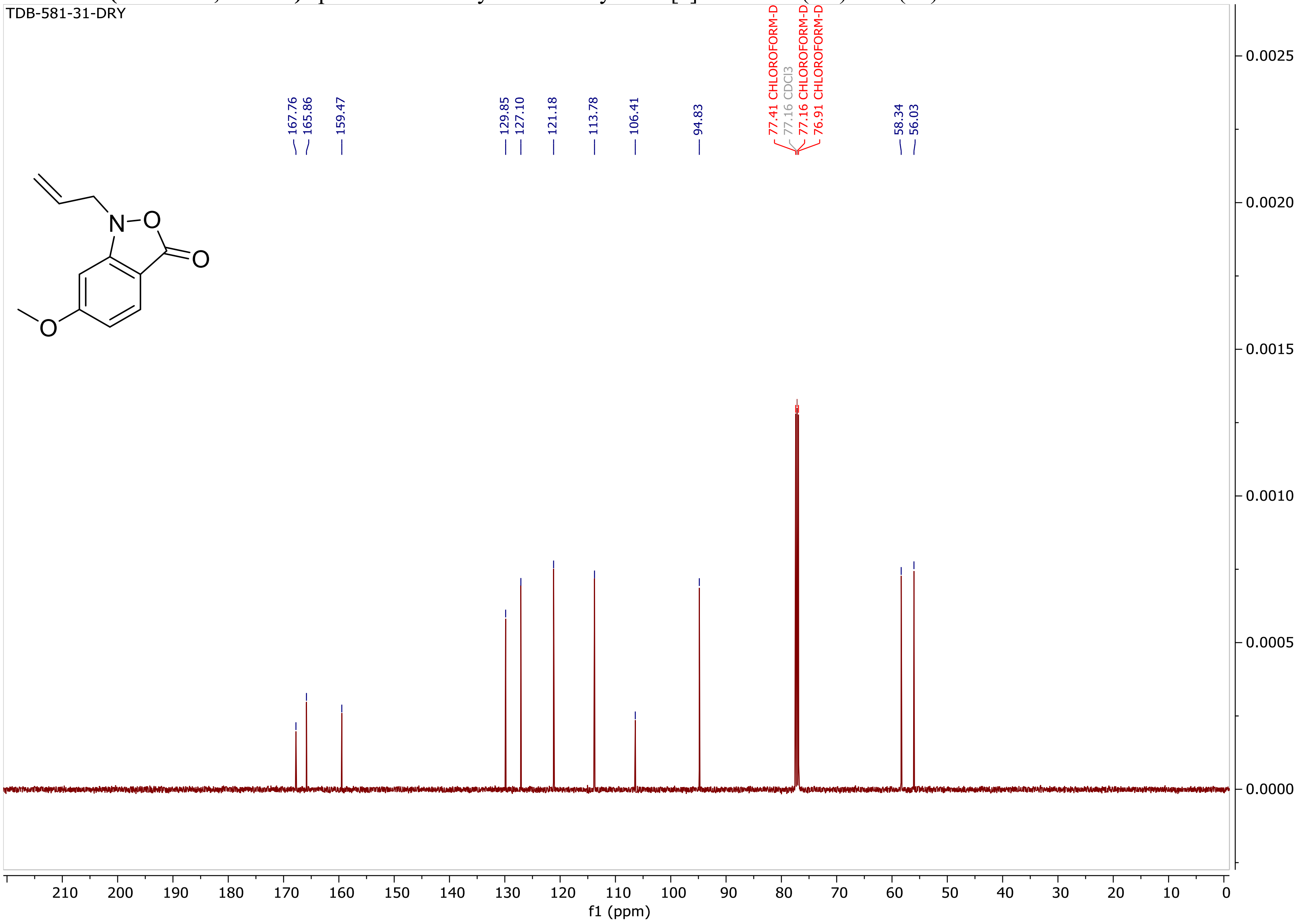

^{1}H NMR (500 MHz, CDCl3) spectrum of 1-benzyl-6-bromobenzo[c]isoxazol-3(*1H*)-one **(15):**

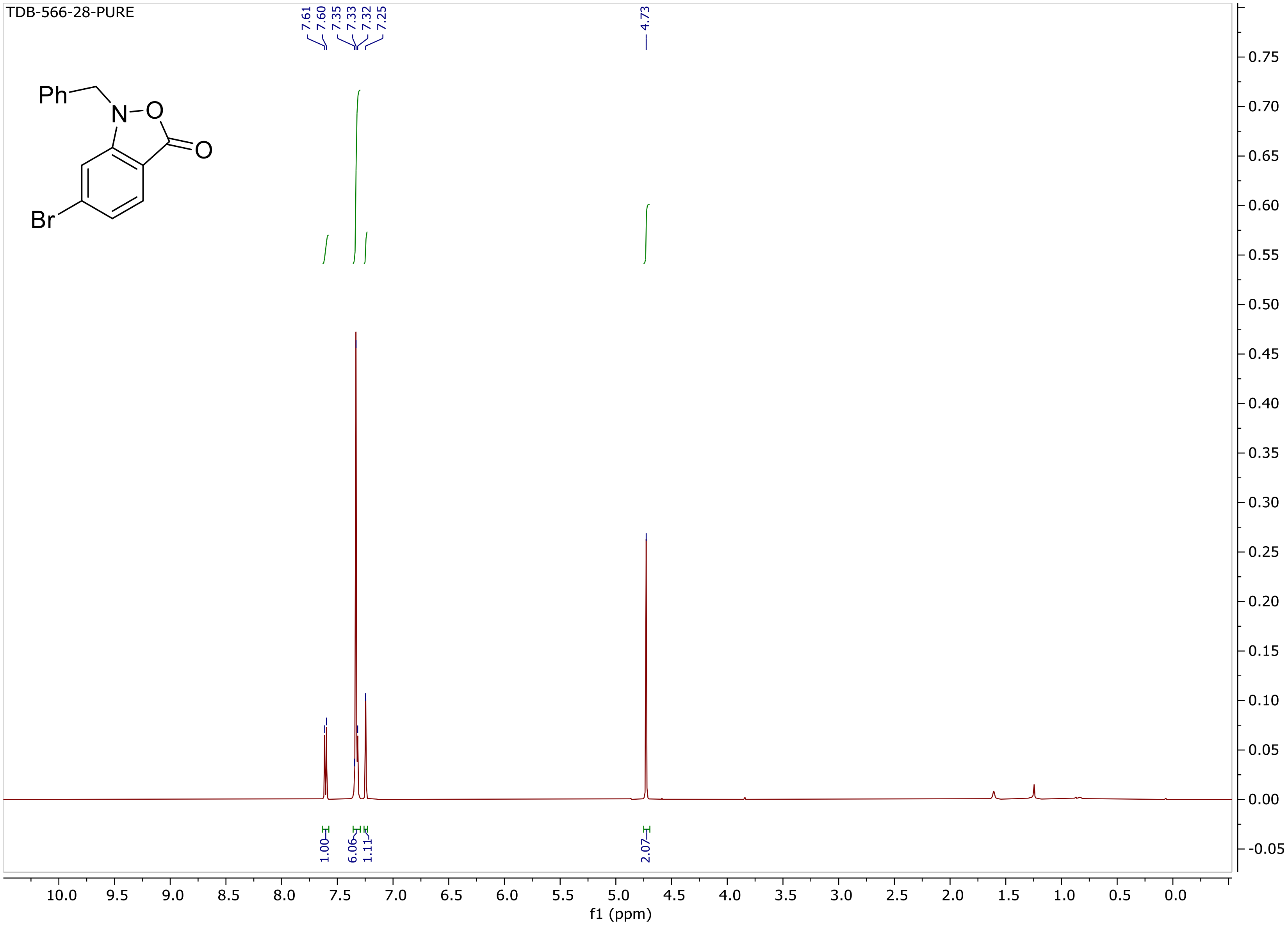

^{13}C NMR (126 MHz, CDCl$_3$) spectrum of 1-benzyl-6-bromobenzo[c]isoxazol-3(*1H*)-one **(15):**

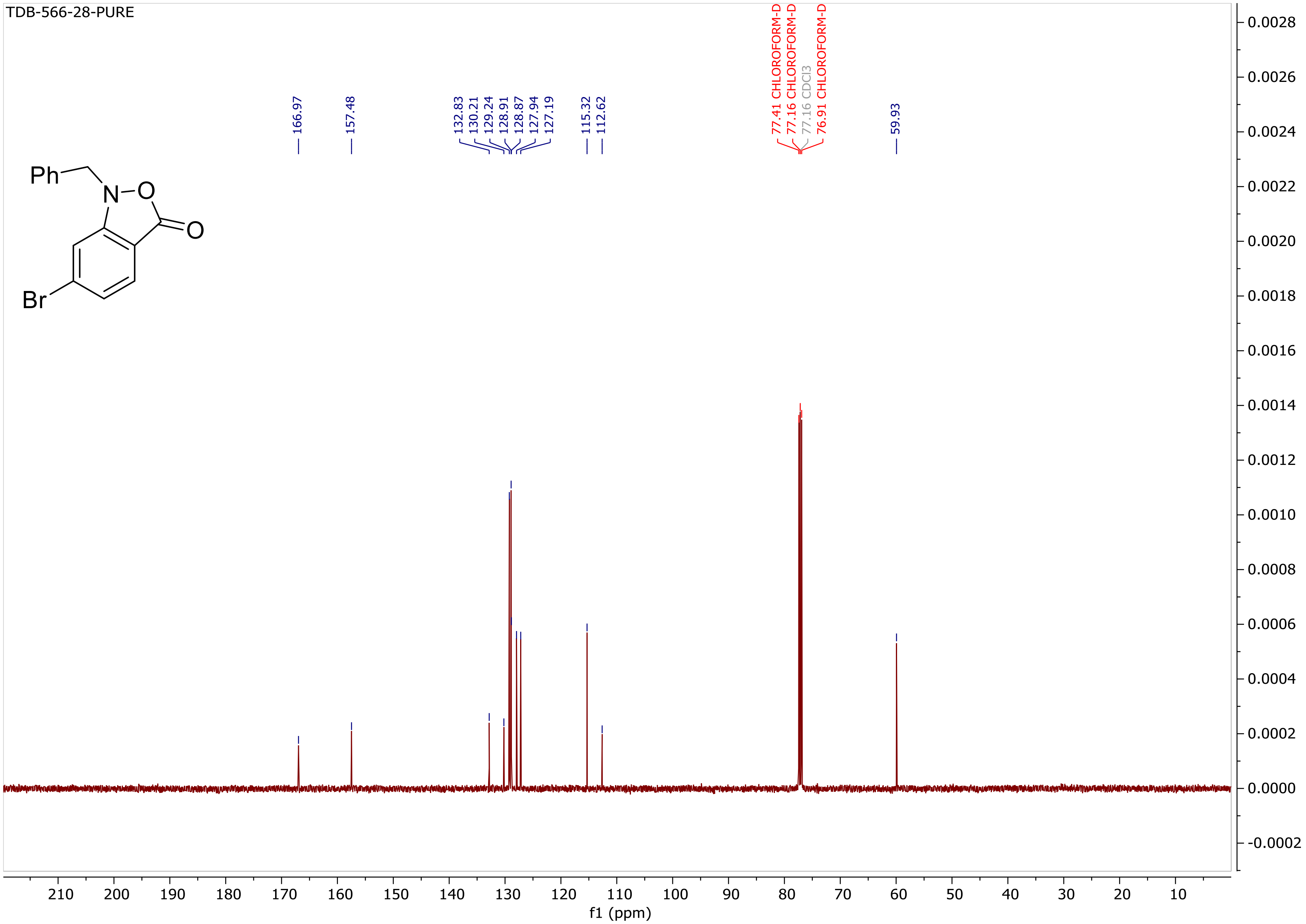

^{1}H NMR (500 MHz, CDCl3) spectrum of 1-(4-bromobenzyl)-6-(trifluoromethyl)-1,3-dihydrobenzo[*c*]isoxazole (**16**):

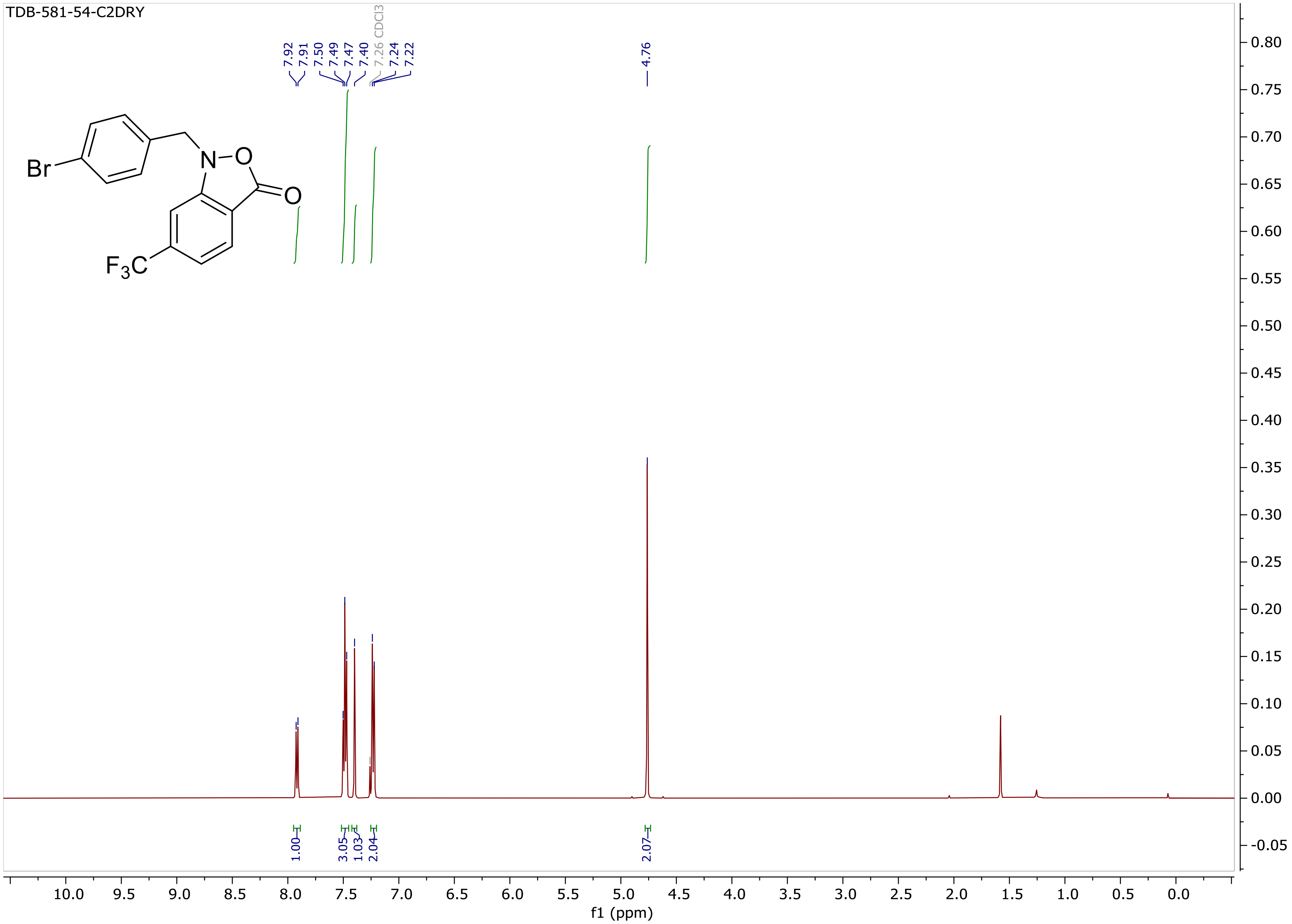

^{13}C NMR (126 MHz, $CDCl_3$) spectrum of 1-(4-bromobenzyl)-6-(trifluoromethyl)benzo[c]isoxazol-3(*1H*)-on (**16**):

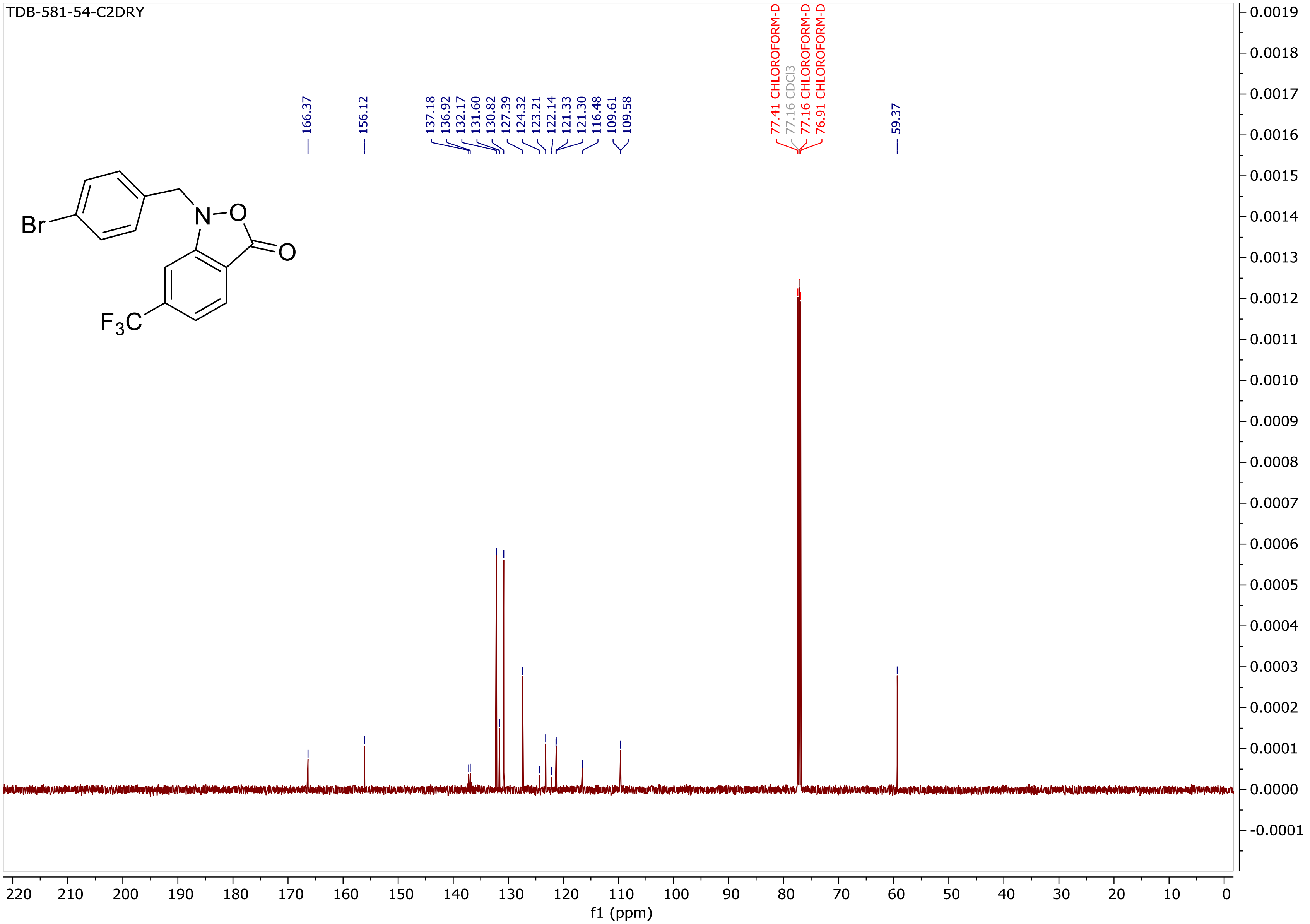

^{13}C NMR {^{19}F} (126 MHz, CDCl$_3$) spectrum of 1-(4-bromobenzyl)-6-(trifluoromethyl)benzo[c]isoxazol-3(*1H*)-one (**16**):

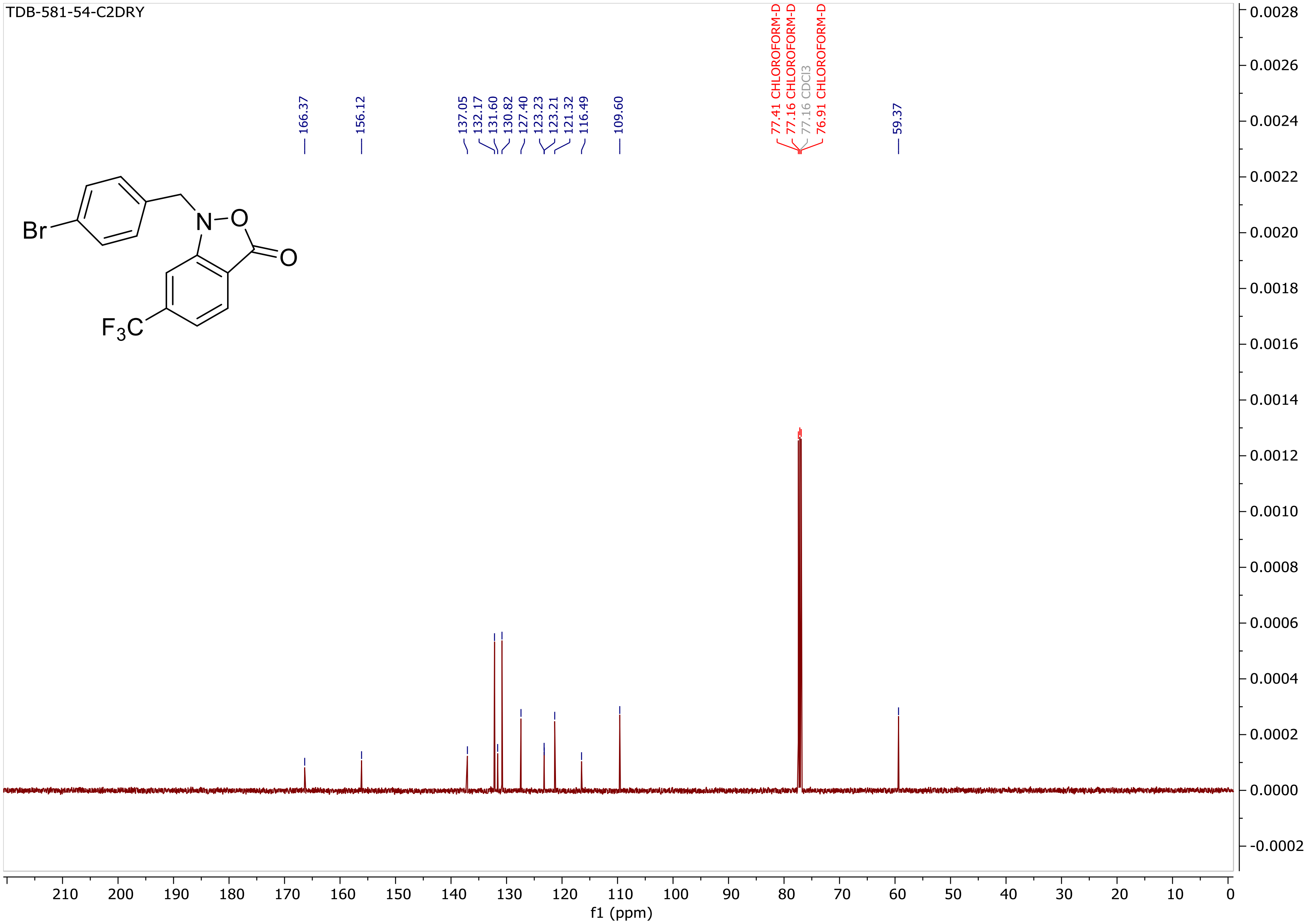

Expanded region of stacked (top) **^{13}C NMR (126 MHz, CDCl$_3$)** and (bottom) **^{13}C NMR {^{19}F} (126 MHz, CDCl$_3$)** spectrum of 1-(4-bromobenzyl)-6-(trifluoromethyl)benzo[c]isoxazol-3(*1H*)-one (**16**):

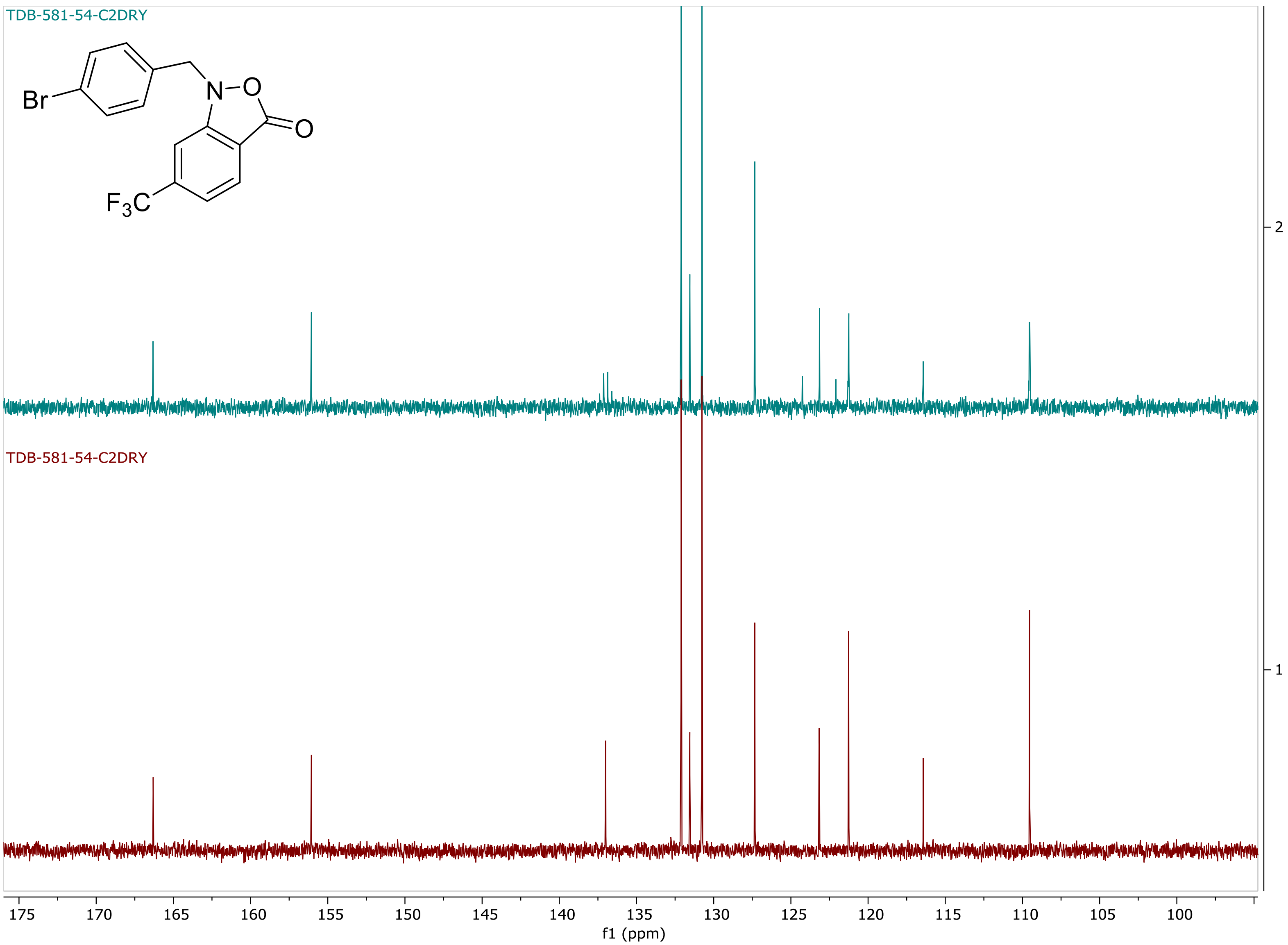

^{19}F NMR {1H} (470 MHz, CDCl$_3$) spectrum of 1-(4-bromobenzyl)-6-(trifluoromethyl)benzo[c]isoxazol-3(*1H*)-one (**16**):

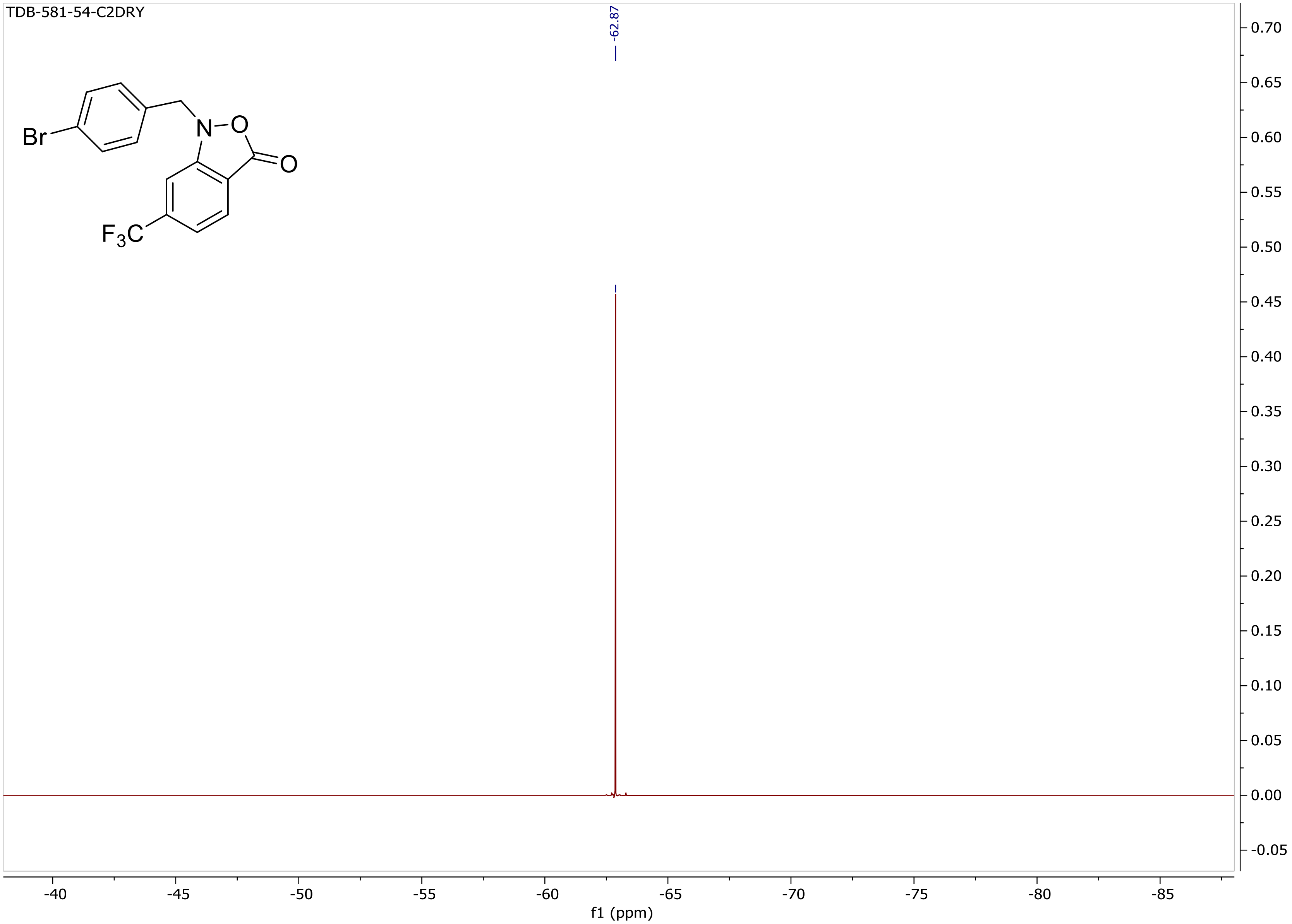

^{1}H NMR (500 MHz, CDCl$_3$) spectrum of 5-fluoro-1-hexylbenzo[*c*]isoxazol-3(*1H*)-one (**17**):

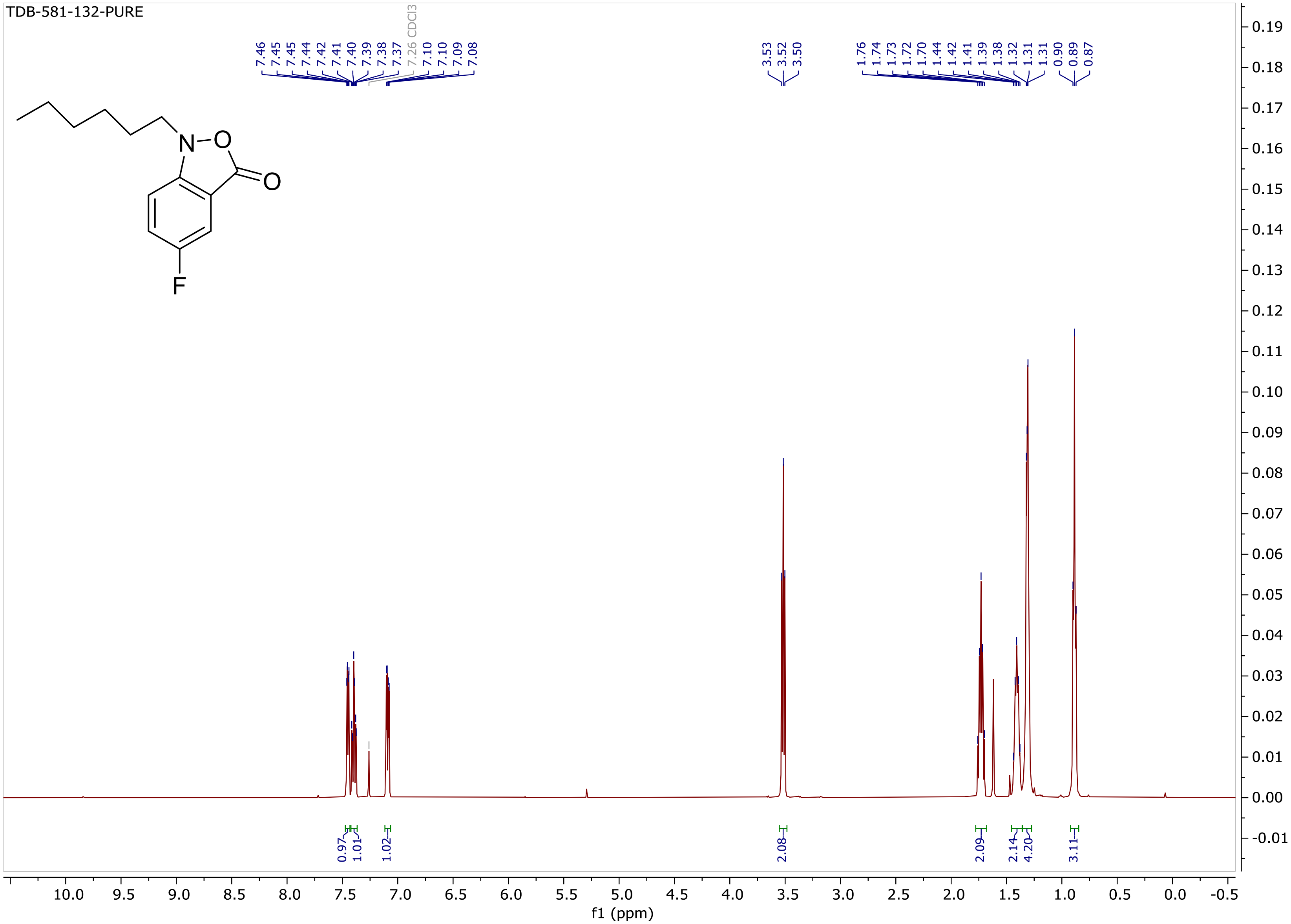

^{13}C NMR (126 MHz, CDCl$_3$) spectrum of 5-fluoro-1-hexylbenzo[*c*]isoxazol-3(*1H*)-one (**17**):

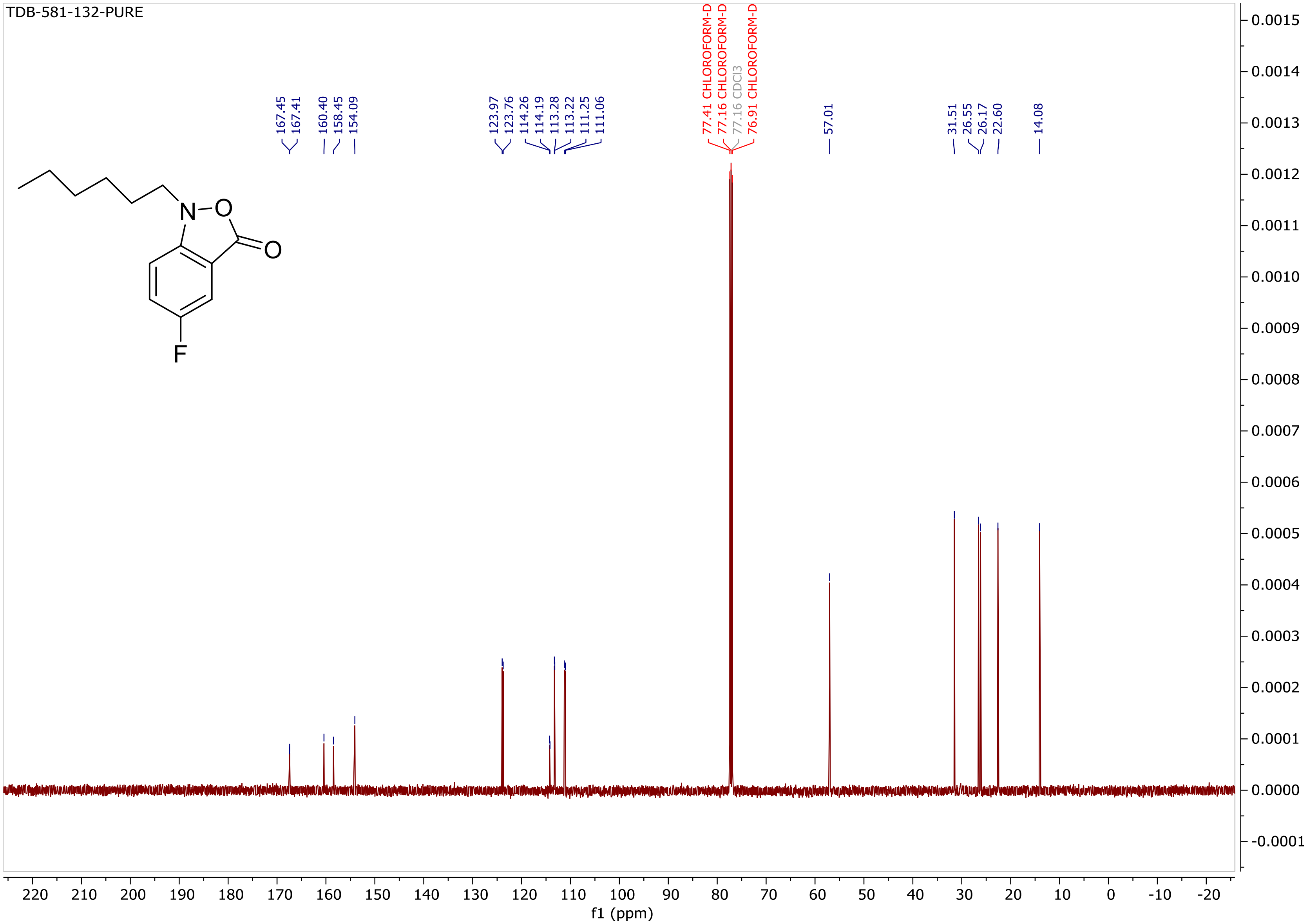

^{13}C NMR {^{19}F} (126 MHz, CDCl$_3$) spectrum of 5-fluoro-1-hexylbenzo[*c*]isoxazol-3(*1H*)-one (**17**):

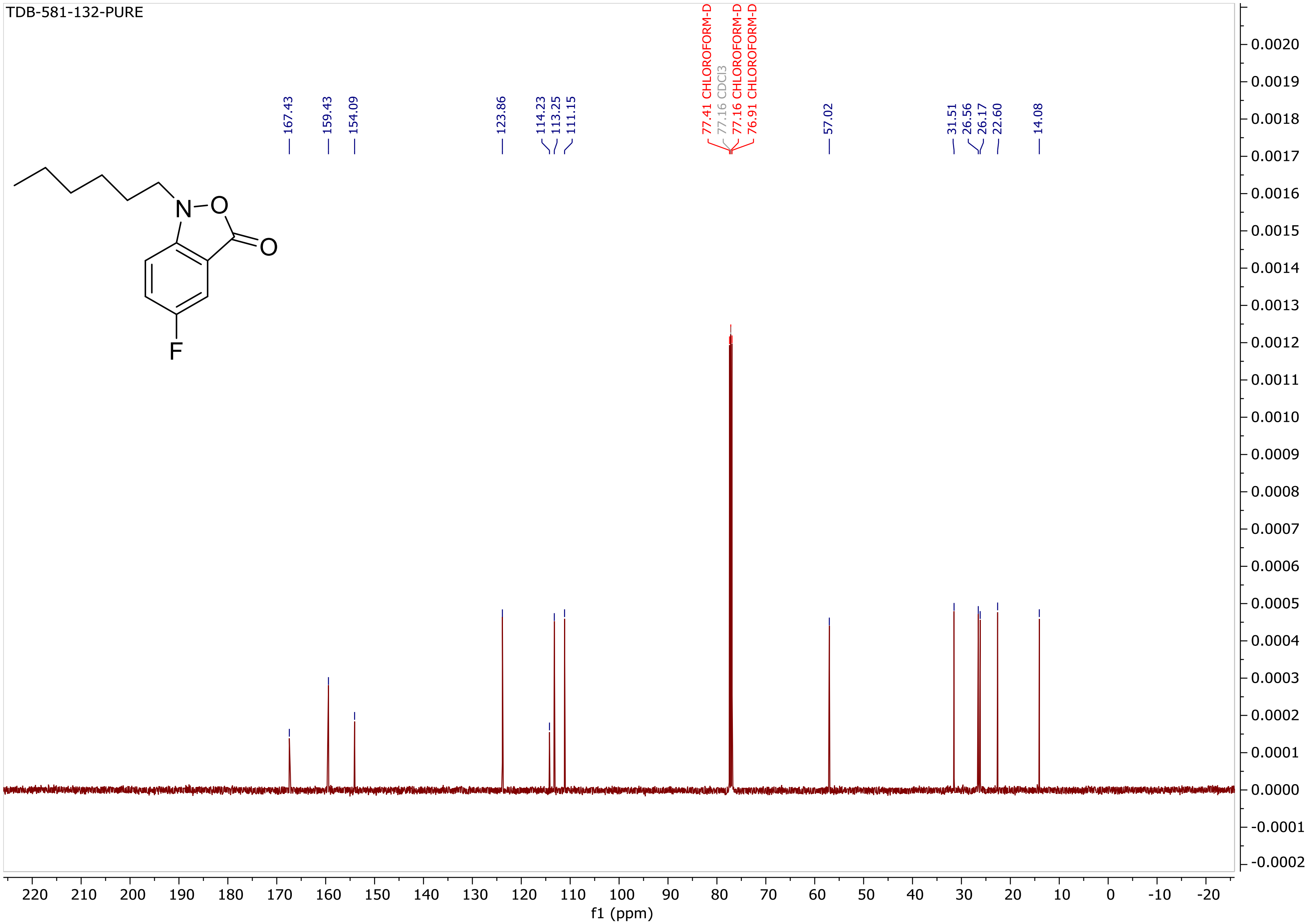

Expanded region of stacked (top) **^{13}C NMR (126 MHz, CDCl$_3$)** and (bottom) **^{13}C NMR {^{19}F} (126 MHz, CDCl$_3$)** spectrum of 5-fluoro-1-hexylbenzo[*c*]isoxazol-3(*1H*)-one (**17**):

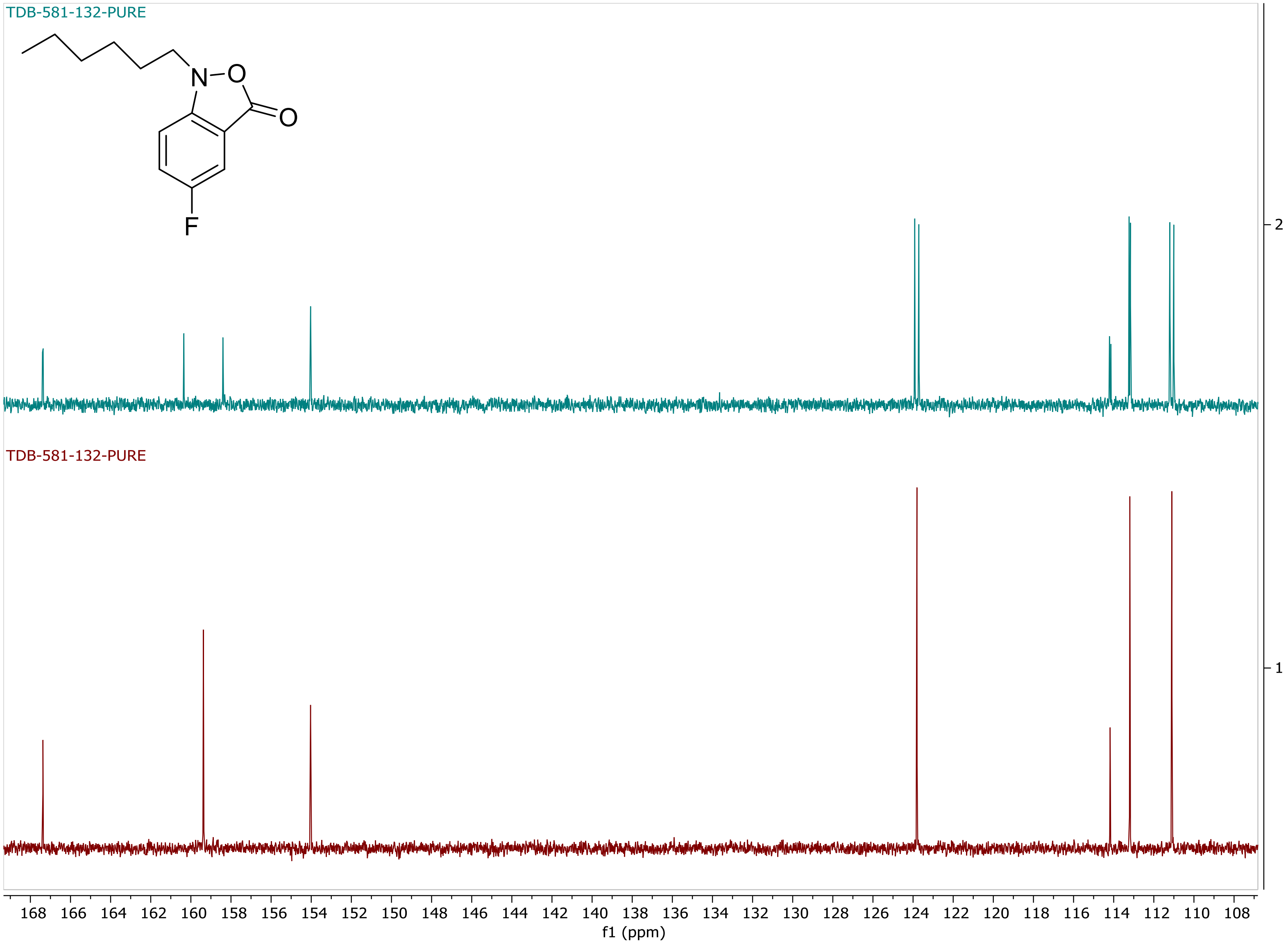

^{19}F NMR {1H} (470 MHz, CDCl$_3$) spectrum of 5-fluoro-1-hexylbenzo[*c*]isoxazol-3(*1H*)-one (**17**):

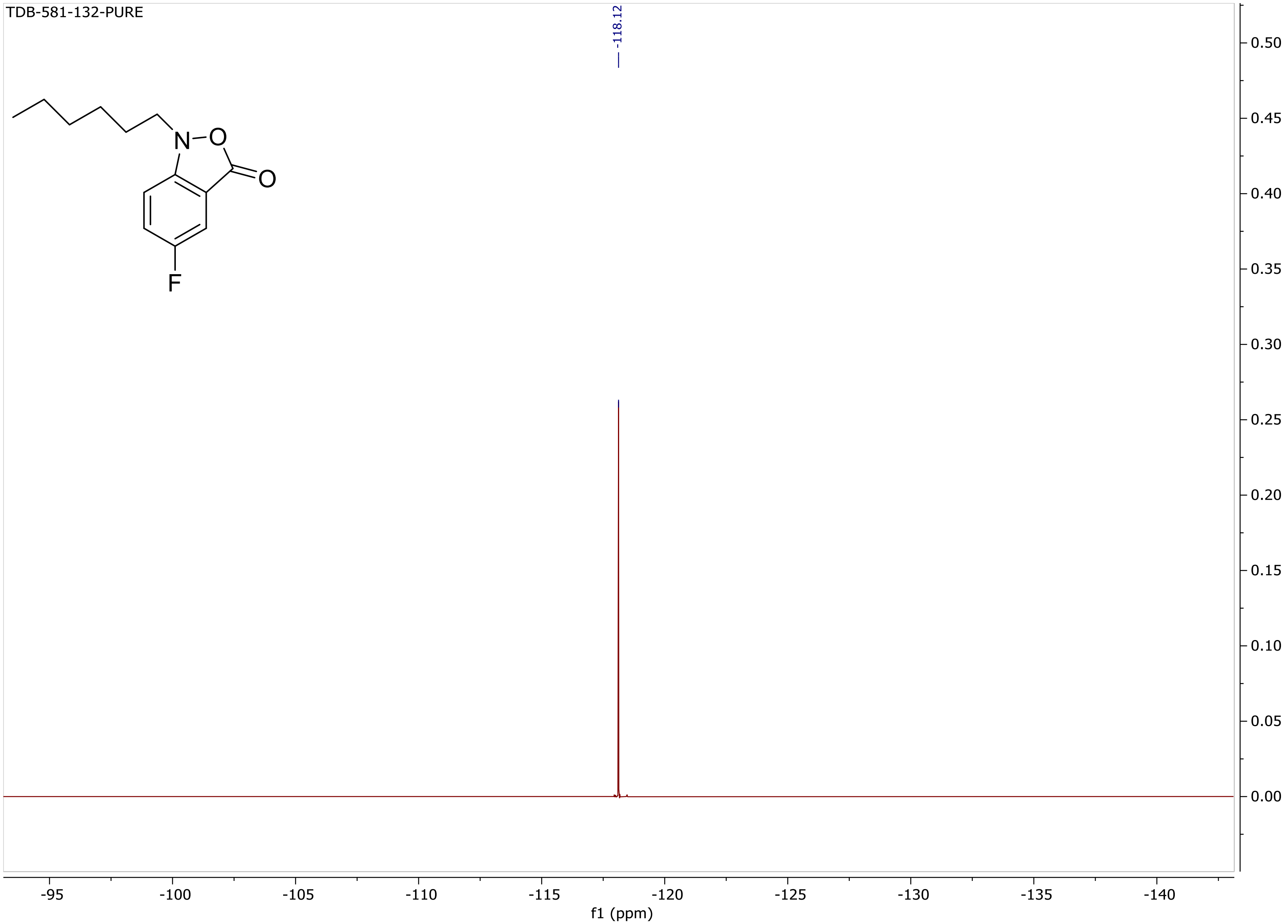

^{1}H NMR (500 MHz, CDCl$_3$) spectrum of 1-(benzyloxy)indolin-2-one **(18):**

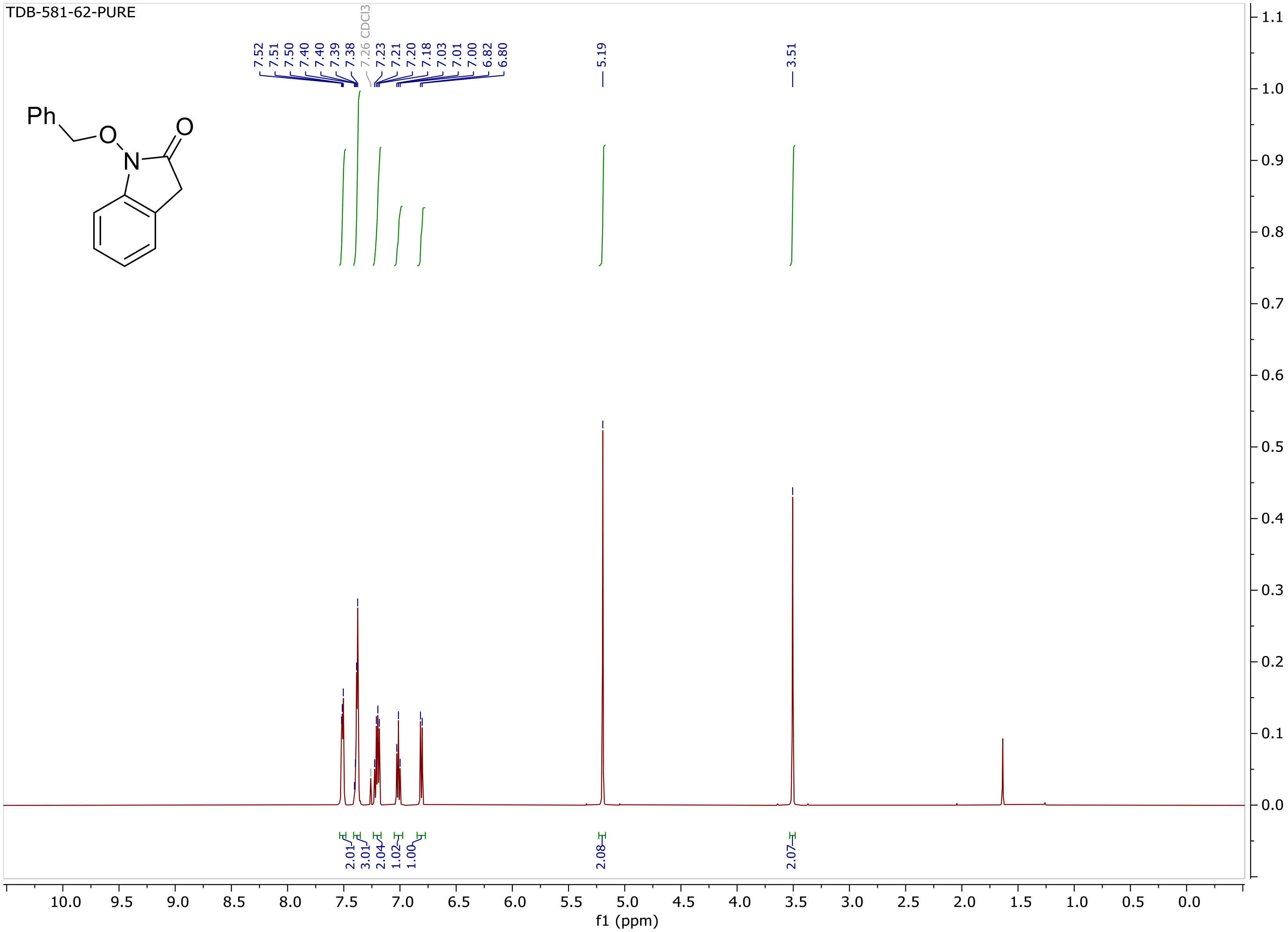

^{13}C NMR (126 MHz, CDCl$_3$) spectrum of 1-(benzyloxy)indolin-2-one **(18):**

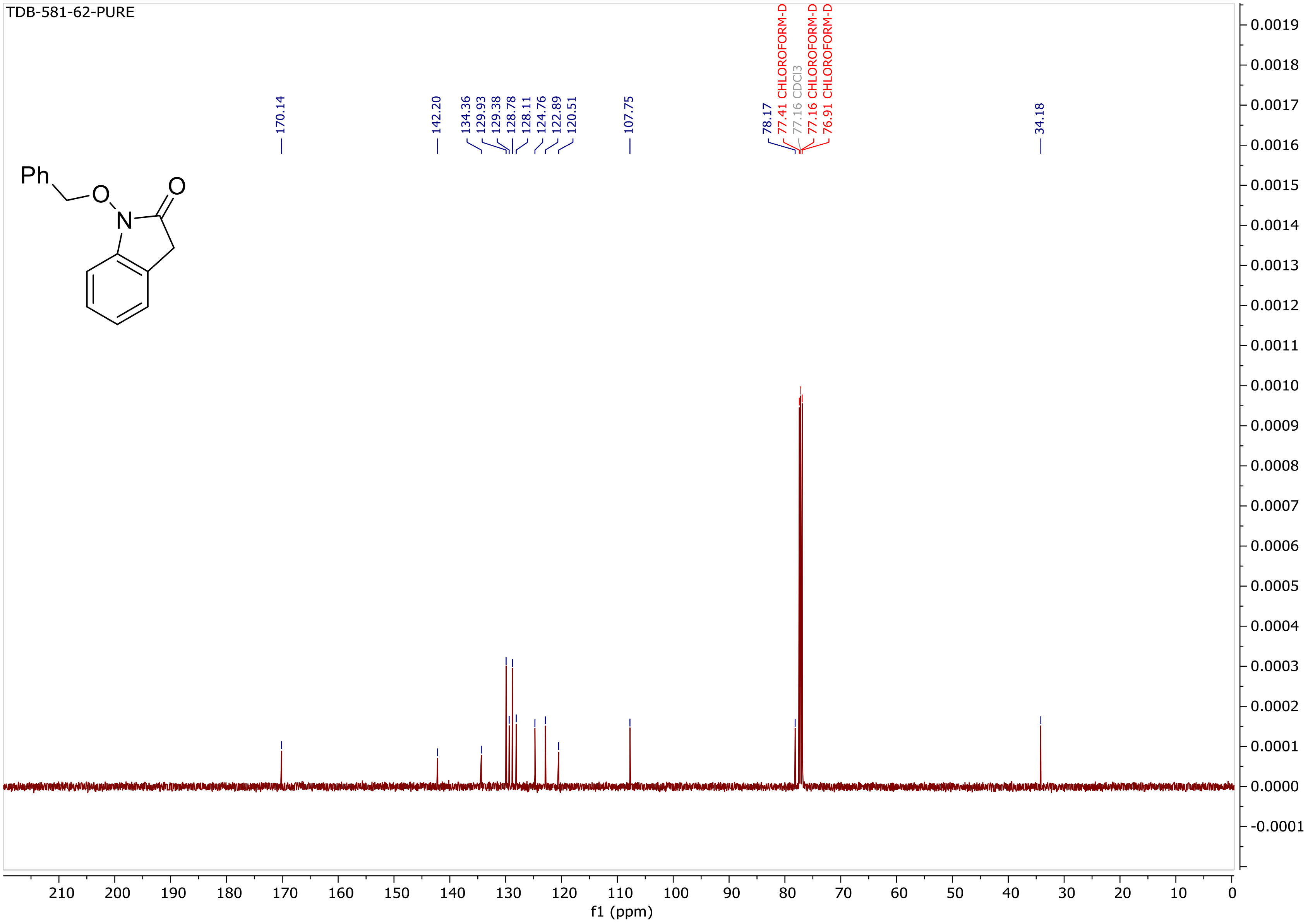

HSQC NMR (500 MHz, CDCl₃) spectrum of 1-(benzyloxy)indolin-2-one **(18):**

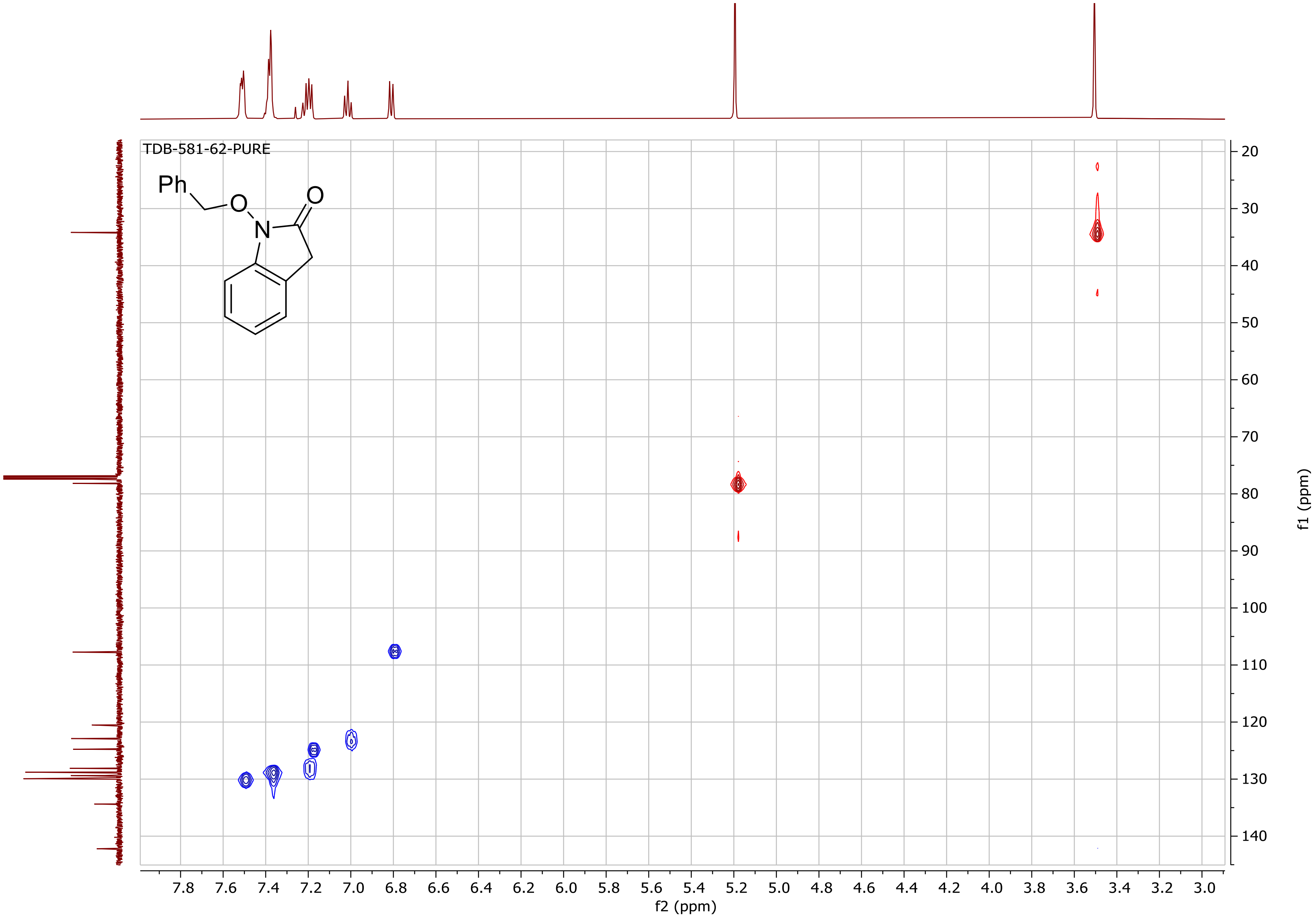

HMBC NMR (500 MHz, $CDCl_3$) spectrum of 1-(benzyloxy)indolin-2-one **(18):**

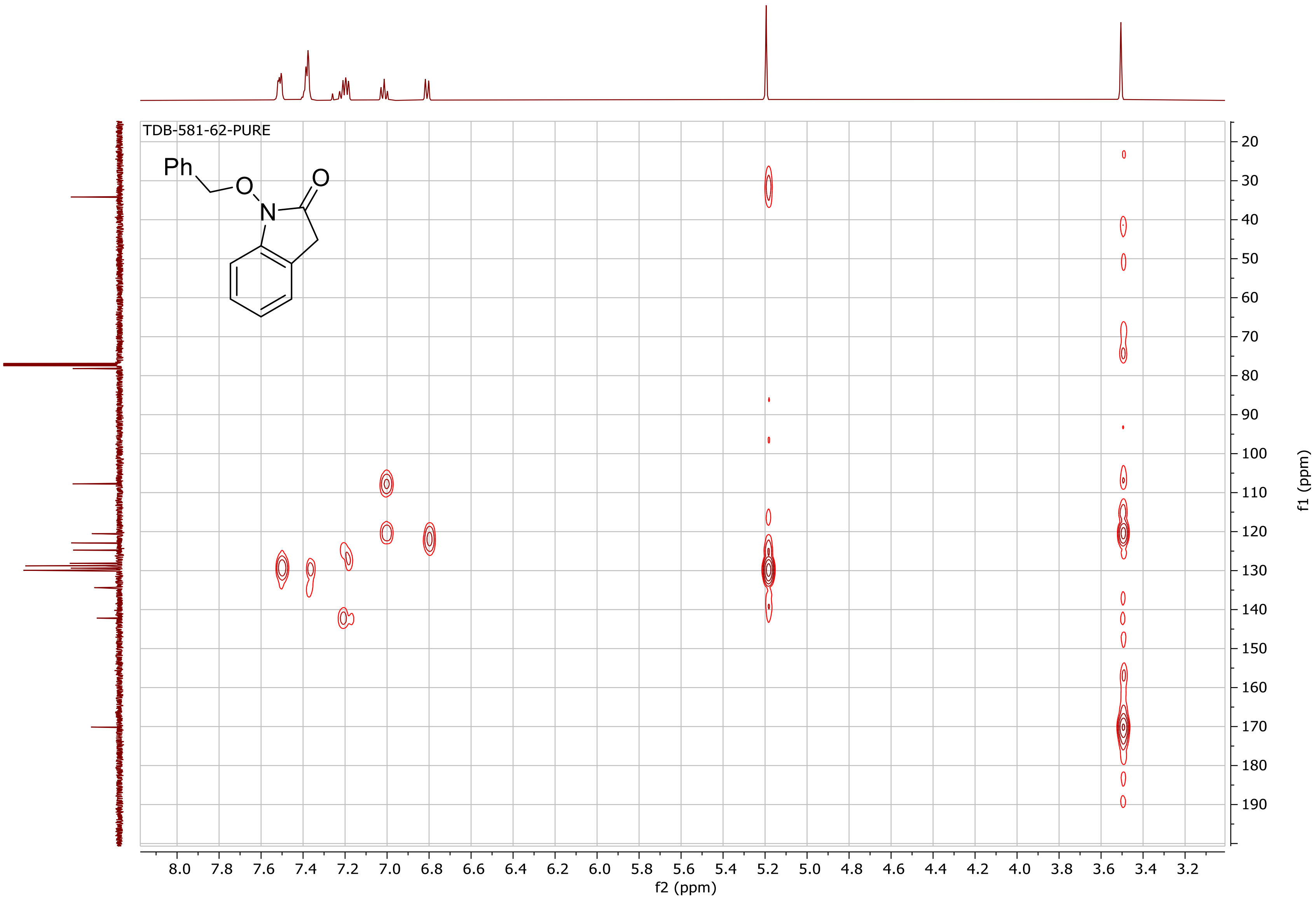

^{1}H NMR (500 MHz, CDCl$_3$) spectrum of 1-(benzyloxy)-3,4-dihydroquinolin-2(*1H*)-one (**19**):

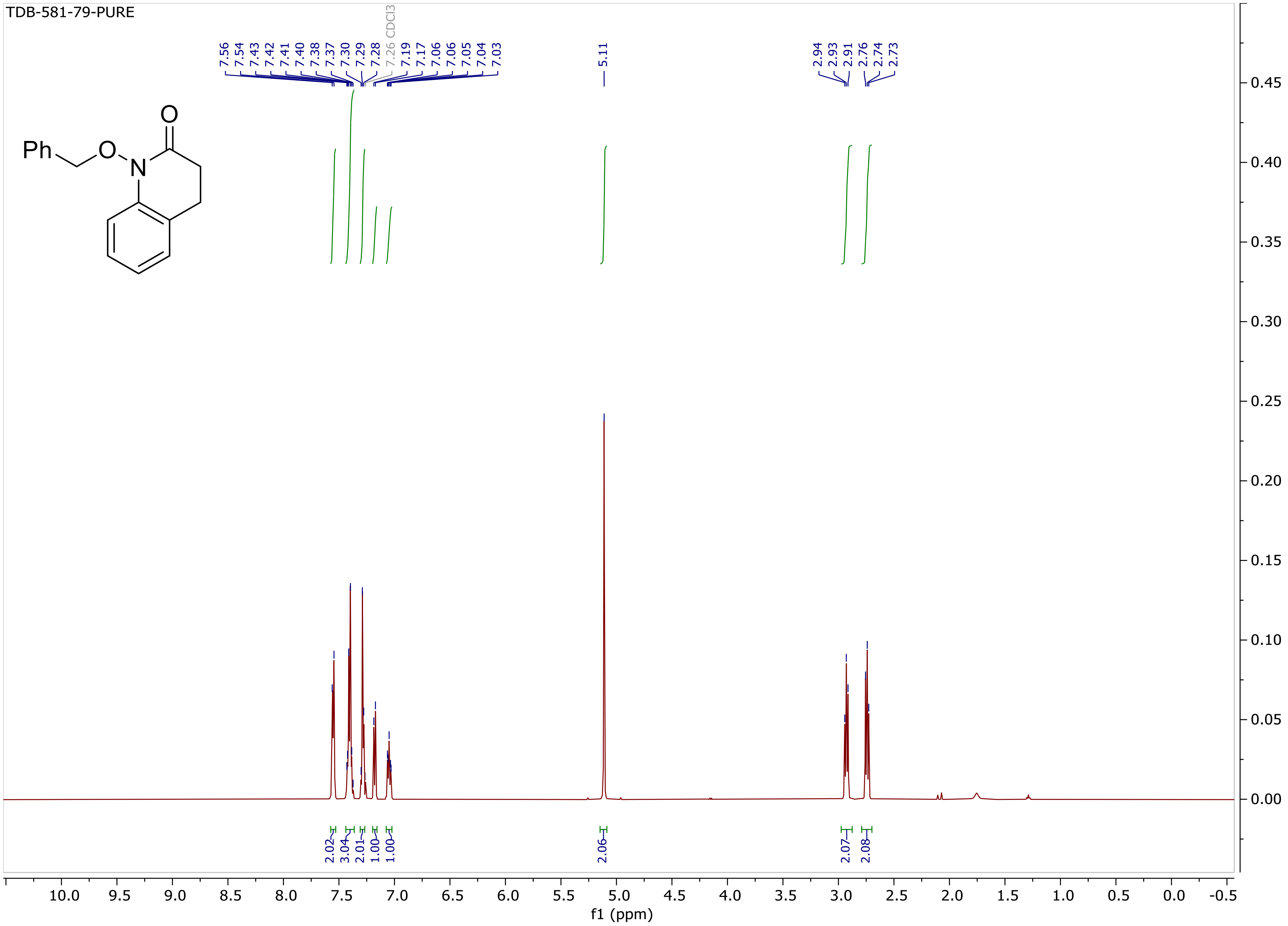

^{13}C NMR (126 MHz, CDCl$_3$) spectrum of 1-(benzyloxy)-3,4-dihydroquinolin-2(*1H*)-one (**19**):

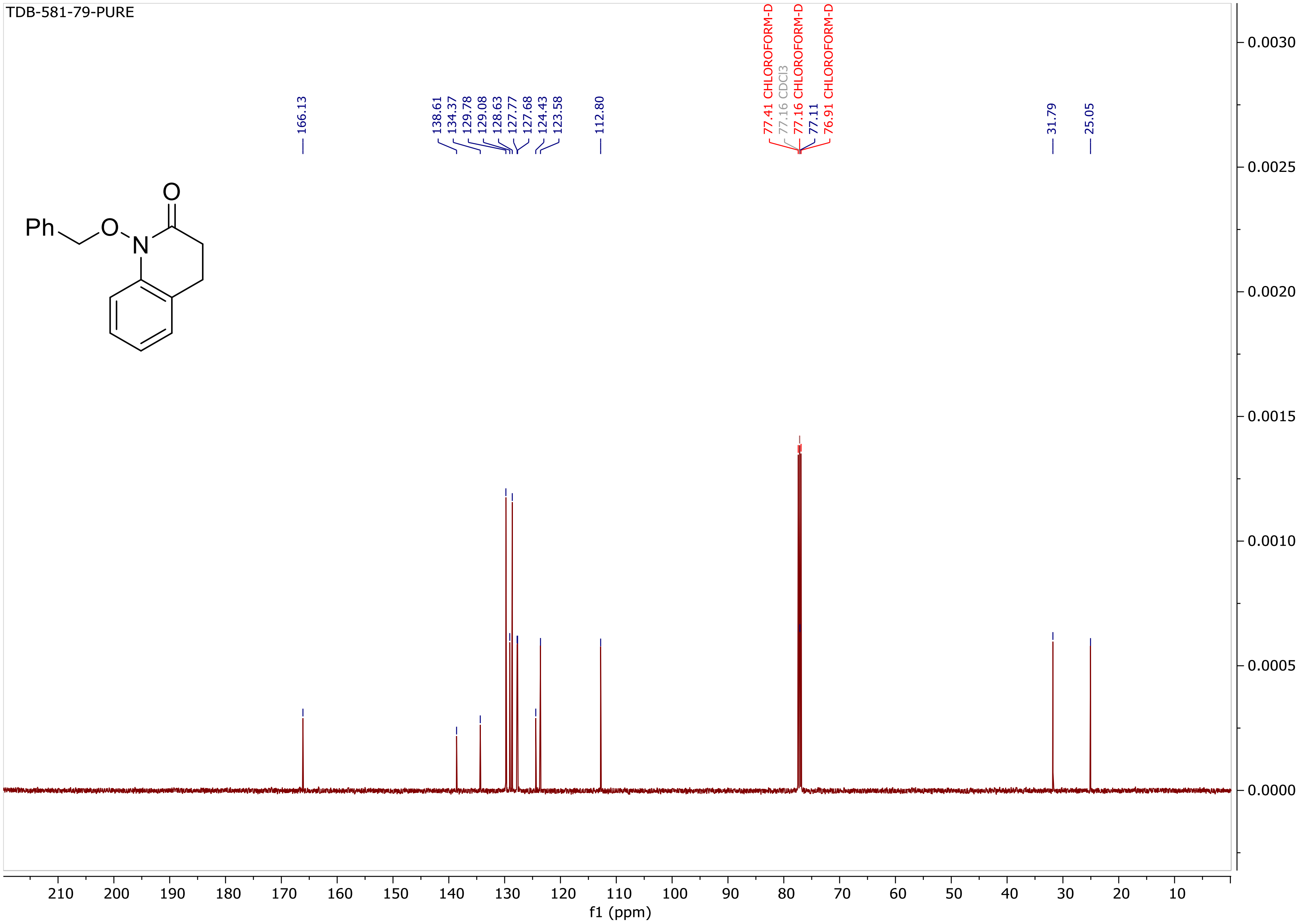

HSQC NMR (500 MHz, CDCl₃) spectrum of 1-(benzyloxy)-3,4-dihydroquinolin-2(*1H*)-one (**19**):

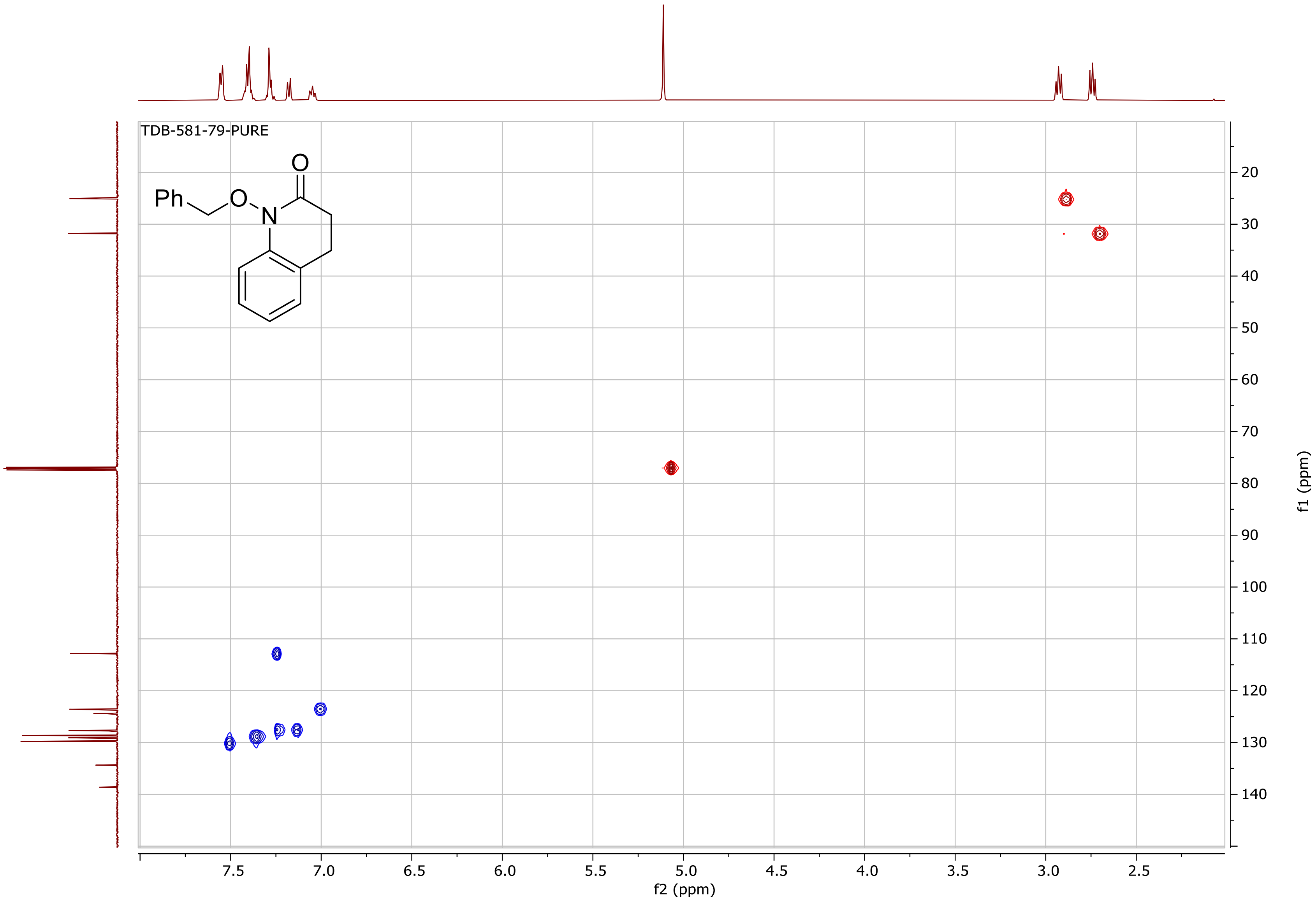

HMBC NMR (500 MHz, CDCl3) spectrum of 1-(benzyloxy)-3,4-dihydroquinolin-2(*1H*)-one (**19**):

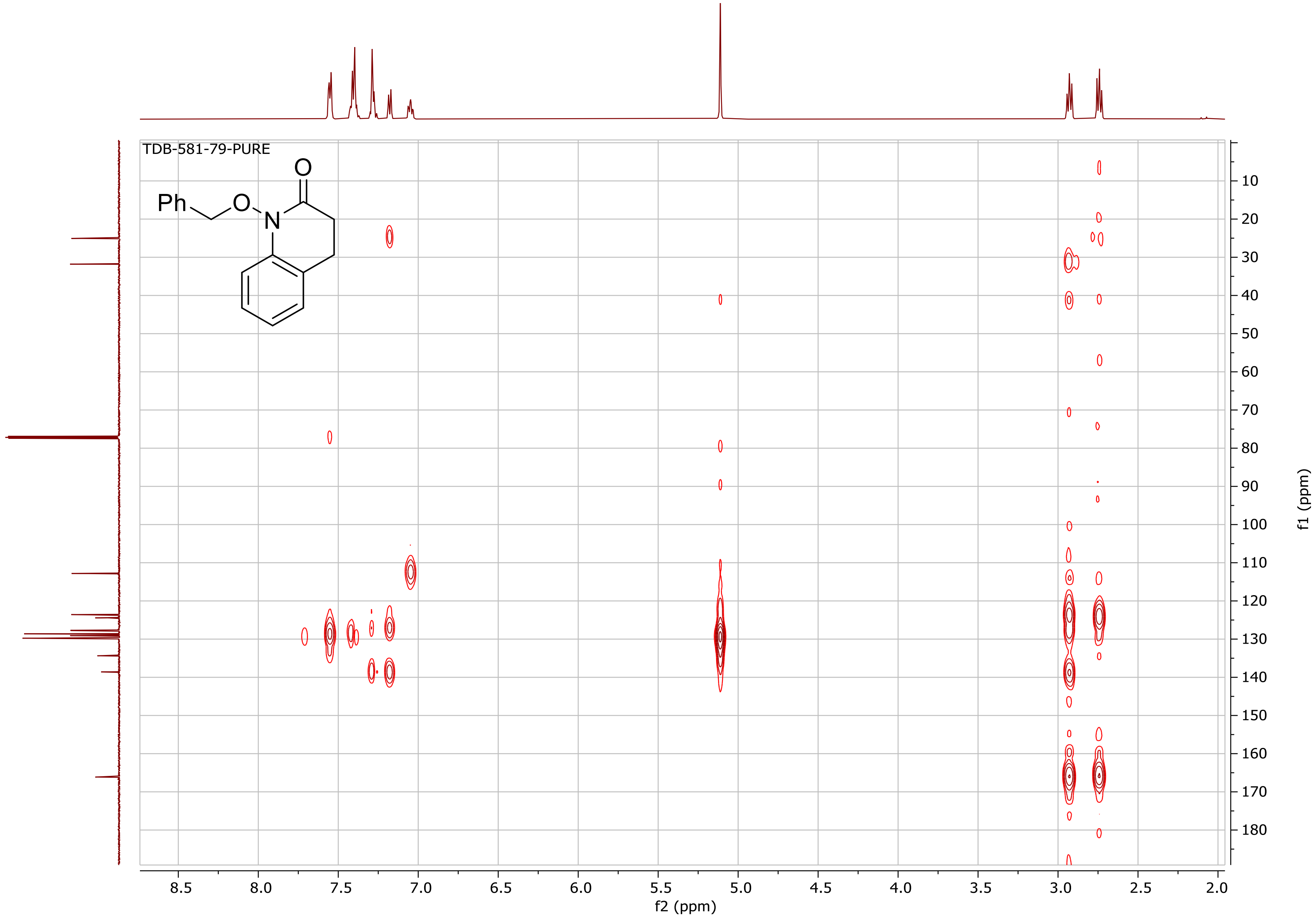

^{1}H NMR (500 MHz, CDCl3) spectrum of 1-benzyl-1,3-dihydrobenzo[c]isoxazole (**24**):

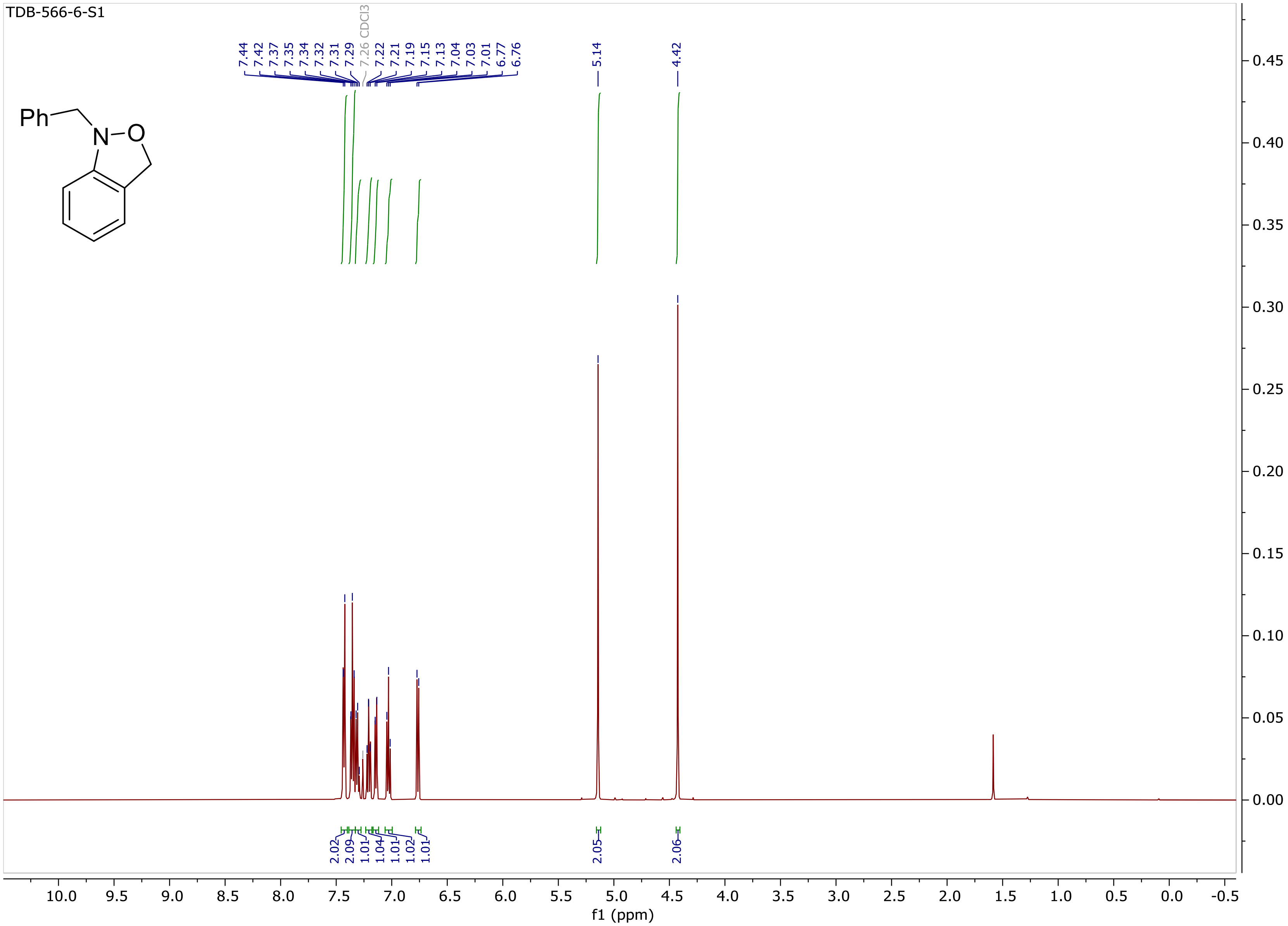

^{13}C NMR (126 MHz, $CDCl_3$) spectrum of 1-benzyl-1,3-dihydrobenzo[c]isoxazole (**24**):

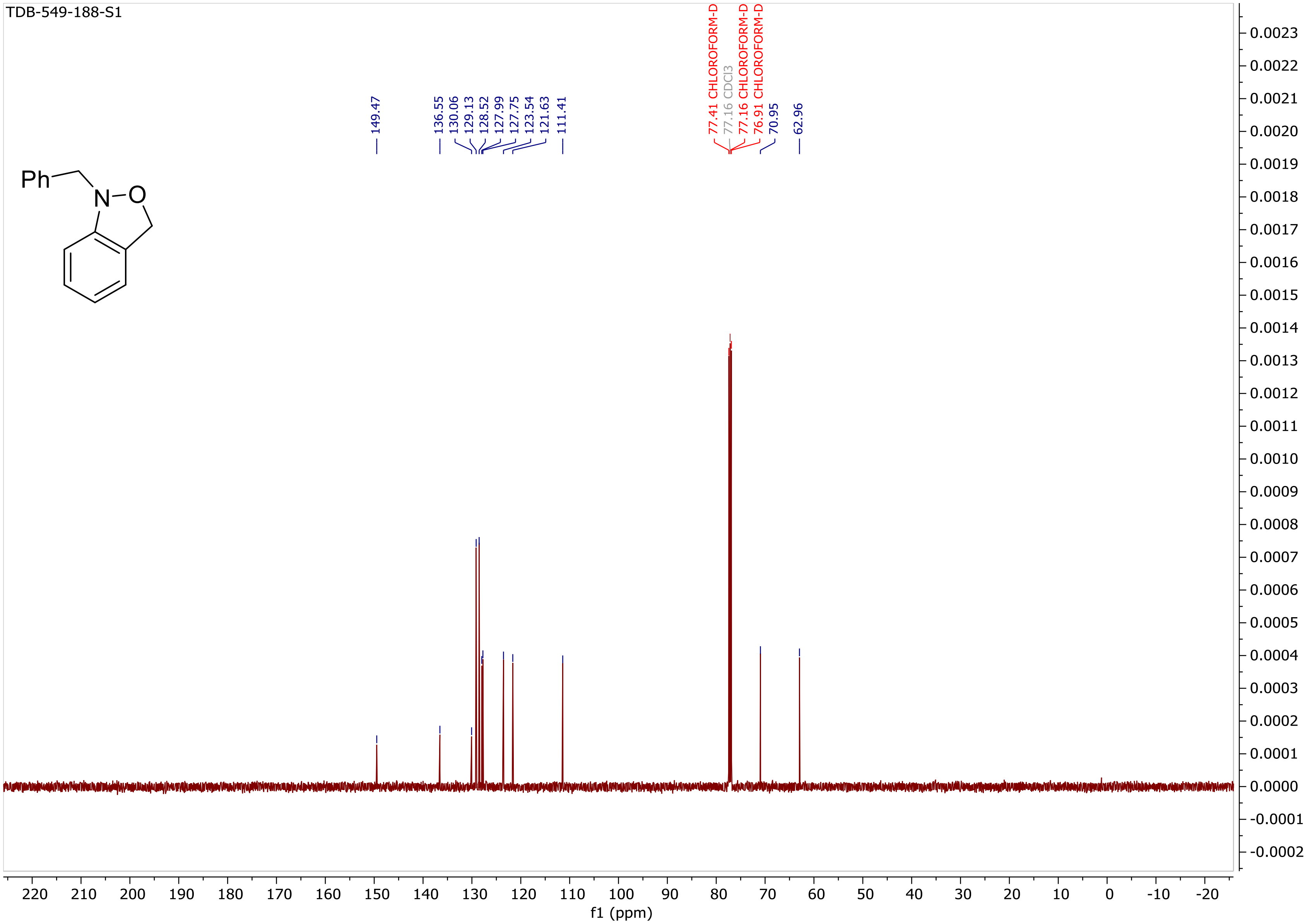

^{1}H NMR (500 MHz, CDCl₃) spectrum of 1-allyl-1,3-dihydrobenzo[*c*]isoxazole (**25**):

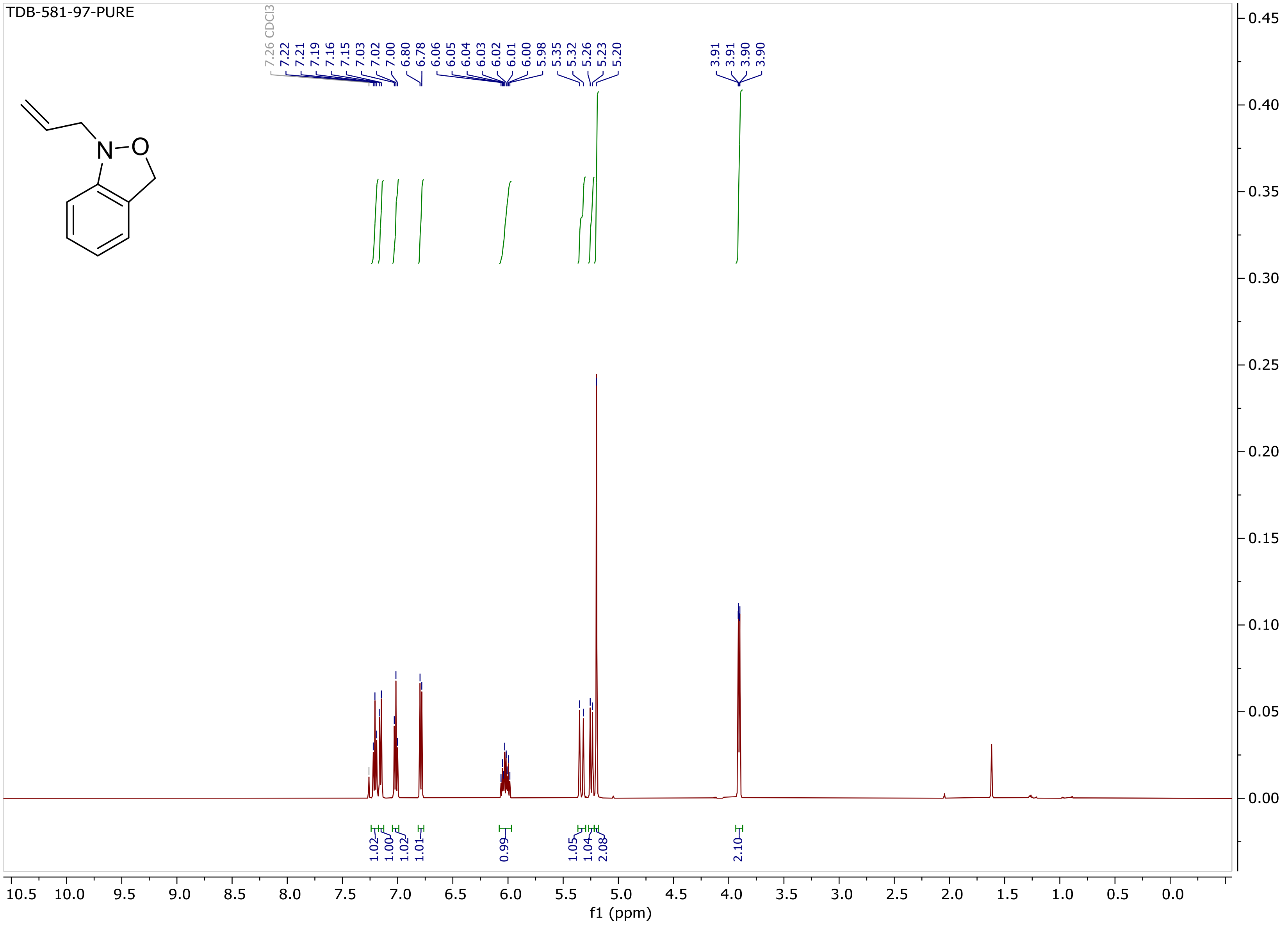

^{13}C NMR (126 MHz, CDCl$_3$) spectrum of 1-allyl-1,3-dihydrobenzo[*c*]isoxazole (**25**):

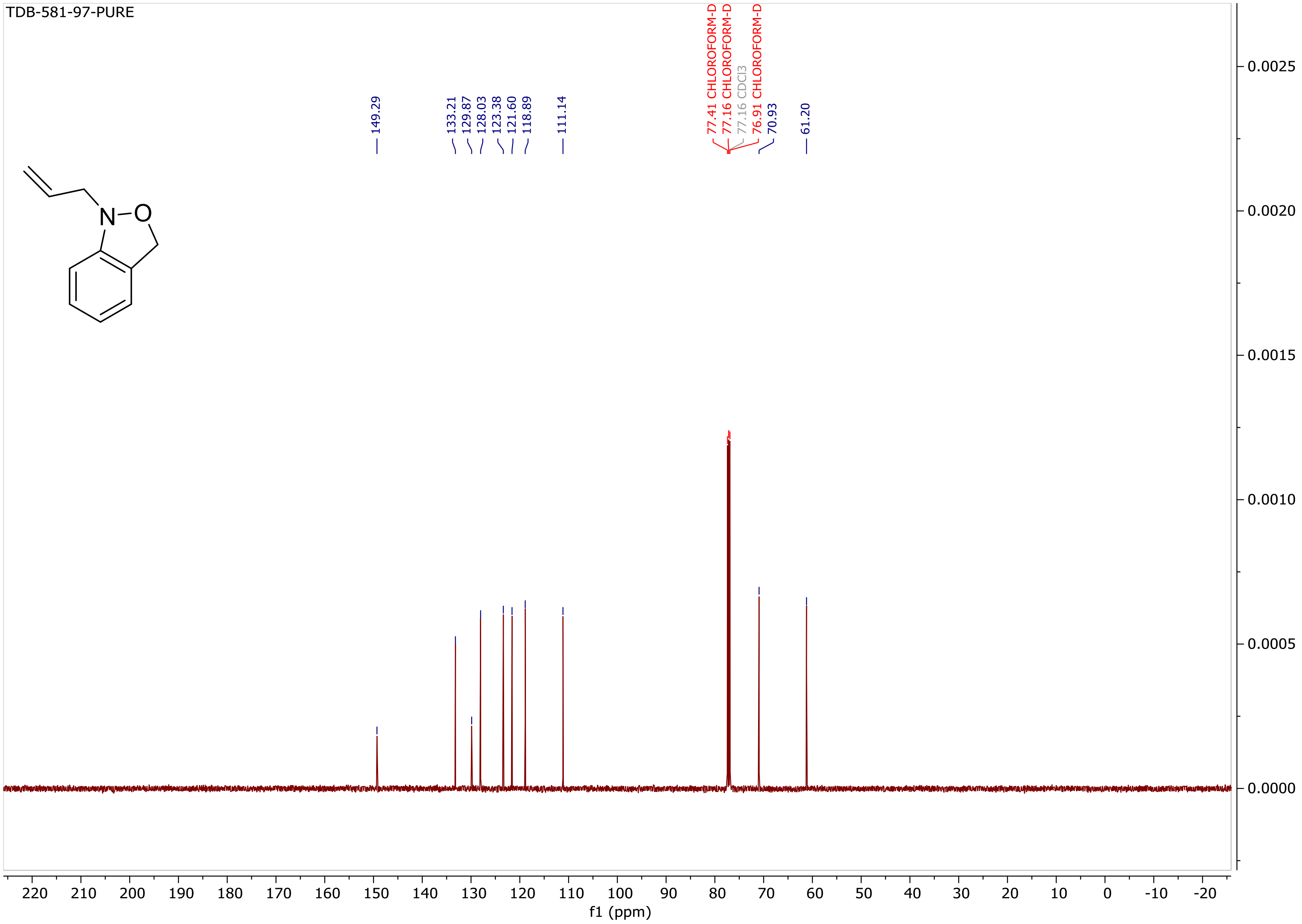

^{1}H NMR (500 MHz, CDCl$_3$) spectrum of 1-benzyl-5-methyl-1,3-dihydrobenzo[c]isoxazole (**26**):

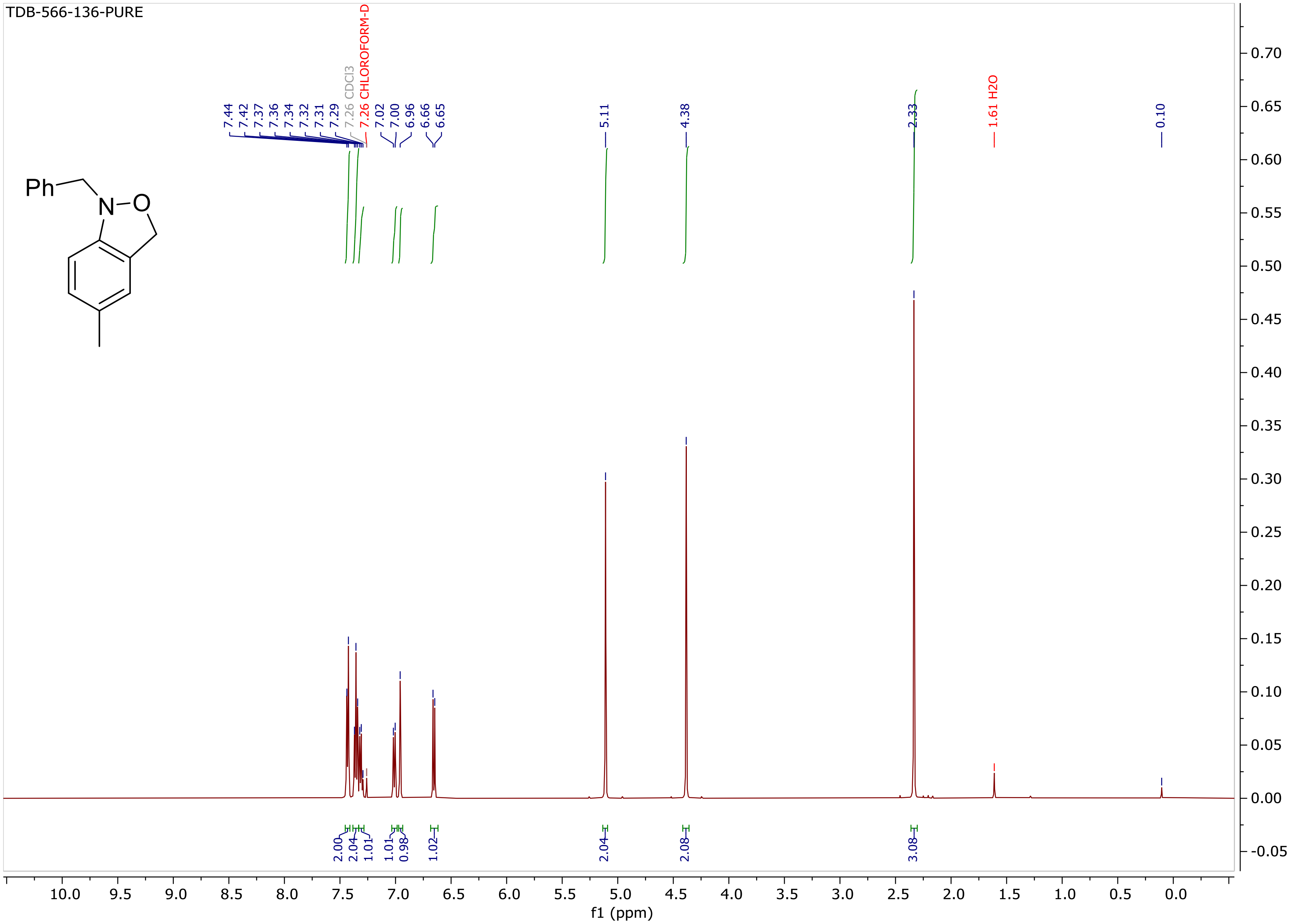

^{13}C NMR (126 MHz, CDCl$_3$) spectrum of 1-benzyl-5-methyl-1,3-dihydrobenzo[c]isoxazole (**26**):

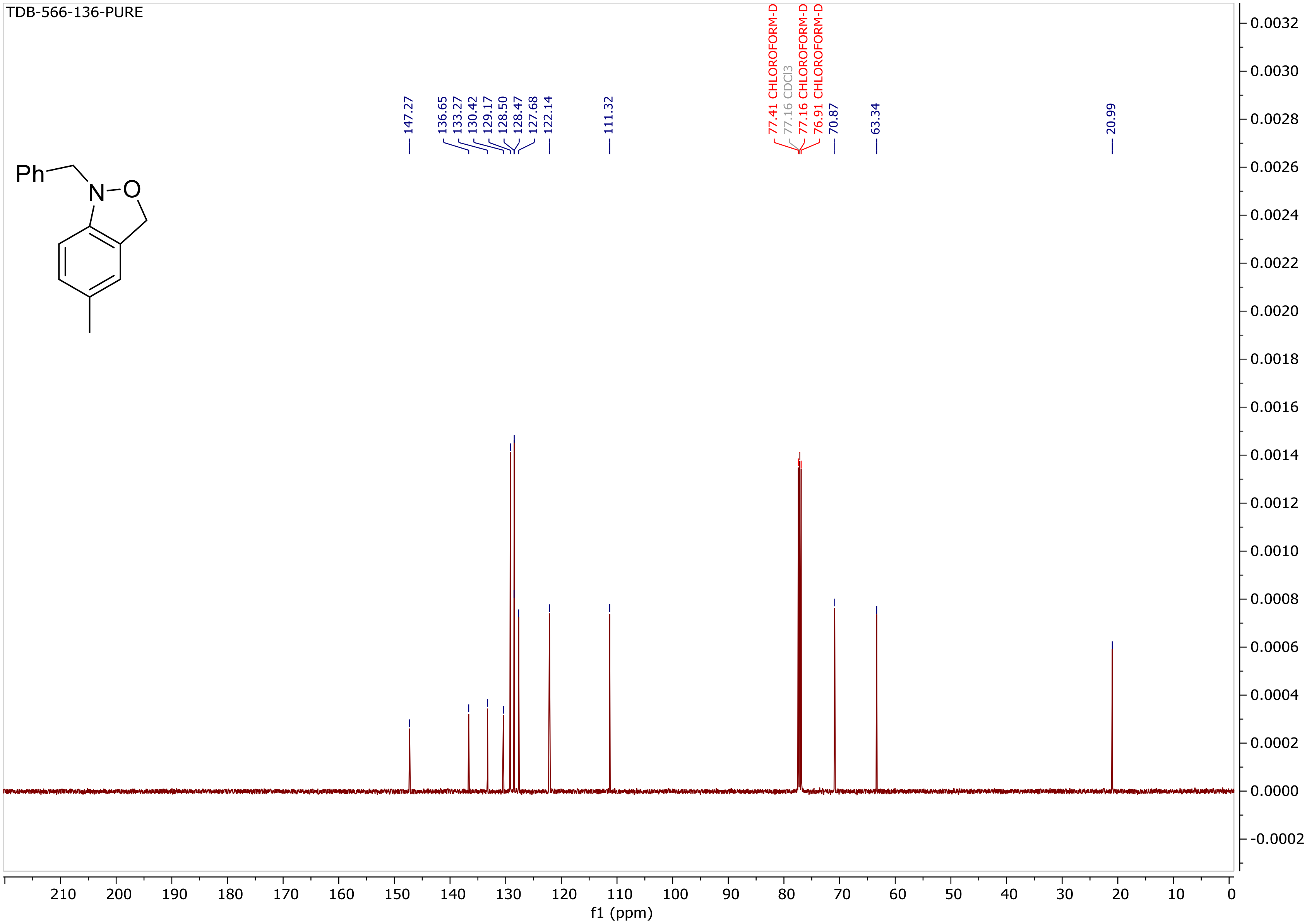

^{1}H NMR (500 MHz, CDCl₃) spectrum of 1-benzyl-5-bromo-1,3-dihydrobenzo[c]isoxazole (**27**):

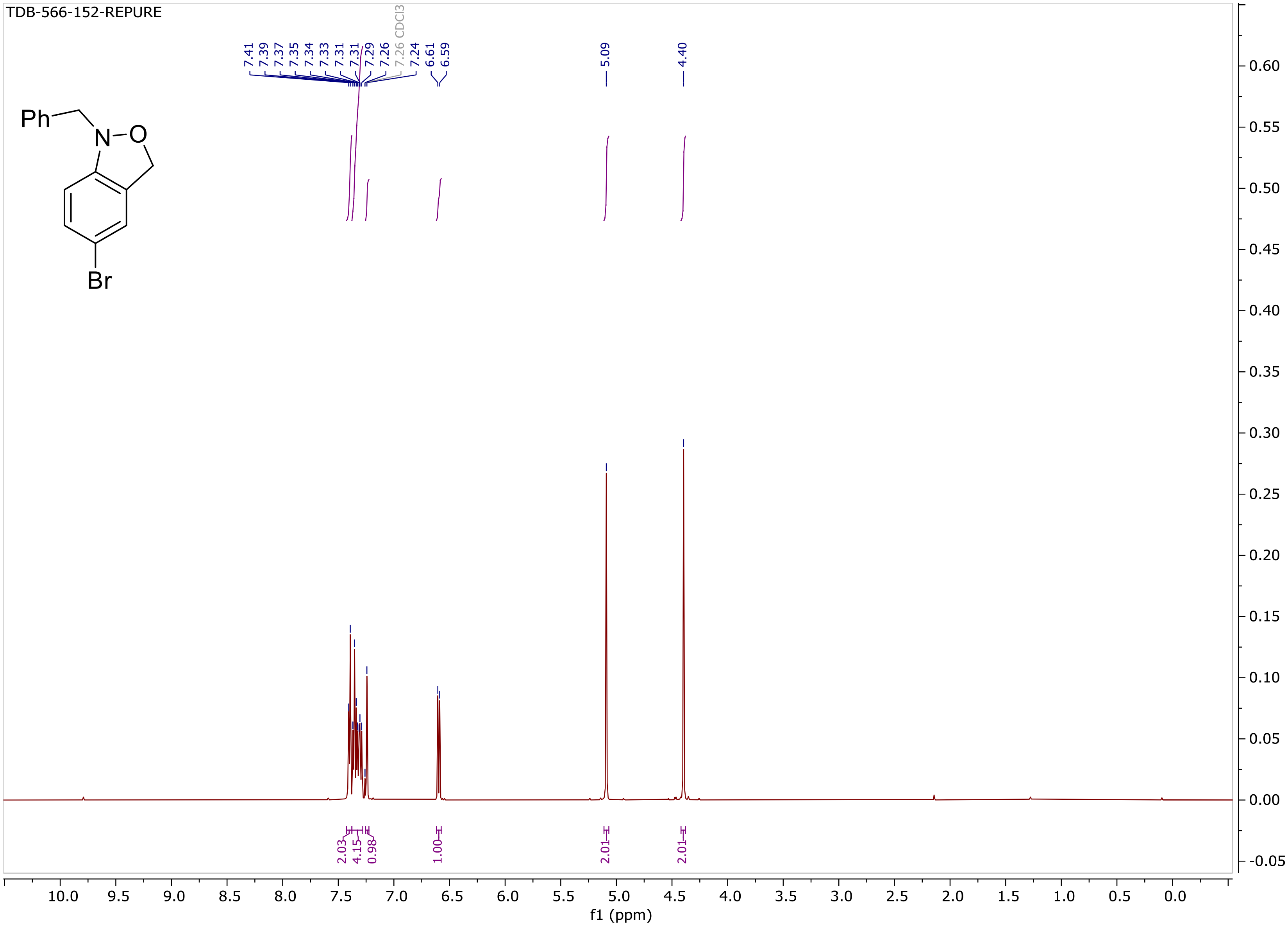

^{13}C NMR (126 MHz, CDCl$_3$) spectrum of 1-benzyl-5-bromo-1,3-dihydrobenzo[c]isoxazole (**27**):

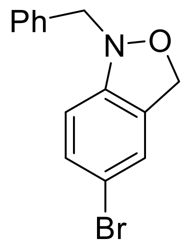

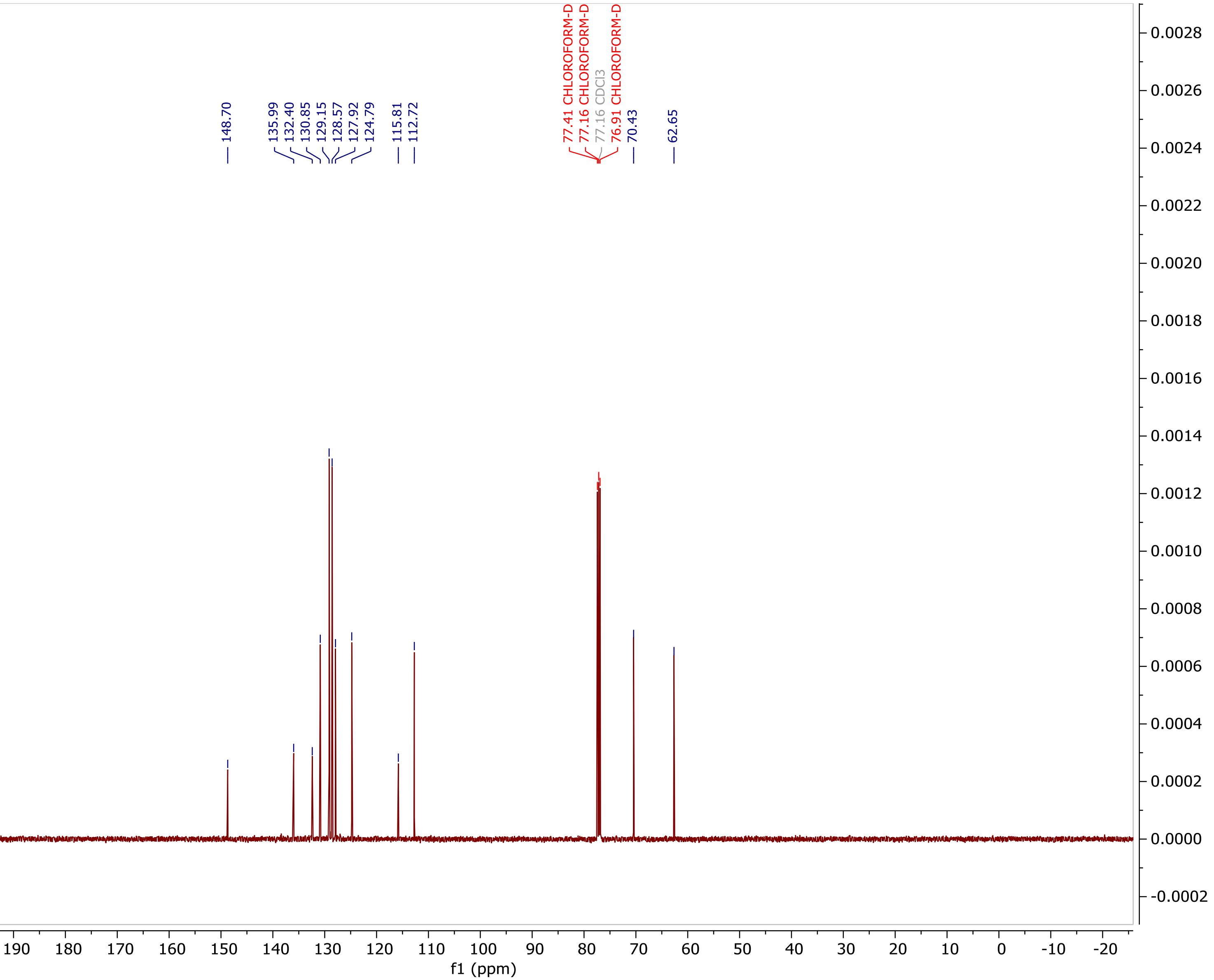

^{1}H NMR (500 MHz, $CDCl_3$) spectrum of 1-allyl-7-fluoro-1,3-dihydrobenzo[c]isoxazole (**28**):

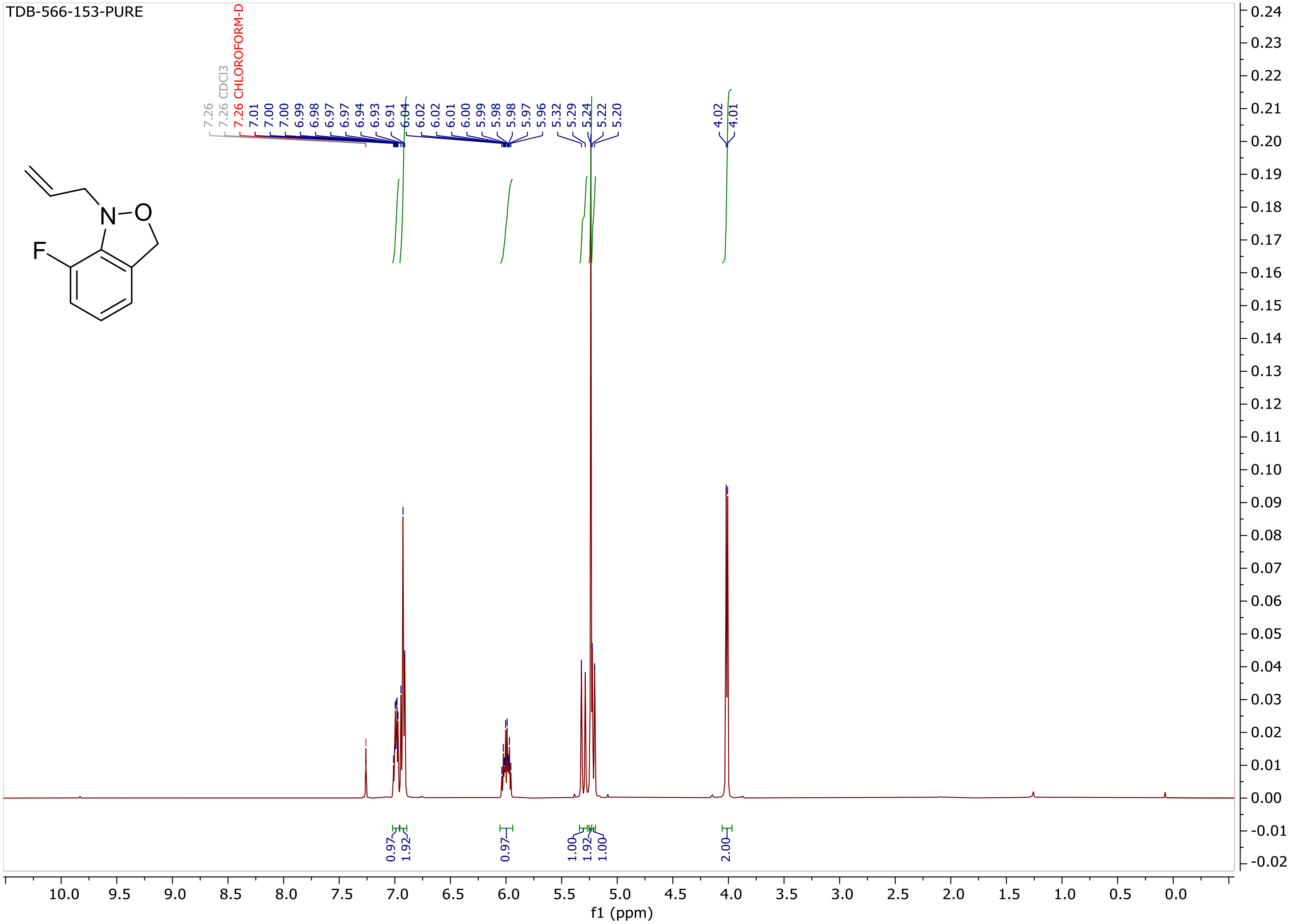

^{13}**C NMR (126 MHz, CDCl$_3$)** spectrum of 1-allyl-7-fluoro-1,3-dihydrobenzo[c]isoxazole (**28**):

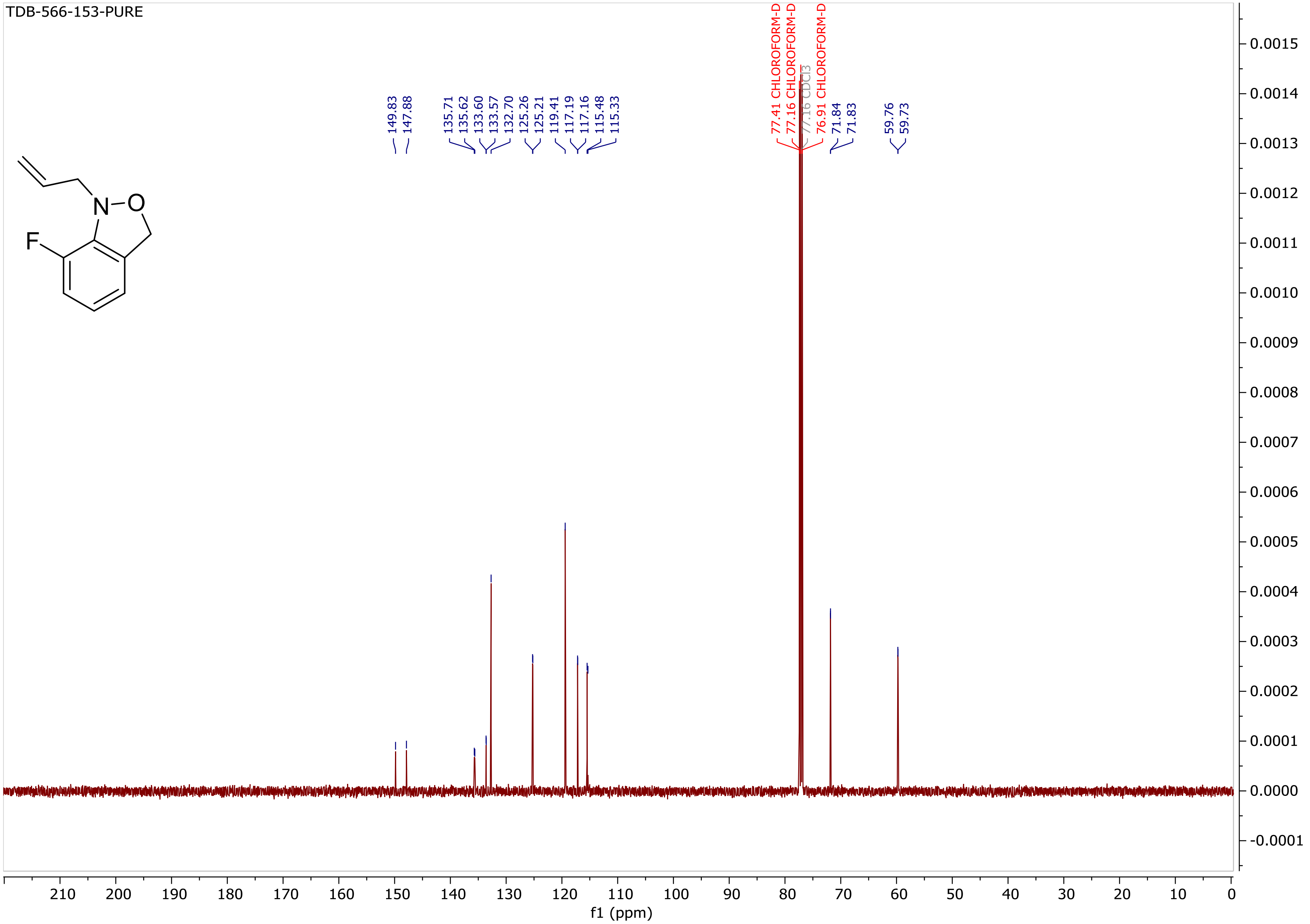

^{13}C NMR {^{19}F} (126 MHz, CDCl$_3$) spectrum of 1-allyl-7-fluoro-1,3-dihydrobenzo[c]isoxazole (**28**):

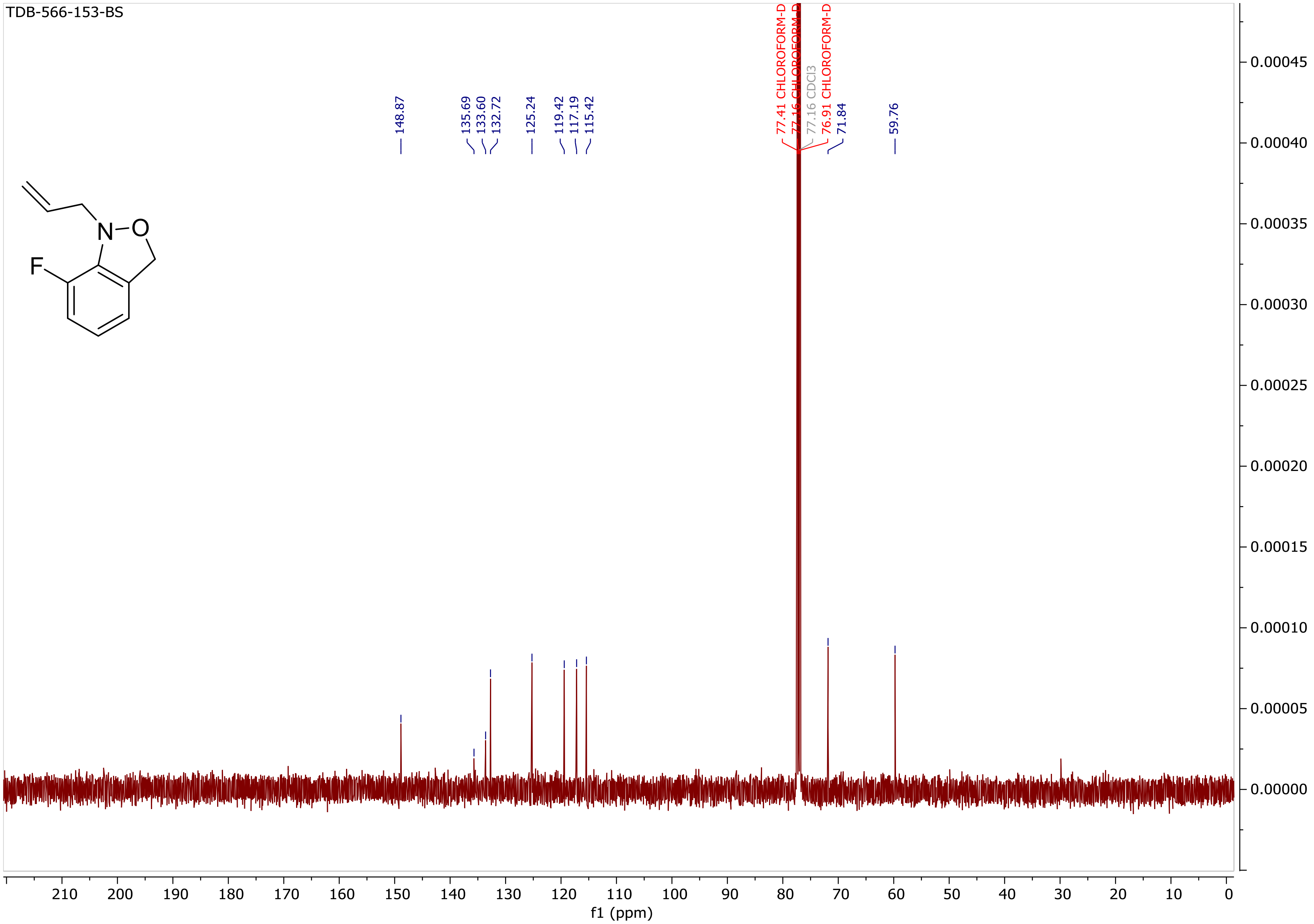

Expanded region of stacked (top) **^{13}C NMR (126 MHz, $CDCl_3$)** and (bottom) **^{13}C NMR {^{19}F} (126 MHz, $CDCl_3$)** spectrum of 1-allyl-7-fluoro-1,3-dihydrobenzo[c]isoxazole (**28**):

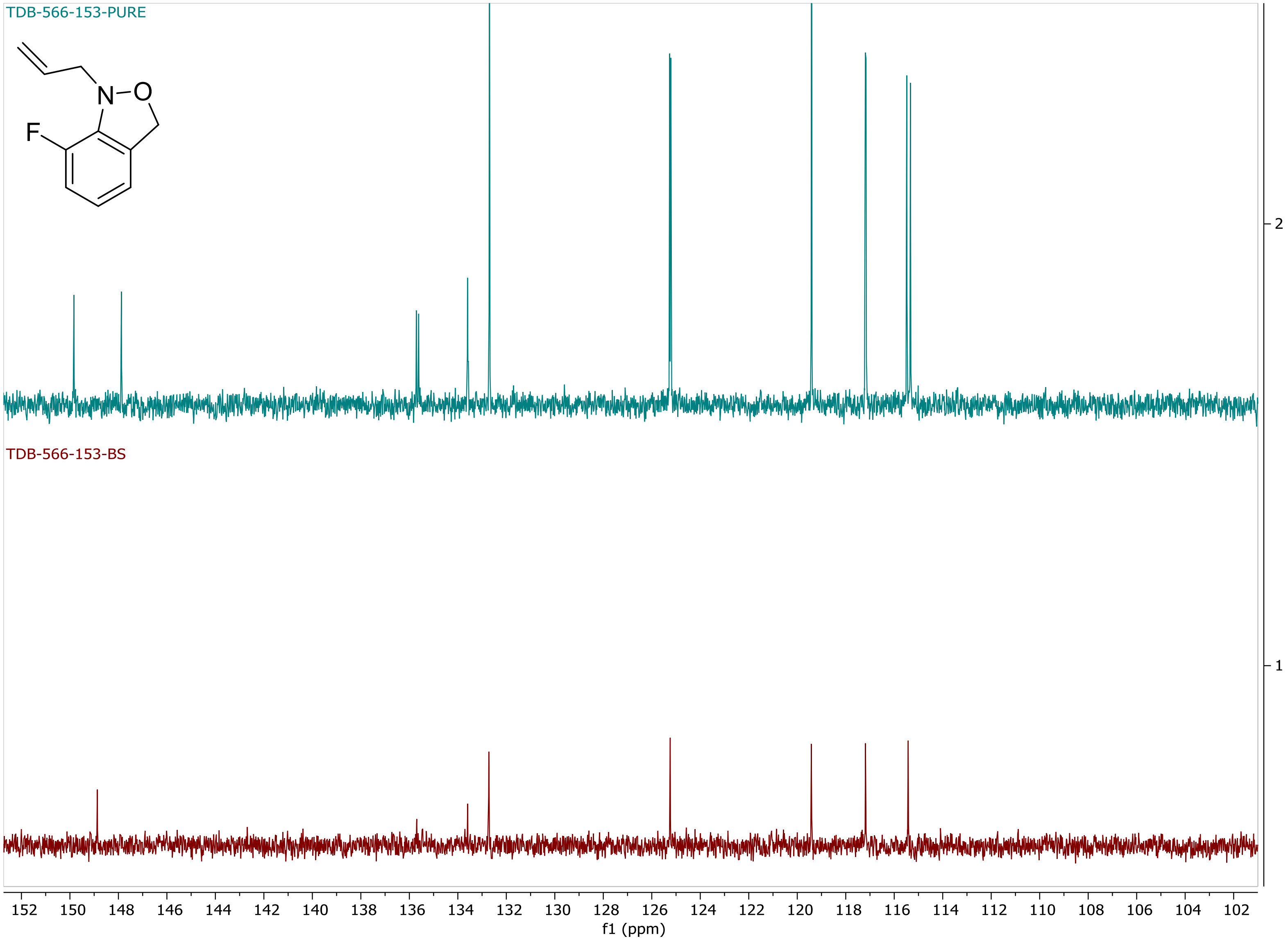

^{19}F NMR {1H} (470 MHz, $CDCl_3$) spectrum of 1-allyl-7-fluoro-1,3-dihydrobenzo[c]isoxazole (**28**):

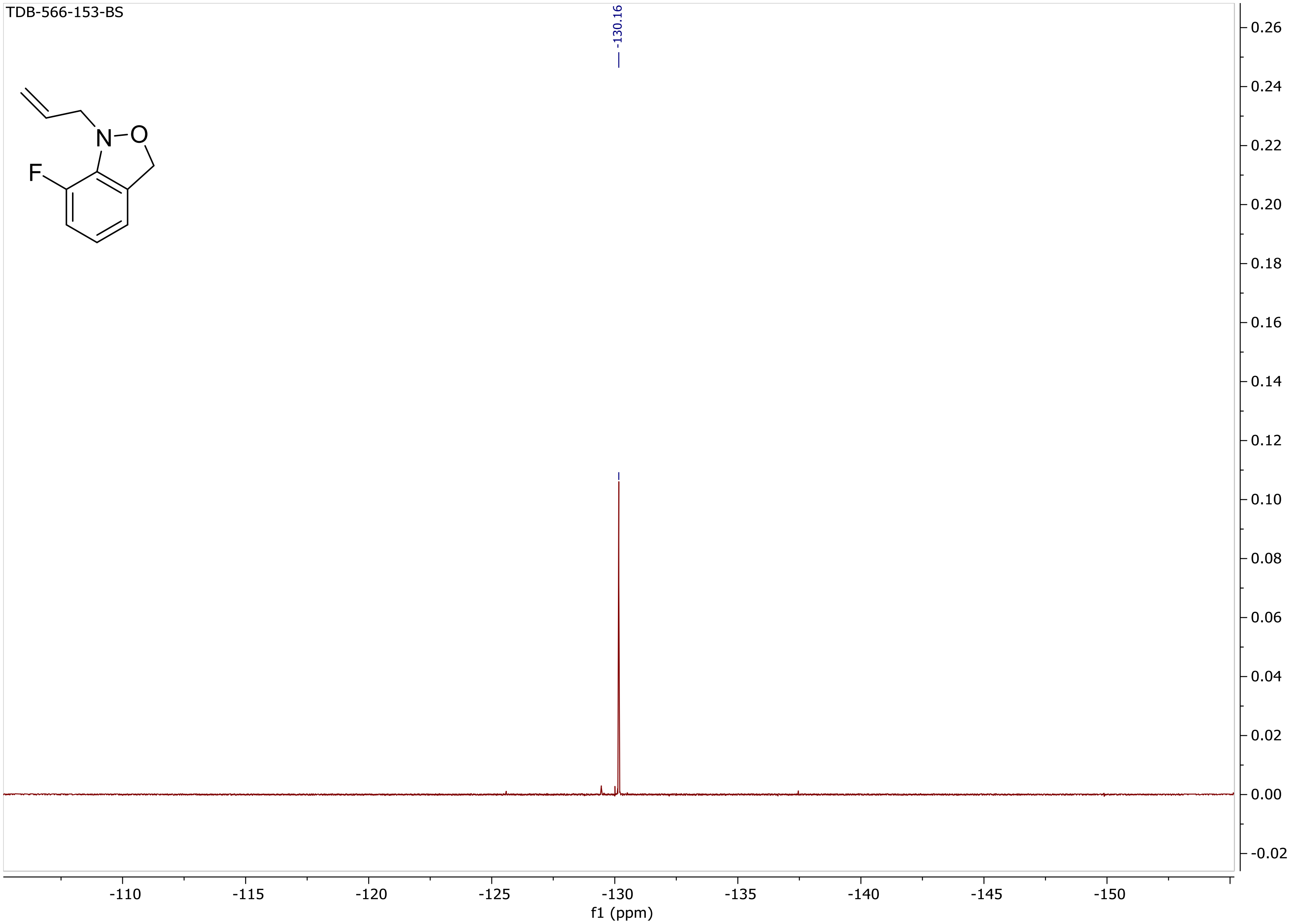

^{1}H NMR (500 MHz, CDCl$_3$) spectrum of 6-bromo-1-(4-bromobenzyl)-1,3-dihydrobenzo[c]isoxazole (**29**):

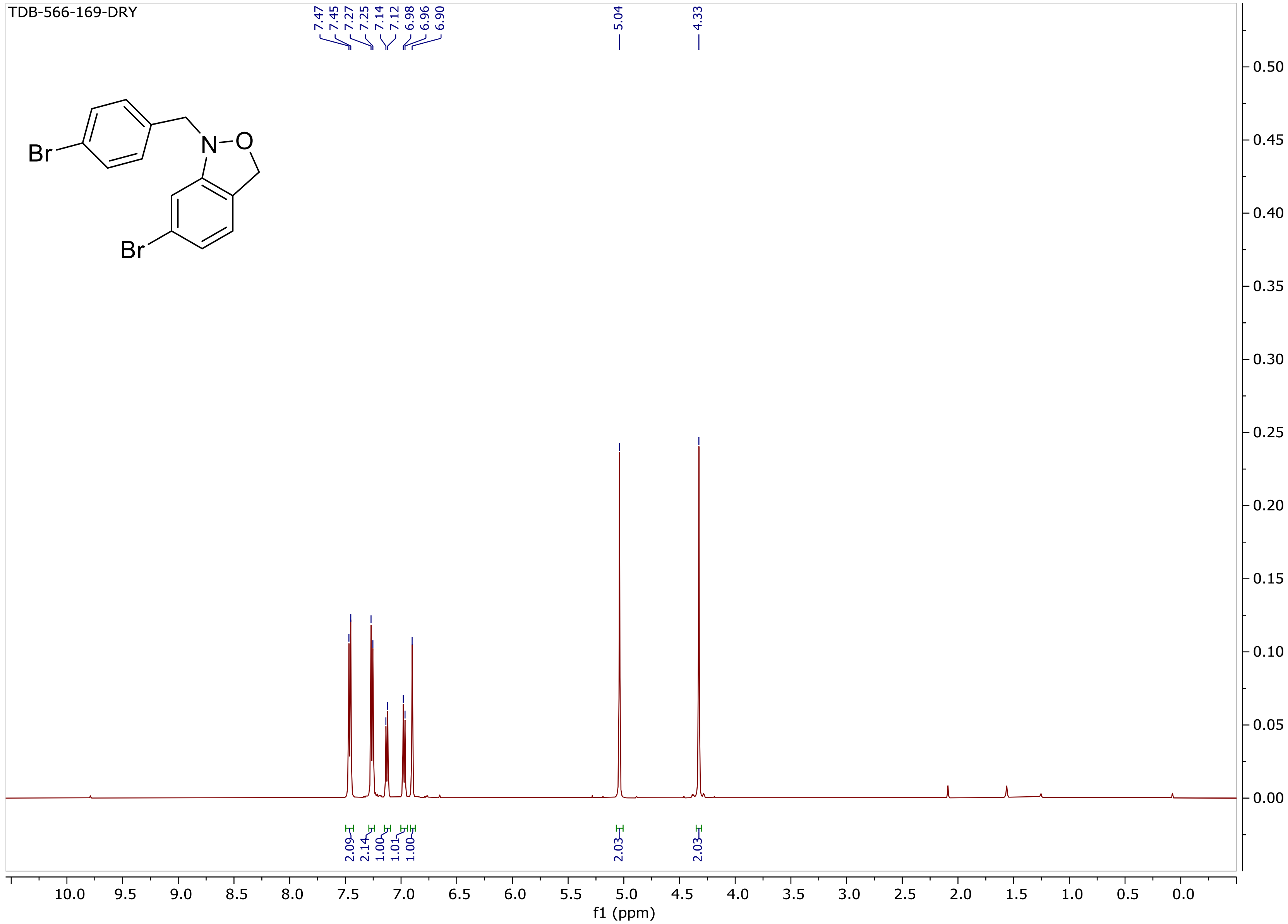

^{13}C NMR (126 MHz, CDCl$_3$) spectrum of 6-bromo-1-(4-bromobenzyl)-1,3-dihydrobenzo[c]isoxazole (**29**):

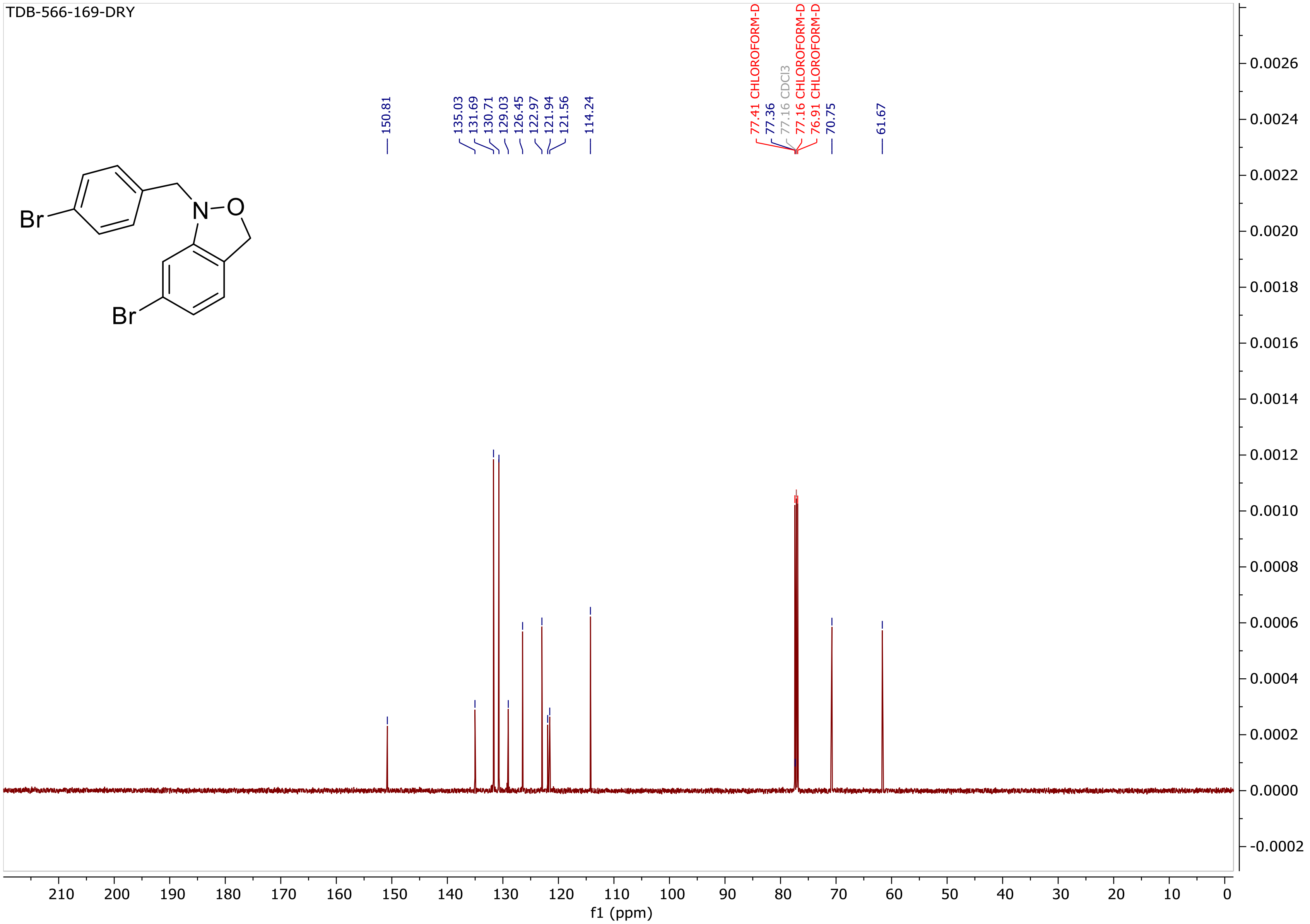

^{1}H NMR (500 MHz, CDCl₃) spectrum of 1-allyl-5-fluoro-1,3-dihydrobenzo[c]isoxazole (**30**):

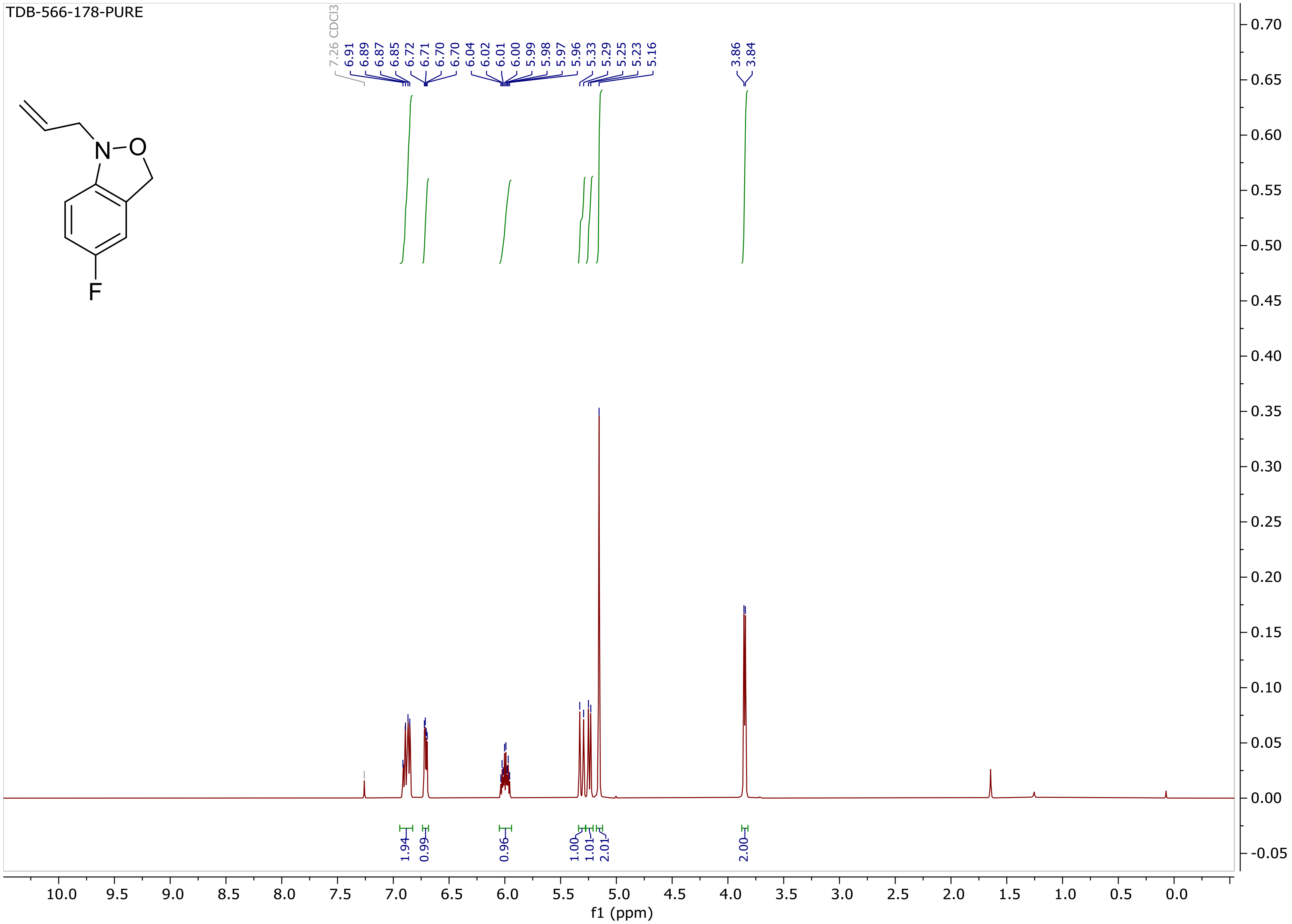

^{13}C NMR (126 MHz, $CDCl_3$) spectrum of 1-allyl-5-fluoro-1,3-dihydrobenzo[c]isoxazole (**30**):

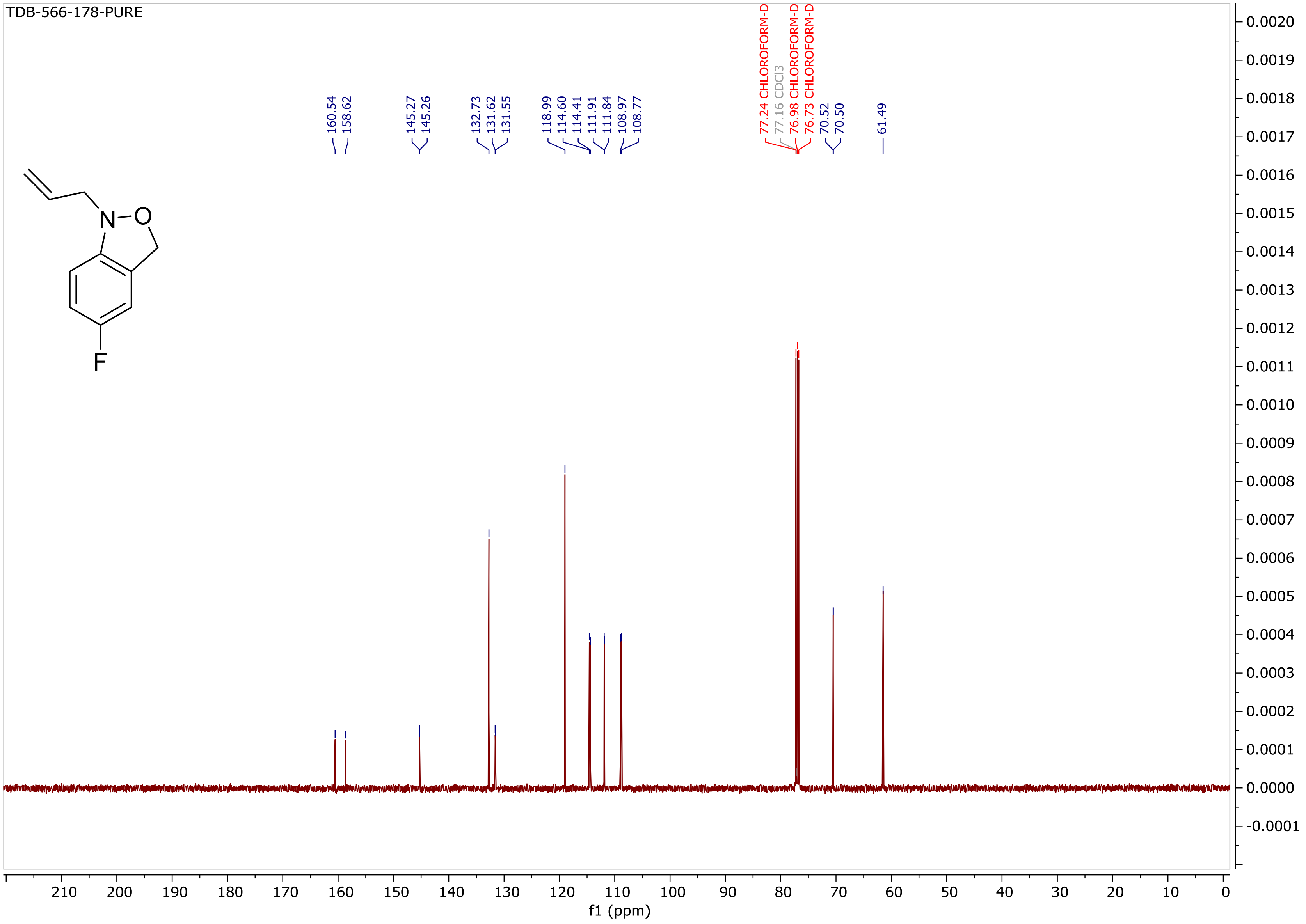

^{13}**C NMR {^{19}F} (126 MHz, CDCl$_3$)** spectrum of 1-allyl-5-fluoro-1,3-dihydrobenzo[c]isoxazole (**30**):

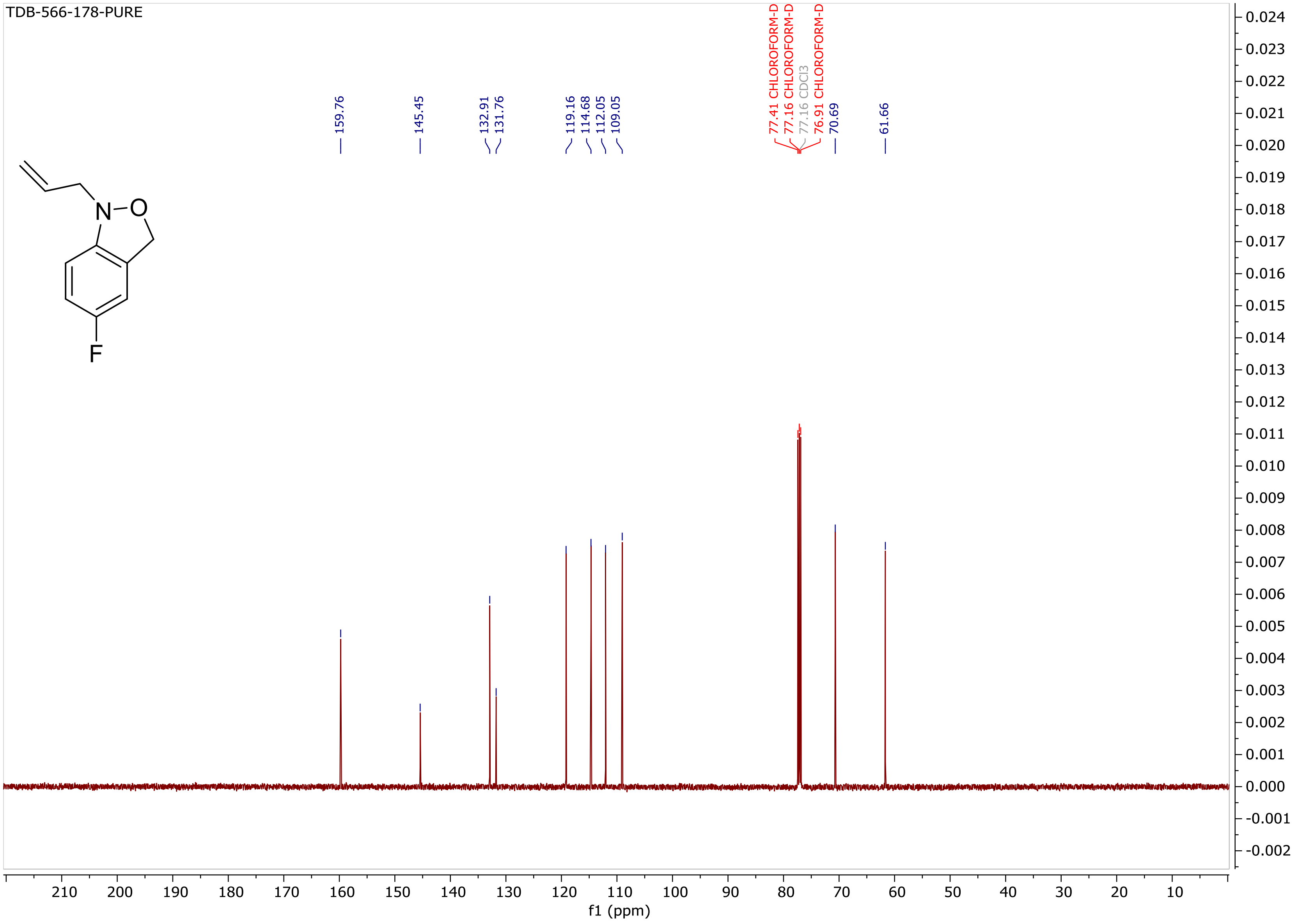

Expanded region of stacked (top) ^{13}C NMR (126 MHz, CDCl$_3$) and (bottom) ^{13}C NMR {^{19}F} (126 MHz, CDCl$_3$) spectrum of 1-allyl-5-fluoro-1,3-dihydrobenzo[c]isoxazole (**30**):

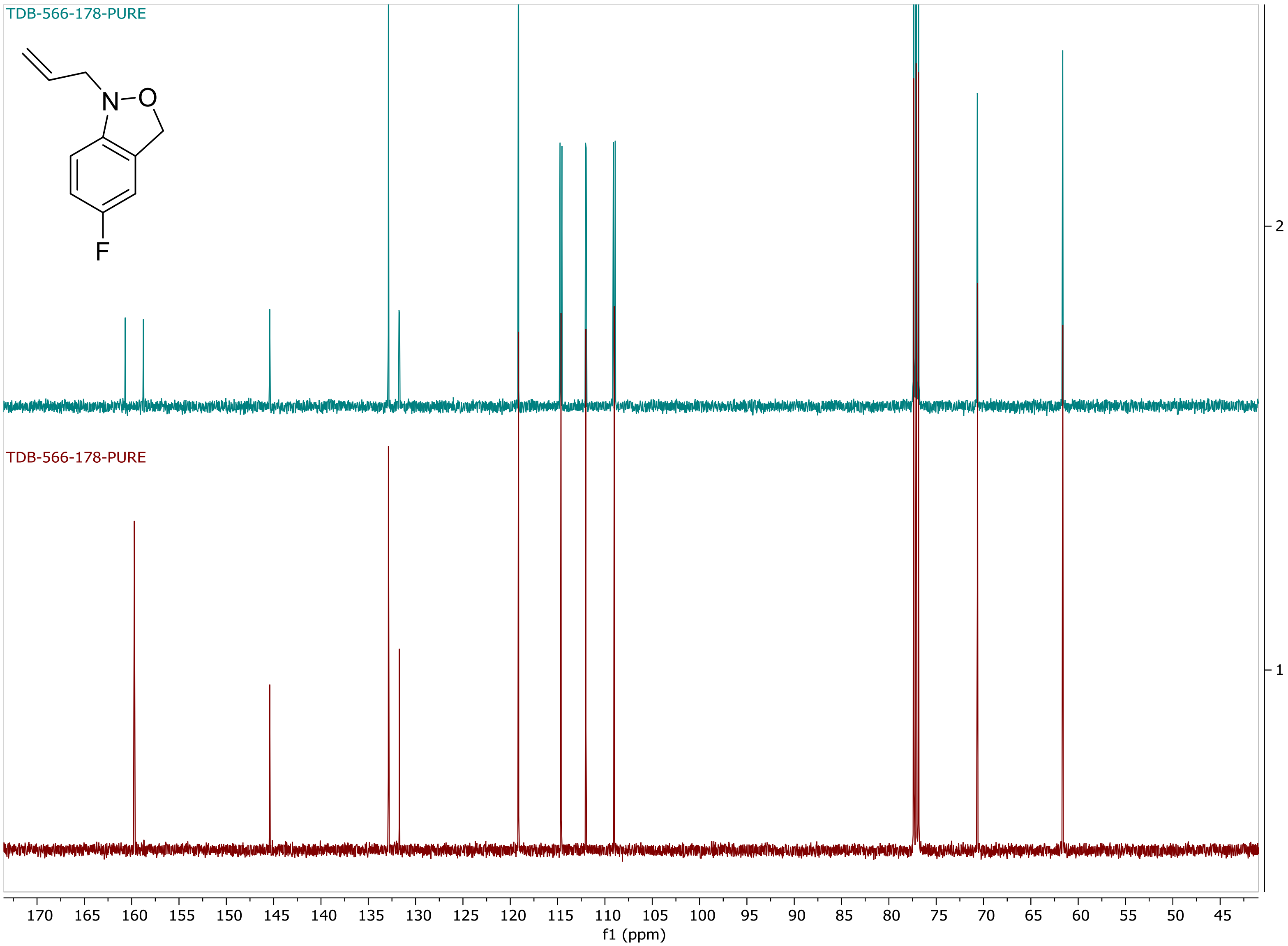

^{19}F NMR {1H} (470 MHz, CDCl$_3$) spectrum of 1-allyl-5-fluoro-1,3-dihydrobenzo[c]isoxazole (**30**):

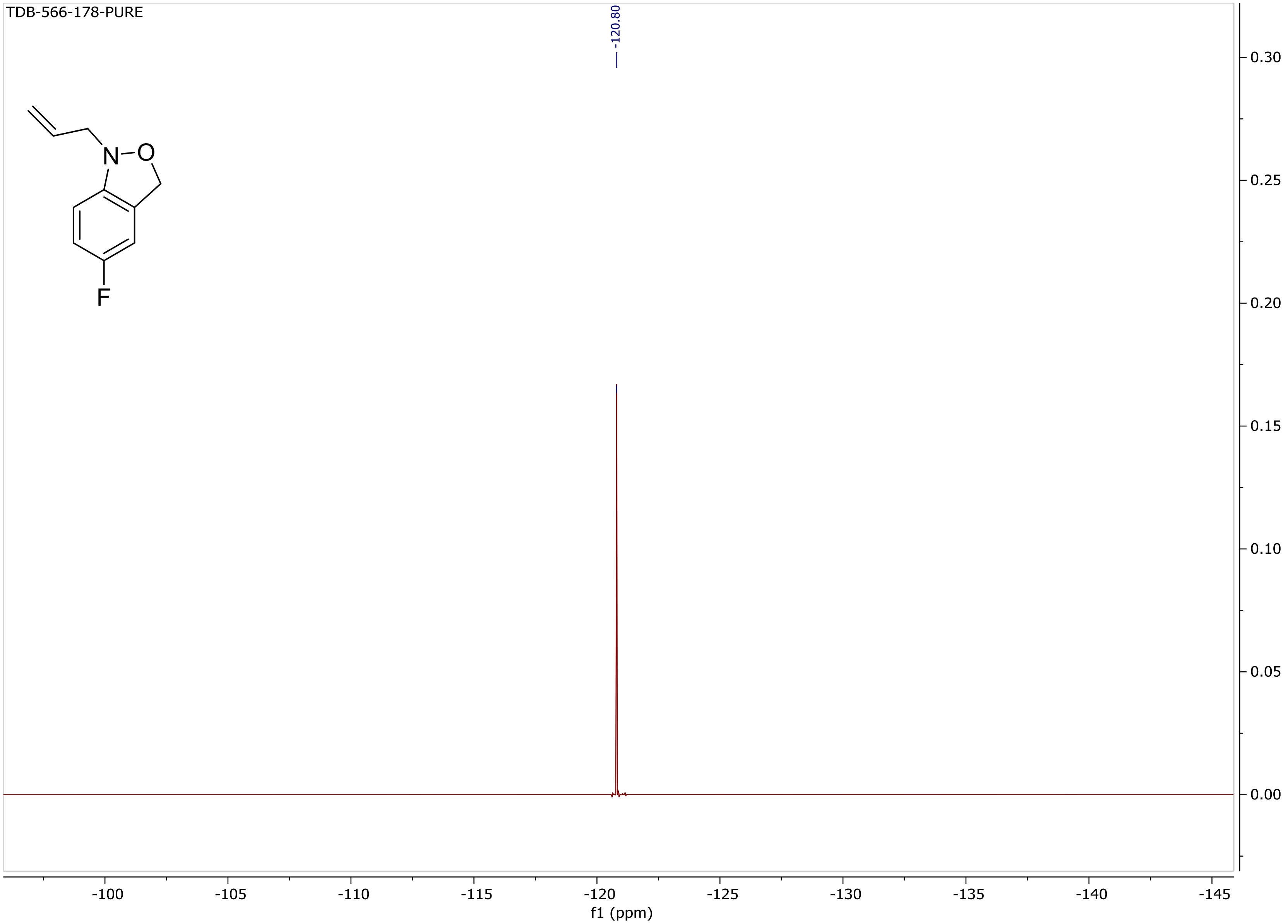

^{1}**H NMR (500 MHz, CDCl$_3$)** spectrum of 1-(4-bromobenzyl)-6-fluoro-1,3-dihydrobenzo[c]isoxazole (**31**):

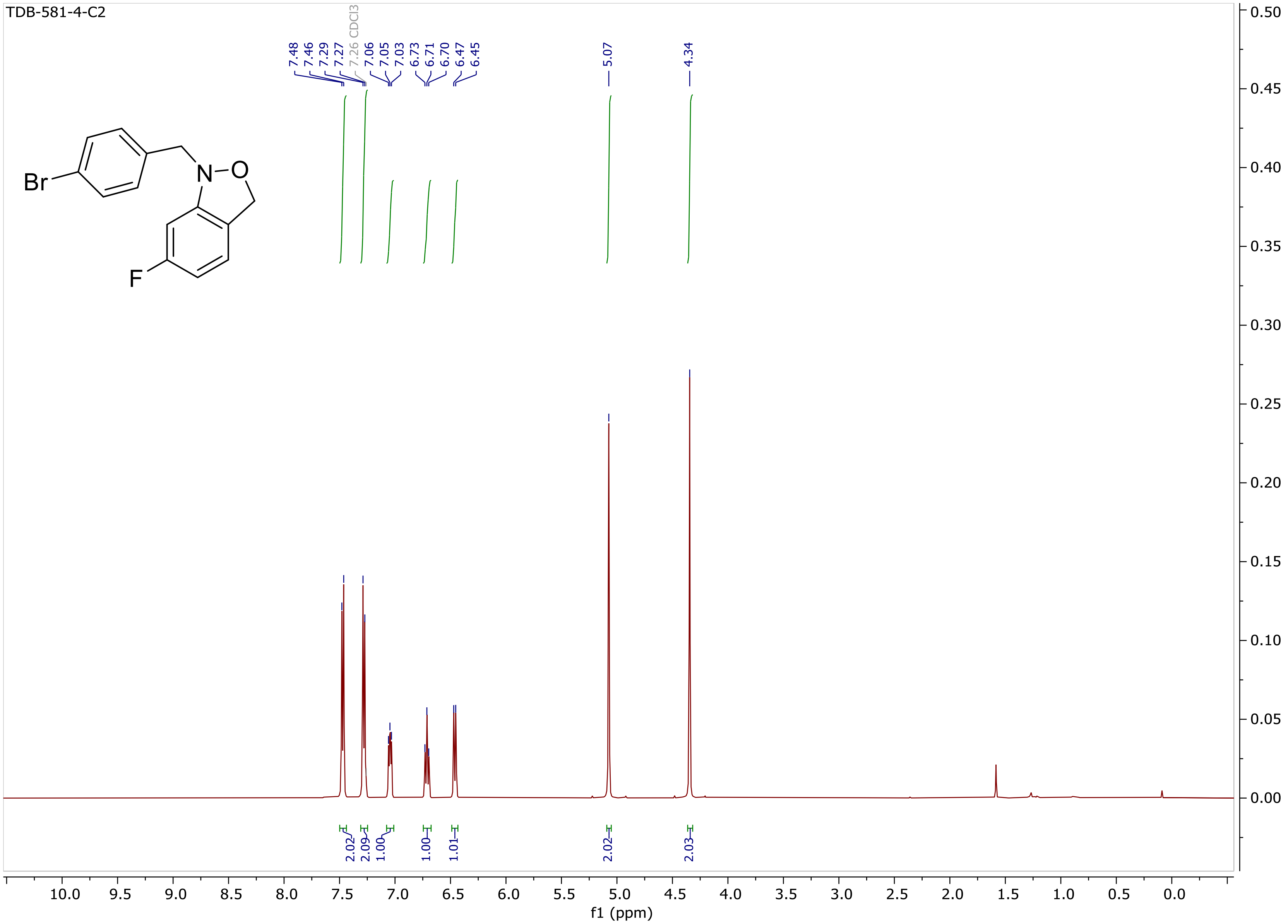

^{13}**C NMR (126 MHz, CDCl$_3$)** spectrum of 1-(4-bromobenzyl)-6-fluoro-1,3-dihydrobenzo[c]isoxazole (**31**):

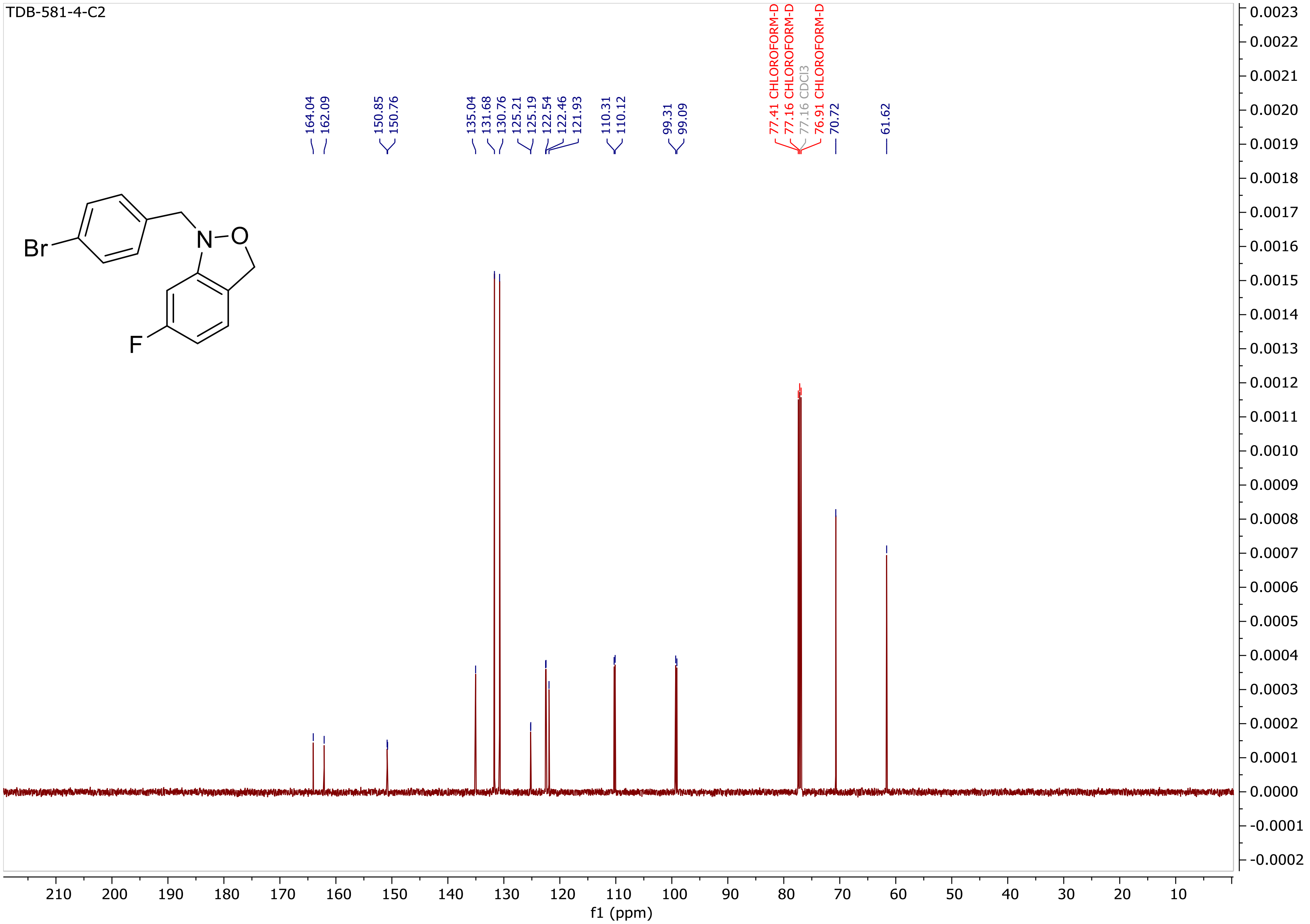

^{13}C NMR {^{19}F} (126 MHz, CDCl$_3$) spectrum of 1-(4-bromobenzyl)-6-fluoro-1,3-dihydrobenzo[c]isoxazole (**31**):

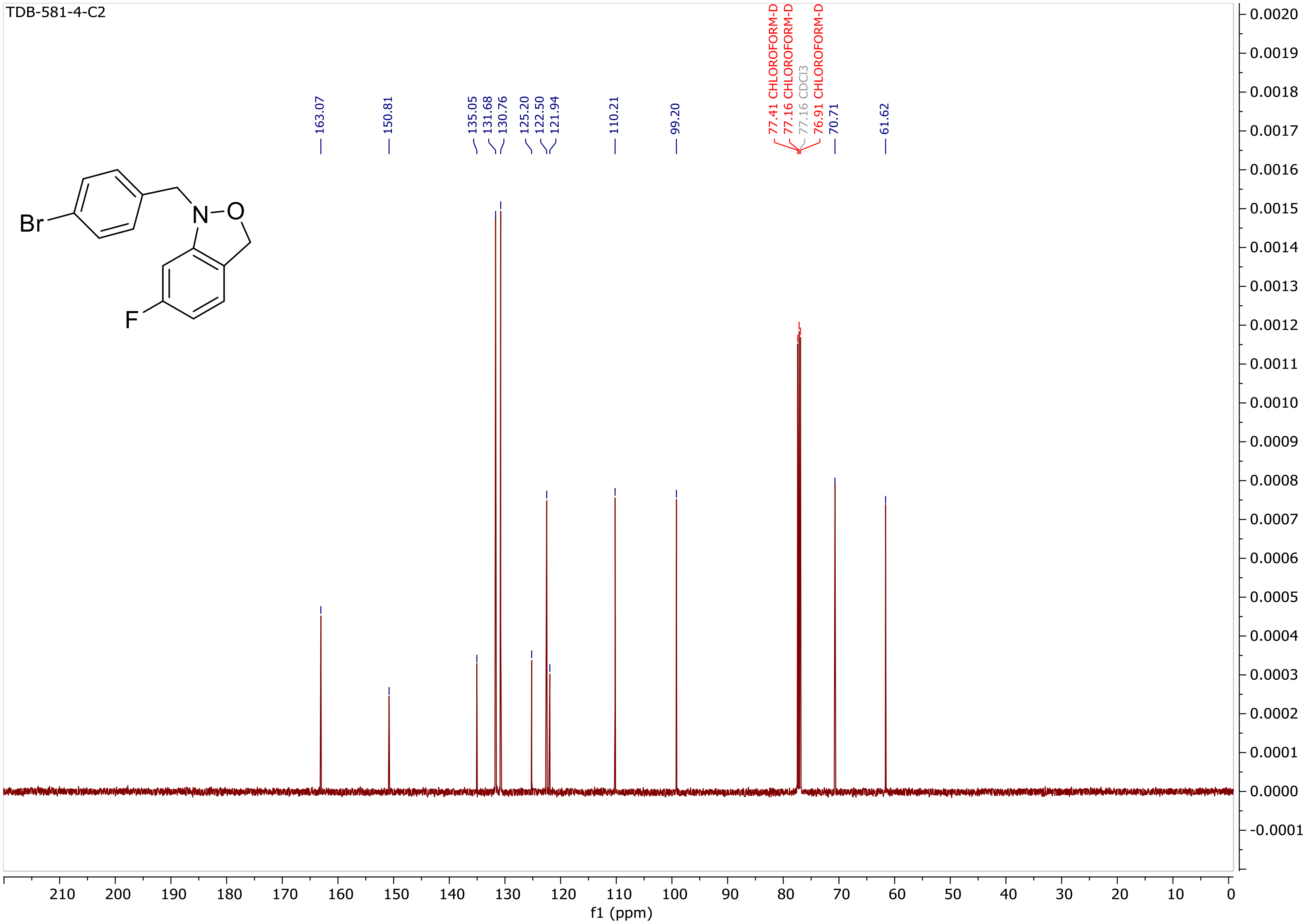

Expanded region of stacked (top) ^{13}C NMR (126 MHz, CDCl$_3$) and (bottom) ^{13}C NMR {^{19}F} (126 MHz, CDCl$_3$) spectrum of 1-(4-bromobenzyl)-6-fluoro-1,3-dihydrobenzo[c]isoxazole (**31**):

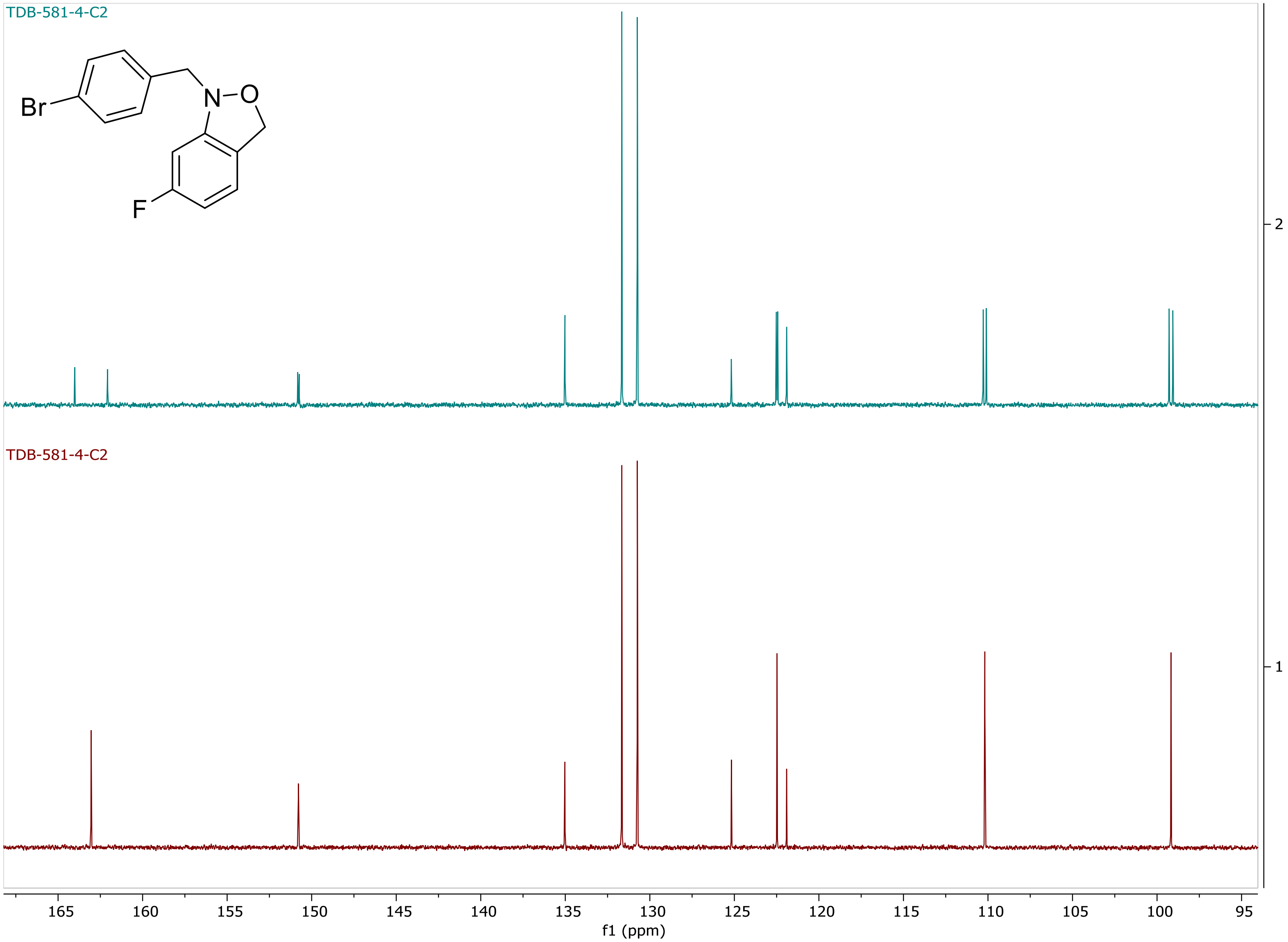

^{19}F NMR {1H} (470 MHz, CDCl$_3$) spectrum of 1-(4-bromobenzyl)-6-fluoro-1,3-dihydrobenzo[c]isoxazole (**31**):

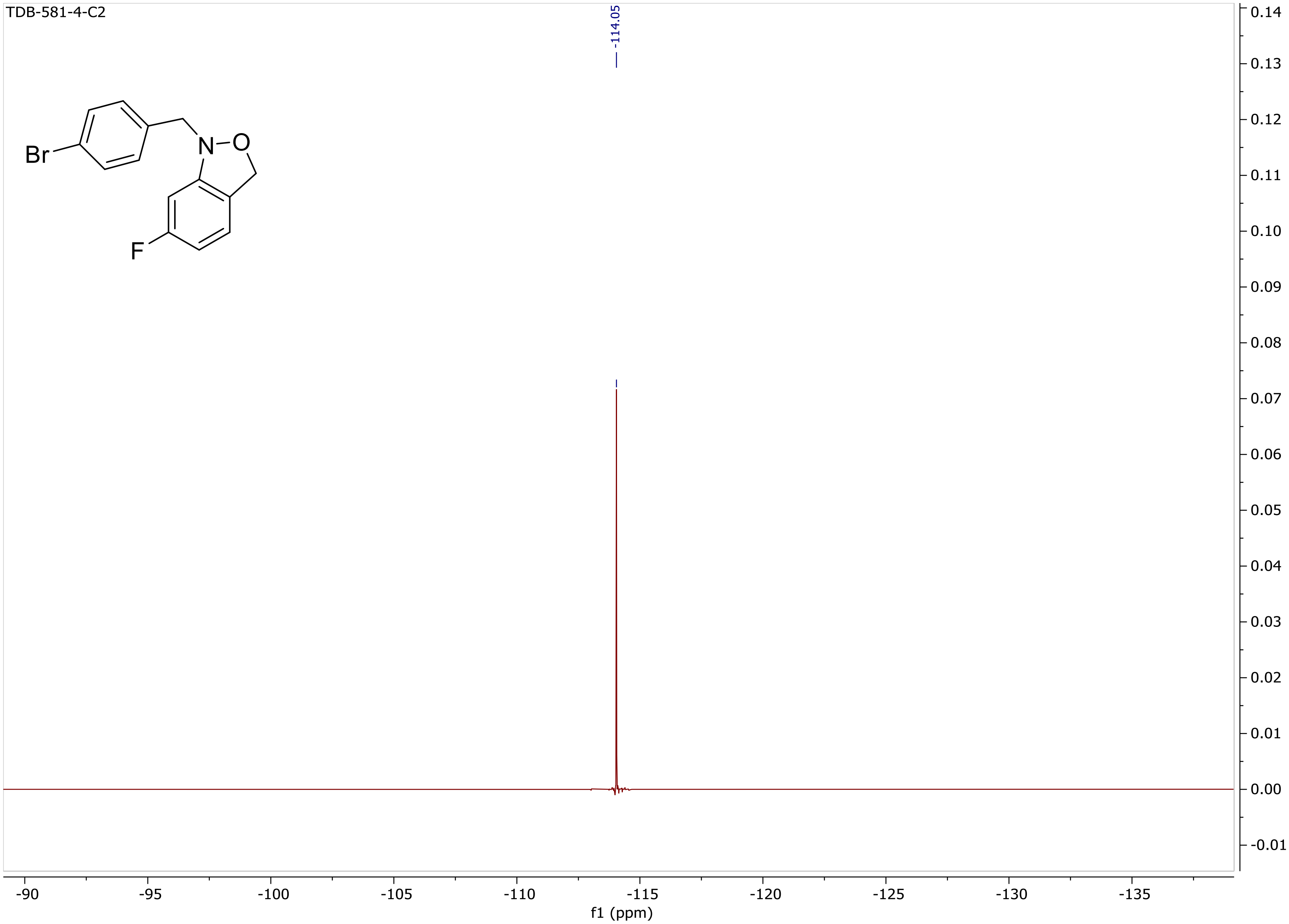

^{1}H NMR (500 MHz, CDCl₃) spectrum of 1-allyl-6-methoxy-1,3-dihydrobenzo[c]isoxazole (**32**):

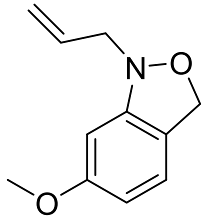

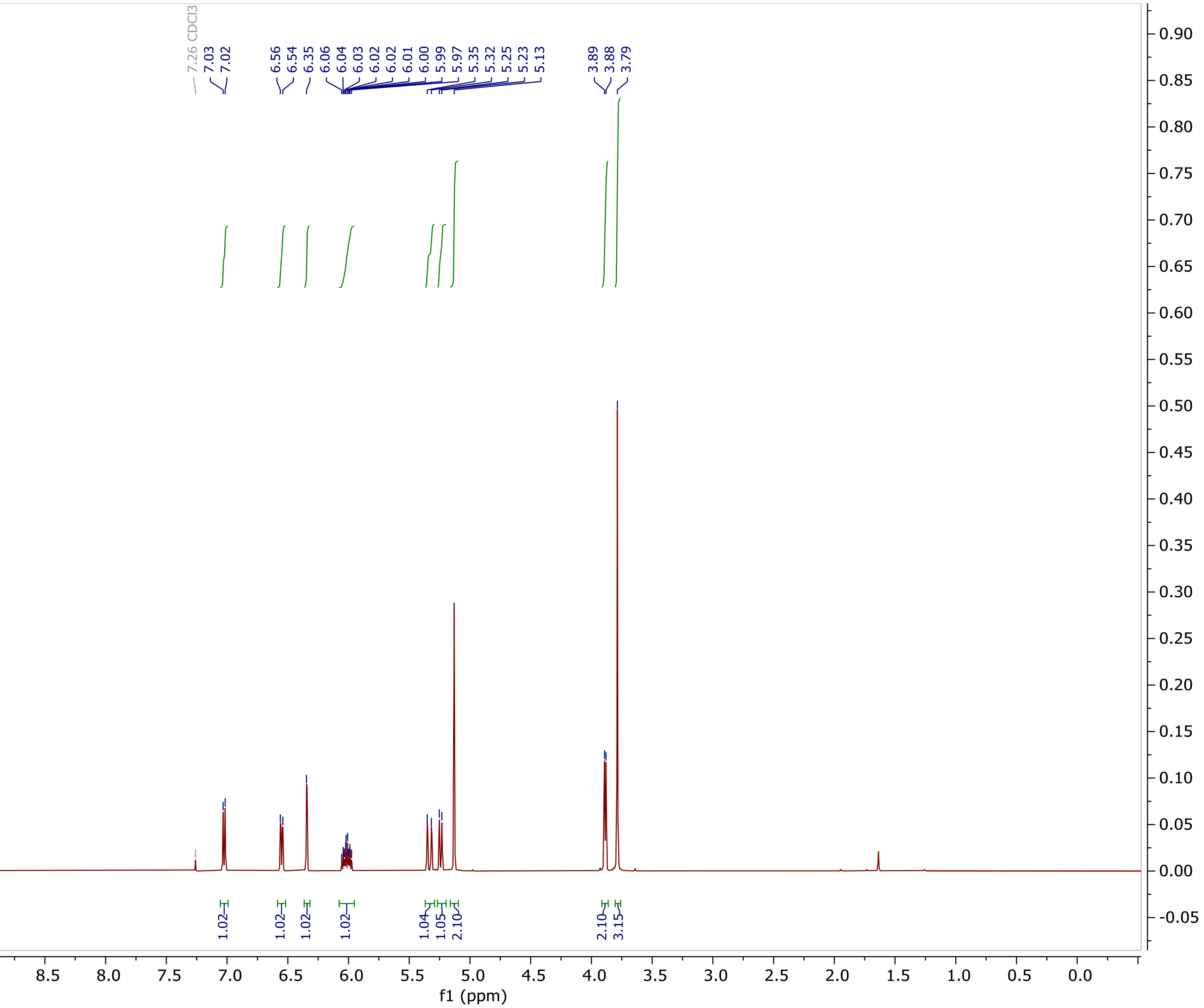

^{13}C NMR (126 MHz, CDCl3) spectrum of 1-allyl-6-methoxy-1,3-dihydrobenzo[c]isoxazole (**32**):

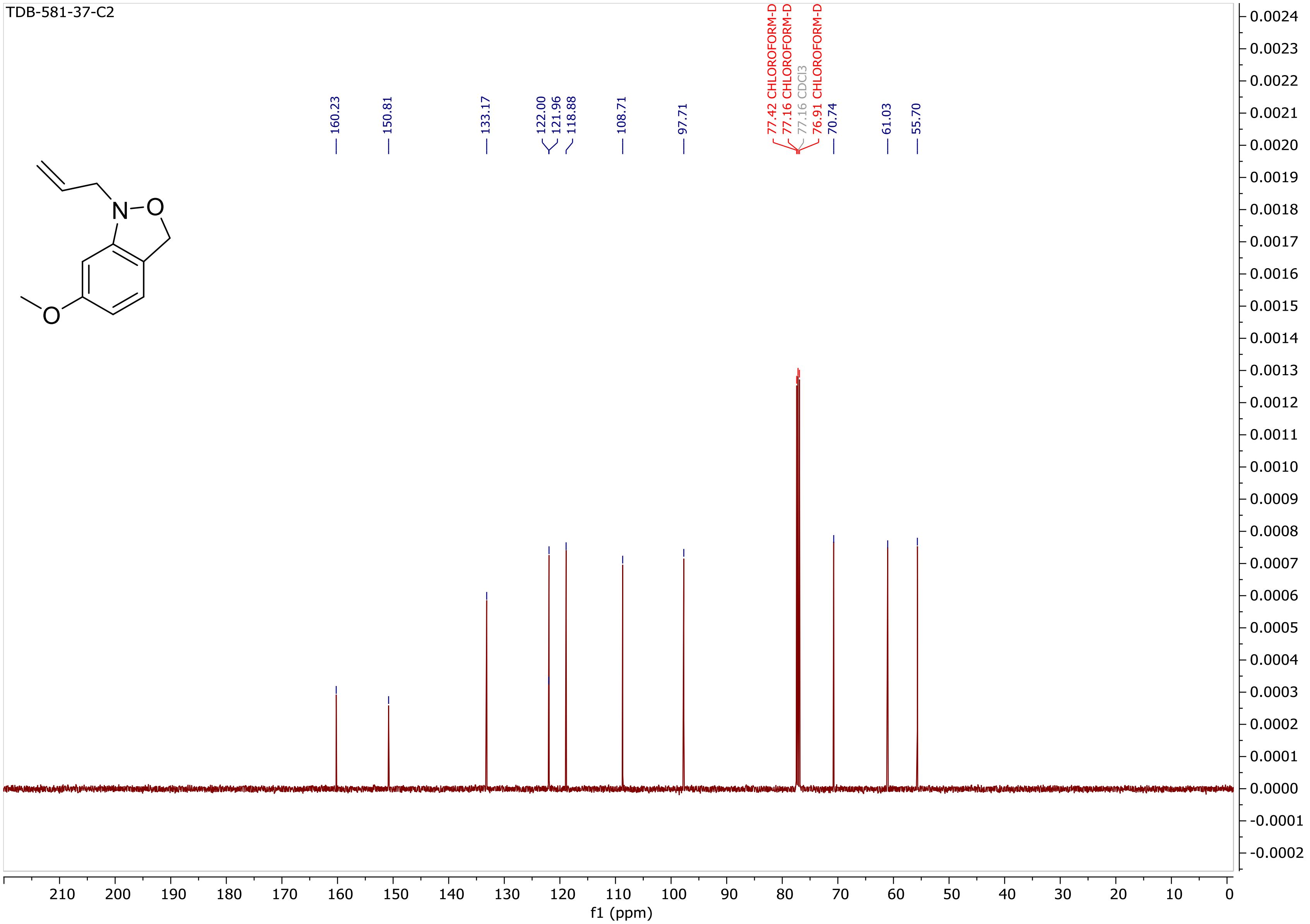

^{1}H NMR (500 MHz, CDCl$_3$) spectrum of 1-benzyl-6-bromo-1,3-dihydrobenzo[c]isoxazole **(33):**

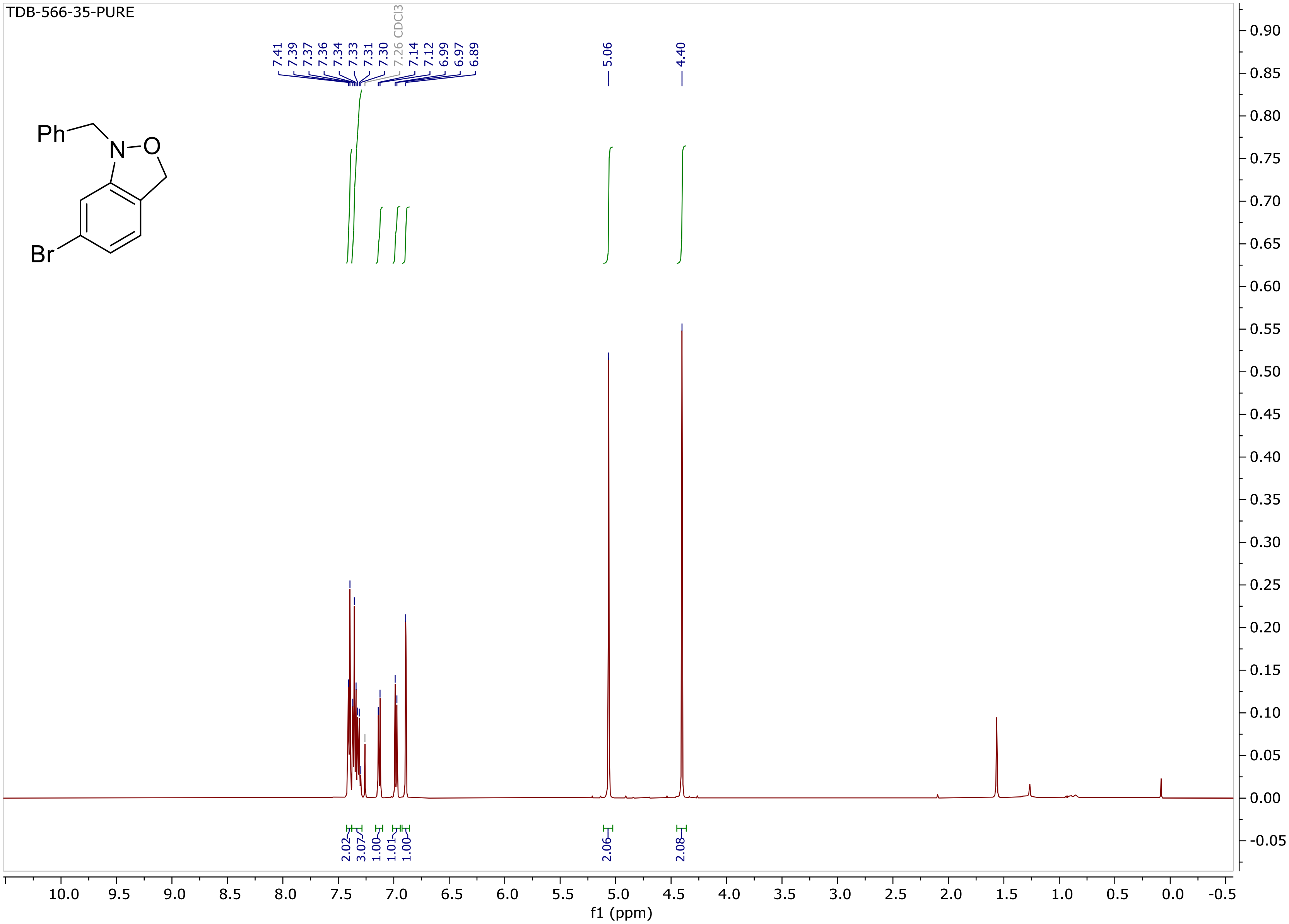

^{13}C NMR (126 MHz, CDCl$_3$) spectrum of 1-benzyl-6-bromo-1,3-dihydrobenzo[c]isoxazole **(33):**

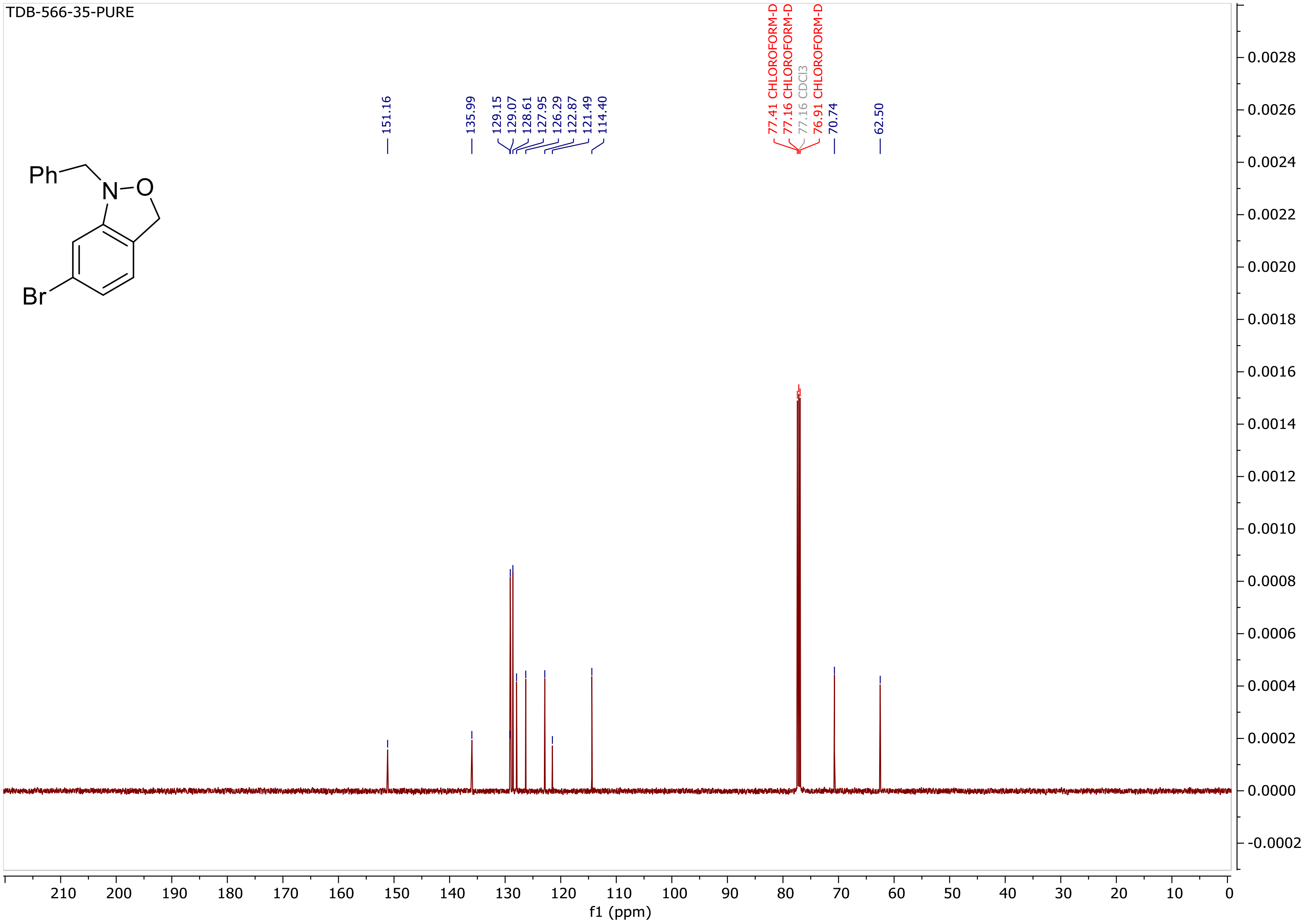

^{1}H NMR (500 MHz, CDCl₃) spectrum of 1-(4-bromobenzyl)-6-(trifluoromethyl)-1,3-dihydrobenzo[*c*]isoxazole (**34**):

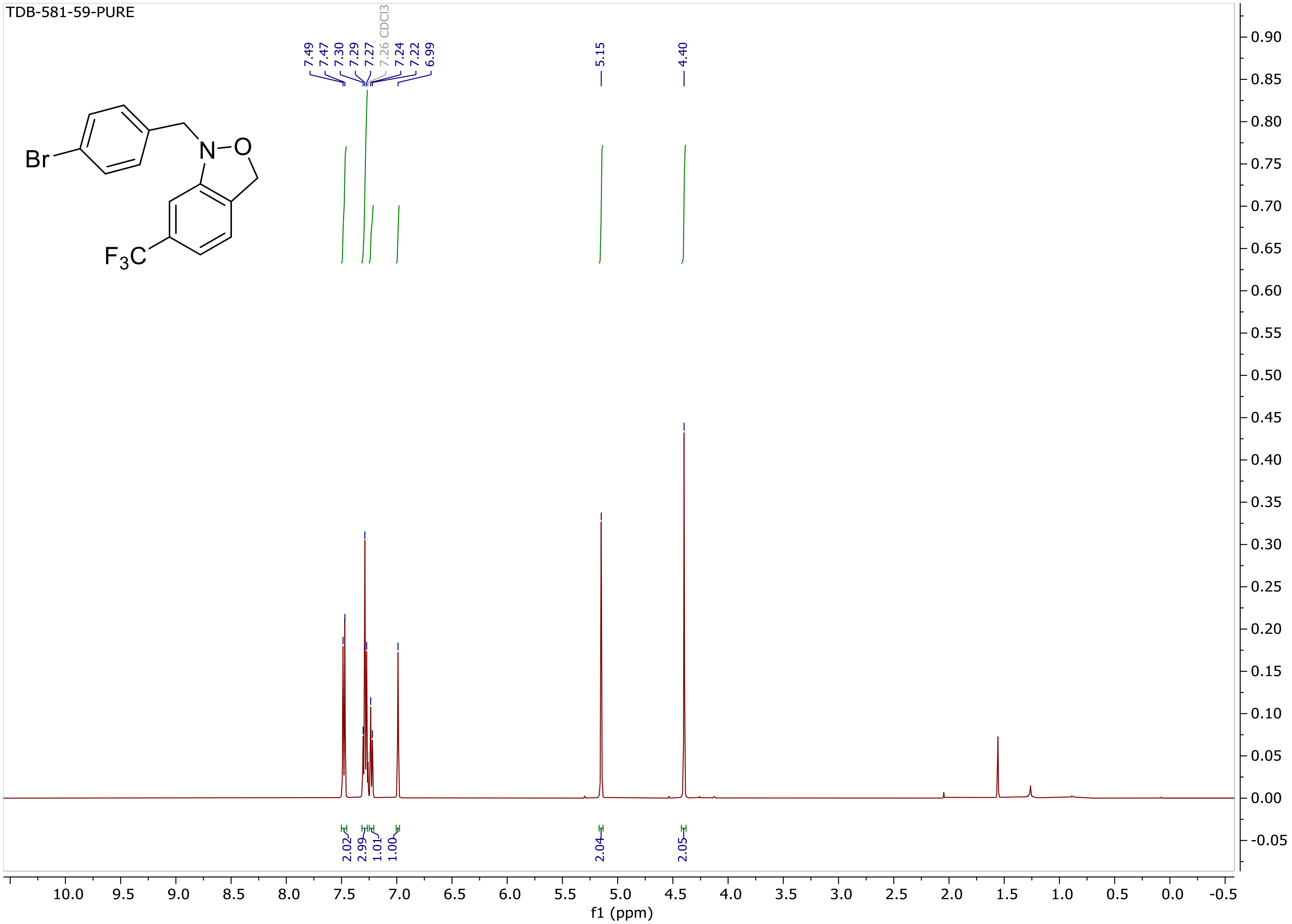

^{13}C NMR (126 MHz, CDCl$_3$) spectrum of 1-(4-bromobenzyl)-6-(trifluoromethyl)-1,3-dihydrobenzo[*c*]isoxazole (**34**):

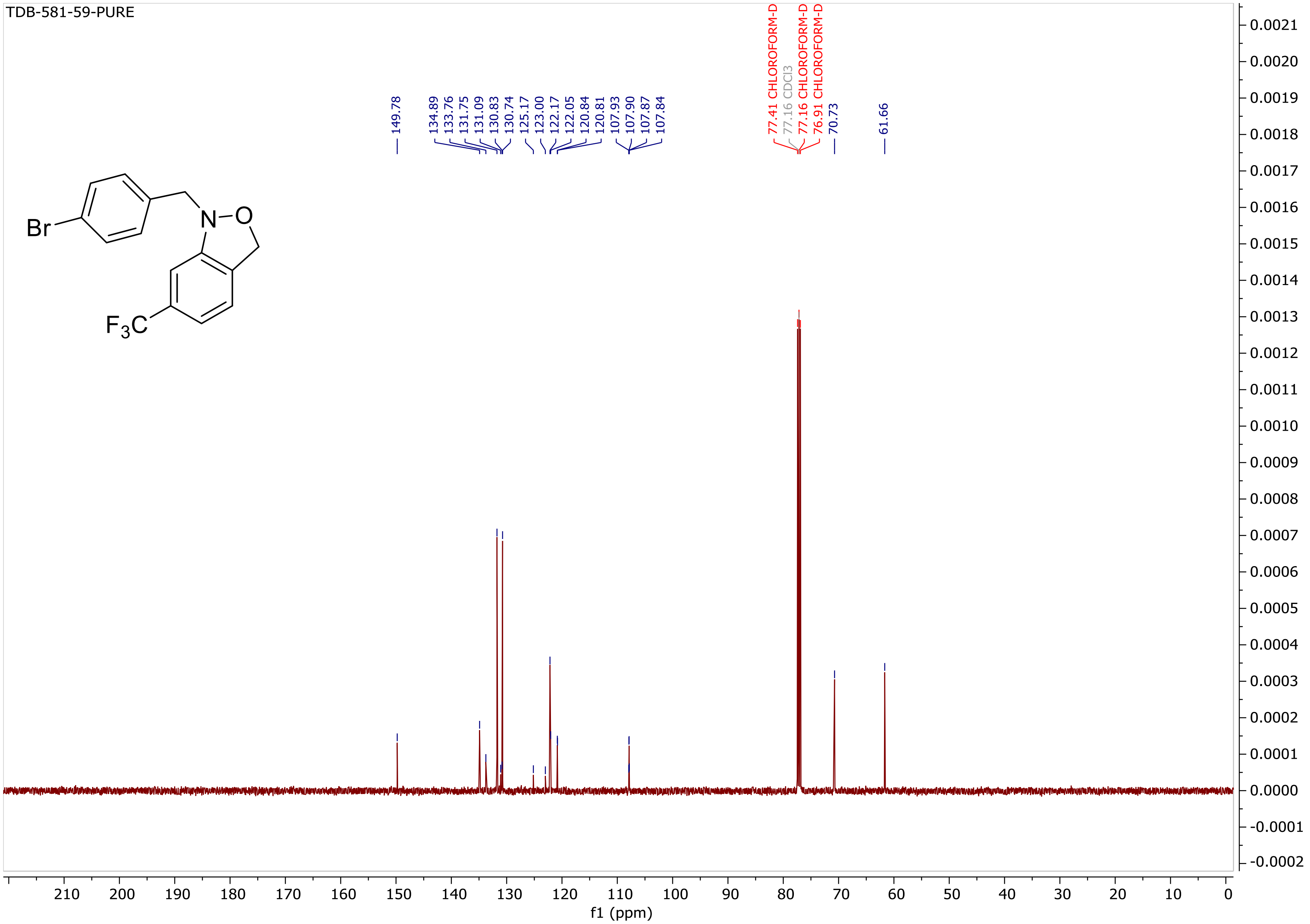

^{13}C NMR {^{19}F} (126 MHz, CDCl$_3$) spectrum of 1-(4-bromobenzyl)-6-(trifluoromethyl)-1,3-dihydrobenzo[*c*]isoxazole (**34**):

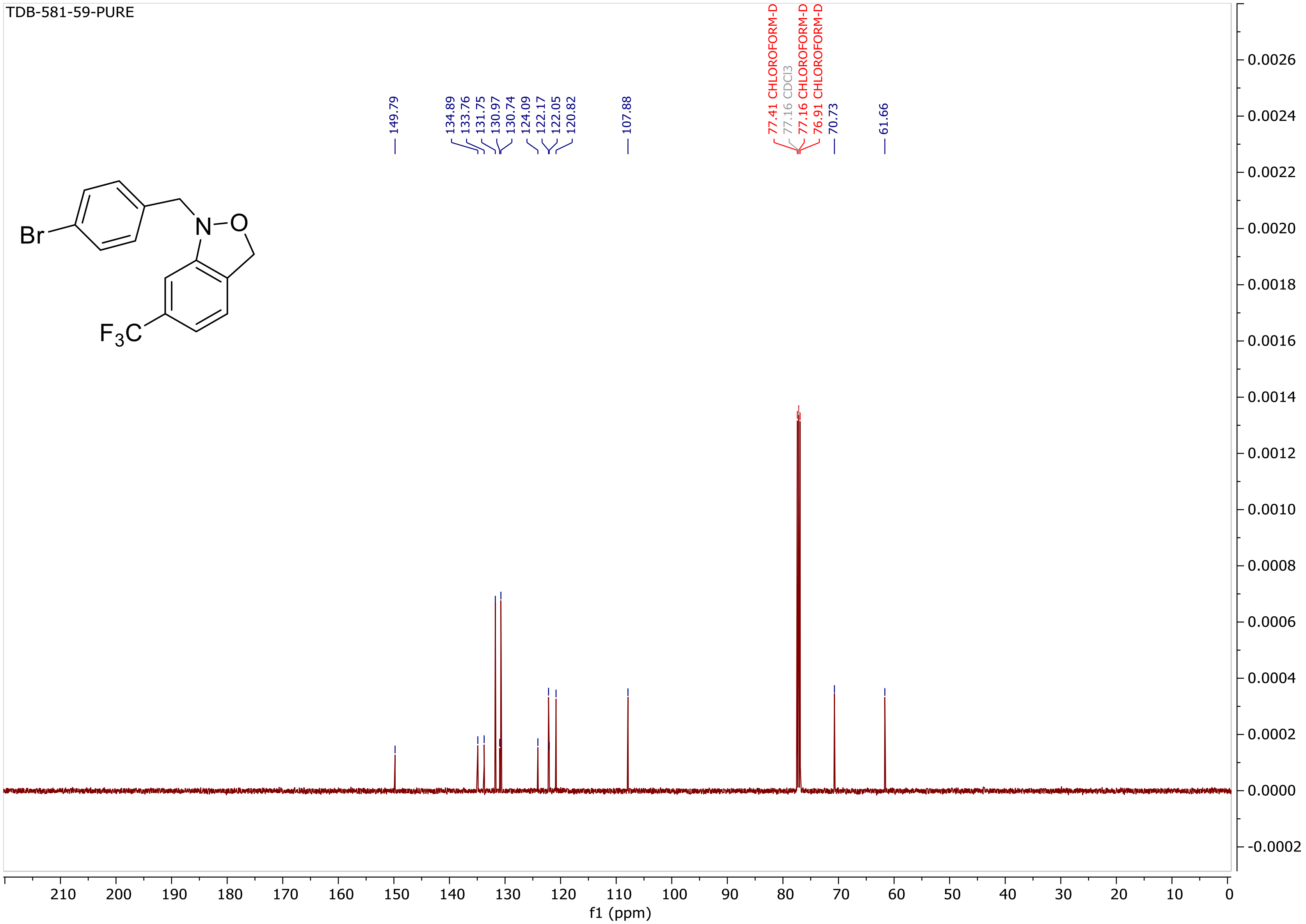

Expanded region of stacked (top) **^{13}C NMR (126 MHz, CDCl$_3$)** and (bottom) **^{13}C NMR {^{19}F} (126 MHz, CDCl$_3$)** spectrum of 1-(4-bromobenzyl)-6-(trifluoromethyl)-1,3-dihydrobenzo[*c*]isoxazole (**34**):

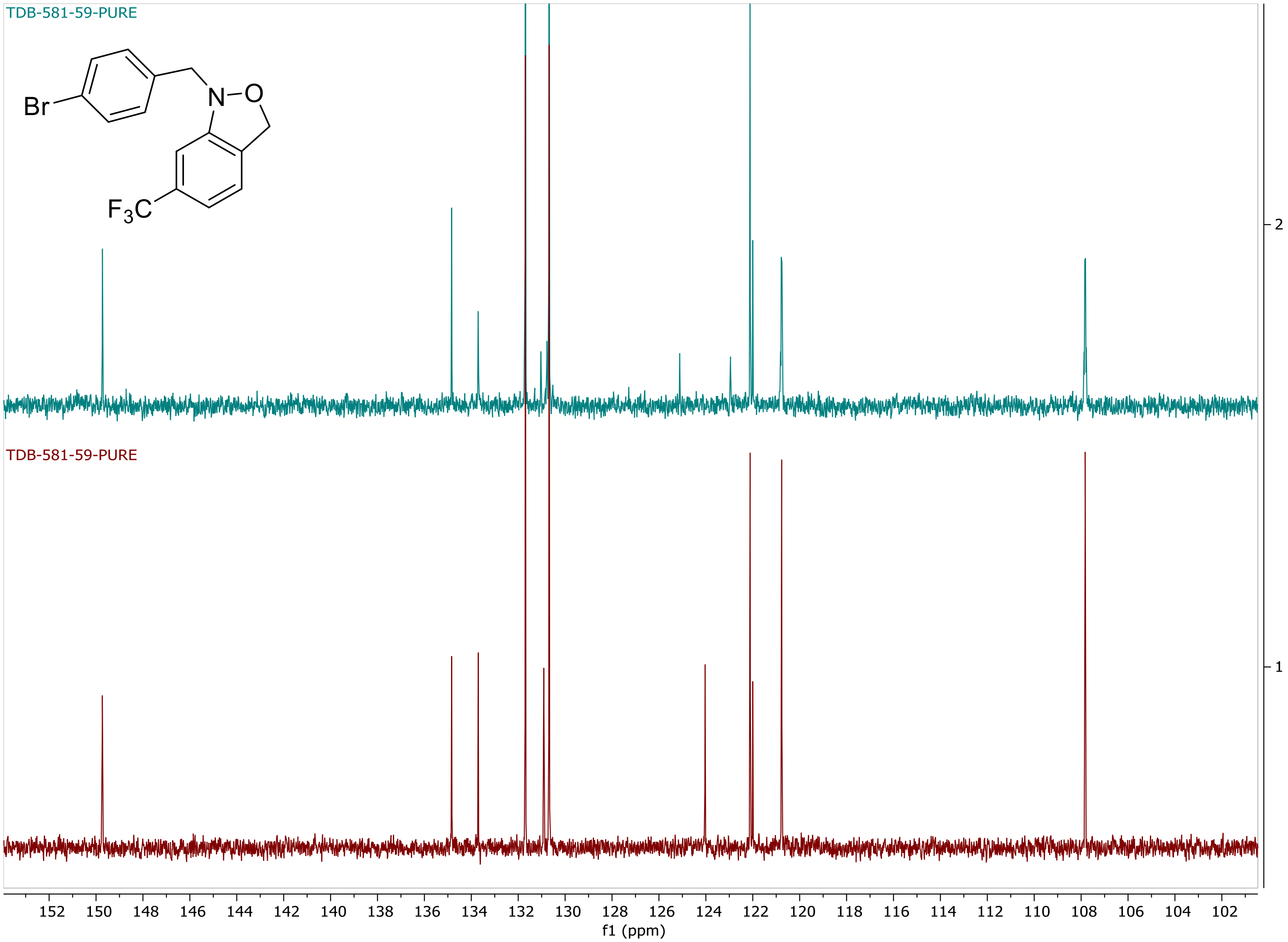

^{19}F NMR {1H} (470 MHz, $CDCl_3$) spectrum of 1-(4-bromobenzyl)-6-(trifluoromethyl)-1,3-dihydrobenzo[*c*]isoxazole (**34**):

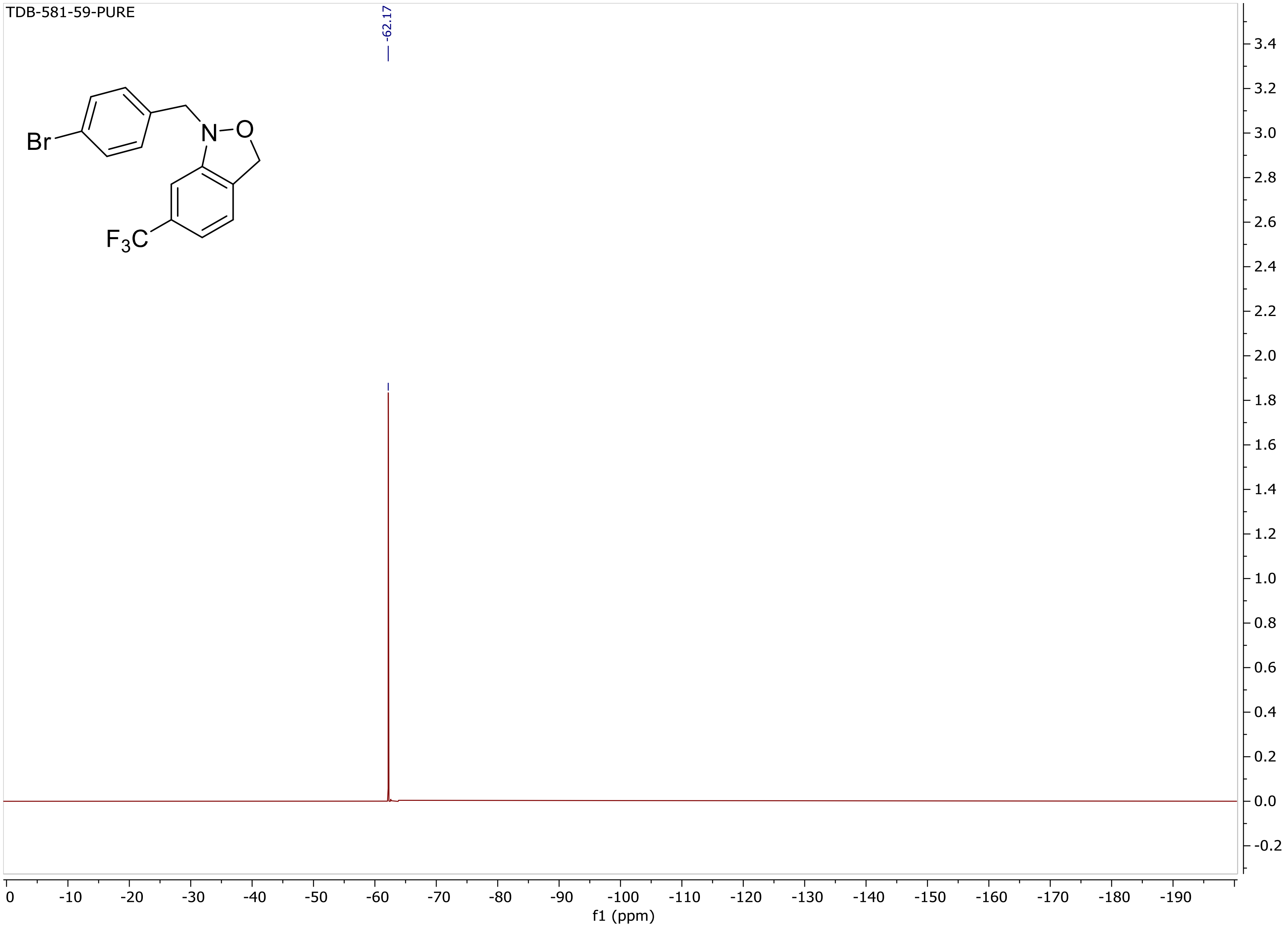

^{1}H NMR (500 MHz, CDCl₃) spectrum of 5-fluoro-1-hexyl-1,3-dihydrobenzo[c]isoxazole (**35**):

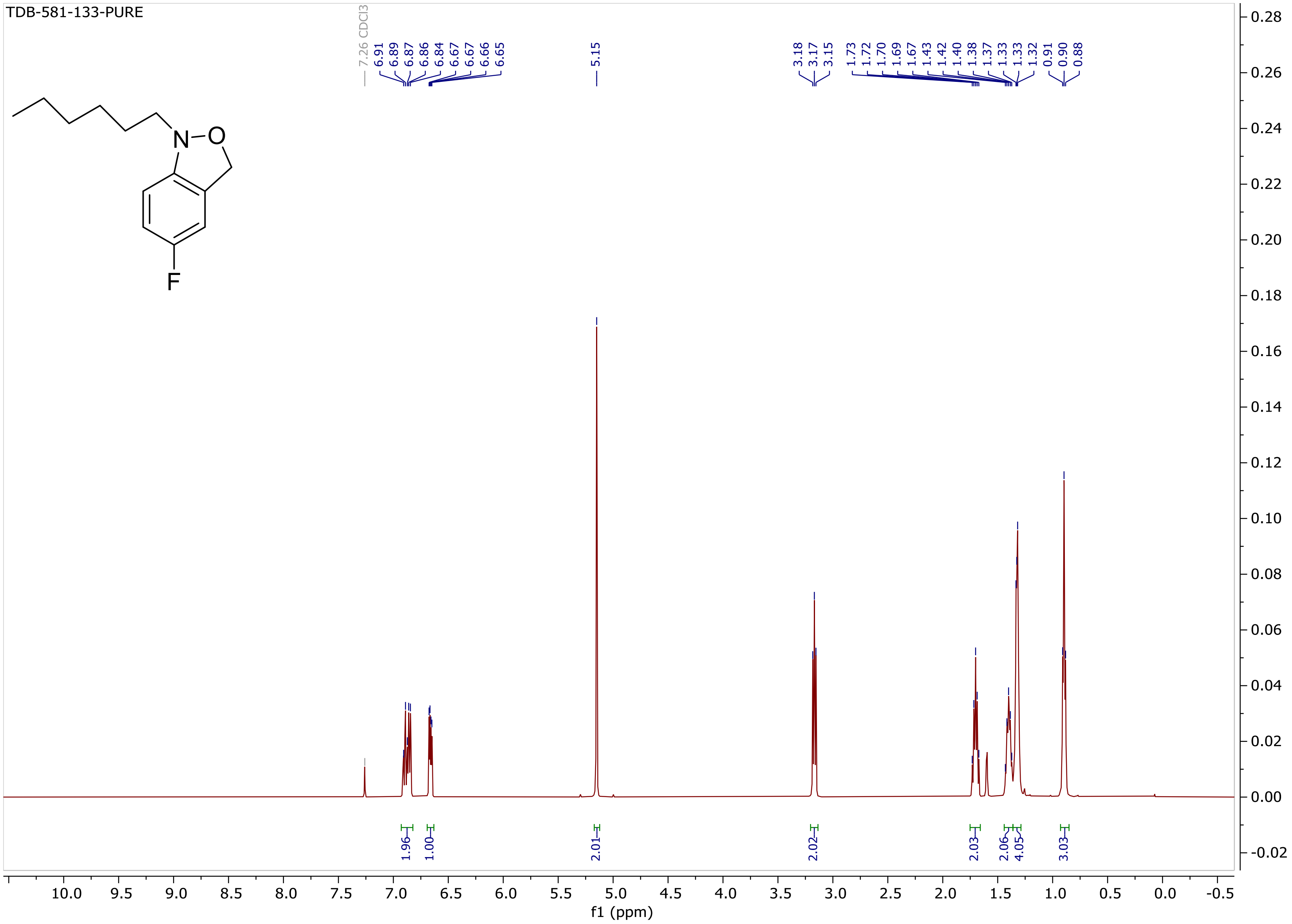

^{13}C NMR (126 MHz, CDCl$_3$) spectrum of 5-fluoro-1-hexyl-1,3-dihydrobenzo[c]isoxazole (**35**):

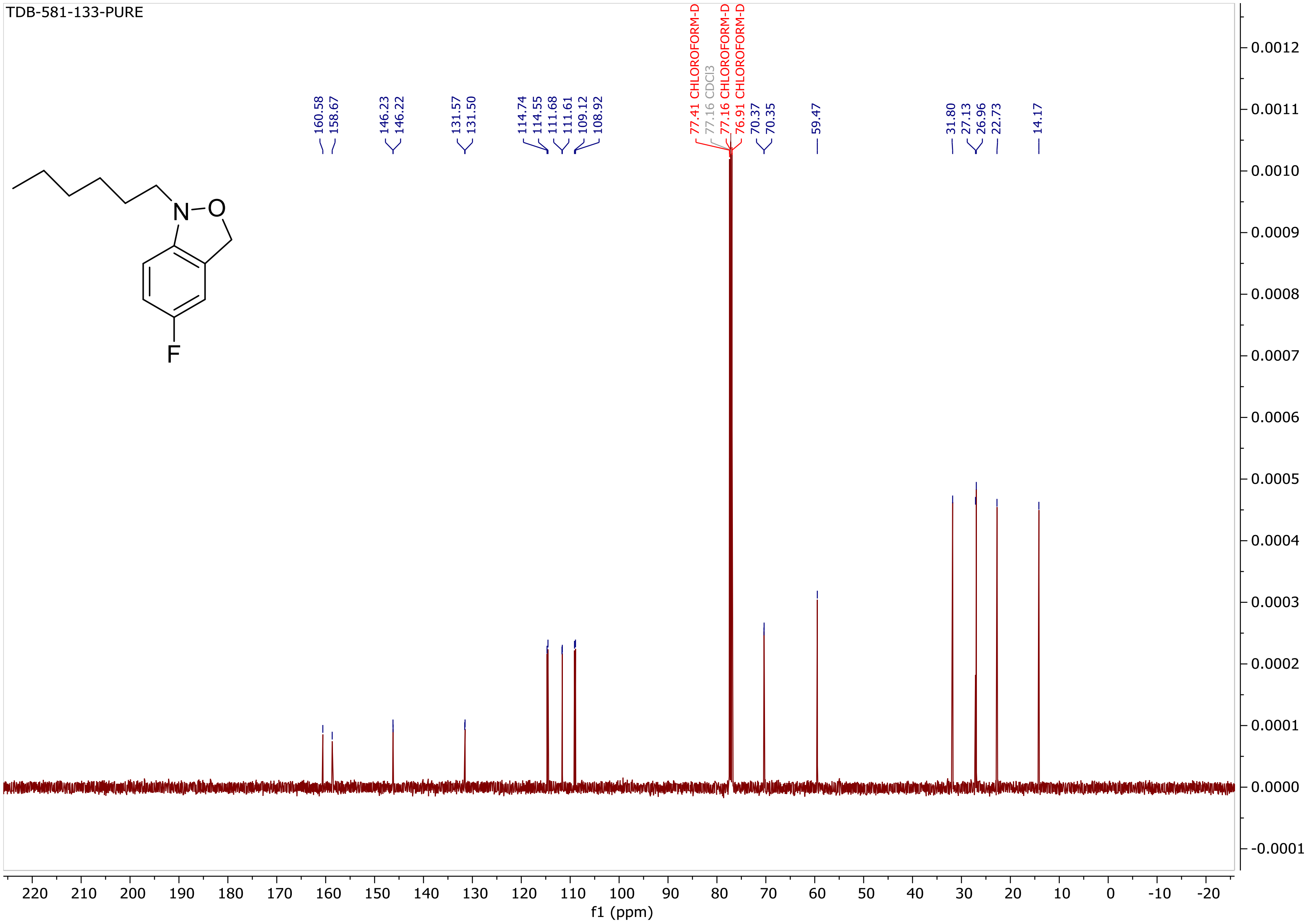

^{13}C NMR {^{19}F} (126 MHz, CDCl$_3$) spectrum of 5-fluoro-1-hexyl-1,3-dihydrobenzo[c]isoxazole (**35**):

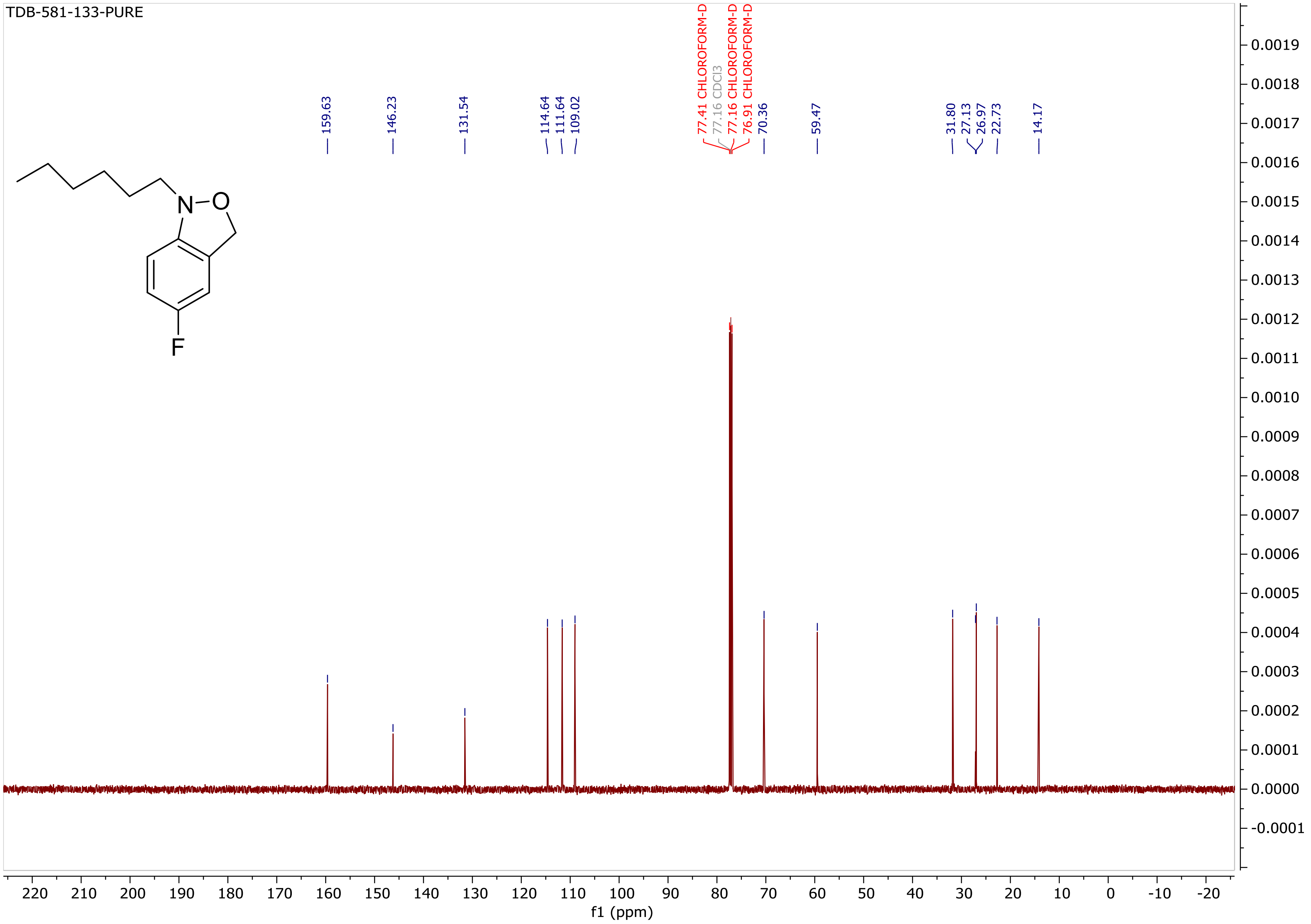

Expanded region of stacked (top) **^{13}C NMR (126 MHz, CDCl$_3$)** and (bottom) **^{13}C NMR {^{19}F} (126 MHz, CDCl$_3$)** spectrum of 5-fluoro-1-hexyl-1,3-dihydrobenzo[c]isoxazole (**35**):

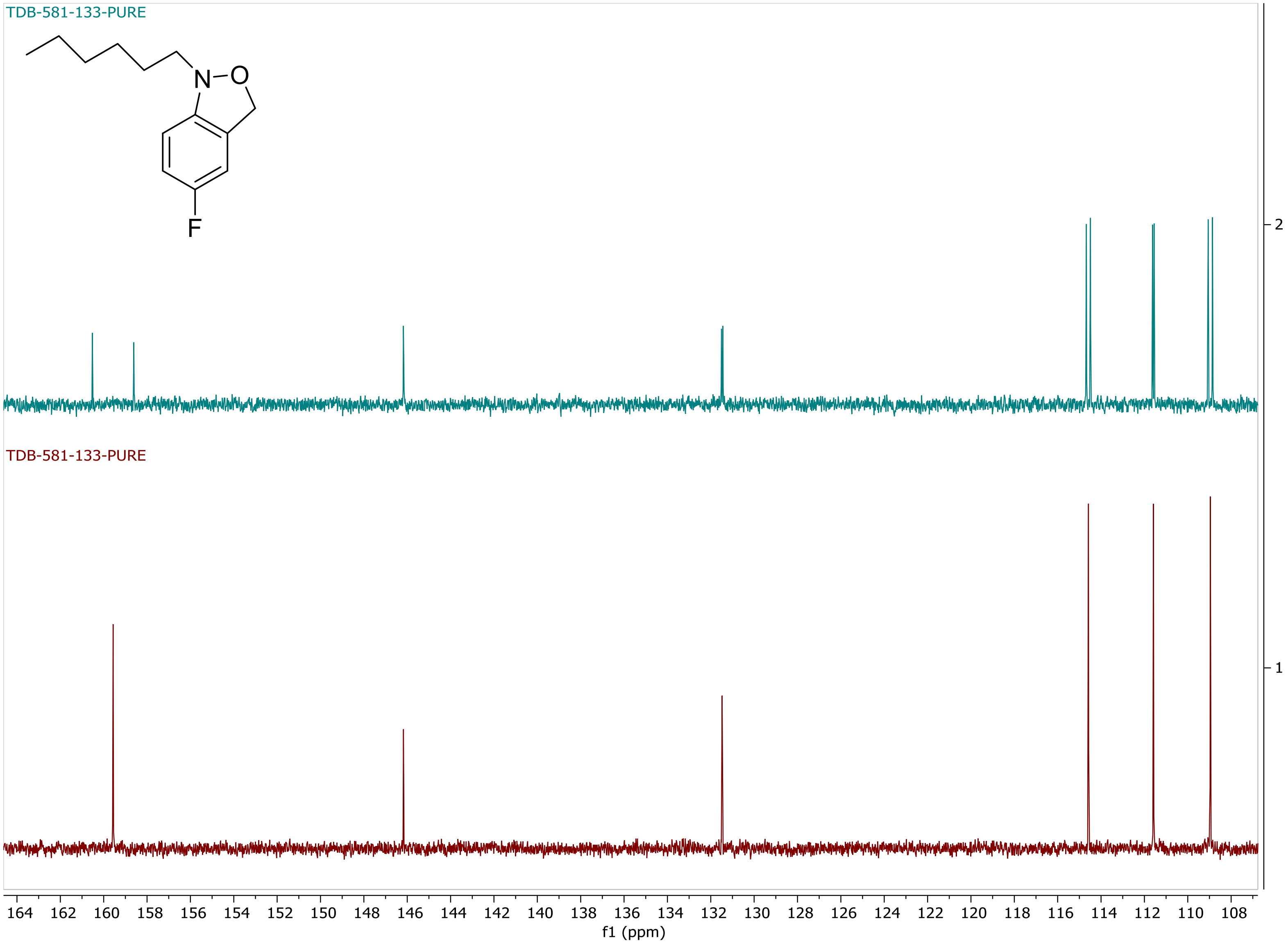

^{19}F NMR {1H} (470 MHz, CDCl$_3$) spectrum of 5-fluoro-1-hexyl-1,3-dihydrobenzo[c]isoxazole (**35**):

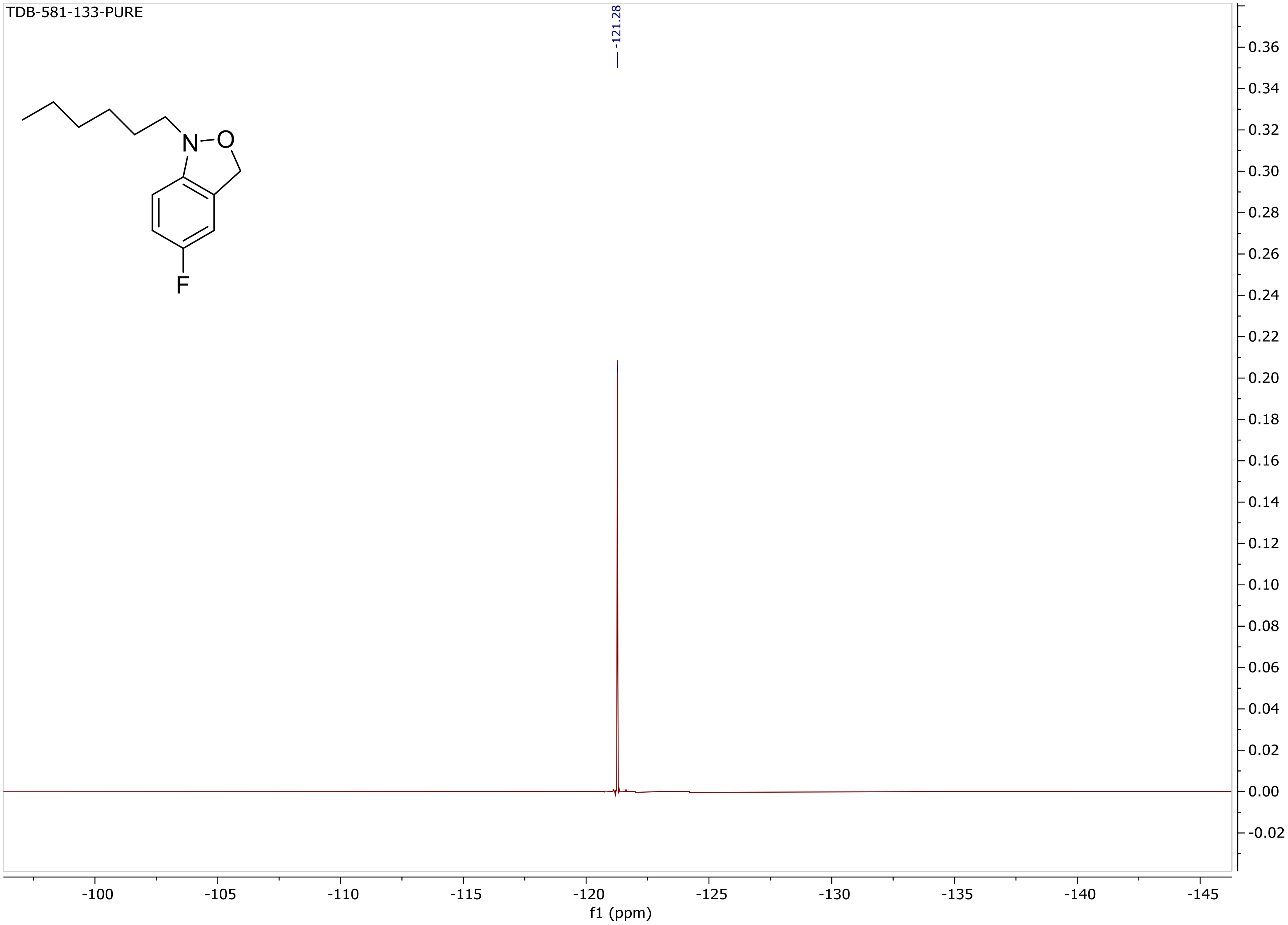

^{1}H NMR (500 MHz, CDCl$_3$) spectrum of 1-benzyl-6-bromo-1,3-dihydrobenzo[*c*]isoxazole (**36**):

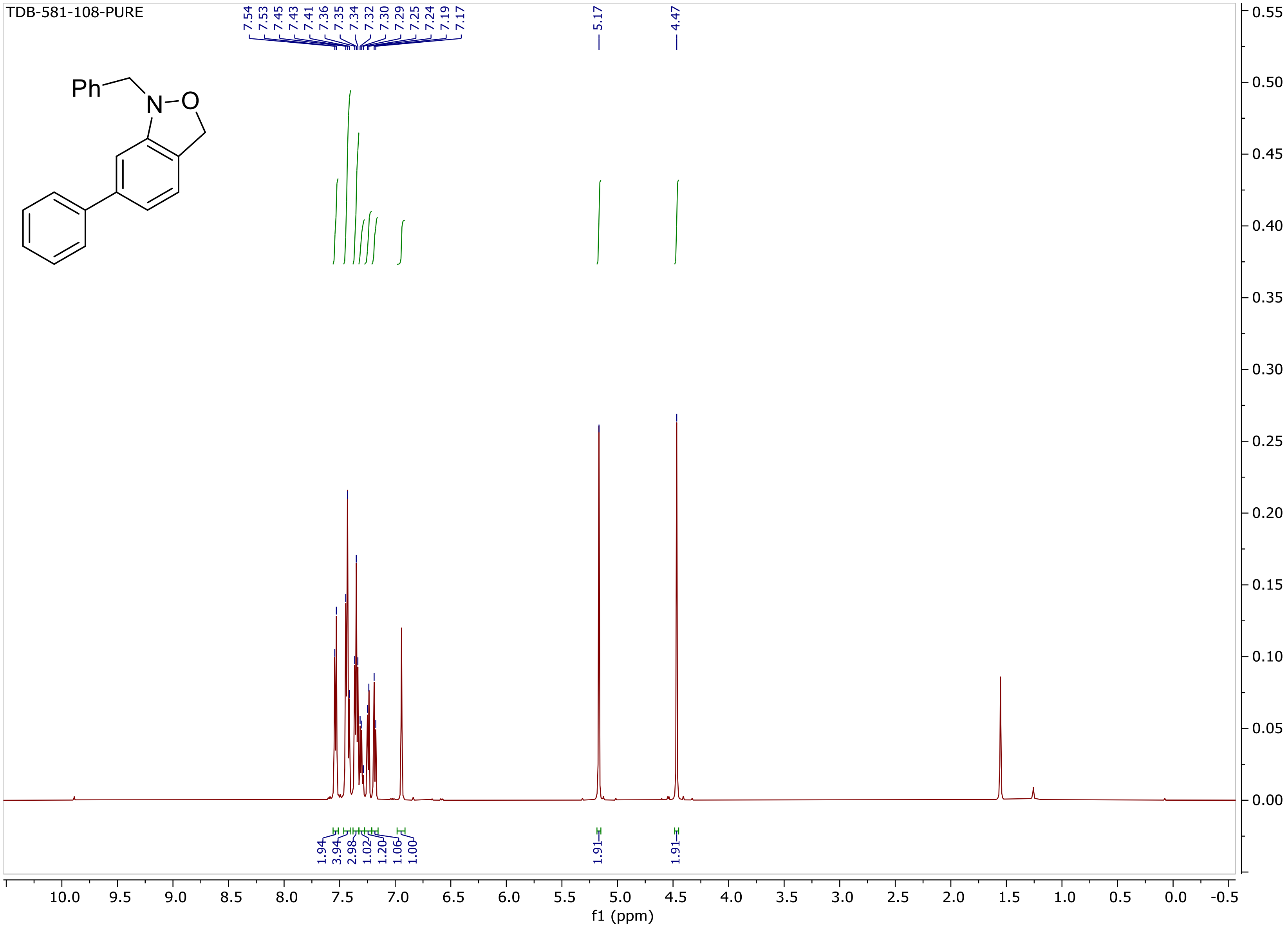

^{13}C NMR (126 MHz, $CDCl_3$) spectrum of 1-benzyl-6-bromo-1,3-dihydrobenzo[*c*]isoxazole (**36**):

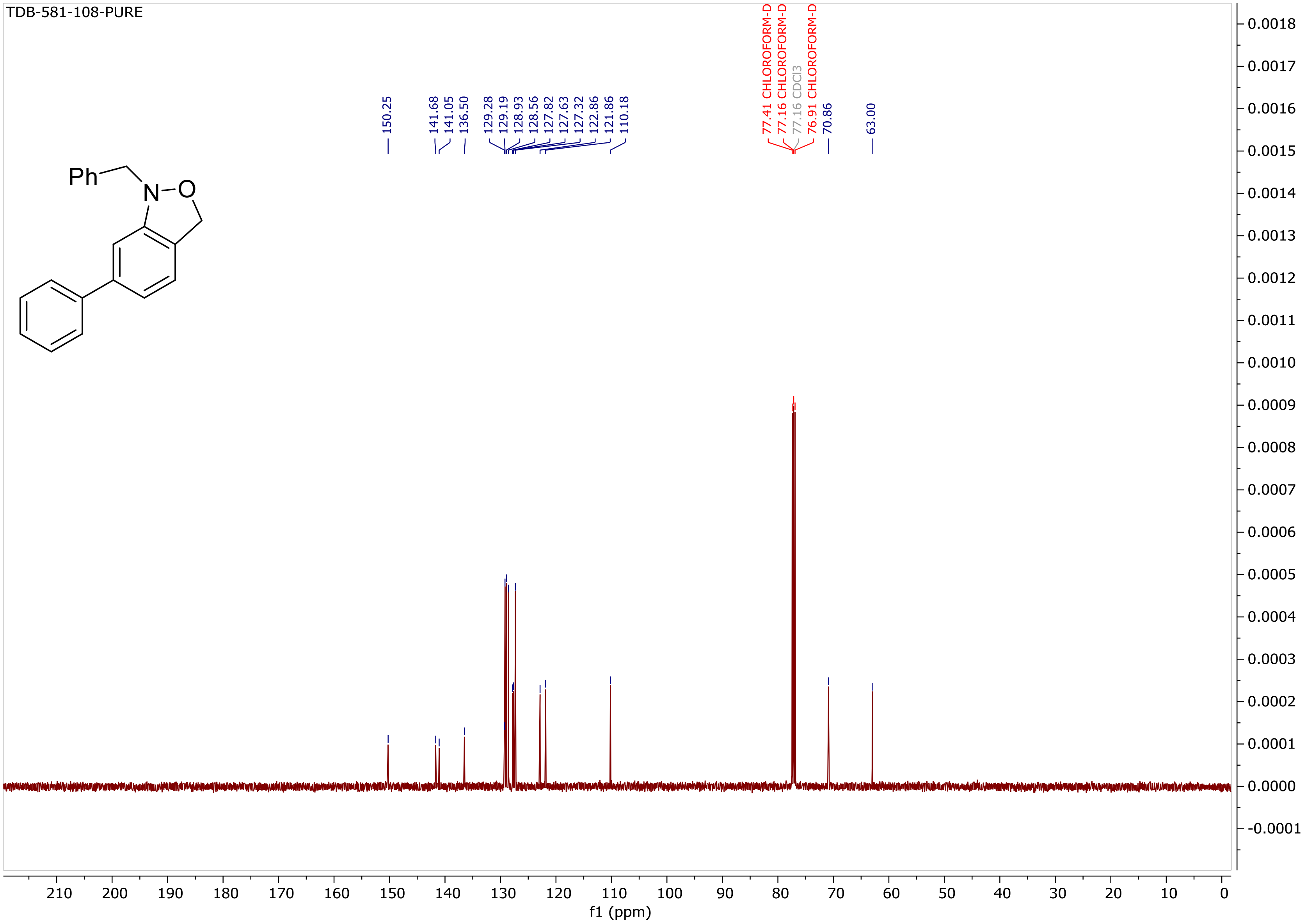

^{1}H NMR (500 MHz, CDCl₃) spectrum of 1-benzyl-6-morpholino-1,3-dihydrobenzo[*c*]isoxazole (**37**):

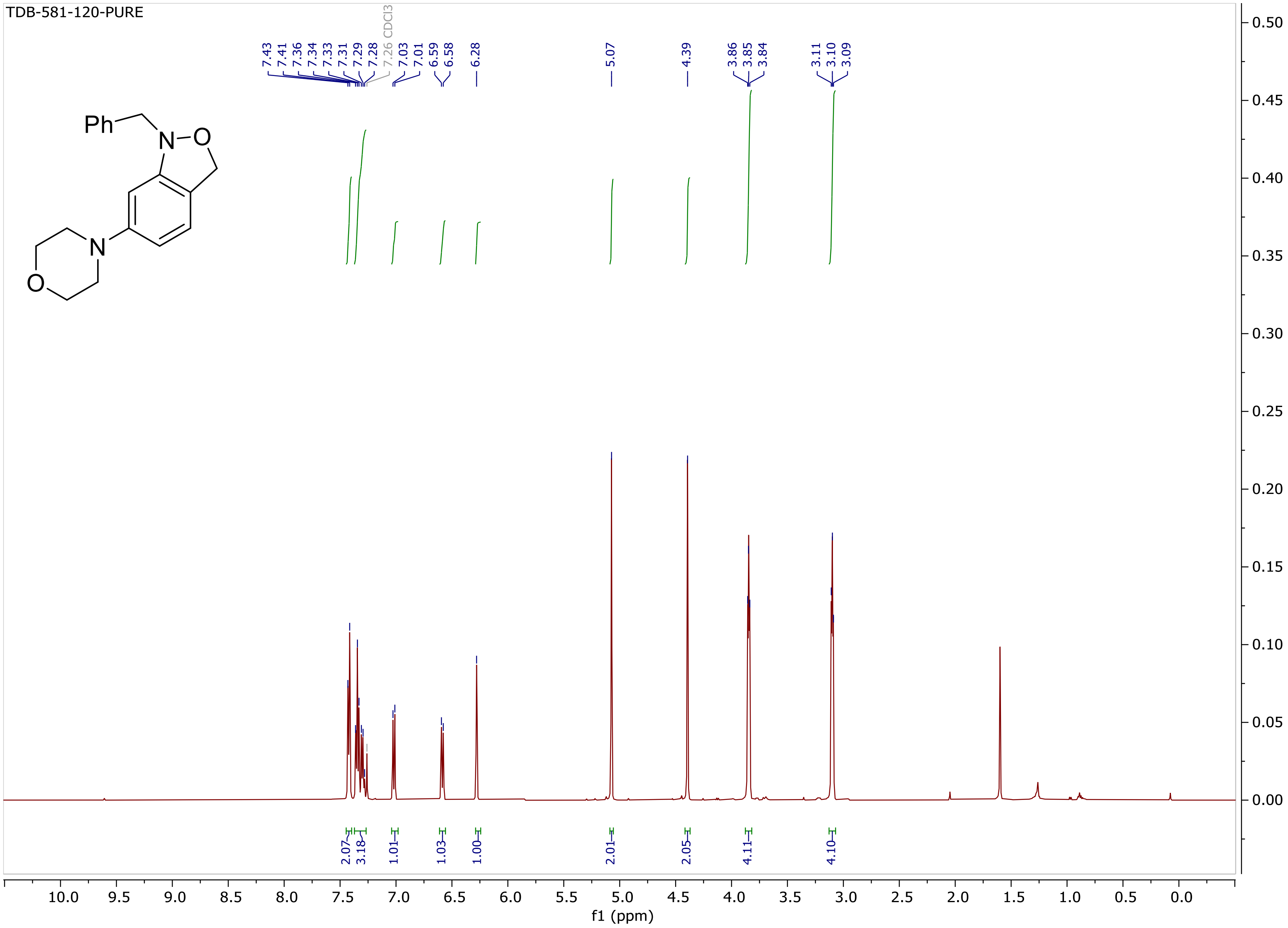

^{13}C NMR (126 MHz, $CDCl_3$) spectrum of 1-benzyl-6-morpholino-1,3-dihydrobenzo[*c*]isoxazole (**37**):

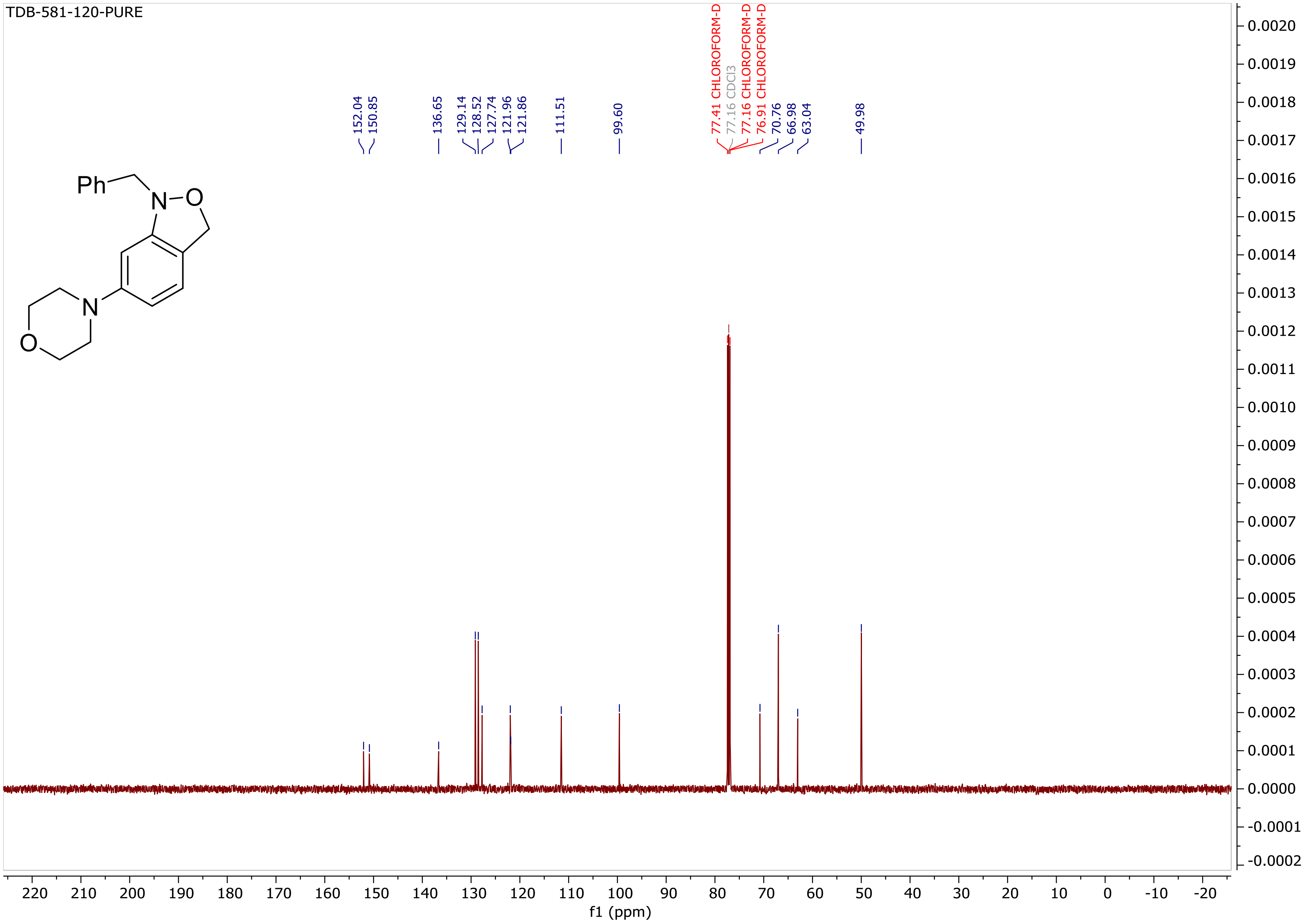

VT ¹H NMR (500 MHz, CDCl₃) spectrum of 1-benzylbenzo[*c*]isoxazol-3(*1H*)-one (**24**):

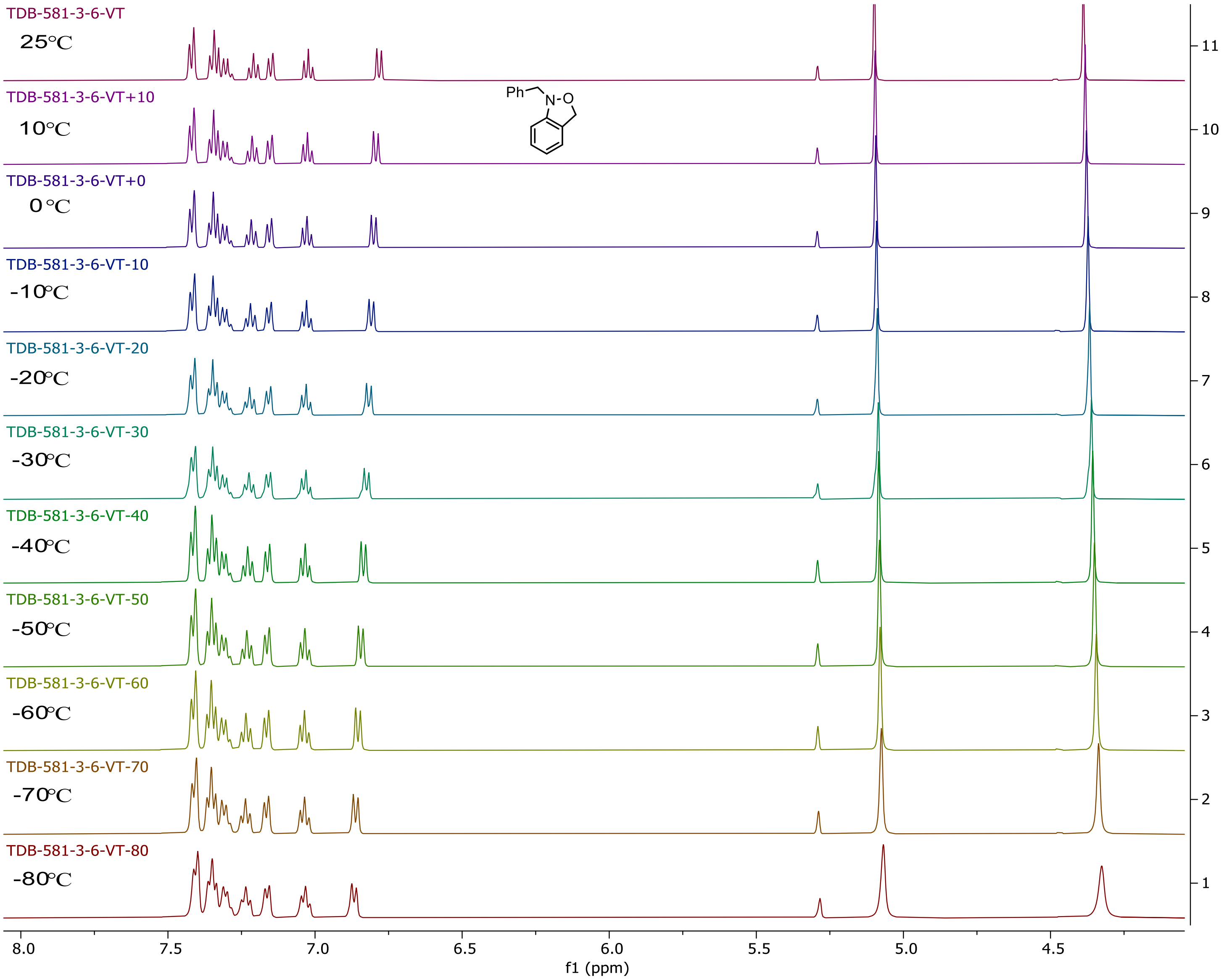

VT ^{13}C NMR (126 MHz, CDCl$_3$) spectrum of 1-benzylbenzo[*c*]isoxazol-3(*1H*)-one (**24**):

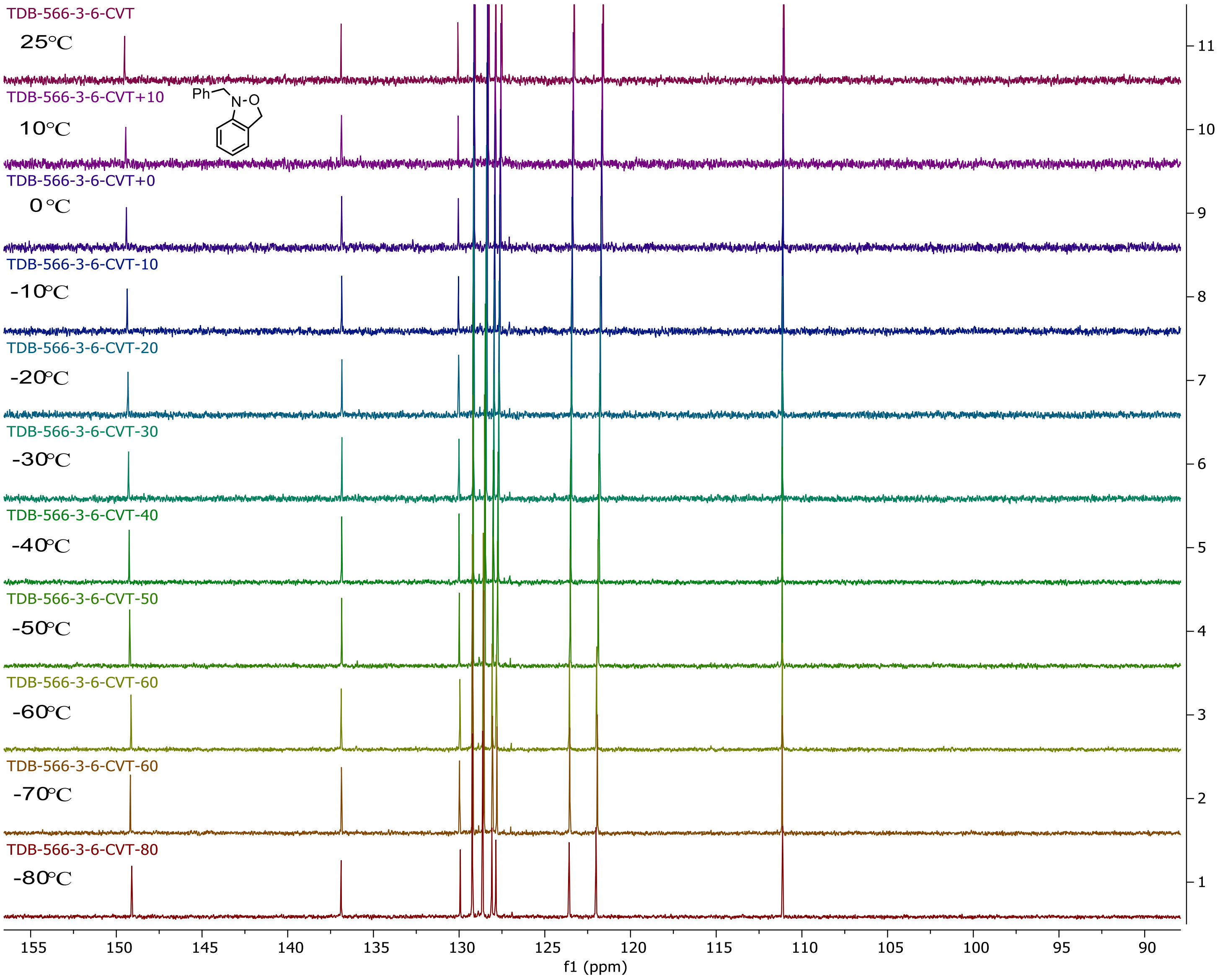

X-Ray Data of 15

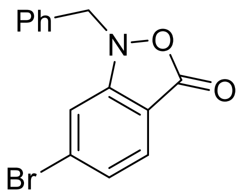

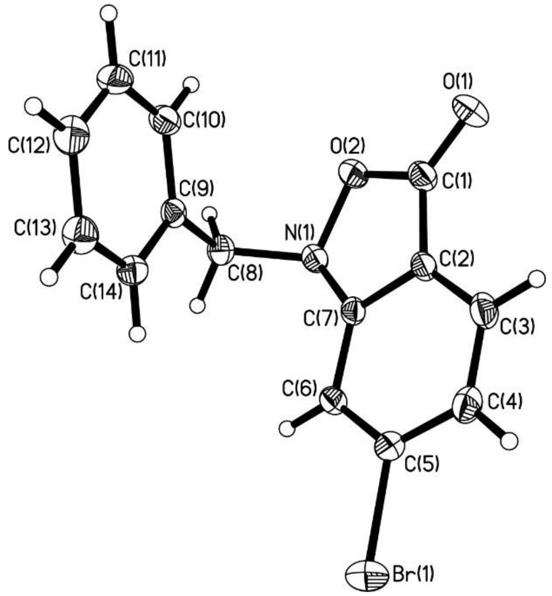

Figure S1. Ortep drawing of **15** with 30% probability for thermal ellipsoids.

Crystal Preparation: Title compound (**15**) was recrystallized in hot isopropanol with slow evaporation of the solvent.

Data Collection: A specimen of $C_{14}H_{10}BrNO_2$, approximate dimensions 0.050 mm x 0.180 mm x 0.320 mm in a colorless crystal was used for the X-ray crystallographic analysis. The X-ray intensity data were measured at room temperature (298K) on a Bruker D8 Quest PHOTON 100 CMOS X-ray diffractometer system with Incoatec Microfocus Source (IμS) monochromated Mo Kα radiation (λ= 0.71073 Å, sealed tube) using phi and omega-scan technique.

The integration of the data using a triclinic unit cell yielded a total of 31844 reflections to a maximum θ angle of 34.97° (0.62 Å resolution), of which 5406 were independent (average redundancy 5.890, completeness = 100.0%, R_{int} = 6.10%, R_{sig} = 4.30%) and 3153 (58.32%) were greater than 2σ(F^2). The final cell constants of a = 7.4984(3) Å, b = 7.6240(3) Å, c = 11.5715(4) Å, α = 104.6370(10)°, β = 98.5080(10)°, γ = 101.1160(10)°, volume = 614.42(4) $Å^3$, are based upon the refinement of the XYZ-centroids of 9915 reflections above 20 σ(I). The data were integrated with the manufacturer's SAINT software and corrected for absorption effects using the Multi-Scan method (SADABS). The calculated minimum and maximum transmission coefficients (based on crystal size) are 0.4400 and 0.7473.

The structure was solved and refined using the Bruker SHELXTL Software Package,[4] using the space group P-1 (No. 2), with Z = 2 for the formula unit, $C_{14}H_{10}BrNO_2$. Non-hydrogen atoms were located from successive difference Fourier map calculations. In the final cycles of each refinement, all the non-hydrogen atoms were refined in anisotropic displacement parameters. All the hydrogen atom positions were calculated and allowed to ride on the carbon to which they are bonded assuming a C–H bond length of m Å (m = 0.930 for Ph-H groups, m = 0.970 for CH_2 groups). Hydrogen atom temperature factors were fixed at n (n = 1.2 for Ph-H, CH_2 groups) times the isotropic temperature factors of the C atoms to which they are bonded. The final anisotropic full-matrix least-squares refinement on F^2 with 164 variables converged at R1 = 4.49%, for the observed data and wR2 = 12.49% for all data. The goodness-of-fit was 1.028. The largest peak in the final difference electron density synthesis was 0.866 $e^-/Å^3$ and the largest hole was -0.843 $e^-/Å^3$ with an RMS deviation of 0.076 $e^-/Å^3$. These largest residues are of no chemical significance. On the basis of the final model, the calculated density was 1.644 g/cm^3 and F(000),

304 e⁻. The asymmetric unit contains one molecule with the formula of $C_{14}H_{10}BrNO_2$. The efforts have been made to resolve as many alerts as possible generated by CheckCIF. The current highest alerts are at level G.

Table 1. Crystal data and structure refinement for j1_a.

Identification code	j1_a
Empirical formula	C14 H10 Br N O2
Formula weight	304.14
Temperature	298(2) K
Wavelength	0.71073 A
Crystal system, space group	Triclinic, P-1
Unit cell dimensions	a = 7.4984(3) A alpha = 104.6370(10) deg.
	b = 7.6240(3) A beta = 98.5080(10) deg.
	c = 11.5715(4) A gamma = 101.1160(10) deg.
Volume	614.42(4) A^3
Z, Calculated density	2, 1.644 Mg/m^3
Absorption coefficient	3.337 mm^-1
F(000)	304
Crystal size	0.320 x 0.180 x 0.050 mm
Theta range for data collection	1.859 to 34.971 deg.
Limiting indices	-12<=h<=12, -12<=k<=12, -18<=l<=18
Reflections collected / unique	31844 / 5406 [R(int) = 0.0610]
Completeness to theta = 25.242	100.0 %
Absorption correction	Semi-empirical from equivalents
Max. and min. transmission	0.7473 and 0.4400
Refinement method	Full-matrix least-squares on F^2
Data / restraints / parameters	5406 / 0 / 164
Goodness-of-fit on F^2	1.028

Final R indices [I>2sigma(I)]	R1 = 0.0449, wR2 = 0.1063
R indices (all data)	R1 = 0.0906, wR2 = 0.1249
Extinction coefficient	0.086(5)
Largest diff. peak and hole	0.866 and -0.843 e.A^-3

Table 2. Atomic coordinates (x 10^4) and equivalent isotropic displacement parameters (A^2 x 10^3) for j1_a.
U(eq) is defined as one third of the trace of the orthogonalized Uij tensor.

	x	y	z	U(eq)
Br(1)	1556(1)	8872(1)	6649(1)	66(1)
O(1)	7569(2)	5664(3)	2960(2)	55(1)
O(2)	4528(2)	4460(2)	2204(1)	40(1)
N(1)	2816(2)	4514(2)	2640(2)	33(1)
C(1)	6001(3)	5615(3)	3100(2)	37(1)
C(2)	5232(2)	6550(3)	4087(2)	32(1)
C(3)	6072(3)	7837(3)	5209(2)	40(1)
C(4)	4954(3)	8501(3)	5970(2)	43(1)
C(5)	3025(3)	7873(3)	5583(2)	37(1)
C(6)	2147(2)	6579(3)	4485(2)	34(1)
C(7)	3319(2)	5917(3)	3742(2)	29(1)
C(8)	1369(3)	4595(3)	1656(2)	34(1)
C(9)	1777(2)	6306(3)	1232(2)	30(1)
C(10)	2818(3)	6382(3)	339(2)	39(1)
C(11)	3157(3)	7939(3)	-69(2)	44(1)
C(12)	2476(3)	9456(3)	420(2)	44(1)
C(13)	1430(3)	9399(3)	1305(2)	44(1)
C(14)	1079(3)	7834(3)	1707(2)	36(1)

Table 3. Bond lengths [A] and angles [deg] for j1_a.

Br(1)-C(5)	1.8884(19)
O(1)-C(1)	1.205(2)
O(2)-C(1)	1.377(3)
O(2)-N(1)	1.451(2)
N(1)-C(7)	1.388(2)
N(1)-C(8)	1.474(2)
C(1)-C(2)	1.445(3)
C(2)-C(3)	1.386(3)
C(2)-C(7)	1.384(2)
C(3)-C(4)	1.375(3)
C(3)-H(3)	0.9300
C(4)-C(5)	1.398(3)
C(4)-H(4)	0.9300
C(5)-C(6)	1.375(3)
C(6)-C(7)	1.394(2)
C(6)-H(6)	0.9300
C(8)-C(9)	1.500(3)
C(8)-H(8A)	0.9700
C(8)-H(8B)	0.9700
C(9)-C(10)	1.390(3)
C(9)-C(14)	1.389(3)
C(10)-C(11)	1.378(3)
C(10)-H(10)	0.9300
C(11)-C(12)	1.378(3)
C(11)-H(11)	0.9300
C(12)-C(13)	1.383(3)
C(12)-H(12)	0.9300
C(13)-C(14)	1.380(3)
C(13)-H(13)	0.9300
C(14)-H(14)	0.9300
C(1)-O(2)-N(1)	109.26(14)
C(7)-N(1)-O(2)	105.23(14)
C(7)-N(1)-C(8)	119.88(15)
O(2)-N(1)-C(8)	109.50(15)
O(1)-C(1)-O(2)	120.0(2)
O(1)-C(1)-C(2)	133.0(2)
O(2)-C(1)-C(2)	106.98(15)
C(3)-C(2)-C(7)	121.28(18)
C(3)-C(2)-C(1)	131.56(17)
C(7)-C(2)-C(1)	107.16(17)
C(4)-C(3)-C(2)	118.28(18)
C(4)-C(3)-H(3)	120.9
C(2)-C(3)-H(3)	120.9
C(3)-C(4)-C(5)	119.16(19)
C(3)-C(4)-H(4)	120.4
C(5)-C(4)-H(4)	120.4
C(6)-C(5)-C(4)	124.07(18)
C(6)-C(5)-Br(1)	118.56(15)
C(4)-C(5)-Br(1)	117.37(16)

C(7)-C(6)-C(5)	115.33(17)
C(7)-C(6)-H(6)	122.3
C(5)-C(6)-H(6)	122.3
C(6)-C(7)-N(1)	127.30(16)
C(6)-C(7)-C(2)	121.85(18)
N(1)-C(7)-C(2)	110.72(16)
N(1)-C(8)-C(9)	114.94(15)
N(1)-C(8)-H(8A)	108.5
C(9)-C(8)-H(8A)	108.5
N(1)-C(8)-H(8B)	108.5
C(9)-C(8)-H(8B)	108.5
H(8A)-C(8)-H(8B)	107.5
C(10)-C(9)-C(14)	118.65(18)
C(10)-C(9)-C(8)	120.99(17)
C(14)-C(9)-C(8)	120.35(17)
C(11)-C(10)-C(9)	120.89(19)
C(11)-C(10)-H(10)	119.6
C(9)-C(10)-H(10)	119.6
C(10)-C(11)-C(12)	119.9(2)
C(10)-C(11)-H(11)	120.0
C(12)-C(11)-H(11)	120.0
C(11)-C(12)-C(13)	119.9(2)
C(11)-C(12)-H(12)	120.1
C(13)-C(12)-H(12)	120.1
C(14)-C(13)-C(12)	120.2(2)
C(14)-C(13)-H(13)	119.9
C(12)-C(13)-H(13)	119.9
C(13)-C(14)-C(9)	120.47(19)
C(13)-C(14)-H(14)	119.8
C(9)-C(14)-H(14)	119.8

Symmetry transformations used to generate equivalent atoms:

Table 4. Anisotropic displacement parameters (A^2 x 10^3) for j1_a.
The anisotropic displacement factor exponent takes the form:
-2 pi^2 [h^2 a*^2 U11 + ... + 2 h k a* b* U12]

	U11	U22	U33	U23	U13	U12
Br(1)	66(1)	90(1)	47(1)	8(1)	24(1)	36(1)
O(1)	34(1)	76(1)	64(1)	23(1)	20(1)	23(1)
O(2)	37(1)	47(1)	40(1)	10(1)	12(1)	21(1)
N(1)	29(1)	37(1)	36(1)	11(1)	6(1)	11(1)
C(1)	32(1)	44(1)	44(1)	19(1)	11(1)	15(1)
C(2)	24(1)	39(1)	36(1)	16(1)	6(1)	10(1)
C(3)	27(1)	48(1)	43(1)	15(1)	0(1)	6(1)
C(4)	41(1)	49(1)	32(1)	8(1)	-2(1)	9(1)
C(5)	38(1)	46(1)	31(1)	13(1)	9(1)	15(1)
C(6)	26(1)	42(1)	36(1)	15(1)	8(1)	10(1)
C(7)	27(1)	32(1)	30(1)	14(1)	4(1)	8(1)
C(8)	32(1)	32(1)	34(1)	7(1)	2(1)	5(1)
C(9)	24(1)	31(1)	27(1)	4(1)	-1(1)	4(1)
C(10)	34(1)	44(1)	39(1)	9(1)	10(1)	12(1)
C(11)	34(1)	57(1)	44(1)	19(1)	14(1)	6(1)
C(12)	39(1)	40(1)	50(1)	19(1)	5(1)	1(1)
C(13)	49(1)	34(1)	46(1)	8(1)	8(1)	14(1)
C(14)	38(1)	37(1)	32(1)	9(1)	8(1)	11(1)

Table 5. Hydrogen coordinates (x 10^4) and isotropic displacement parameters (A^2 x 10^3) for j1_a.

	x	y	z	U(eq)
H(3)	7359	8242	5441	48
H(4)	5474	9356	6732	51
H(6)	860	6174	4254	40
H(8A)	210	4529	1939	41
H(8B)	1192	3504	964	41
H(10)	3292	5368	12	46
H(11)	3844	7967	-673	53
H(12)	2721	10516	156	53
H(13)	961	10417	1630	52
H(14)	370	7801	2299	43

Table 6. Torsion angles [deg] for j1_a.

C(1)-O(2)-N(1)-C(7)	7.81(19)
C(1)-O(2)-N(1)-C(8)	137.92(16)
N(1)-O(2)-C(1)-O(1)	174.25(19)
N(1)-O(2)-C(1)-C(2)	-4.8(2)
O(1)-C(1)-C(2)-C(3)	0.6(4)
O(2)-C(1)-C(2)-C(3)	179.4(2)
O(1)-C(1)-C(2)-C(7)	-178.9(2)
O(2)-C(1)-C(2)-C(7)	-0.1(2)
C(7)-C(2)-C(3)-C(4)	-1.0(3)
C(1)-C(2)-C(3)-C(4)	179.6(2)
C(2)-C(3)-C(4)-C(5)	-0.7(3)
C(3)-C(4)-C(5)-C(6)	1.6(3)
C(3)-C(4)-C(5)-Br(1)	-178.65(17)
C(4)-C(5)-C(6)-C(7)	-0.8(3)
Br(1)-C(5)-C(6)-C(7)	179.47(14)
C(5)-C(6)-C(7)-N(1)	174.61(18)
C(5)-C(6)-C(7)-C(2)	-0.9(3)
O(2)-N(1)-C(7)-C(6)	176.13(17)
C(8)-N(1)-C(7)-C(6)	52.4(3)
O(2)-N(1)-C(7)-C(2)	-7.9(2)
C(8)-N(1)-C(7)-C(2)	-131.68(17)
C(3)-C(2)-C(7)-C(6)	1.8(3)
C(1)-C(2)-C(7)-C(6)	-178.59(17)
C(3)-C(2)-C(7)-N(1)	-174.34(18)
C(1)-C(2)-C(7)-N(1)	5.2(2)
C(7)-N(1)-C(8)-C(9)	58.9(2)
O(2)-N(1)-C(8)-C(9)	-62.8(2)
N(1)-C(8)-C(9)-C(10)	86.0(2)
N(1)-C(8)-C(9)-C(14)	-95.6(2)
C(14)-C(9)-C(10)-C(11)	0.1(3)
C(8)-C(9)-C(10)-C(11)	178.57(19)
C(9)-C(10)-C(11)-C(12)	0.7(3)
C(10)-C(11)-C(12)-C(13)	-1.0(3)
C(11)-C(12)-C(13)-C(14)	0.6(3)
C(12)-C(13)-C(14)-C(9)	0.3(3)
C(10)-C(9)-C(14)-C(13)	-0.6(3)
C(8)-C(9)-C(14)-C(13)	-179.06(19)

Symmetry transformations used to generate equivalent atoms:

X-Ray Data of 31

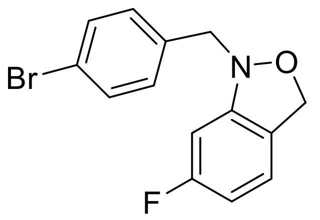

Figure S2. Ortep drawing of **31** with 30% probability for thermal ellipsoids.

Crystal Preparation: Title compound (**31**) was recrystallized in hot isopropanol with slow evaporation of the solvent.

Data Collection: A specimen of $C_{14}H_{11}BrFNO$, approximate dimensions 0.140 mm x 0.260 mm x 0.340 mm in a colorless crystal was used for the X-ray crystallographic analysis. The X-ray intensity data were measured at room temperature (299K) on a Bruker D8 Quest PHOTON 100 CMOS X-ray diffractometer system with Incoatec Microfocus Source (IμS) monochromated Mo Kα radiation (λ= 0.71073 Å, sealed tube) using phi and omega-scan technique.

The integration of the data using a monoclinic unit cell yielded a total of 31932 reflections to a maximum θ angle of 25.45° (0.83 Å resolution), of which 2336 were independent (average redundancy 13.670, completeness = 99.6%, R_{int} = 6.06%, R_{sig} = 2.19%) and 1781 (76.24%) were greater than $2\sigma(F^2)$. The final cell constants of a = 8.2458(3) Å, b = 14.1812(4) Å, c = 10.9373(4) Å, β = 98.171(2)°, volume = 1265.97(7) $Å^3$, are based upon the refinement of the XYZ-centroids of 9912 reflections above 20 σ(I). The data were integrated with the manufacturer's SAINT software and corrected for absorption effects using the Multi-Scan method (SADABS). The calculated minimum and maximum transmission coefficients (based on crystal size) are 0.4607 and 0.7452.

The structure was solved and refined using the Bruker SHELXTL Software Package[4], using the

space group $P2_1/c$ (No. 14), with Z = 4 for the formula unit, $C_{14}H_{11}BrFNO$. Non-hydrogen atoms were located from successive difference Fourier map calculations. In the final cycles of each refinement, all the non-hydrogen atoms were refined in anisotropic displacement parameters with proper constraints on some atoms of two phenyl rings. All the hydrogen atom positions were calculated and allowed to ride on the carbon to which they are bonded assuming a C–H bond length of m Å (m = 0.930 for Ph-H groups, m = 0.970 for CH_2 groups). Hydrogen atom temperature factors were fixed at n (n = 1.2 for Ph-H, CH_2 groups) times the isotropic temperature factors of the C atoms to which they are bonded. The final anisotropic full-matrix least-squares refinement on F^2 with 139 variables converged at R1 = 4.51%, for the observed data and wR2 = 12.18% for all data. The goodness-of-fit was 1.020. The largest peak in the final difference electron density synthesis was 0.495 $e^-/Å^3$ and the largest hole was -0.545 $e^-/Å^3$ with an RMS deviation of 0.067 $e^-/Å^3$. These largest residues are of no chemical significance. On the basis of the final model, the calculated density was 1.617 g/cm^3 and F(000), 616 e^-. The asymmetric unit contains one molecule with the formula of $C_{14}H_{11}BrFNO$. The efforts have been made to resolve as many alerts as possible generated by CheckCIF. The current highest alerts are at level C, which might be attributed to a little bit high thermal motions of some atoms in the molecule.

Table 1. Crystal data and structure refinement for j3_a.

Identification code	j3_a
Empirical formula	C14 H11 Br F N O
Formula weight	308.15
Temperature	299(2) K
Wavelength	0.71073 A
Crystal system, space group	Monoclinic, P2(1)/c

Unit cell dimensions	a = 8.2458(3) A alpha = 90 deg.
	b = 14.1812(4) A beta = 98.171(2) deg.
	c = 10.9373(4) A gamma = 90 deg.
Volume	1265.97(7) A^3
Z, Calculated density	4, 1.617 Mg/m^3
Absorption coefficient	3.245 mm^-1
F(000)	616
Crystal size	0.340 x 0.260 x 0.140 mm
Theta range for data collection	2.367 to 25.445 deg.
Limiting indices	-9<=h<=9, -17<=k<=17, -13<=l<=13
Reflections collected / unique	31932 / 2336 [R(int) = 0.0606]
Completeness to theta = 25.242	99.6 %
Absorption correction	Semi-empirical from equivalents
Max. and min. transmission	0.7452 and 0.4607
Refinement method	Full-matrix least-squares on F^2
Data / restraints / parameters	2336 / 0 / 139
Goodness-of-fit on F^2	1.020
Final R indices [I>2sigma(I)]	R1 = 0.0451, wR2 = 0.1083
R indices (all data)	R1 = 0.0650, wR2 = 0.1218
Extinction coefficient	n/a
Largest diff. peak and hole	0.495 and -0.545 e.A^-3

Table 2. Atomic coordinates (x 10^4) and equivalent isotropic displacement parameters (A^2 x 10^3) for j3_a.
U(eq) is defined as one third of the trace of the orthogonalized Uij tensor.

	x	y	z	U(eq)
Br(1)	1726(1)	7602(1)	4534(1)	72(1)
F(1)	1920(4)	-252(2)	5062(3)	82(1)
N(1)	3311(4)	2875(2)	3832(3)	53(1)
O(1)	4120(5)	3141(2)	2769(4)	92(1)
C(1)	5007(7)	2352(3)	2417(5)	67(1)
C(2)	4329(3)	1542(2)	3017(2)	52(1)
C(3)	4537(3)	573(2)	2921(2)	59(1)
C(4)	3725(4)	-38(1)	3622(3)	59(1)
C(5)	2705(3)	320(2)	4419(2)	56(1)
C(6)	2497(3)	1289(2)	4514(2)	52(1)
C(7)	3309(3)	1900(1)	3813(2)	46(1)
C(8)	1694(5)	3318(3)	3605(5)	60(1)
C(9)	1777(4)	4366(1)	3802(3)	51(1)
C(10)	2709(5)	4745(2)	4845(3)	99(2)
C(11)	2702(5)	5712(2)	5053(3)	98(2)
C(12)	1762(4)	6300(1)	4217(3)	53(1)
C(13)	830(4)	5922(2)	3173(3)	100(2)
C(14)	838(4)	4955(2)	2966(3)	90(2)

Table 3. Bond lengths [A] and angles [deg] for j3_a.

Br(1)-C(12)	1.880(2)
F(1)-C(5)	1.305(3)
N(1)-C(7)	1.384(4)
N(1)-O(1)	1.469(5)
N(1)-C(8)	1.462(5)
O(1)-C(1)	1.419(6)
C(1)-C(2)	1.473(5)
C(1)-H(1A)	0.9700
C(1)-H(1B)	0.9700
C(2)-C(3)	1.3900
C(2)-C(7)	1.3900
C(3)-C(4)	1.3900
C(3)-H(3)	0.9300
C(4)-C(5)	1.3900
C(4)-H(4)	0.9300
C(5)-C(6)	1.3900
C(6)-C(7)	1.3900
C(6)-H(6)	0.9300
C(8)-C(9)	1.503(5)
C(8)-H(8A)	0.9700
C(8)-H(8B)	0.9700
C(9)-C(10)	1.3900
C(9)-C(14)	1.3900
C(10)-C(11)	1.3900
C(10)-H(10)	0.9300
C(11)-C(12)	1.3900
C(11)-H(11)	0.9300
C(12)-C(13)	1.3900
C(13)-C(14)	1.3900
C(13)-H(13)	0.9300
C(14)-H(14)	0.9300
C(7)-N(1)-O(1)	104.1(3)
C(7)-N(1)-C(8)	115.3(3)
O(1)-N(1)-C(8)	105.2(3)
C(1)-O(1)-N(1)	108.8(3)
O(1)-C(1)-C(2)	104.6(3)
O(1)-C(1)-H(1A)	110.8
C(2)-C(1)-H(1A)	110.8
O(1)-C(1)-H(1B)	110.8
C(2)-C(1)-H(1B)	110.8
H(1A)-C(1)-H(1B)	108.9
C(3)-C(2)-C(7)	120.0
C(3)-C(2)-C(1)	132.8(2)
C(7)-C(2)-C(1)	107.2(2)
C(2)-C(3)-C(4)	120.0
C(2)-C(3)-H(3)	120.0
C(4)-C(3)-H(3)	120.0
C(5)-C(4)-C(3)	120.0
C(5)-C(4)-H(4)	120.0

C(3)-C(4)-H(4)	120.0
F(1)-C(5)-C(4)	120.1(2)
F(1)-C(5)-C(6)	119.9(2)
C(4)-C(5)-C(6)	120.0
C(7)-C(6)-C(5)	120.0
C(7)-C(6)-H(6)	120.0
C(5)-C(6)-H(6)	120.0
C(6)-C(7)-C(2)	120.0
C(6)-C(7)-N(1)	127.9(2)
C(2)-C(7)-N(1)	112.0(2)
N(1)-C(8)-C(9)	112.3(3)
N(1)-C(8)-H(8A)	109.2
C(9)-C(8)-H(8A)	109.2
N(1)-C(8)-H(8B)	109.2
C(9)-C(8)-H(8B)	109.2
H(8A)-C(8)-H(8B)	107.9
C(10)-C(9)-C(14)	120.0
C(10)-C(9)-C(8)	120.5(3)
C(14)-C(9)-C(8)	119.4(3)
C(11)-C(10)-C(9)	120.0
C(11)-C(10)-H(10)	120.0
C(9)-C(10)-H(10)	120.0
C(12)-C(11)-C(10)	120.0
C(12)-C(11)-H(11)	120.0
C(10)-C(11)-H(11)	120.0
C(11)-C(12)-C(13)	120.0
C(11)-C(12)-Br(1)	119.36(17)
C(13)-C(12)-Br(1)	120.62(17)
C(12)-C(13)-C(14)	120.0
C(12)-C(13)-H(13)	120.0
C(14)-C(13)-H(13)	120.0
C(13)-C(14)-C(9)	120.0
C(13)-C(14)-H(14)	120.0
C(9)-C(14)-H(14)	120.0

Symmetry transformations used to generate equivalent atoms:

Table 4. Anisotropic displacement parameters (A^2 x 10^3) for j3_a.
The anisotropic displacement factor exponent takes the form:
-2 pi^2 [h^2 a*^2 U11 + ... + 2 h k a* b* U12]

	U11	U22	U33	U23	U13	U12
Br(1)	74(1)	44(1)	104(1)	2(1)	38(1)	8(1)
F(1)	102(2)	47(2)	105(2)	14(1)	37(2)	0(1)
N(1)	56(2)	45(2)	61(2)	-2(2)	21(2)	-3(2)
O(1)	118(3)	56(2)	118(3)	7(2)	76(3)	-6(2)
C(1)	71(3)	71(3)	65(3)	-7(2)	28(2)	-5(2)
C(2)	50(2)	59(2)	46(2)	-8(2)	7(2)	1(2)
C(3)	58(3)	64(3)	53(2)	-16(2)	4(2)	14(2)
C(4)	68(3)	45(2)	59(3)	-7(2)	-1(2)	10(2)
C(5)	60(3)	46(2)	62(3)	4(2)	5(2)	1(2)
C(6)	55(2)	49(2)	56(3)	-3(2)	16(2)	4(2)
C(7)	48(2)	45(2)	47(2)	-8(2)	9(2)	-3(2)
C(8)	54(3)	47(2)	78(3)	0(2)	10(2)	-4(2)
C(9)	48(2)	46(2)	59(3)	7(2)	10(2)	-2(2)
C(10)	158(6)	46(3)	77(4)	-1(3)	-42(4)	15(3)
C(11)	152(6)	52(3)	75(3)	-4(3)	-36(4)	12(3)
C(12)	48(2)	46(2)	68(3)	7(2)	21(2)	3(2)
C(13)	104(4)	59(3)	119(5)	5(3)	-41(4)	24(3)
C(14)	92(4)	61(3)	102(4)	-5(3)	-38(3)	13(3)

Table 5. Hydrogen coordinates (x 10^4) and isotropic displacement parameters (A^2 x 10^3) for j3_a.

	x	y	z	U(eq)
H(1A)	6169	2422	2703	81
H(1B)	4844	2278	1526	81
H(3)	5220	333	2389	71
H(4)	3864	-686	3558	70
H(6)	1814	1528	5047	63
H(8A)	998	3043	4155	72
H(8B)	1202	3188	2763	72
H(10)	3338	4351	5405	119
H(11)	3326	5965	5751	117
H(13)	201	6316	2614	120
H(14)	214	4702	2267	108

Table 6. Torsion angles [deg] for j3_a.

C(7)-N(1)-O(1)-C(1)	-18.4(5)
C(8)-N(1)-O(1)-C(1)	-140.1(4)
N(1)-O(1)-C(1)-C(2)	16.7(5)
O(1)-C(1)-C(2)-C(3)	171.3(3)
O(1)-C(1)-C(2)-C(7)	-8.9(4)
C(7)-C(2)-C(3)-C(4)	0.0
C(1)-C(2)-C(3)-C(4)	179.8(4)
C(2)-C(3)-C(4)-C(5)	0.0
C(3)-C(4)-C(5)-F(1)	178.9(3)
C(3)-C(4)-C(5)-C(6)	0.0
F(1)-C(5)-C(6)-C(7)	-178.9(3)
C(4)-C(5)-C(6)-C(7)	0.0
C(5)-C(6)-C(7)-C(2)	0.0
C(5)-C(6)-C(7)-N(1)	-176.6(3)
C(3)-C(2)-C(7)-C(6)	0.0
C(1)-C(2)-C(7)-C(6)	-179.8(3)
C(3)-C(2)-C(7)-N(1)	177.1(3)
C(1)-C(2)-C(7)-N(1)	-2.7(3)
O(1)-N(1)-C(7)-C(6)	-170.5(2)
C(8)-N(1)-C(7)-C(6)	-55.8(4)
O(1)-N(1)-C(7)-C(2)	12.7(3)
C(8)-N(1)-C(7)-C(2)	127.4(3)
C(7)-N(1)-C(8)-C(9)	172.6(3)
O(1)-N(1)-C(8)-C(9)	-73.3(4)
N(1)-C(8)-C(9)-C(10)	-46.4(5)
N(1)-C(8)-C(9)-C(14)	137.3(3)
C(14)-C(9)-C(10)-C(11)	0.0
C(8)-C(9)-C(10)-C(11)	-176.3(3)
C(9)-C(10)-C(11)-C(12)	0.0
C(10)-C(11)-C(12)-C(13)	0.0
C(10)-C(11)-C(12)-Br(1)	178.5(2)
C(11)-C(12)-C(13)-C(14)	0.0
Br(1)-C(12)-C(13)-C(14)	-178.4(2)
C(12)-C(13)-C(14)-C(9)	0.0
C(10)-C(9)-C(14)-C(13)	0.0
C(8)-C(9)-C(14)-C(13)	176.3(3)

Symmetry transformations used to generate equivalent atoms:

D-H...A	d(D-H)	d(H...A)	d(D...A)	<(DHA)

References

(1) Bin, H.; Deng, L. Catalytic Asymmetric Synthesis of Trifluoromethylated γ-Amino Acids through the Umpolung Addition of Trifluoromethyl Imines to Carboxylic Acid Derivatives. *Agnew. Chem. Int. Ed.* **2018**, 57, 2233-2237. https://doi.org/10.1002/anie.201710915

(2) Fuerstner, C.; Riedl, B.; Ergueden, J.; Boes, F.; Schmidt, B.; Van Der Staay, F.; Schroeder, W.; Schlemmer, K.; Moriwaki, T.; Yoshida, N. New 3-Oxo-2,1-benzisoxazol-1 (3H)-carboxamides for the Treatment of CNS Diseases. German Patent DE19960917A1, **1999**.

(3) Niemeier, J. K.; Kjell, D. P. Hydrazine and Aqueous Hydrazine Solutions: Evaluating Safety in Chemical Processes. *Org. Process Res. Dev.* **2013**, 17, 1580-1590

(4) (a) Bruker AXS Inc. *APEX3 Crystallography Software Suite*, 2016, 5465 East Cheryl Parkway, Madison, WI 53711,USA; (b) Sheldrick, G.M. *A Short History of SHELX*, Acta Cryst. 2008, A64, 112-122; (c) Sheldrick, G.M. *Crystal structure refinement with SHELXL,* Acta Cryst. 2015, C71, 3-8.
